# Conserved miR164-targeted NAC genes negatively regulate drought resistance in rice

**DOI:** 10.1093/jxb/eru072

**Published:** 2014-03-06

**Authors:** Yujie Fang, Kabin Xie, Lizhong Xiong

**Affiliations:** National Key Laboratory of Crop Genetic Improvement, National Center of Plant Gene Research (Wuhan), Huazhong Agricultural University, Wuhan 430070, China

**Keywords:** Abiotic stress, miRNA, NAC, *Oryza sativa*, transactivation.

## Abstract

MicroRNAs constitute a large group of endogenous small RNAs of ~22 nt that emerge as vital regulators, mainly by targeting mRNAs for post-transcriptional repression. Previous studies have revealed that the miR164 family in *Arabidopsis* is comprised of three members which guide the cleavage of the mRNAs of five NAC genes to modulate developmental processes. However, the functions of the miR164-targeted NAC genes in crops are poorly deciphered. In this study, the conserved features of six miR164-targeted NAC genes (*OMTN1*–*OMTN6*) in rice are described, and evidence is provided that four of them confer a negative regulatory role in drought resistance. OMTN proteins have the characteristics of typical NAC transcriptional factors. The miR164 recognition sites of the *OMTN* genes are highly conserved in rice germplasms. Deletion of the recognition sites impaired the transactivation activity, indicating that the conserved recognition sites play a crucial role in maintaining the function of the OMTN proteins. The *OMTN* genes were responsive to abiotic stresses, and showed diverse spatio-temporal expression patterns in rice. Overexpression of *OMTN2*, *OMTN3*, *OMTN4*, and *OMTN6* in rice led to negative effects on drought resistance at the reproductive stage. The expression of numerous genes related to stress response, development, and metabolism was altered in *OMTN2*-, *OMTN3*-, *OMTN4*-, and *OMTN6*-overexpressing plants. Most of the up-regulated genes in the *OMTN*-overexpressing plants were down-regulated by drought stress. The results suggest that the conserved miR164-targeted NAC genes may be negative regulators of drought tolerance in rice, in addition to their reported roles in development.

## Summary

Six NAC transcription factor genes were found to be targeted by miR164 at a highly conserved region for transactivation activity and to regulate drought tolerance negatively at the reproductive stage in rice.

## Introduction

MicroRNAs (miRNAs) are endogenous small single-stranded non-coding RNAs that play vital regulatory roles in both animals and plants, mainly by promoting cleavage or translation inhibition of the targeting mRNAs (Bartel, 2004). miRNAs recognize their targets based on the sequence near-perfect complementarity principle (Rhoades *et al.*, 2002). Most miRNAs control their targets by suppressing their expression through transcript cleavage in a sequence-specific manner, inducing *trans*-acting small interfering RNA (ta-siRNA) or inhibiting the translation (Palatnik *et al.*, 2003; Brodersen *et al.*, 2008). A recent finding supported the theory that miRNAs can up-regulate the translation of target genes under certain conditions, whereas no similar miRNA-mediated regulation mechanisms have been reported in plants (Vasudevan *et al.*, 2007). Furthermore, miRNAs may cause epigenetic modifications including DNA and histone methylation to control their targets (Bao *et al.*, 2004; Khraiwesh *et al.*, 2010; Wu *et al.*, 2010).

The biological functions of miRNAs are intimately relevant to the functions of their target genes. Identification of the potential target genes therefore provides an effective and essential approach to investigate in depth the complex miRNA-mediated regulatory mechanisms. Early exploration for miRNA targets in plants mainly relied on the use of empirical parameters and algorithms deduced from known miRNA–target interactions using computational prediction in *Arabidopsis* (Rhoades *et al.*, 2002). In recent years, information about miRNA targets has been extensively enriched and updated in many plant species using *in silico* bioinformatics analysis and experimental validation including PAGE northern, 5′-RACE (rapid amplification of cDNA ends), and degradome sequencing (X.J. Wang *et al.*, 2004; Wu *et al.*, 2009; Lv *et al.*, 2010).

Tremendous research effort has demonstrated that miRNAs and their targets have regulatory effects on very diverse aspects throughout the entire life cycle in higher plants, such as plant growth, organ development, morphogenesis, signal transduction, and pathogen infection (Navarro *et al.*, 2006; He *et al.*, 2008; Lu and Huang, 2008; Sanan-Mishra *et al.*, 2009; D.H. Jeong *et al.*, 2010; Khraiwesh *et al.*, 2012). Compared with the ample studies reporting the roles of miRNAs and their targets in the morphogenesis and development of plants, much less has been reported about the roles of miRNAs and their targets in the environmental stress responses of plants. Abiotic stress seriously influences plant growth and development, and reduces crop yields worldwide. An enhanced understanding of the miRNA-guided regulation mechanisms responsible for abiotic stress adaptation may help unveil the regulatory networks of stress response and adaptation, and it may also help in designing new strategies to engineer plants with improved stress tolerance.

Emerging evidence suggests that miRNAs and their targets may serve as the main governing factors in response to various stresses, encompassing drought, salinity, extreme temperatures, abscisic acid (ABA)-mediated stress, nutrient homeostasis, UV-B radiation, hypoxia and oxidative stress, and even mechanical stress (Fujii *et al.*, 2005; Lu *et al.*, 2005; Reyes and Chua, 2007; Zhou *et al.*, 2007; Li *et al.*, 2008; Jia *et al.*, 2009; Li *et al.*, 2011; Xin *et al.*, 2010). In *Arabidopsis*, Li *et al.* (2008) reported that miR169a and miR169c were substantially down-regulated by drought stress, and functioned as crucial players in the regulation of the cognate target *NFYA5* at the post-transcriptional level (Li *et al.*, 2008). Zhao *et al.* (2009) identified that miR169g and miR169n, which also targeted an NF-YA gene, exhibited overlapping and distinct responses to drought and salt stresses. Reyes and Chua (2007) described a homeostatic mechanism of ABA-induced accumulation of miR159 to direct the transcript degradation of two positive regulators of ABA responses (MYB33 and MYB101), which desensitizes hormone signalling during the stress response. Previous studies reported that miR399 is strongly induced by low phosphate stress, and partially controls phosphate homeostasis through targeting a gene encoding a putative ubiquitin-conjugating enzyme E2-UBC24 (PHO2) in *Arabidopsis* (Fujii *et al.*, 2005; Bari *et al.*, 2006; Chiou *et al.*, 2006).

NAC (NAM, ATAF1/2, and CUC2) proteins constitute a large plant-specific transcription factor family with >100 members in both *Arabidopsis* and rice (Nuruzzaman *et al.*, 2010). In general, NAC proteins share a consensus NAC domain which consists of ~150 well-conserved amino acids in the N-terminus, and a diversified transcription regulation region in the C-terminus (Olsen *et al.*, 2005). In recent years, NAC proteins have been intensively investigated for their multiple roles in developmental programmes and environmental adaptations (Nakashima *et al.*, 2012). NAC transcription factors can be regulated by certain *cis*-acting elements and *trans*-acting factors on a transcriptional level, miRNAs on a post-transcriptional level, and on a post-translational regulation level encompassing phosphorylation, protein degradation, and dimerization (Xie *et al.*, 2002; Tran *et al.*, 2007; Jeong *et al.*, 2009; Kleinow *et al.*, 2009).

A number of NAC proteins have been reported for their roles in response to abiotic stresses (Puranik *et al.*, 2012; Nuruzzaman *et al.*, 2013). *Arabidopsis ANAC019/055/072* and *RD26* (*RESPONSIVE TO DEHYDRATION 26*) were reported to function in drought, salt, and ABA response (Fujita *et al.*, 2004; Tran *et al.*, 2004). NTL9 was found to mediate osmotic stress signalling in leaf senescence (Yoon *et al.*, 2008). LOV1 (LONG VEGETATIVE PHASE 1) was reported to be a regulator of cold response in *Arabidopsis* (S.Y. Yoo *et al.*, 2007). Recently, more and more rice NAC factors, such as SNAC1 (Hu *et al.*, 2006), SNAC2 (Nakashima *et al.*, 2007; Hu *et al.*, 2008), OsNAC5 (Sperotto *et al.*, 2009), ONAC045 (Zheng *et al.*, 2009), and OsNAC10 (J.S. Jeong *et al.*, 2010), were also documented to participate in stress responses. A rice stress-responsive NAC gene, *SNAC1*, confers drought resistance under field drought conditions by promoting stomatal closure (Hu *et al.*, 2006). Overexpression of *OsNAC10* driven by a root-specific promoter *RCc3* in rice also increased grain yield under field drought conditions (J.S. Jeong *et al.*, 2010).

Previous studies demonstrated that the miR164 family in *Arabidopsis* is comprised of three members (ath-miR164a/b/c) which guide the cleavage of the mRNAs of five NAC transcription factor genes (*CUC1/At3g15170*, *CUC2/At5g53950*, *NAC1/At1g56010*, *At5g07680*, and *At5g61430*) that are required for boundary establishment and maintenance, lateral root emergence, formation of vegetative and floral organs, and age-dependent cell death (Rhoades *et al.*, 2002; Laufs *et al.*, 2004; Mallory *et al.*, 2004; Guo *et al.*, 2005; Kim *et al.*, 2009). The *CUC1* and *CUC2* genes were initially found to be regulated by miR164 to constrain the expansion of the boundary domain (Laufs *et al.*, 2004). Expression of a miR164-resistant version of *CUC1* mRNA caused cotyledon orientation defects, reduction of rosette leaf petioles, dramatically misshapen rosette leaves, 1–4 extra petals, and one or two missing sepals in *Arabidopsis*; abolition of miR164 regulation of *CUC2* resulted in progressive enlargement of the boundary domain (Laufs *et al.*, 2004; Mallory *et al.*, 2004). Guo *et al.* (2005) subsequently reported that the late auxin-responsive miR164 expression provided a homeostatic mechanism to cleave *NAC1* mRNA to attenuate auxin signals for *Arabidopsis* lateral root development (Guo *et al.*, 2005). Recent studies have shed light on a trifurcate feed-forward pathway involving *ORE1/AtNAC2*, *miR164*, and *EIN2* for the regulation of age-dependent cell death in *Arabidopsis* (Kim *et al.*, 2009). Several studies have shown that miR164 may also be involved in response to abiotic and biotic stress in plants (Lu *et al.*, 2005, 2008; Bazzini *et al.*, 2009; Jia *et al.*, 2009, 2010; Li *et al.*, 2009; Xin *et al.*, 2010; Zhao *et al.*, 2012).

High-throughput sequencing revealed that the miR164 family in rice (*Oryza sativa* L.) has six members (osa-miR164a/b/c/d/e/f) (Sunkar *et al.*, 2008). However, the functions of the miR164 family and their target genes in rice or other cereal crops are poorly deciphered. This work focused on the characterization of *Oryza m*iR164-*t*argeted *N*AC (*OMTN*) genes. It was observed that most of the *OMTN* genes were differentially expressed under various abiotic stresses and phytohormone treatments. The miR164 recognition sites of the *OMTN* genes are highly conserved. Overexpression of *OMTN2*, *OMTN3*, *OMTN4*, and *OMTN6* caused increased drought sensitivity in transgenic rice plants. A large number of drought-responsive genes were found to be down-regulated in the transgenic plants. This study suggests that the OMTNs may act as negative regulators of drought tolerance in rice.

## Materials and methods

### Sequence analysis of the miR164 family and prediction of target genes

The mature sequences of the plant miR164 family were obtained from miRbase and aligned by CLUSTALX (Thompson *et al.*, 1997; Griffiths-Jones, 2006). The known rice open reading frame (ORF) sequences were downloaded from the TIGR Rice Genome Annotation Project Database (http://rice.plantbiology.msu.edu/, last accessed on 24 February 2014) and used for target gene prediction for miR164. The prediction was performed on the basis of near-perfect complementarity using a four-mismatch cut-off between miR164 and its target mRNA (Rhoades *et al.*, 2002; Jones-Rhoades and Bartel, 2004).

### Conservation analysis of the miR164-targeted sites of the *OMTN* genes

To elucidate the variation of the miR164 target sites of the target genes, 158 rice varieties were selected from a mini-core germplasm resource (Supplementary Table S1 available at *JXB* online). The DNA samples were extracted from the leaves of rice plants at the tilling stage using a CTAB (cetyltrimethylammonium bromide) method (Murray and Thompson, 1980). The regions covering the targeted sites were amplified by the gene-specific primers listed in Supplementary Table S2 (available at *JXB* online), and treated with *Exo*I/SAP. The purified PCR products were used as templates for sequencing. The sequencing procedure was carried out according to the manufacturer’s instructions (ABI 3730). The sequences derived from the sequencing analysis were aligned with CLUSTALX software.

### Constructs and transformation of rice

Full-length cDNAs of the miR64-targeted NAC genes were obtained from KOME (http://cdna01.dna.affrc.go.jp/cDNA/, last accessed 24 February 2014) or by real-time PCR (RT-PCR) from the sequenced *japonica* rice cultivar Nipponbare cDNA templates. To generate the *OMTN*-overexpression (OE) constructs, the sequence-confirmed fragments containing the ORFs of the *OMTN* genes were amplified by PCR with gene-specific primers (Supplementary Table S2 available at *JXB* online) and inserted into the pCAMBIA1301U (pU1301) vector under the control of a maize *ubiquitin1* promoter via an enzyme (*Kpn*I/*Bam*HI) digestion–ligation method. The constructs were transformed into Zhonghua11 (ZH11) (*O. sativa* L. ssp *japonica*) through the *Agrobacterium*-mediated transformation method (Lin and Zhang, 2005).

### Stress treatments

To verify the expression profiles of the miR164-targeted NAC genes under various abiotic stresses and phytohormone treatments, ZH11 seedlings were grown under normal conditions for ~3 weeks. Stress and phytohormone treatments were applied to the seedlings at the four-leaf stage. For drought stress, irrigation was withheld for 7 d. For high salinity treatment, the seedlings were irrigated with 200mM NaCl solution. For cold and heat stress, seedlings were transferred to a growth chamber at 4 °C and 42 °C, respectively. For phytohormone treatments, 0.1mM ABA, IAA (indoleacetic acid), and KT (kinetin) were sprayed on the leaves. Leaf samples were collected according to the designated time courses.

To investigate the spatio-temporal expression profile of the *OMTN* genes, seeds of ZH11 were grown under normal conditions. Eleven samples representing the major tissues and organs of rice during an entire life cycle were collected for quantitative expression level analysis.

To identify the performance of transgenic plants under drought stress treatment, positive transgenic plants were selected by germinating seeds on hygromycin-containing (50mg l^–1^) Murashige and Skoog (MS) medium, while wild-type (WT) and negative transgenic lines were germinated on normal MS medium. Drought stress testing at the panicle development stage (~2 weeks before flowering) was performed in a paddy field facilitated with a removable rainproof shelter. Drought stress was initiated and developed by stopping the supply of water until all of the leaves became rolled (wilted), and recovery was followed by re-irrigation.

### Quantification of gene expression

Total RNA was extracted from rice leaves with TRIzol reagent (Invitrogen, Carlsbad, CA, USA) and then digested with RNase-free DNase I (Invitrogen) to remove genomic DNA contamination. First-strand cDNA was synthesized with an oligo(dT)_15_ primer using Superscript III reverse transcriptase (Invitrogen) according to the manufacturer’s instructions. Transcript levels of the genes were detected by quantitative RT-PCR in an optical 96-well plate using the ABI PRISM 7500 Real-Time PCR System (Applied Biosystems, Foster City, CA, USA) with SYBR Premix^®^ Ex Taq™ (TAKARA, Dalian, China) according to the manufacturer’s handbook. The rice *Ubiquitin* gene (TIGR accession no. LOC_Os03g13170) was used as an internal control. The relative expression levels were examined as described previously (Livak and Schmittgen, 2001). Gene-specific primers designed for RT-PCR are listed in Supplementary Table S2 available at *JXB* online.

### RLM-RACE PCR

To detect the putative truncated mRNAs from the target genes at the miR164 cleavage sites, nested RACE PCRs were conducted using RNA ligase-mediated (RLM)-based reverse transcriptions without CIP and TAP treatment in conjunction with a GeneRacer kit (Invitrogen) according to the manufacturer’s instructions. The total RNA for RACE was obtained from the young panicles of rice. RACE and nested-RACE PCR were subsequently used for checking the truncation of *OMTN1* and *OMTN2*. PCR products were cloned using the pGEM-T easy ligation kit (Promega) with *Escherichia coli* Top10 competent cells (Invitrogen) and sequenced.

### Transient expression assay in rice protoplasts

To investigate the subcellular localization of the proteins encoded by the miR164 target genes, the *35S*:OMTN–GFP (green fluorescent protein) fusion constructs were produced by inserting the full ORFs of OMTN1, OMTN2, OMTN3, OMTN4, and OMTN6 into the pM999-35 vector. The gene-specific primers used for PCR amplification are listed in Supplementary Table S2 available at *JXB* online. Ghd7 was used as a nuclear marker (Xue *et al.*, 2008). Plasmids were extracted and purified using the Plasmid Midi Kit (QIAGEN, Germany) following the manufacturer’s manual. The *35S*:OMTN–GFP and *35S*:Ghd7–CFP (cyan fluorescent protein) plasmids were co-transformed into rice protoplasts according to the procedure described below. The florescence images were captured by using a confocal laser-scanning microscope (TCS SP2, Leica, Germany).

The rice protoplasts were isolated and transformed by following a method described previously (S.D. Yoo *et al.*, 2007) with minor modifications. Rice seeds were germinated on half-strength MS medium under light conditions for 3 d, and then transferred to dark conditions at 26 °C and grown for ~2 weeks. The sheath portion of the etiolated young seedlings was cut into 0.5mm pieces using sharp razors and these were immediately immersed in enzyme solution [0.6M mannitol, 10mM MES (pH 5.7), 1.5% cellulase RS, 0.75% macerozyme, 0.1% bovine serum albumin (BSA), 1mM CaC1_2_, and 50 μg ml^–1^ carbenicillin]. After incubation for 4h at 25–28 °C under dark conditions with gentle agitation (<80rpm), protoplasts were passed through two layers of nylon mesh (35 μm pore). The protoplasts were washed with 1vol. of W5 solution [154mM NaCl, 125mM CaC1_2_, 5mM KC1, 2mM MES (pH 5.7)] and collected by centrifugation at 100 *g* for 5min. After removing the supernatant, the protoplasts were re-suspended in 5ml of pre-chilled W5 solution and incubated on ice for 30min. The protoplasts were collected by centrifugation at 100 *g* for 5min and re-suspended in M solution [0.6M mannitol, 15mM MgC1_2_, 4mM MES (pH 5.7)]. After the addition of 10 μg of plasmid DNA, 120 μl of DNA uptake solution containing 40% (w/v) polyethylene glycol 3350, 0.6M mannitol, and 100mM CaC1_2_ was added to 100 μl of the protoplast solution to perform the transformation. The mixture was kept at room temperature for 20min and diluted with 5ml of W5 solution. The protoplasts were incubated at 28 °C for 16–20h under dark conditions.

### Biochemical assay in yeast

To examine the transactivation activity of the OMTNs, the full coding region and the C-terminal truncated cDNA fragments amplified with the OMTN-Y2H primers were fused in-frame to the yeast GAL4 DNA-binding domain and inserted into the pDONR221 entry vector through *att*B×*att*P (BP) recombination cloning, and then into the gateway destination vector pDEST32 using the *att*L×*att*R (LR) reaction (Invitrogen). The primers used for PCR amplification are listed in Supplementary Table S2 available at *JXB* online. The pDEST32-OMTN constructs were co-transformed with the pEXP-AD502 vector into the yeast strain MaV203. The transformed yeast cells were spread on a synthetic complete selection medium lacking leucine and tryptophan (SD/–Leu/–Trp) and incubated for 3 d. The colonies which appeared were picked to perform the colony-lift assay (β-gal assay) according to the manufacturer’s instructions (Invitrogen).

For the yeast one-hybrid assay, the promoter region of *OsERD1* containing the NACRS and NDBS *cis*-elements was inserted into the pHIS2 reporter vector. The ORFs of the OMTNs were fused to the GAL4 activation domain in the pGAD7-Rec2 vector (Clontech, Palo Alto, CA, USA), and then co-transformed with the pHIS2-*OsERD1* reporter construct into the yeast strain Y187. The primers used for the amplification of the ORFs of the OMTNs are listed in Supplementary Table S2 available at *JXB* online. The transformants were grown on a synthetic complete selection medium lacking leucine and tryptophan, and were further cultured on a nutrient-deficient medium lacking leucine, tryptophan, and and histidine (SD/–Leu/–Trp/–His) containing 30mM 3-AT (3-amino-1,2,4-triazole). The DNA–protein interactions were verified by the growth performance of the transformants on SD/–Leu/–Trp and SD/–Leu/–Trp/–His containing 3-AT. This particular procedure is referred to in the manuals pertaining to the ProQuest Two-Hybrid System (Invitrogen) and Matchmaker one-hybrid system (Clontech), respectively. The primers used for construction of the mutated forms of the *OMTN* genes are listed in Supplementary Table S2 (available at *JXB* online).

### Microarray analysis

For each of the four *OMTN* genes (*OMTN2*, *OMTN3*, *OMTN4*, and *OMTN6*), progeny of two independent overexpressing transgenic plants were selected for microarray analysis. Leaves of 1-month-old plants grown under normal growth conditions were collected from the overexpression and WT plants (each with two independent biological replicates). RNA samples were submitted for microarray hybridization after the detection of the *OMTN* transcript level by quantitative RT-PCR. Chip hybridization and data processing were implemented complying with the standard protocol of the Affymetrix Gene Chip service (CapitalBio, Beijing, China).

The differentially expressed genes (up- or down-regulated) between the overexpression transgenic and WT plants were analysed with the MAS 3.0 molecule annotation system (http://bioinfo.capitalbio.com/mas3/, last accessed 24 February 2014) and MapMan.

## Results

### Comparison of the miR164 family in plants

**Fig. 1.**
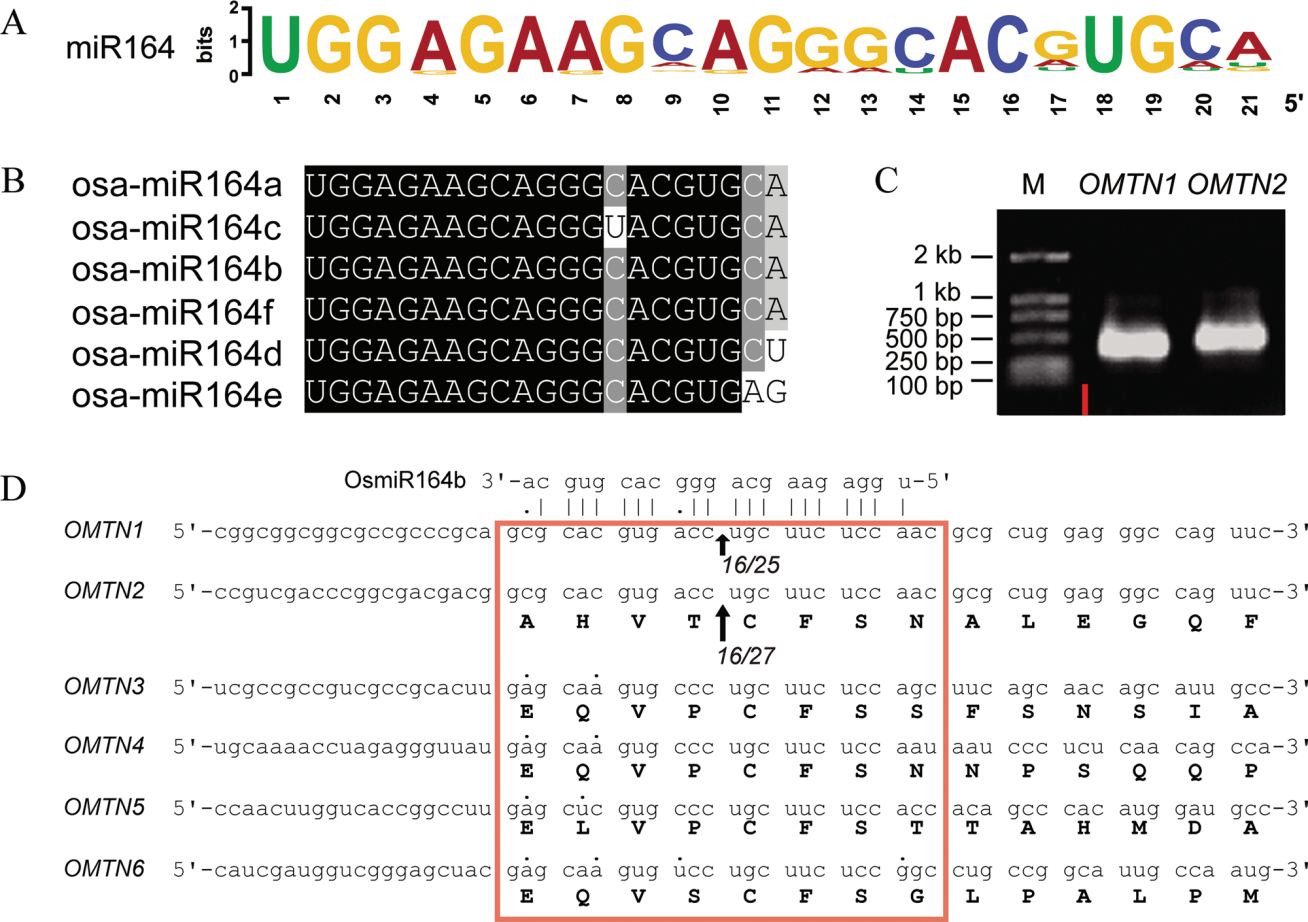
Mature miR164 sequence analysis in plants and validation of the mRNA cleavage sites of the rice miR164 target genes by RLM-RACE. (A) Sequence LOGO view of the mature miR164 sequences based on 92 plant miR164 sequences. The height of the letter at each position represents the degree of conservation. (B) Alignment of the rice mature miR164 sequences. (C) The 5′-RACE products for the predicted target genes *OMTN1* and *OMTN2* amplified by PCR are shown in the agarose gel. M, DNA marker. (D) Mapping of the *OMTN1* and *OMTN2* mRNA cleavage sites by RLM-RACE. The cleavage sites are indicated by arrows and the terminated mRNA ratios are shown at the bottom of the arrows. (This figure is available in colour at *JXB* online.)

There have been some reports on miR164 and the targeted genes in *Arabidopsis*; however, the function of the rice miR164 and its target genes is still largely unknown. To increase the understanding of miR164 and its target genes in rice, a sequence analysis of all known plant miR164s was first conducted. A total of 92 members of the miR164 family were retrieved from 24 plant species in the miRBase (version 20.0, http://www.mirbase.org, last accessed 24 February 2014). The number of miR164 family members ranges from one (such as tae-miR164 from *Triticum aestivum*) to 11 (such as the gma-miR164a–k from *Glycine max*) in different plant species. Different members in the same plant species are encoded by different gene loci, of which the precursors vary, but the mature sequences were identical or highly similar. Among the 92 members, 59 share the same mature sequence (5′-UGGAGAAGCAGGGCACGUGCA-3′, regarded as the standard mature sequence of miR164, Fig. 1A), while other members showed 1–5 nucleotide differences in their mature sequences when compared with the standard sequence, respectively. The differential nucleotides were located in the fourth, seventh, ninth, 10th, 12th, 13th, 14th, 17th, 20th, and 21st positions of the mature sequence.

MiR164 is presumably encoded by six genomic loci (miR164a–miR164f) in rice, and yet only miR164b is supported with transcript evidence. The mature sequences of miR164a, miR164b, and miR164f consistently match the standard sequence. However, miR164c and miR164d have a differentiated nucleotide compared with the standard mature sequence (the 13th base of the mature miR164c sequence is U, and the 3′ end of the mature miR164d sequence is U). Moreover, there are two nucleotides which are different between the mature sequence of miR164e and the conserved sequence (the 3′ end of the mature miR164e sequence is AG) (Fig. 1B).

### Prediction and validation of miR164-targeted genes in rice

To date, all the miR164-targeted genes identified from *Arabidopsis* belong to the NAC gene family. To identify miR164-targeted genes in rice, a search was made for rice mRNA sequences which contained complementary sequences (with no more than four mismatches) to the mature osamiR164 sequences based on the near-perfect complementarity principle and the criteria described in previous studies (Rhoades *et al.*, 2002; Jones-Rhoades and Bartel, 2004). Six NAC genes (Os02g36880, Os04g38720, Os12g41680, Os06g46270, Os06g23650, and Os08g10080) containing the osa-miR164 complementary sites were predicted to be the putative targets and designated as *OMTN1*–*OMTN6* in this study (Supplementary Table S3 available at *JXB* online). The miR164-binding regions (miR164BRs) of *OMTN1–OMTN6* are located downstream of the NAC domain in the coding regions, and the miR164BR-encoded amino acid sequences are highly conserved compared with the ath-miR164-targeted NAC genes.

With the rapid development of deep sequencing and degradome sequencing technology, three additional osa-miR164 target genes (*OMT7–OMT9*) which are not from the NAC family were predicted previously (Supplementary Table S3 available at *JXB* online) (Li *et al.*, 2010; Zhou *et al.*, 2010). Two of them were predicted to encode phytanoyl-CoA dioxygenases (Li *et al.*, 2010), and the third one was predicted to encode a phytosulphokine precursor (Zhou *et al.*, 2010). There are two mismatches between the mRNA sequences of the *OMTN1–OMTN5* genes and the mature miR164b sequence (located at the 13th and 21st nucleotides for *OMTN1* and *OMTN2*, the 18th and 21st for *OMTN3* and *OMTN4*, and the 17th and 21st for *OMTN5*, respectively), whereas four mismatches exist between the mRNA sequence of the *OMTN6* gene and the mature miR164b sequence (located at the first, 13th, 17th, and 21st nucleotides of the mature miR164b sequence) (Fig. 1D). The locations of the target sites in *OMT7* and *OMT8* were identified in the 3′-untranslated region (UTR) (Li *et al.*, 2010), whereas the target sites of *OMT9* were documented to be located in the ORF region (Zhou *et al.*, 2010).

To verify that the *OMTN* genes are direct targets of miR164, two genes (*OMTN1* and *OMTN2*) were selected in order to examine the cleavage sites by RLM-RACE analysis. The cleavage products generated by miR164 processing of the mRNA fragments of *OMTN1* and *OMTN2* were successfully detected (Fig. 1C). Sequence analysis of 24 independent cDNA clones suggested that the cleavage sites were located in the middle of the miR164–*OMTN* base-pairing interaction regions corresponding to the 10th nucleotide position of the mature miR164 sequence (Fig. 1D). This result is identical to the cleavage positions of the target mRNAs directed by miR164 and documented in *Arabidopsis* previously (Guo *et al.*, 2005).

**Fig. 2.**
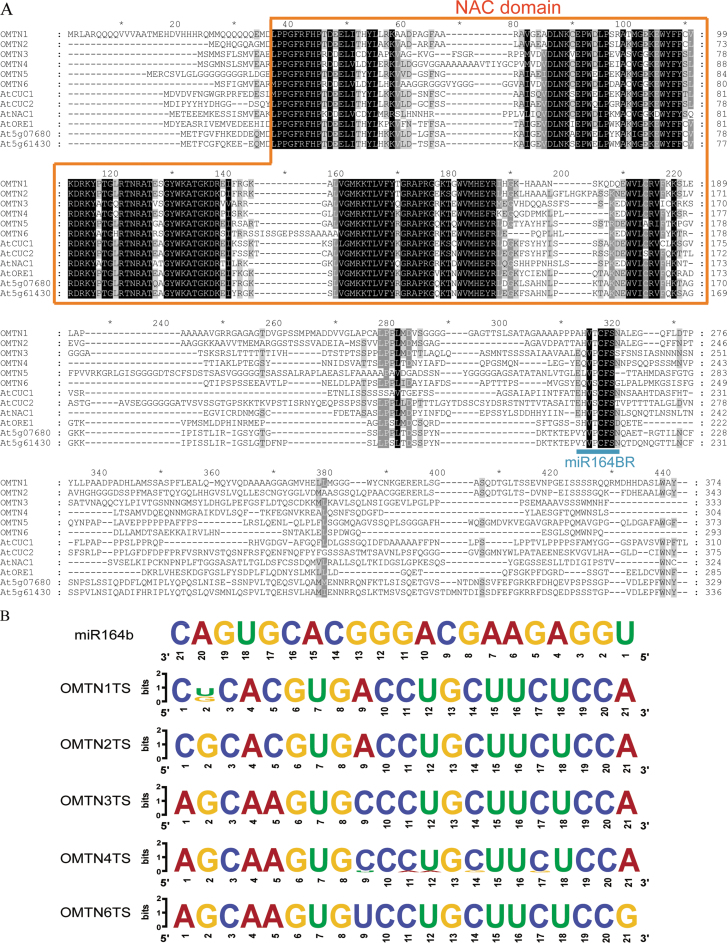
Sequence analysis of the *OMTN* genes. (A) Multi-sequence alignments of the miR164 NAC target genes in rice and *Arabidopsis*. NAC domains of the target genes are indicated by a block diagram, and the miR164BRs (miR164-binding regions) are labelled by an underline. (B) Sequence LOGO view of the consensus miR164 target sites of the *OMTN* genes based on the sequences derived from a mini-core rice germplasm collection. The height of the letter at each position represents the degree of conservation. TS, target site. (This figure is available in colour at *JXB* online.)

Comparison of the protein sequences of the miR164-targeted NAC genes in rice and *Arabidopsis* revealed that the N-terminal NAC domains of the proteins are highly conserved, while the C-terminal sequences showed considerable variation (Fig. 2A). However, the amino acid sequences corresponding to the miR164BR, which is located in the C-terminus, are also highly conserved (Fig. 2A).

### The miR164-targeted sites of the *OMTN* genes are highly conserved

Although the mature sequences of miR164 are highly conserved, it was decided to investigate if the miR164-targeted sites in the *OMTN* genes have any natural variations in rice germplasms since such variations may be associated with important biological functions of the target genes.

A total of 158 rice varieties were selected from a mini-core collection of germplasm resources for this analysis. Genomic DNAs of 158 varieties were used as PCR templates to amplify the fragments containing the miR164-targeted sites of the *OMTN* genes. Eventually 135, 138, 141, 122, and 154 valid sequences were obtained for the targeted sites of *OMTN1*, *OMTN2*, *OMTN3*, *OMTN4*, and *OMTN6*, respectively. Sequence alignment revealed that the miR164-targeted sites of *OMTN2*, *OMTN3*, and *OMTN6* were completely conserved among the amplified sequences. However, an SNP (G/T) was found in the miR164-targeted sites of *OMTN1* at the position corresponding to the 20th nucleotide of the mature miR164 sequence, and the T allele accounts for the majority. Moreover, SNPs were present in the miR164-targeted sites of *OMTN4* at the positions corresponding to the fifth (C/G), eighth (C/G), 10th (T/A), 11th (C/A), and 13th (C/T) nucleotides of the mature miR164 sequence, respectively.

Plant miRNAs recognize their target sites following a few principles: mismatches between mature miRNAs and the targets are <4 (U:U pairs recorded as 0.5 mismatches) in most cases; and <1 mismatch is allowed to exist in the region from the second to the eighth nucleotides of the mature miRNA since this region is crucial for the recognition of miRNAs and their targets. Among the tested varieties, no SNP was found in this critical recognition region of the *OMTN* genes except for the sequence from the landrace rice Hongkezhenuo (Fig. 2B). Two nucleotides are different (mismatched) in the critical recognition region of *OMTN4* in Hongkezhenuo, and these two SNPs together with the other three SNPs in the target site of *OMTN4* may cause differential regulation by miR164 in Hongkezhenuo. Other SNPs in the target sites resulting in 2–4 mismatches may not affect the recognition and regulation of *OMTN* genes by miR164 according to the rule of miRNA recognition (Fig. 2B). These results suggested that the miR164-targeted sites in the O*MTN* genes are generally highly conserved in rice germplasms.

### The OMTNs are typical NAC transcription factors

**Fig. 3.**
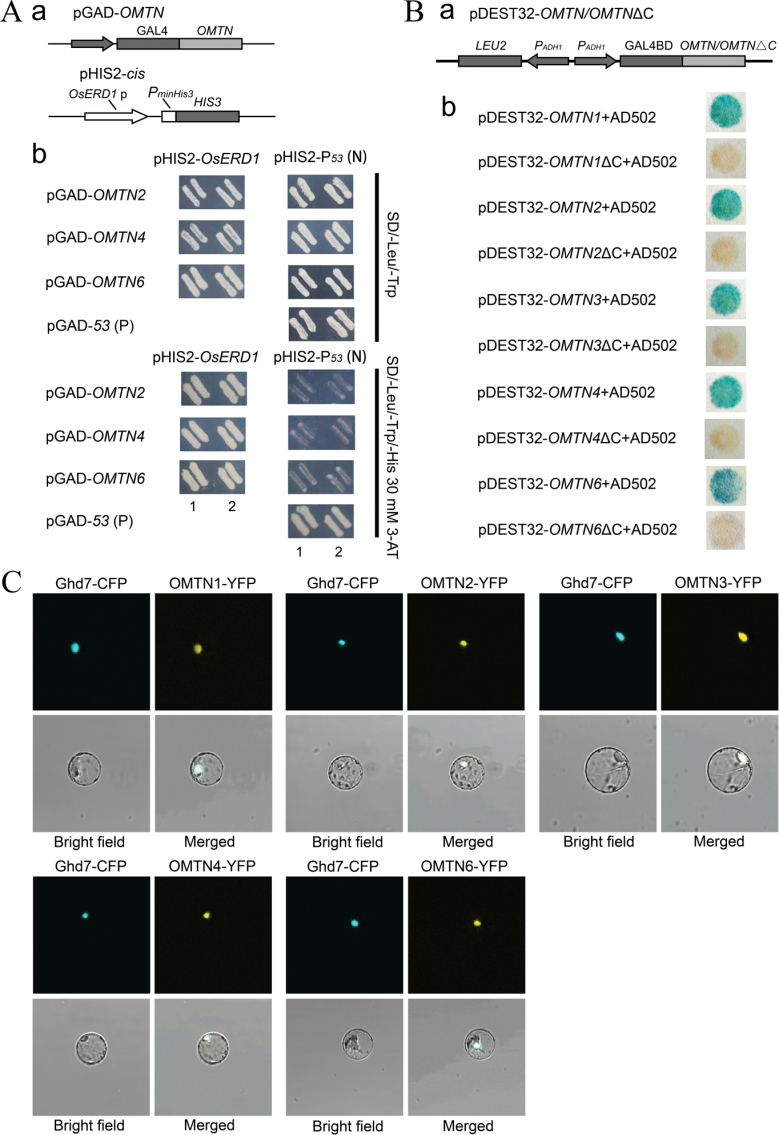
Transcription factor characteristics identified in the OMTN proteins. (A) DNA binding activity analysis of the OMTN proteins by yeast one-hybrid assay. (a) The schematic structure of the constructs for yeast one-hybrid assay. (b) pGAD-*OMTN* and the reporter constructs were co-transformed into the yeast strain Y187, and the transformants were examined by their growth performance on SD/–Leu/–Trp medium and on SD/–Leu/–Trp/–His medium containing 30 mmol l^–1^ 3-AT. pGAD-*OMTN* was co-transformed with pHIS2-P_*53*_ as a positive control (P), and pGAD-*53* was co-transformed with pHIS2-P_*53*_ as a negative control (N). Labels 1 and 2 indicate two independent transformants for each transformation event. Note that the results for OMTN1 and OMTN3 have been presented in a previous study (Fang *et al.*, 2008). (B) Transactivation activity analysis of the OMTN proteins by yeast two-hybrid. (a) The schematic structure of the OMTN fusion constructs. (b) The full-length C-terminal truncated OMTN proteins were fused to the GAL4-binding domain (GAL4 BD) and co-transformed with the pEXP-AD502 plasmid into the yeast strain MaV203, and a β-gal assay was performed to examine the transactivation activity. (C) Subcellular localization of the OMTN proteins in rice protoplasts. Ghd7–CFP and OMTN–GFP were co-transformed into etiolated shoot protoplasts of rice. Ghd7–CFP was used as a nuclear marker. (This figure is available in colour at *JXB* online.)

The features of OMTNs as putative transcription factors were further examined. To examine whether the OMTNs have DNA binding activity, the full-length coding sequences of the *OMTN* genes were fused with the GAL4 activation domain on the pGADT7-Rec2 vector (named as pGADT7-OMTN). Meanwhile, the promoter region (containing a CDBS element) of *OsERD1* was constructed into the pHIS2 vector (named as pHIS2-*cis*). The pGADT7-OMTN plasmids were co-transformed with pHIS2-*cis* into the yeast strain Y187. The results showed that only the co-transformed clones of pHIS2-*cis* and pGADT7-OMTN as well as the positive control (pGAD-*53*+pHIS2-P_*53*_) maintained normal growth states on SD/–Leu/–Trp/–His medium with the presence of 30mM 3-AT (Fig. 3A). The results indicated that the OMTNs can recognize and bind to the corresponding elements in the promoter region of *OsERD1*, and then induce the downstream *HIS3* reporter gene.

The Invitrogen yeast two-hybrid system was used for the transactivation assay of the OMTNs. We generated constructs by fusing full-length or C-terminal truncated OMTN fragments with the GAL4 DNA-binding domain located on the pDEST32 vector through a recombination reaction (named as pDEST32-OMTN). The pDEST32-OMTN constructs were co-transformed with the pEXP-AD502 vector into the yeast strain MaV203, and the monoclonal transformants were then picked for a β-gal assay. As shown in Fig. 3B, the full-length OMTN proteins have transactivation activities, and loss of the C-terminal fragments abolished the activation of the expression of the *LacZ* reporter gene. These results implied that the full-length OMTNs are putative transcriptional activators, and the C-terminal region is critical for the transactivation activity of OMTN proteins.

In general, transcription factors are proposed to be nuclear located. However, a few of the NAC proteins were reported to be first anchored to the plasma membrane or endoplasmic reticulum membrane via an α-helical transmembrane, and then to be imported to nuclei under specific conditions or the action of certain proteases. To determine whether OMTNs are directly targeted to the nucleus, the subcellular locations of the OMTN proteins were analysed using transient expression in a rice protoplast system. OMTNs were fused in-frame to the N-terminus of yellow fluorescent protein (YFP) and co-expressed with CFP-tagged Ghd7 in rice protoplasts. Ghd7–CFP was used as a positive control since Ghd7 was verified as a nuclear protein in rice (Xue *et al.*, 2008). The yellow fluorescence generated by OMTN–YFP was distributed in the same area as that of the cyan fluorescence generated by Ghd7–CFP, suggesting that OMTNs are nuclear proteins (Fig. 3C). These results together indicate that OMTNs are typical NAC transcription factors.

### The miR164-targeted sites of the OMTNs are indispensable for transactivation

**Fig. 4.**
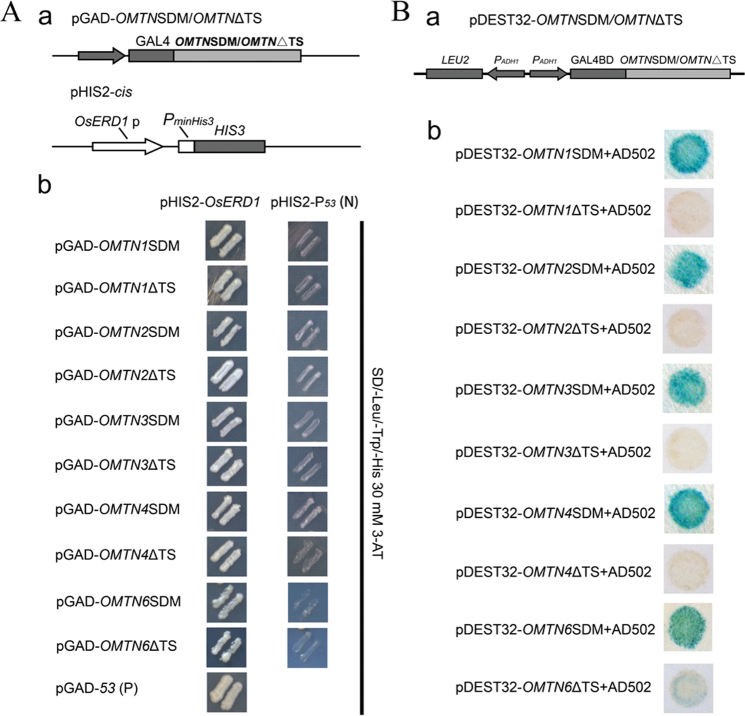
Functional analysis of the miR164 target sites of the *OMTN* genes. (A) DNA binding activity analysis of OMTNSDM/OMTNΔTS. (a) The schematic structure of the constructs for yeast one-hybrid assay. (b) Growth performance of the transformants on the SD/–Leu/–Trp/–His medium containing 30 mmol l^–1^ 3-AT. pGAD-*OMTN*SDM/*OMTN*ΔTS was co-transformed with pHIS2-P_*53*_ as a positive control (P), and pGAD-*53* was co-transformed pHIS2-P_*53*_ as a negative control (N). SDM, site-directed mutagenesis; ΔTS, target site deletion. (B) Transactivation activity analysis of OMTNSDM/OMTNΔTS. (a) The schematic structure of the OMTNSDM/OMTNΔTS fusion constructs. (b) The site-directed mutated or the target site-deleted OMTN proteins were fused to the GAL4-binding domain (GAL4 BD) and co-transformed with the pEXP-AD502 plasmid into the yeast strain MaV203, and a β-gal assay was performed to examine the transactivation activity. SDM, site-directed mutagenesis; ΔTS, target site deletion. (This figure is available in colour at *JXB* online.)

The conservation of target sites in the *OMTN* genes implied that the target sites may be important for the functions of the OMTNs as transcription factors. To test this hypothesis, target site mutation or deletion forms were constructed to test the DNA binding and transactivation activities in a yeast system. The results indicated that the point mutations of the target sites affect neither DNA binding nor transactivation activity of the mutated OMTN proteins in yeast (Fig. 4A). Deletion of the target sites did not affect the DNA binding activity, but the deletion abolished or strongly impaired the transactivition activity (Fig. 4B). These results suggested that the amino acid sequence encoded by the target sites is indispensable for the transactivition activity of the OMTN proteins.

### Expression profiles of the *OMTN* genes

In a microarray expression profiling analysis of rice seedlings under abiotic stresses (Zhou *et al.*, 2007), it was noticed that some of the *OMTN* genes were responsive to various abiotic stresses. To elucidate further the expression pattern of the *OMTN* genes under abiotic stresses, qPCR was performed to monitor the expression levels of the *OMTN* genes under various abiotic stresses and phytohormone treatments. *OMTN5* was not included in this analysis because it exhibited an extremely low background expression level and the corresponding full-length cDNA clone was absent in the KOME database (http://cdna01.dna.affrc.go.jp/cDNA/).

**Fig. 5.**
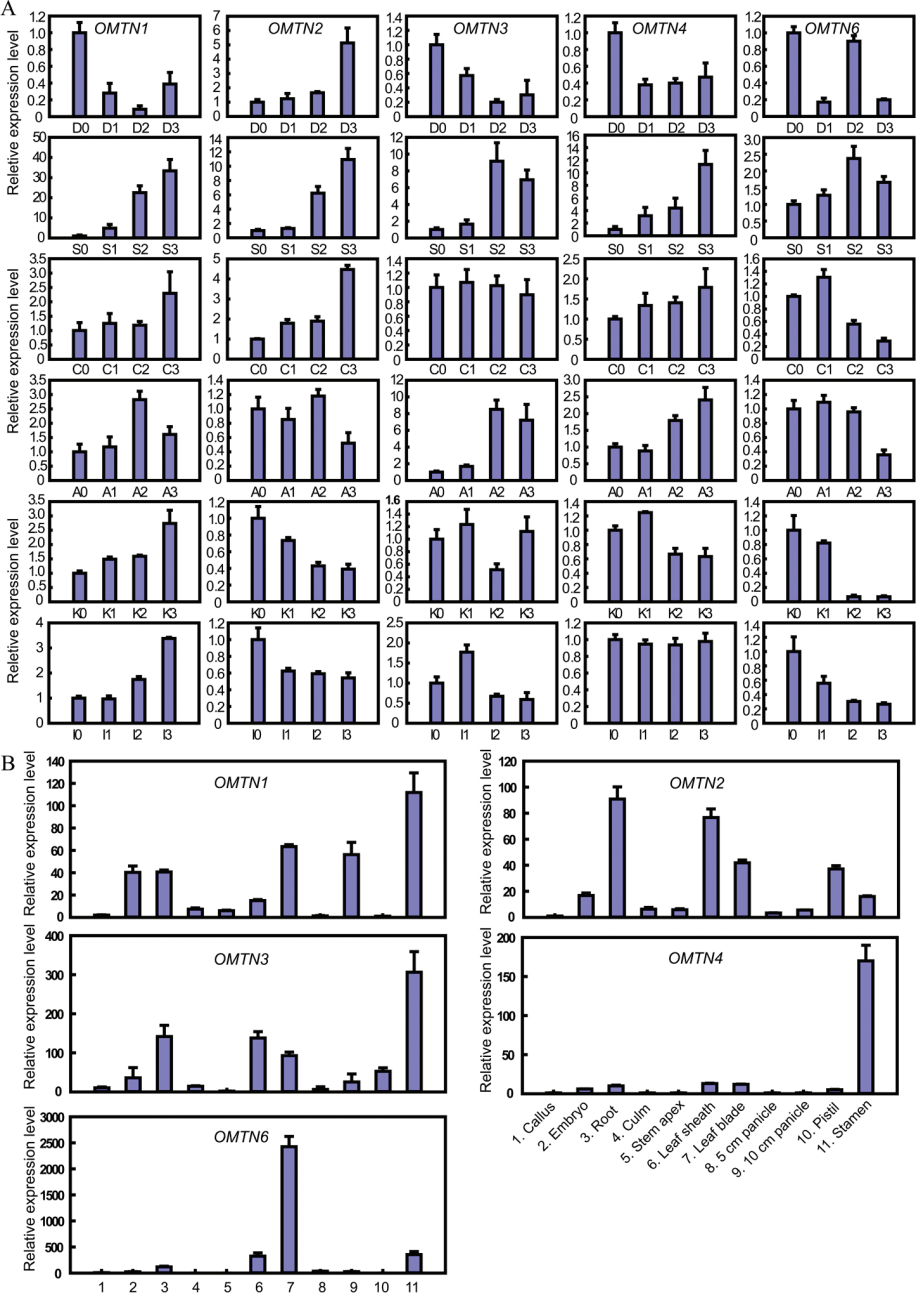
Expression profiles of the *OMTN* genes. (A) Expression of the *OMTN* genes under various abiotic stresses and phytohormone treatments. Four-leaf stage seedlings were subjected to various abiotic stresses and phytohormone treatments. D, drought (growth without water supply; D0, D1, D2, and D3 indicate 0, 1, 3, and 5 d after drought stress, respectively); S, salt (200 mmol l^–1^ NaCl; S0, S1, S2, and S3 indicate 0, 1, 6, and 12h after salt stress, respectively); C, cold (4 °C; C0, C1, C2, and C3 indicate 0, 1, 3, and 10h after cold stress, respectively); A, ABA (100 μM ABA; A0, A1, A2, and A3 indicate 0, 0.5, 3, and 6h after ABA treatment, respectively); K, KT (100 μM KT; K0, K1, K2, and K3 indicate 0, 0.5, 3, and 12h after KT treatment, respectively); I, IAA (100 μM IAA; I0, I1, I2, and I3 indicate 0, 0.5, 3, and 6h after IAA treatment, respectively). Error bars indicate the SE based on three technical replicates. (B) Spatio-temporal expression patterns of the *OMTN* genes. Error bars indicate the SE based on three technical replicates. (This figure is available in colour at *JXB* online.)

The results indicated that the expression levels of *OMTN1*, *OMTN3*, *OMTN4*, and *OMTN6* were strikingly reduced under drought stress conditions (Fig. 5A). Under high salinity stress, the expression levels of the five *OMTN* genes detected were increased. Under cold stress, the expression levels of *OMTN1* and *OMTN2* were increased, while the expression levels of the other three genes (*OMTN3*, *OMTN4*, and *OMTN6*) did not change significantly. Three genes (*OMTN1*, *OMTN3*, and *OMTN4*) were induced by ABA treatment. Interestingly, the changes in expression of the *OMTN* genes showed very similar trends under KT and IAA treatments (Fig. 5A).

In order to examine the spatio-temporal expression patterns of the five *OMTN* genes, 11 tissues/organs [callus, embryo, root, culm, stem apex, leaf sheath, leaf blade, panicle (5cm and 10cm), pistil, and stamen] of rice ZH11 grown under normal growth conditions were sampled for qPCR. The results demonstrated that the *OMTN* genes were ubiquitously detected in all of the rice tissues/organs with diverse expression patterns (Fig. 5B). *OMTN1* exhibited a higher expression level in stamen, leave blade, embryo, root, and panicle than in the other tissues/organs. *OMTN2* exhibited higher expression levels in root, leaf, and pistil, while *OMTN3* showed high expression levels in stamen, root, and leaf. Notably, *OMTN4* and *OMTN6* exhibited particularly high levels of expression in stamen and leaf blade, respectively. Such distinct spatio-temporal expression patterns of the *OMTN* genes implied that they may have diverse biological functions during different developmental stages in various tissues and organs of rice.

### *OMTN*-OE transgenic plants are sensitive to drought stress at the reproductive stage

**Fig. 6.**
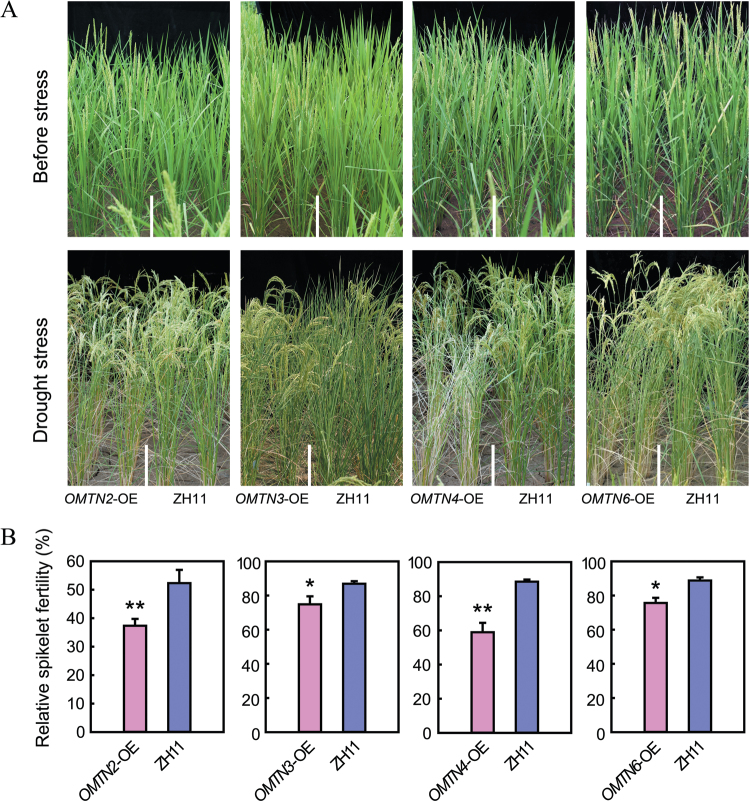
Enhanced drought sensitivity of the *OMTN*-OE transgenic plants at the reproductive stage. (A) Phenotype of the *OMTN2*-OE, *OMTN3*-OE, *OMTN4*-OE, *OMTN6*-OE, and ZH11 plants under drought stress at the reproductive stage. (B) Relative spikelet fertility of the *OMTN2*-OE, *OMTN3*-OE, *OMTN4*-OE, *OMTN6*-OE, and ZH11 plants under drought stress conditions at the reproductive stage. Data represent the mean ±SE (*n*=8). *P<0.05, *t*-test; **P<0.01, *t*-test.

The stress-responsive expression pattern prompted us to investigate the effect of *OMTN* overexpression on stress resistance. The full-length cDNAs of the *OMTN* genes driven by the ubiquitin promoter were transformed into the *japonica* cultivar ZH11, and the transgenic plants were subjected to stress testing. The *OMTN2*-, *OMTN3*-, *OMTN4*-, and *OMTN6*-OE transgenic plants showed increased sensitivity to drought stress at the reproductive stage (Fig. 6). The transgenic plants showed earlier leaf rolling and wilting compared with the WT control during the process of drought stress (Fig. 6A). After exposure to severe drought stress conditions, the relative spikelet fertility was significantly lower in the *OMTN2*-, *OMTN3*-, *OMTN4*-, and *OMTN6*-OE transgenic plants than in the WT plants (Fig. 6B). Nevertheless, the overexpression plants showed no obvious alterations in their tolerance to other stresses such as salinity and cold. These results suggested that *OMTN2*, *OMTN3*, *OMTN4*, and *OMTN6* may have negative roles in regulating drought resistance at the reproductive stage. An attempt was made to examine if suppression of the *OMTN* genes had any effects on drought resistance. However, the *OMTN*-RNAi (RNA interference) transgenic plants showed severe abnormal phenotypes such as twisted leaves and fusion organs (Supplementary Fig. S1 available at *JXB* online), which were very similar to the phenotypes exhibited by the miR164-OE plants (unpublished data), and therefore the abnormal *OMTN*-RNAi plants were not suitable for stress tolerance testing at later vegetative and reproductive stages. The *OMTN*-RNAi plants were further tested for dehydration stress [15% polyethylene glycol (PEG) 6000] tolerance at the early seedling stage at which the developmental defects were not visible. The *OMTN4*- and *OMTN6*-RNAi plants showed decreased sensitivity to the PEG treatment and accumulated less H_2_O_2_ based on 3,3′-diaminobenzidine tetrahydrochloride (DAB) staining (Supplementary Fig. S2 available at *JXB* online), which further supports the results of *OMTN4*-OE and *OMTN6*-OE with increased drought sensitivity.

### Expression profiles in the *OMTN*-OE transgenic plants

**Fig. 7.**
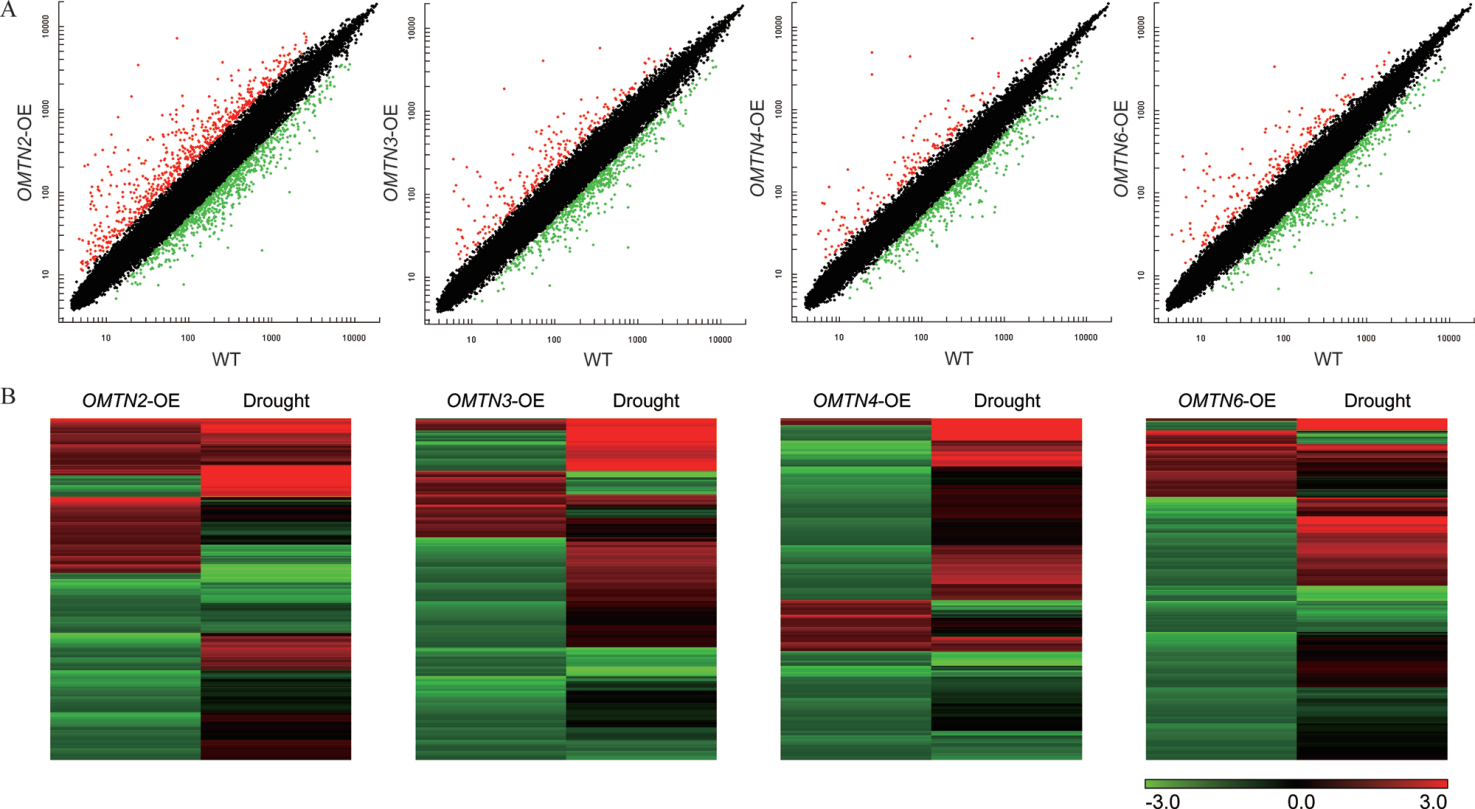
Whole-genome chip analysis of the *OMTN*-OE transgenic plants. (A) Scatter plots of the expression profiles of whole-genome genes in the *OMTN2*-, *OMTN3*-, *OMTN4*-, and *OMTN6*-OE transgenic plants compared with the WT. The *x*- and *y*-axes indicate the chip hybridization signals in the WT and *OMTN*-OE plants, respectively. The red and green dots indicate the probe sets with signal ratios of *OMTN*-OE/WT >2 and <0.5, respectively. OE indicates overexpression. (B) Drought-responsive patterns of the differentially expressed genes in the *OMTN*-OE transgenic plants. The drought expression profile data of the genes are based on the GEO public chip data at the NCBI (http://www.ncbi.nlm.nih.gov/geo, accession no. GSE6901).

To reveal possible molecular mechanisms for the increased drought sensitivity of the *OMTN*-OE rice, genome-wide expression profiling of *OMTN2*-. *OMTN3*-, *OMTN4*-, and *OMTN6*-OE plants was conducted in comparison with the WT control using Affymetrix GeneChip (Fig. 7A). The descendants of two independent transgenic plants were examined for each overexpressor, and a 2-fold change in both transgenic plants was taken as a threshold to determine the differentially regulated genes in the overexpressors. A large number of genes showed significant expression changes in the *OMTN* overexpressors, and significantly more genes were down-regulated than were up-regulated. The genes whose transcript abundance was significantly changed are provided in Supplementary Table S4–S7 available at *JXB* online.

**Fig. 8.**
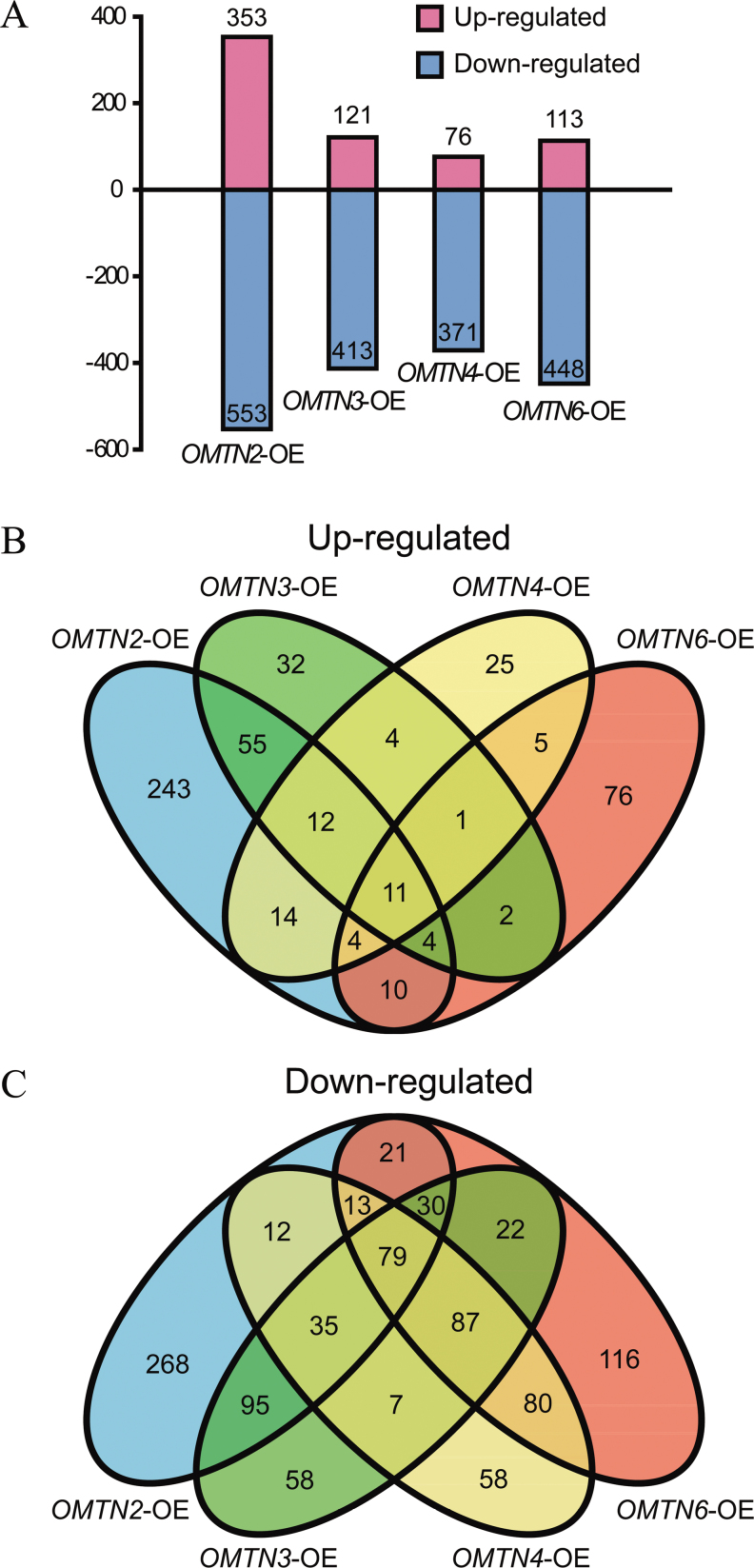
Significantly regulated genes in the *OMTN* overexpressors. (A) Distribution of the differentially expressed genes in the *OMTN*-OE plants. Terms on the horizontal axis represent the *OMTN*-OE materials, and the numbers on the vertical axis represent the numbers of up- and down-regulated genes. (B) Venn diagram analysis of the up-regulated genes in the *OMTN*-OE plants. (C) Venn diagram analysis of the down-regulated genes in the *OMTN*-OE plants. (This figure is available in colour at *JXB* online.)

Compared with the WT, a total of 353, 121, 76, and 113 genes were up-regulated, whereas 553, 413, 371, and 448 genes were down-regulated in the *OMTN2*-, *OMTN3*-, *OMTN4*-, and *OMTN6*-OE plants, respectively (Fig. 8A). It was observed that a considerable portion of the genes with significant changes in the transcript abundance in the *OMTN*-OE plants were also stress responsive based on the rice microarray under stress conditions in the NCBI GEO database (http://www.ncbi.nlm.nih.gov/geo, accession number: GSE6901) (Supplementary Table S4–S7 available at *JXB* online). Notably, cluster analysis revealed that most of the up-regulated genes in the *OMTN* overexpressors were down-regulated by drought stress, while most of the down-regulated genes in the *OMTN* overexpressors are up-regulated by drought stress conditions (Fig. 7B). These results suggested that overexpression of the *OMTN* genes caused reversed differential expression for many drought-responsive genes, which provides a partial explanation for the drought-sensitive phenotype of the *OMTN*-OE transgenic rice.

Based on GO (gene ontology) analysis, the differentially expressed genes in the *OMTN* overexpressors are mainly enriched in the following categories: genes responding to environmental stimuli (including abiotic stimuli, oxidative stress, heavy metal stress), genes related to developmental processes (such as pollen recognition), regulatory- (such as transcriptional regulation and protein phosphorylation) related genes, and metabolism- (including carbohydrate synthesis and catabolism, isoflavones secondary metabolism, and lipid metabolism) related genes.

**Fig. 9.**
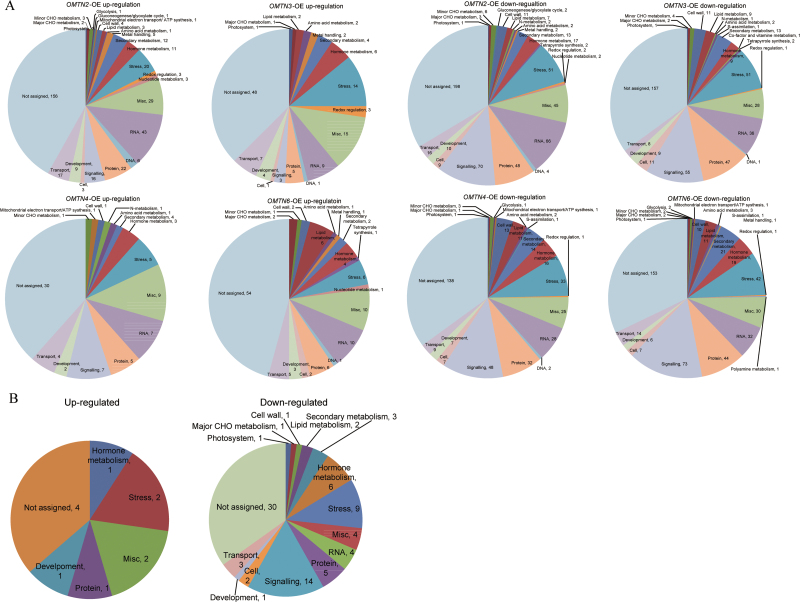
Functional categorization of up- and down-regulated genes. Mapman was used to classify the genes into different biological processes. Gene numbers are displayed next to the terms. (A) Distribution of the up- and down-regulated genes in each of the *OMTN* overexpressors into major biological functions. (B) Categorization of the commonly up- and down-regulated genes in the *OMTN2*, *OMTN3*, *OMTN4*, and *OMTN6* overexpressors. (This figure is available in colour at *JXB* online.)

It is worth noting that 79 and 11 genes were down-regulated and up-regulated, respectively, in all of the transgenic materials overexpressing the four *OMTN* genes (Fig. 8B, C; Supplementary Table S8 available at *JXB* online). MapMan was used to classify the genes with consistent expression change patterns in the *OMTN* overexpressors into different biological function categories. As shown in Fig. 9B, the down-regulated genes were classified into 14 groups, with the exception of the genes whose functions have yet to be assigned. The regulatory category contains some down-regulated genes encoding transcription factors (e.g. NAC and zinc finger factors), signalling transduction components (e.g. calcium-regulated cascade proteins), and protein modification groups (e.g. protein kinases and phosphatases). Functional proteins encoded by some down-regulated genes include metabolism-related enzymes (e.g. α-amylase isozyme 3D precursor, 1-aminocyclopropane-1-carboxylate oxidase 1, ent-kaurene synthase A, and a very-long-chain fatty acid condensing enzyme), ion transporters (e.g. potassium transporter 7, magnesium transporter CorA), chaperones (e.g. heat shock proteins), and redox-related enzymes (e.g. peroxidase, oxidoreductase, multicopper oxidase). In fact, many homologues of these down-regulated genes have roles in the response or adaptation to environmental stimuli (for details, see the Discussion).

In addition, promoter regions containing 1kb upstream of the predicted start codon of each of the down- and up-regulated genes were further analysed. The NAC recognition sites (NACRS) and core DNA-binding sites identified in *Arabidopsis* (Tran *et al.*, 2004) were found to be widely present in the promoters of the majority of these genes (data not shown), implying that some of these genes might be directly recognized and regulated by the OMTNs.

## Discussion

### Comparison of the miR164 targets in rice and *Arabidopsis*

A subset of NAC genes comprised of *CUC1* (*At3g15170*), *CUC2* (*At5g53950*), *NAC1* (*At1g56010*), *At5g07680*, and *At5g61430* were documented to be miR164 targets in *Arabidopsis* (Rhoades *et al.*, 2002), and they perform diverse biological functions. *CUC1* and *CUC2* together participate in the maintenance of organ boundaries, and *NAC1* is involved in the regulation of lateral root development in *Arabidopsis* (Xie *et al.*, 2000; Hibara *et al.*, 2003; Nikovics *et al.*, 2006; Peaucelle *et al.*, 2007; Sieber *et al.*, 2007). To date, nine genes have been considered as putative miR164 targets in rice. Six of them are NAC genes, of which the target sites are located downstream of the conserved NAC domain. The remaining targets include two genes encoding phytanoyl-CoA dioxygenase and one encoding a phytosulphokine precursor (Supplementary Table S3 available at *JXB* online), and the target sites are located in the 3′-UTR and CDS region of the genes, respectively. The *Arabidopsis* miR164 family consists of three members, whereas the miR164 family in rice is composed of six members. Consequently, it is hardly surprising that the number of miR164 targets in rice is more than that in *Arabidopsis*. Interestingly, the *Arabidopsis* miR164 targets are all NAC genes, and they are mainly involved in the regulation of organ architecture and the development of lateral roots. Yet, the present study suggests that the miR164 targets include not only NAC genes, but also two other types of genes in rice. The NAC targets of miR164 in rice appear to be associated with the response to abitotic stresses. This study revealed the differences in the numbers, gene types, as well as the biological functions of the miR164 targets between dicot (*Arabidopsis*) and monocot (rice) plants. To date, investigations on rice miR164 and their targets are rather rare. Clarifying the biological functions of the miR164 targets will help uncover the function of miR164 in crops. Furthermore, it will help us obtain a better understanding of the functional conservation and diversity of miRNAs and their targets between dicot and monocot plants.

### The miR164-targeted sites of the *OMTN* genes were highly conserved

Recently, more and more plant miRNAs and their targets have been explored by bioinformatics tools and improved molecular biology techniques. However, the natural sequence variation in the mature miRNAs and their targets was seldom reported. Sequence analysis of 92 mature miR164 sequences from 24 plant species suggested that most of the mature sequences were identical. Three out of six rice miR164 family members (miR164a, miR164b, and miR164f) have exactly the same mature sequence, and 1–2 nucleotide differences are found in the mature sequences of the other three members. The miR164 recognition sites of the *OMTN* genes were further checked in 158 rice varieties representing a mini-core collection of rice germplasm. Comparative sequencing results revealed that the miR164 key recognition sites in the *OMTN* genes are fairly conserved in the 158 rice varieties which were tested. A few SNPs were found to exist within the target sites, but most of them do not affect the recognition between miR164 and the *OMTN* genes. The evolutionary conservation suggested that the target sites may have important functions. Further research showed that deletion of the target sites abolished or impaired the transactivation activities of the OMTNs in yeast, implying an essential role for the target sites for maintaining the normal functions of the OMTN proteins. Further illuminating the biological function of the target sites will reveal the significance of the high conservation of the target sites during evolution.

### OMTNs negatively regulate drought resistance in rice

Current research on miR164 targets in plants mostly focuses on their roles in regulating developmental processes. To date it has not been reported that the miR164 targets are involved in abiotic stress responses. It was noticed here that most of the *OMTN* genes exhibited abiotic stress-responsive expression patterns in the rice seedling cDNA microarray, which implied roles for the *OMTN* genes in responding to abiotic stresses. The results of drought stress testing at the reproductive stage showed that overexpressing *OMTN2*, *OMTN3*, *OMTN4*, and *OMTN6* increased the sensitivity of transgenic plants to drought stress to varying degrees at the reproductive stage. The overexpression lines and WT were tested for drought tolerance at other developmental stages, but no significant difference was observed, which suggests that the roles of the *OMTN* genes in regulating drought resistance may be associated with reproductive development.

To decipher the possible regulatory mechanisms of the increased drought sensitivity of the *OMTN* overexpressors, the genomic expression profiles of the *OMTN*-overexpressing transgenic rice and the WT were compared. The profiling data revealed that a large set of genes were up-regulated or down-regulated in the *OMTN2*-, *OMTN3*-, *OMTN4*-, and *OMTN6*-OE plants (Fig. 7B). As shown in Fig. 9A, these up- and down-regulated gene sets could be primarily grouped into >10 categories with similar proportions, including genes related to stress, redox regulation, signalling, metabolism, development, and so on. Significantly more genes were down-regulated than were up-regulated in all of the tested transgenic plants (Fig. 8A). Further investigation revealed that most of the genes with transcript abundance significantly decreased in the transgenic plants are responsive to stresses, which correlates with the drought-sensitive phenotype of the transgenic plants which was observed. It is proposed that the increased drought sensitivity of the *OMTN*-OE transgenic plants may be derived from the reduction of many regulatory and functional genes. These regulatory genes include the genes encoding various transcription factors which belong to the zinc finger, NAC, Myb, WRKY, bHLH, and AP2 families; and signalling pathway components including the genes mainly involved in auxin, ethylene, and gibberellin metabolism and signalling; protein kinases, etc. The functional genes category contains the genes whose products are transporters, enzymes mediating ROS (reactive oxygen species) scavenging and detoxification, chaperones, proteins related to cell wall synthesis and secondary metabolism, and so forth. The Venn diagram analysis presented a complex crossover relationship of the differentially expressed genes among the *OMTN* overexpressors (Fig. 8B, C). Notably, there were 79 down-regulated and 11 up-regulated genes overlapping among all of the transgenic plants. There is evidence that many homologues of these down-regulated genes have roles in response or adaptation to environmental stimuli. For instance, calmodulin-binding protein and calcium-binding EGF domain-containing protein were found to be involved in multiple environmental signalling pathways in plants (Yang and Poovaiah, 2002). Protein kinases have been widely reported with important roles in stress signalling, and are potentially beneficial for plant tolerance engineering (Vinocur and Altman, 2005). Heat shock proteins (Hsps) function in helping to maintain proper folding and conformation of proteins, thus preventing protein dysfunction and denaturation caused by adverse environmental conditions (W. Wang *et al.*, 2004). Detoxication enzymes such as peroxidase, oxidoreductase, and multicopper oxidase were documented to alleviate the oxidative damage and confer stress tolerance in plants (Murgia *et al.*, 2004; Vinocur and Altman, 2005). Cytochrome P450s were also suggested to take part in a wide range of biochemical pathways and protect plants from the damage caused by various stresses (Narusaka *et al.*, 2004). Moreover, other genes generate products such as heavy metal-associated proteins (Barth *et al.*, 2004), flavonoids (Winkel-Shirley, 2002), transportors (Shabala and Cuin, 2008), and glycosyl transferases (Cheong *et al.*, 2002) which have been reported to be associated with stress adaption in plants. Interestingly, it was also noticed that some genes whose products were pathogen-related proteins, wall-associated kinases (Sivaguru *et al.*, 2003; Decreux and Messiaen, 2005), acidic endochitinase precursors, and NBS-LRR-type disease resistance proteins were also down-regulated in transgenic plants, suggesting that the OMTNs might also be involved in defence responses. Taken together, the expression profiling results well supported that the *OMTN* genes negatively regulate drought tolerance in rice.

### OMTNs may also function in growth and development in rice

MiR164-tartgeted NAC genes have been reported to participate in the regulation of growth and development in *Arabidopsis* (Mallory *et al.*, 2004; Guo *et al.*, 2005; Li *et al.*, 2012); therefore, it was assumed that the *OMTN* genes may also function in growth and developmental processes in rice. Twisted leaves and fusion organs were observed in the *OMTN4*-RNAi and *OMTN6*-RNAi transgenic plants, which were very similar to the phenotype of the miR164-OE plants (unpublished data). The mesophyll cells exhibited abnormal morphology and structure, and many chloroplasts were fused in the leaves of the RNAi plants. Moreover, the osmiophilic granules were also significantly increased (Supplementary Fig. S1 available at *JXB* online). Although the relationship between the changes of the organelles at the microscopic level and the altered phenotypes is unclear, it was assumed that, based on these observations, the *OMTN* genes together with miR164 participate in the regulation of maintenance of organ boundaries and normal development in rice.

In conclusion, the miR164-targeted NAC genes in rice were characterized for the features of transcription factor, conservation of the miR164 recognition sites, and stress responsiveness. Overexpressing *OMTN2*, *OMTN3*, *OMTN4*, and *OMTN6* in rice significantly decreased drought resistance at the reproductive stage, revealing that the OMTNs have novel functions as negative regulators of drought resistance in rice, in addition to the conserved roles in regulating the developmental processes as reported in *Arabidopsis*.

## Supplementary data

Supplementary data are available at *JXB* online.

Figure S1. Developmental defects of the *OMTN4*- and *OMTN6*-RNAi transgenic rice plants.

Figure S2. Suppression of *OMTN4* and *OMTN6* slightly increased tolerance to PEG treatment.

Table S1. List of the rice varieties used in the target sites conservation analysis.

Table S2. List of primers used in this study.

Table S3. General information of the miR164-targeted genes in rice.

Table S4. Up- and down-regulated genes in the transgenic rice plants overexpressing *OMTN2*.

Table S5. Up- and down-regulated genes in the transgenic rice plants overexpressing *OMTN3*.

Table S6. Up- and down-regulated genes in the transgenic rice plants overexpressing *OMTN4*.

Table S7. Up- and down-regulated genes in the transgenic rice plants overexpressing *OMTN6*.

Table S8. List of genes with consistent expression change patterns in the *OMTN2*, *OMTN3*, *OMTN4*, and *OMTN6* overexpressors.

Supplementary Data

## References

[CIT0001] Bao N Lye KW Barton MK 2004 MicroRNA binding sites in *Arabidopsis* class III HD-ZIP mRNAs are required for methylation of the template chromosome. Developmental Cell 7, 653–662 15525527 10.1016/j.devcel.2004.10.003

[CIT0002] Bari R Datt Pant B Stitt M Scheible WR 2006 PHO2, microRNA399, and PHR1 define a phosphate-signaling pathway in plants. Plant Physiology 141, 988–999 16679424 10.1104/pp.106.079707 PMC1489890

[CIT0003] Bartel DP 2004 MicroRNAs: genomics, biogenesis, mechanism, and function. Cell 116, 281–297 14744438 10.1016/s0092-8674(04)00045-5

[CIT0004] Barth O Zschiesche W Siersleben S Humbeck K 2004 Isolation of a novel barley cDNA encoding a nuclear protein involved in stress response and leaf senescence. Physiologia Plantarum 121, 282–293 15153196 10.1111/j.0031-9317.2004.00325.x

[CIT0005] Bazzini AA Almasia NI Manacorda CA 2009 Virus infection elevates transcriptional activity of miR164a promoter in plants. BMC Plant Biology 9, 152 20042107 10.1186/1471-2229-9-152 PMC2809068

[CIT0006] Brodersen P Sakvarelidze-Achard L Bruun-Rasmussen M Dunoyer P Yamamoto YY Sieburth L Voinnet O 2008 Widespread translational inhibition by plant miRNAs and siRNAs. Science 320, 1185–1190 18483398 10.1126/science.1159151

[CIT0007] Cheong YH Chang H-S Gupta R Wang X Zhu T Luan S 2002 Transcriptional profiling reveals novel interactions between wounding, pathogen, abiotic stress, and hormonal responses in Arabidopsis. Plant Physiology 129, 661–677 12068110 10.1104/pp.002857 PMC161692

[CIT0008] Chiou TJ Aung K Lin SI Wu CC Chiang SF Su CL 2006 Regulation of phosphate homeostasis by MicroRNA in *Arabidopsis* . The Plant Cell 18, 412–421 16387831 10.1105/tpc.105.038943 PMC1356548

[CIT0009] Decreux A Messiaen J 2005 Wall-associated kinase WAK1 interacts with cell wall pectins in a calcium-induced conformation. Plant and Cell Physiology 46, 268–278 15769808 10.1093/pcp/pci026

[CIT0010] Fang Y You J Xie K Xie W Xiong L 2008 Systematic sequence analysis and identification of tissue-specific or stress-responsive genes of NAC transcription factor family in rice. Molecular Genetics and Genomics 280, 547–563 18813954 10.1007/s00438-008-0386-6

[CIT0011] Fujii H Chiou TJ Lin SI Aung K Zhu JK 2005 A miRNA involved in phosphate-starvation response in *Arabidopsis* . Current Biology 15, 2038–2043 16303564 10.1016/j.cub.2005.10.016

[CIT0012] Fujita M Fujita Y Maruyama K Seki M Hiratsu K Ohme-Takagi M Tran LS Yamaguchi-Shinozaki K Shinozaki K 2004 A dehydration-induced NAC protein, RD26, is involved in a novel ABA-dependent stress-signaling pathway. The Plant Journal 39, 863–876 15341629 10.1111/j.1365-313X.2004.02171.x

[CIT0013] Griffiths-Jones S 2006 miRBase: the microRNA sequence database. Methods in Molecular Biology 342, 129–138 16957372 10.1385/1-59745-123-1:129

[CIT0014] Guo HS Xie Q Fei JF Chua NH 2005 MicroRNA directs mRNA cleavage of the transcription factor *NAC1* to downregulate auxin signals for arabidopsis lateral root development. The Plant Cell 17, 1376–1386 15829603 10.1105/tpc.105.030841 PMC1091761

[CIT0015] He XF Fang YY Feng L Guo HS 2008 Characterization of conserved and novel microRNAs and their targets, including a TuMV-induced TIR-NBS-LRR class *R* gene-derived novel miRNA in Brassica. FEBS Letters 582, 2445–2452 18558089 10.1016/j.febslet.2008.06.011

[CIT0016] Hibara K Takada S Tasaka M 2003 *CUC1* gene activates the expression of SAM-related genes to induce adventitious shoot formation. The Plant Journal 36, 687–696 14617069 10.1046/j.1365-313x.2003.01911.x

[CIT0017] Hu H Dai M Yao J Xiao B Li X Zhang Q Xiong L 2006 Overexpressing a NAM, ATAF, and CUC (NAC) transcription factor enhances drought resistance and salt tolerance in rice. Proceedings of the National Academy of Sciences, USA 103, 12987–12992 10.1073/pnas.0604882103 PMC1559740 16924117

[CIT0018] Hu H You J Fang Y Zhu X Qi Z Xiong L 2008 Characterization of transcription factor gene *SNAC2* conferring cold and salt tolerance in rice. Plant Molecular Biology 67, 169–181 18273684 10.1007/s11103-008-9309-5

[CIT0019] Jeong DH German MA Rymarquis LA Thatcher SR Green PJ 2010 Abiotic stress-associated miRNAs: detection and functional analysis. Methods in Molecular Biology 592, 203–230 19802598 10.1007/978-1-60327-005-2_14

[CIT0020] Jeong JS Kim YS Baek KH Jung H Ha SH Do Choi Y Kim M Reuzeau C Kim JK 2010 Root-specific expression of *OsNAC10* improves drought tolerance and grain yield in rice under field drought conditions. Plant Physiology 153, 185–197 20335401 10.1104/pp.110.154773 PMC2862432

[CIT0021] Jeong JS Park YT Jung H Park SH Kim JK 2009 Rice NAC proteins act as homodimers and heterodimers. Plant Biotechnology Reports 3, 127–134

[CIT0022] Jia X Mendu V Tang G 2010 An array platform for identification of stress-responsive microRNAs in plants. Methods in Molecular Biology 639, 253–269 20387051 10.1007/978-1-60761-702-0_15

[CIT0023] Jia X Ren L Chen QJ Li R Tang G 2009 UV-B-responsive microRNAs in *Populus tremula* . Journal of Plant Physiology 166, 2046–2057 19628301 10.1016/j.jplph.2009.06.011

[CIT0024] Jones-Rhoades MW Bartel DP 2004 Computational identification of plant microRNAs and their targets, including a stress-induced miRNA. Molecular Cell 14, 787–799 15200956 10.1016/j.molcel.2004.05.027

[CIT0025] Khraiwesh B Arif MA Seumel GI Ossowski S Weigel D Reski R Frank W 2010 Transcriptional control of gene expression by microRNAs. Cell 140, 111–122 20085706 10.1016/j.cell.2009.12.023

[CIT0026] Khraiwesh B Zhu JK Zhu J 2012 Role of miRNAs and siRNAs in biotic and abiotic stress responses of plants. Biochimica et Biophysica Acta 1819, 137–148 21605713 10.1016/j.bbagrm.2011.05.001 PMC3175014

[CIT0027] Kim JH Woo HR Kim J Lim PO Lee IC Choi SH Hwang D Nam HG 2009 Trifurcate feed-forward regulation of age-dependent cell death involving *miR164* in *Arabidopsis* . Science 323, 1053–1057 19229035 10.1126/science.1166386

[CIT0028] Kleinow T Himbert S Krenz B Jeske H Koncz C 2009 NAC domain transcription factor ATAF1 interacts with SNF1-related kinases and silencing of its subfamily causes severe developmental defects in *Arabidopsis* . Plant Science 177, 360–370

[CIT0029] Laufs P Peaucelle A Morin H Traas J 2004 MicroRNA regulation of the CUC genes is required for boundary size control in *Arabidopsis* meristems. Development 131, 4311–4322 15294871 10.1242/dev.01320

[CIT0030] Li B Yin W Xia X 2009 Identification of microRNAs and their targets from *Populus euphratica* . Biochemical and Biophysical Research Communications 388, 272–277 19664594 10.1016/j.bbrc.2009.07.161

[CIT0031] Li J Guo G Guo W Tong D Ni Z Sun Q Yao Y 2012 miRNA164-directed cleavage of *ZmNAC1* confers lateral root development in maize (*Zea mays* L.). BMC Plant Biology 12, 220 23171309 10.1186/1471-2229-12-220 PMC3554535

[CIT0032] Li T Li H Zhang YX Liu JY 2011 Identification and analysis of seven H_2_O_2_-responsive miRNAs and 32 new miRNAs in the seedlings of rice (*Oryza sativa* L. ssp. *indica*). Nucleic Acids Research 39, 2821–2833 21113019 10.1093/nar/gkq1047 PMC3074118

[CIT0033] Li WX Oono Y Zhu J He XJ Wu JM Iida K Lu XY Cui X Jin H Zhu JK 2008 The *Arabidopsis* NFYA5 transcription factor is regulated transcriptionally and posttranscriptionally to promote drought resistance. The Plant Cell 20, 2238–2251 18682547 10.1105/tpc.108.059444 PMC2553615

[CIT0034] Li YF Zheng Y Addo-Quaye C Zhang L Saini A Jagadeeswaran G Axtell MJ Zhang W Sunkar R 2010 Transcriptome-wide identification of microRNA targets in rice. The Plant Journal 62, 742–759 20202174 10.1111/j.1365-313X.2010.04187.x

[CIT0035] Lin YJ Zhang Q 2005 Optimising the tissue culture conditions for high efficiency transformation of indica rice. Plant Cell Reports 23, 540–547 15309499 10.1007/s00299-004-0843-6

[CIT0036] Livak KJ Schmittgen TD 2001 Analysis of relative gene expression data using real-time quantitative PCR and the 2(-Delta Delta C(T)) Method. Methods 25, 402–408 11846609 10.1006/meth.2001.1262

[CIT0037] Lu S Sun YH Chiang VL 2008 Stress-responsive microRNAs in *Populus* . The Plant Journal 55, 131–151 18363789 10.1111/j.1365-313X.2008.03497.x

[CIT0038] Lu S Sun YH Shi R Clark C Li L Chiang VL 2005 Novel and mechanical stress-responsive microRNAs in *Populus trichocarpa* that are absent from *Arabidopsis* . The Plant Cell 17, 2186–2203 15994906 10.1105/tpc.105.033456 PMC1182482

[CIT0039] Lu XY Huang XL 2008 Plant miRNAs and abiotic stress responses. Biochemical and Biophysical Research Communications 368, 458–462 18267107 10.1016/j.bbrc.2008.02.007

[CIT0040] Lv DK Bai X Li Y Ding XD Ge Y Cai H Ji W Wu N Zhu YM 2010 Profiling of cold-stress-responsive miRNAs in rice by microarrays. Gene 459, 39–47 20350593 10.1016/j.gene.2010.03.011

[CIT0041] Mallory AC Dugas DV Bartel DP Bartel B 2004 MicroRNA regulation of NAC-domain targets is required for proper formation and separation of adjacent embryonic, vegetative, and floral organs. Current Biology 14, 1035–1046 15202996 10.1016/j.cub.2004.06.022

[CIT0042] Murgia I Tarantino D Vannini C Bracale M Carravieri S Soave C 2004 *Arabidopsis thaliana* plants overexpressing thylakoidal ascorbate peroxidase show increased resistance to *Paraquat*-induced photooxidative stress and to nitric oxide-induced cell death. The Plant Journal 38, 940–953 15165186 10.1111/j.1365-313X.2004.02092.x

[CIT0043] Murray MG Thompson WF 1980 Rapid isolation of high molecular weight plant DNA. Nucleic Acids Research 8, 4321–4325 7433111 10.1093/nar/8.19.4321 PMC324241

[CIT0044] Nakashima K Takasaki H Mizoi J Shinozaki K Yamaguchi-Shinozaki K 2012 NAC transcription factors in plant abiotic stress responses. Biochimica et Biophysica Acta 1819, 97–103 22037288 10.1016/j.bbagrm.2011.10.005

[CIT0045] Nakashima K Tran LS Van Nguyen D Fujita M Maruyama K Todaka D Ito Y Hayashi N Shinozaki K Yamaguchi-Shinozaki K 2007 Functional analysis of a NAC-type transcription factor OsNAC6 involved in abiotic and biotic stress-responsive gene expression in rice. The Plant Journal 51, 617–630 17587305 10.1111/j.1365-313X.2007.03168.x

[CIT0046] Narusaka Y Narusaka M Seki M Umezawa T Ishida J Nakajima M Enju A Shinozaki K 2004 Crosstalk in the responses to abiotic and biotic stresses in *Arabidopsis*: analysis of gene expression in *cytochrome P450* gene superfamily by cDNA microarray. Plant Molecular Biology 55, 327–342 15604685 10.1007/s11103-004-0685-1

[CIT0047] Navarro L Dunoyer P Jay F Arnold B Dharmasiri N Estelle M Voinnet O Jones JD 2006 A plant miRNA contributes to antibacterial resistance by repressing auxin signaling. Science 312, 436–439 16627744 10.1126/science.1126088

[CIT0048] Nikovics K Blein T Peaucelle A Ishida T Morin H Aida M Laufs P 2006 The balance between the *MIR164A* and *CUC2* genes controls leaf margin serration in *Arabidopsis* . The Plant Cell 18, 2929–2945 17098808 10.1105/tpc.106.045617 PMC1693934

[CIT0049] Nuruzzaman M Manimekalai R Sharoni AM Satoh K Kondoh H Ooka H Kikuchi S 2010 Genome-wide analysis of NAC transcription factor family in rice. Gene 465, 30–44 20600702 10.1016/j.gene.2010.06.008

[CIT0050] Nuruzzaman M Sharoni AM Kikuchi S 2013 Roles of NAC transcription factors in the regulation of biotic and abiotic stress responses in plants. Frontiers in Microbiology 4, 248 24058359 10.3389/fmicb.2013.00248 PMC3759801

[CIT0051] Olsen AN Ernst HA Leggio LL Skriver K 2005 NAC transcription factors: structurally distinct, functionally diverse. Trends in Plant Science 10, 79–87 15708345 10.1016/j.tplants.2004.12.010

[CIT0052] Palatnik JF Allen E Wu X Schommer C Schwab R Carrington JC Weigel D 2003 Control of leaf morphogenesis by microRNAs. Nature 425, 257–263 12931144 10.1038/nature01958

[CIT0053] Peaucelle A Morin H Traas J Laufs P 2007 Plants expressing a *miR164*-resistant *CUC2* gene reveal the importance of post-meristematic maintenance of phyllotaxy in *Arabidopsis* . Development 134, 1045–1050 17251269 10.1242/dev.02774

[CIT0054] Puranik S Sahu PP Srivastava PS Prasad M 2012 NAC proteins: regulation and role in stress tolerance. Trends in Plant Science 17, 369–381 22445067 10.1016/j.tplants.2012.02.004

[CIT0055] Reyes JL Chua NH 2007 ABA induction of miR159 controls transcript levels of two MYB factors during *Arabidopsis* seed germination. The Plant Journal 49, 592–606 17217461 10.1111/j.1365-313X.2006.02980.x

[CIT0056] Rhoades MW Reinhart BJ Lim LP Burge CB Bartel B Bartel DP 2002 Prediction of plant microRNA targets. Cell 110, 513–520 12202040 10.1016/s0092-8674(02)00863-2

[CIT0057] Sanan-Mishra N Kumar V Sopory SK Mukherjee SK 2009 Cloning and validation of novel miRNA from basmati rice indicates cross talk between abiotic and biotic stresses. Molecular Genetics and Genomics 282, 463–474 20131478 10.1007/s00438-009-0478-y

[CIT0058] Shabala S Cuin TA 2008 Potassium transport and plant salt tolerance. Physiologia Plantarum 133, 651–669 18724408 10.1111/j.1399-3054.2007.01008.x

[CIT0059] Sieber P Wellmer F Gheyselinck J Riechmann JL Meyerowitz EM 2007 Redundancy and specialization among plant microRNAs: role of the *MIR164* family in developmental robustness. Development 134, 1051–1060 17287247 10.1242/dev.02817

[CIT0060] Sivaguru M Ezaki B He ZH Tong H Osawa H Baluska F Volkmann D Matsumoto H 2003 Aluminum-induced gene expression and protein localization of a cell wall-associated receptor kinase in Arabidopsis. Plant Physiology 132, 2256–2266 12913180 10.1104/pp.103.022129 PMC181309

[CIT0061] Sperotto RA Ricachenevsky FK Duarte GL Boff T Lopes KL Sperb ER Grusak MA Fett JP 2009 Identification of up-regulated genes in flag leaves during rice grain filling and characterization of OsNAC5, a new ABA-dependent transcription factor. Planta 230, 985–1002 19697058 10.1007/s00425-009-1000-9

[CIT0062] Sunkar R Zhou X Zheng Y Zhang W Zhu JK 2008 Identification of novel and candidate miRNAs in rice by high throughput sequencing. BMC Plant Biology 8, 25 18312648 10.1186/1471-2229-8-25 PMC2292181

[CIT0063] Thompson JD Gibson TJ Plewniak F Jeanmougin F Higgins DG 1997 The CLUSTAL_X windows interface: flexible strategies for multiple sequence alignment aided by quality analysis tools. Nucleic Acids Research 25, 4876–4882 9396791 10.1093/nar/25.24.4876 PMC147148

[CIT0064] Tran LS Nakashima K Sakuma Y Osakabe Y Qin F Simpson SD Maruyama K Fujita Y Shinozaki K Yamaguchi-Shinozaki K 2007 Co-expression of the stress-inducible zinc finger homeodomain ZFHD1 and NAC transcription factors enhances expression of the *ERD1* gene in *Arabidopsis* . The Plant Journal 49, 46–63 17233795 10.1111/j.1365-313X.2006.02932.x

[CIT0065] Tran LS Nakashima K Sakuma Y Simpson SD Fujita Y Maruyama K Fujita M Seki M Shinozaki K Yamaguchi-Shinozaki K 2004 Isolation and functional analysis of Arabidopsis stress-inducible NAC transcription factors that bind to a drought-responsive *cis*-element in the *early responsive to dehydration stress 1* promoter. The Plant Cell 16, 2481–2498 15319476 10.1105/tpc.104.022699 PMC520947

[CIT0066] Vasudevan S Tong Y Steitz JA 2007 Switching from repression to activation: microRNAs can up-regulate translation. Science 318, 1931–1934 18048652 10.1126/science.1149460

[CIT0067] Vinocur B Altman A 2005 Recent advances in engineering plant tolerance to abiotic stress: achievements and limitations. Current Opinion in Biotechnology 16, 123–132 15831376 10.1016/j.copbio.2005.02.001

[CIT0068] Wang W Vinocur B Shoseyov O Altman A 2004 Role of plant heat-shock proteins and molecular chaperones in the abiotic stress response. Trends in Plant Science 9, 244–252 15130550 10.1016/j.tplants.2004.03.006

[CIT0069] Wang XJ Reyes JL Chua NH Gaasterland T 2004 Prediction and identification of *Arabidopsis thaliana* microRNAs and their mRNA targets. Genome Biology 5, R65 15345049 10.1186/gb-2004-5-9-r65 PMC522872

[CIT0070] Winkel-Shirley B 2002 Biosynthesis of flavonoids and effects of stress. Current Opinion in Plant Biology 5, 218–223 11960739 10.1016/s1369-5266(02)00256-x

[CIT0071] Wu L Zhang Q Zhou H Ni F Wu X Qi Y 2009 Rice MicroRNA effector complexes and targets. The Plant Cell 21, 3421–3435 19903869 10.1105/tpc.109.070938 PMC2798332

[CIT0072] Wu L Zhou H Zhang Q Zhang J Ni F Liu C Qi Y 2010 DNA methylation mediated by a microRNA pathway. Molecular Cell 38, 465–475 20381393 10.1016/j.molcel.2010.03.008

[CIT0073] Xie Q Frugis G Colgan D Chua NH 2000 *Arabidopsis* NAC1 transduces auxin signal downstream of TIR1 to promote lateral root development. Genes and Development 14, 3024–3036 11114891 10.1101/gad.852200 PMC317103

[CIT0074] Xie Q Guo HS Dallman G Fang S Weissman AM Chua NH 2002 SINAT5 promotes ubiquitin-related degradation of NAC1 to attenuate auxin signals. Nature 419, 167–170 12226665 10.1038/nature00998

[CIT0075] Xin M Wang Y Yao Y Xie C Peng H Ni Z Sun Q 2010 Diverse set of microRNAs are responsive to powdery mildew infection and heat stress in wheat (*Triticum aestivum* L.). BMC Plant Biology 10, 123 20573268 10.1186/1471-2229-10-123 PMC3095282

[CIT0076] Xue W Xing Y Weng X Zhao Y Tang W Wang L Zhou H Yu S Xu C Li X Zhang Q 2008 Natural variation in *Ghd7* is an important regulator of heading date and yield potential in rice. Nature Genetics 40, 761–767 18454147 10.1038/ng.143

[CIT0077] Yang T Poovaiah BW 2002 A calmodulin-binding/CGCG box DNA-binding protein family involved in multiple signaling pathways in plants. Journal of Biological Chemistry 277, 45049–45058 12218065 10.1074/jbc.M207941200

[CIT0078] Yoo SD Cho YH Sheen J 2007 *Arabidopsis* mesophyll protoplasts: a versatile cell system for transient gene expression analysis. Nature Protocols 2, 1565–1572 10.1038/nprot.2007.199 17585298

[CIT0079] Yoo SY Kim Y Kim SY Lee JS Ahn JH 2007 Control of flowering time and cold response by a NAC-domain protein in *Arabidopsis* . PLoS One 2, e642 17653269 10.1371/journal.pone.0000642 PMC1920552

[CIT0080] Yoon HK Kim SG Kim SY Park CM 2008 Regulation of leaf senescence by NTL9-mediated osmotic stress signaling in *Arabidopsis* . Molecules and Cells 25, 438–445 18443413

[CIT0081] Zhao B Ge L Liang R Li W Ruan K Lin H Jin Y 2009 Members of miR-169 family are induced by high salinity and transiently inhibit the NF-YA transcription factor. BMC Molecular Biology 10, 29 19351418 10.1186/1471-2199-10-29 PMC2670843

[CIT0082] Zhao JP Jiang XL Zhang BY Su XH 2012 Involvement of microRNA-mediated gene expression regulation in the pathological development of stem canker disease in *Populus trichocarpa* . PLoS One 7, e44968 23028709 10.1371/journal.pone.0044968 PMC3445618

[CIT0083] Zheng X Chen B Lu G Han B 2009 Overexpression of a NAC transcription factor enhances rice drought and salt tolerance. Biochemical and Biophysical Research Communications 379, 985–989 19135985 10.1016/j.bbrc.2008.12.163

[CIT0084] Zhou J Wang X Jiao Y 2007 Global genome expression analysis of rice in response to drought and high-salinity stresses in shoot, flag leaf, and panicle. Plant Molecular Biology 63, 591–608 17225073 10.1007/s11103-006-9111-1 PMC1805039

[CIT0085] Zhou M Gu L Li P Song X Wei L Chen Z Cao X 2010 Degradome sequencing reveals endogenous small RNA targets in rice (*Oryza sativa* L. ssp. *indica*). Frontiers in Biology 5, 67–90

