# Supplementary material for: Conserved miR164-targeted NAC genes negatively regulate drought resistance in rice
Source: J Exp Bot. 2014 Mar 6;65(8):2119–35. doi: 10.1093/jxb/eru072 (PMC3991743; doi:10.1093/jxb/eru072)
Supplement: Supplementary Data [file supp_65_8_2119__index.html]

Conserved miR164-targeted NAC genes negatively regulate drought resistance in rice — Conserved miR164-targeted NAC genes negatively regulate drought resistance in rice — Supplementary Data 

# Conserved miR164-targeted NAC genes negatively regulate drought resistance in rice

## Supplementary Data

Data files

**Files in this Data Supplement:**

- Supplementary Data - Supplementary Data
