# Supplementary material for: Conserved miR164-targeted NAC genes negatively regulate drought resistance in rice
Source: J Exp Bot. 2014 Mar 6;65(8):2119–35. doi: 10.1093/jxb/eru072 (PMC3991743; doi:10.1093/jxb/eru072)
Supplement: Supplementary Data [file supp_eru072_jexbot116699_file001.pdf]

## Supplementary Figures

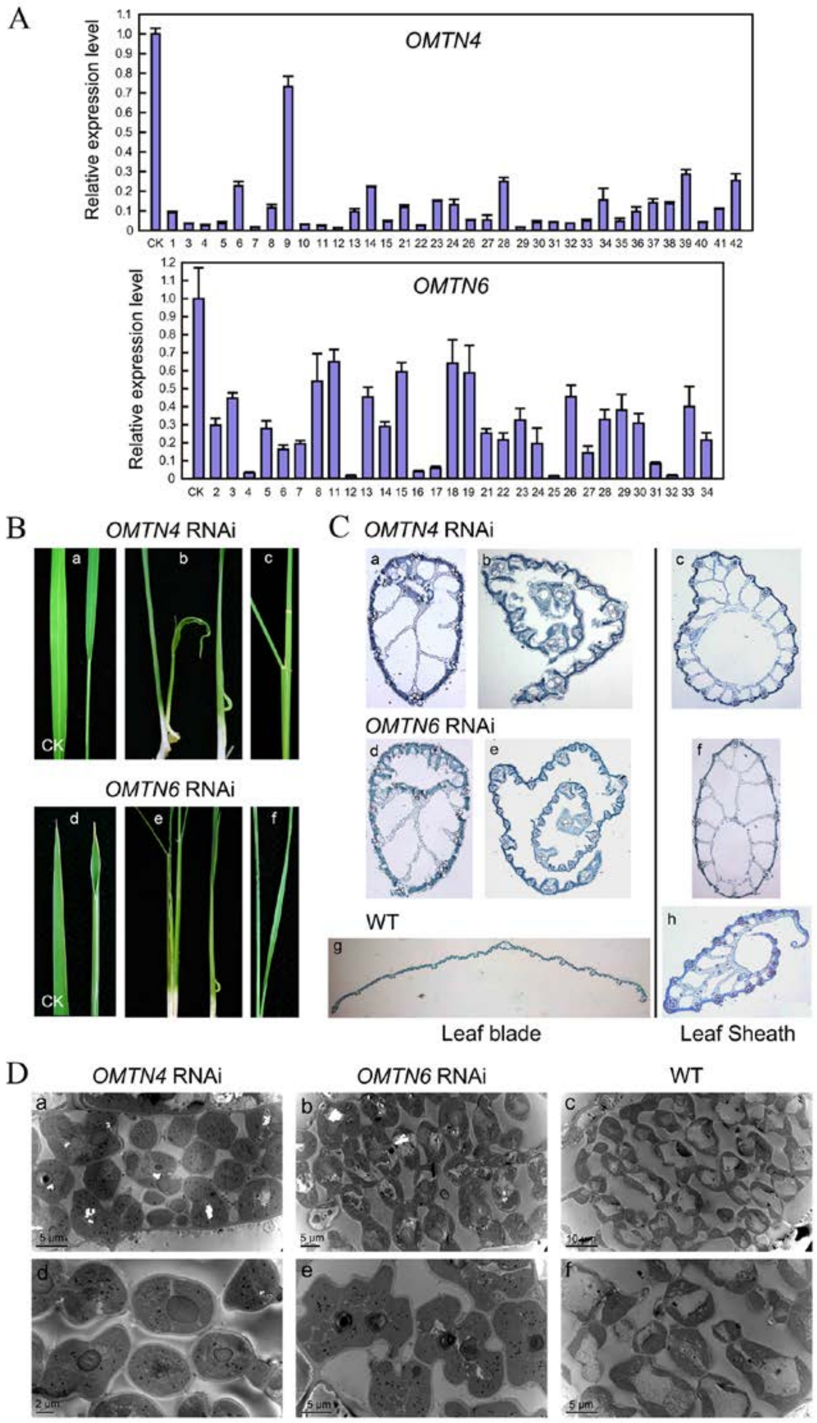


Supplementary Figure S1. Developmental defects of the *OMTN4* and *OMTN6* RNAi transgenic rice plants. (A) Transcript levels of *OMTN4* and *OMTN6* in RNAi transgenic plants. Error bars indicated SE based on three technical replicates. (B) Aberrant phenotype of the *OMTN4* and *OMTN6* RNAi plants. Ba, Bb, Bd and Be

showed blade fusion and curled leaves. Bc and Bf showed leaf sheath fusion. C, .Leaf blade and sheath sections of the *OMTN4* and *OMTN6* RNAi plants (Ca-Cf) and WT (Cg-Ch) plants. D, TEM of the leaves from the *OMTN4* RNAi plants (Da and Dd), the *OMTN6* RNAi plants (Db and De) and WT (Dc and Df) plants. Dd-Df are enlarged view of Da-Dc, respectively.

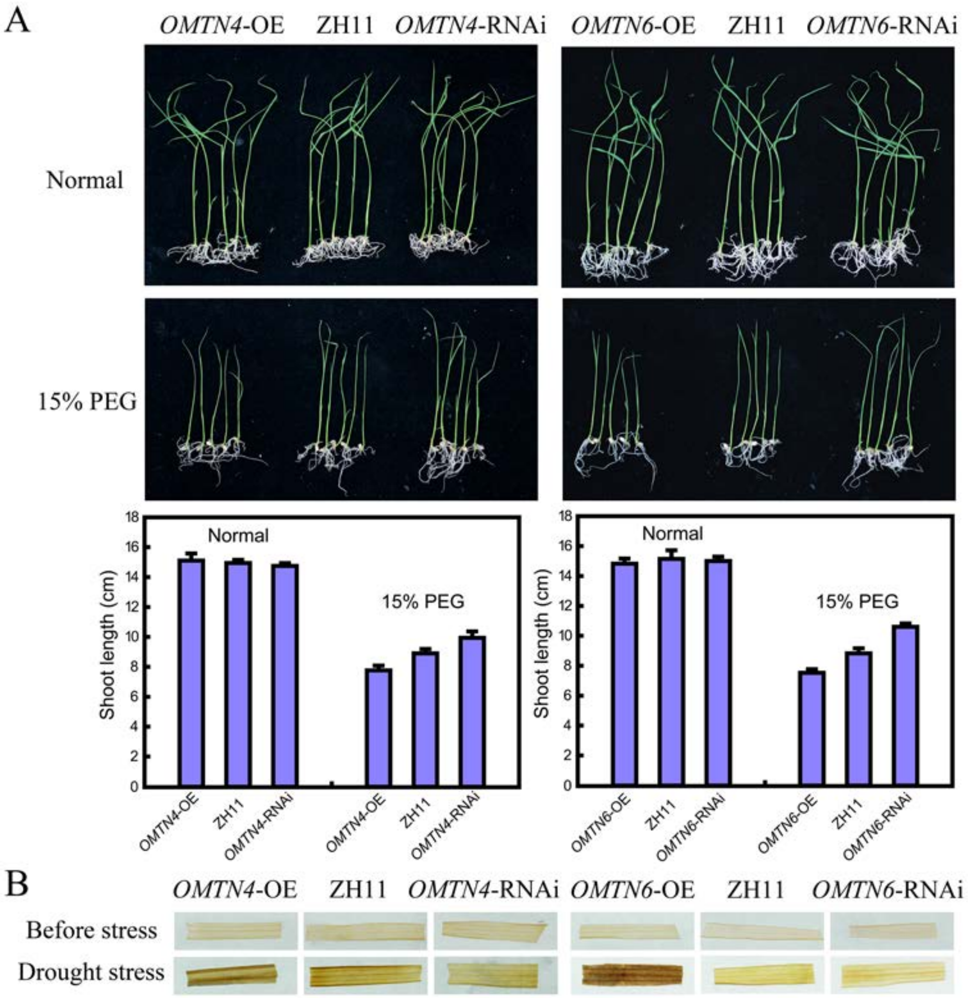


Supplementary Figure S2. Suppression of *OMTN4* and *OMTN6* slightly increased drought tolerance. (A) Suppression of *OMTN4* and *OMTN6* slightly increased tolerance to PEG. Top panel, performance of WT, *OMTN4* and *OMTN6* RNAi transgenic rice plants under normal conditions and PEG treatment. Bottom panel, Shoot length of WT, *OMTN4* and *OMTN6* RNAi plants under normal conditions and PEG treatment. (B) DAB staining analysis of WT, *OMTN4* and *OMTN6* RNAi plants under normal conditions and drought stress.

**Supplementary Table S1. List of rice varieties used in the target sites conservation analysis.**

| Series number | Name of rice variety | Series number | Name of rice variety |
|---|---|---|---|
| 1 | Sadu-cho | 42 | Guizhao2 |
| 2 | Dom Sufid | 43 | Huke3 |
| 3 | Gerdeh | 44 | Teqingxuanhui |
| 4 | Li-Jiang-Xin-Tuan-Hei-Gu | 45 | Huangsiguizhan |
| 5 | AZUCENA | 46 | Xiangwanxian3 |
| 6 | Shan-Huang Zhan-2 | 47 | Taizhong65 |
| 7 | Swarna | 48 | Zaoshunonghu6 |
| 8 | MOROBEREKAN | 49 | Jinyou1 |
| 9 | CYPRESS | 50 | Chengnongshuijin |
| 10 | IR64 | 51 | PeiC122 |
| 11 | Tainong67 | 52 | Guihuahuang |
| 12 | N 22 | 53 | Momi |
| 13 | M202 | 54 | Xiushui115 |
| 14 | Dular | 55 | Sanbaili |
| 15 | Laoguangtou83 | 56 | Jindao1 |
| 16 | Baimaodao | 57 | Dandongludao |
| 17 | AnnongwanjingB | 58 | Liusha1 |
| 18 | Aijiaonante | 59 | Bawangbian |
| 19 | Guangluai4 | 60 | Dongtingwanxian |
| 20 | Xiangaizao10 | 61 | Yangdao2 |
| 21 | Jing7623 | 62 | Zhengdao5 |
| 22 | JinnanteB | 63 | Jing87-304 |
| 23 | Funingzipi | 64 | Sujing2 |
| 24 | Zhenshan97B | 65 | Wanhong1 |
| 25 | Qingsiai16B | 66 | Liushizao |
| 26 | Weiguo | 67 | Muxiqiu |
| 27 | Dianrui409B | 68 | Taizhongxianxuan2 |
| 28 | Liangjing287 | 69 | Dangyu5 |
| 29 | Huhui628 | 70 | Youmangzaojing |
| 30 | 88B | 71 | Laohuzhong |
| 31 | Xianghui91269 | 72 | Nantehao |
| 32 | Longhuamaohu | 73 | Esiniu |
| 33 | Lucaihao | 74 | Cunsanli |
| 34 | Zhonglouyihao1 | 75 | Huangkezaonan |
| 35 | Yelicanghua | 76 | Nantiangangjiu |
| 36 | Shufeng101 | 77 | Benbanggu |
| 37 | Chengduai3 | 78 | Qitoubaigu |
| 38 | Sankecun | 79 | Muguanuo |
| 39 | Gongju73 | 80 | Hanmadao |
| 40 | Jiabala | 81 | Heidu4 |
| 41 | Taishannuo | 82 | Gaoyangdiandao |

| Series number | Name of rice variety | Series number | Name of rice variety |
|---|---|---|---|
| 83 | Chikenuo | 121 | XiangaiB |
| 84 | Haobuka | 122 | Aituogu151 |
| 85 | Shanjiugu | 123 | Taidongludao |
| 86 | Fanhaopi | 124 | Menjiading2 |
| 87 | Lixinjing | 125 | Jiefangdao |
| 88 | Baigedao | 126 | Hongqi5 |
| 89 | Tieganwu | 127 | Baikehualuo |
| 90 | Sanlicun | 128 | Liuyezhan |
| 91 | Meihuanuo | 129 | Aihechi |
| 92 | Lamujia | 130 | Xiangnuo |
| 93 | Magunuo | 131 | Xuanenchangtan |
| 94 | Menjiagao1 | 132 | Jinzhinuo |
| 95 | Xiaohugu | 133 | Laohongdao |
| 96 | Jinxibai | 134 | Wuzidui |
| 97 | Jinbaoyin | 135 | Xibaizhan |
| 98 | Hongainuo | 136 | Zegu |
| 99 | Aizaizhan | 137 | Cungunuo |
| 100 | Dongtingwanxian | 138 | Lengshuigu2 |
| 101 | Babaili | 139 | Banjiemang |
| 102 | Qiyuexian | 140 | Wuzuihonggu |
| 103 | Yuyannuo | 141 | Nangaogu |
| 104 | Mamagu | 142 | Mowanggunei |
| 105 | Qingke | 143 | Qitougu |
| 106 | Hongkezhenuo | 144 | Huangpinuo |
| 107 | Mowanggunei | 145 | ZS97 |
| 108 | Honggu | 146 | Nipponbare |
| 109 | Zimi | 147 | MH63 |
| 110 | Xianggu | 148 | 9311 |
| 111 | Ximaxian | 149 | Zhonghua11 |
| 112 | Jinnante43B | 150 | Balila |
| 113 | Xiangzaoxian7 | 151 | Nangjing11 |
| 114 | 80B | 152 | 02428 |
| 115 | Baoxie123B | 153 | IRAT109 |
| 116 | LimingB | 154 | Xingguo |
| 117 | Jiangnongzao1 | 155 | Dongtingwanxian |
| 118 | Gu154 | 156 | Sanbaili |
| 119 | Ninghui21 | 157 | Baoxuan21 |
| 120 | Shuiyuan300li | 158 | L301B |

**Supplementary Table S2. List of primers used in this study.**

| Purpose | Gene | Sense primer (5'→3') | Anti-sense primer (5'→3') |
|---|---|---|---|
| Amplification of the miR164 target sites of the *OMTN* genes | *OMTN1* | GTGCTGTGCAGGGTGT | GATCCATGCGTTGCTG |
| | *OMTN2* | GCGTAGAACGAGTGGG | TCACGTCGGAGGTGAG |
| | *OMTN3* | GCAGGAAGACTGGGTC | TTCAGATGGAGGAGGC |
| | *OMTN4* | CTCATGCCAGCTCTAA | CTTCACATTGCCTTCA |
| | *OMTN6* | CTATGCAGGGTGTTCT | TCAGTAGTAATCGCTATCT |
| QPCR | *OMTN1* | AGGACACCGGCCTCACGT | GATCCATGCGTTGCTGCC |
| | *OMTN2* | TCGCGATCGAGGCATCTAC | CCCAAATCACCAAATAATCTACACCTA |
| | *OMTN3* | TGCCTGAATTTGGCAGCTT | ACACCTCACCCCCAATGGA |
| | *OMTN4* | ACAATATGGGTAGGGCGATCAA | TCCCTCTTCACATTGCCTTCA |
| | *OMTN6* | GGTCGGGAGCTACGAGCAA | TGATGGAGCCCTTCATTGG |
| | *Ubiquitin* | AACCAGCTGAGGCCCAAGA | ACGATTGATTTAACCAGTCCATGA |
| Amplification of the full-length cDNA of the *OMTN* genes | *OMTN1* | TTggatccAAAAGGTTTAGTAGATGCGATT | AAgtcgacGTAACATTCACTTGGGTTCAA |
| | *OMTN2* | TTggtaccAAGGAGCAGTTAGCCAGGTA | TTggatccACATCTCCCAAATCACCAA |
| | *OMTN3* | TTggtaccCTACCCTTCAATTTCCTTAA | TTggatccGACAACGCAAACCTCTACT |
| | *OMTN4* | TTggtaccCTTCATCAACACACACTGG | TTggtaccCAATAGCACATAAGCACAC |
| | *OMTN6* | TTggtaccCCATTAGCTTGCTATTCTC | TTggatccAGCCATTTTCACTACTCTAC |
| Subcellular location constructs | *OMTN1* | GCtctagaCGTCGATCTAGTCTAAAAGG | GCtctagaATAGGCCCAGAGGGAC |
| | *OMTN2* | GCtctagaTAAGCAAGGAGCAGTTAGCC | GCtctagaCTCGTGGTCGAATTTTTG |
| | *OMTN3* | GCtctagaCTACCCTTCAATTTCCTTAA | GCtctagaGAAGTGATTCATCCAAGAA |
| | *OMTN4* | GCtctagaGATCCAGCATCTCAAG | GCtctagaACTGAGTGAGTTCCACAT |
| | *OMTN6* | GCtctagaCCATTAGCTTGCTATTCT | GCtctagaTTGGGGGTTCCACATC |

| Purpose | Gene | Sense primer (5'→3') | Anti-sense primer (5'→3') |
|---|---|---|---|
| Yeast two-hybrid constructs | *OMTN1* | *att*B1-TGCGATTAGCCAGGCA | *att*B2-TCGGTGGCCAACAGTA |
| | | | *att*B2-GGAACTGGCCCTCCAG |
| | *OMTN2* | *att*B1-TGGAGCAGCATCAGGGCC | *att*B2-CGCCCGCGATTAACGTAC |
| | | | *att*B2-AGAACTGGCCCTCCAGCG |
| | *OMTN3* | *att*B1-TGTCGATGATGAGCTTCT | *att*B2-CAACGCAAACCTCTACTT |
| | | | *att*B2-CTGCCTTGAGCATCTTCT |
| | *OMTN4* | *att*B1-TGAGCGGGATGAATTC | *att*B2-CAATAGCACATAAGCAC |
| | | | *att*B2-CAAACTTGGTGAATTGG |
| | *OMTN6* | *att*B1-TGGAGGAGGAGATGAGTT | *att*B2-CCAGCAGAGGAAAGAATAT |
| Yeast one-hybrid constructs | *OMTN1* | *CAGgaattcAAGATGCGATTAGCCAGG* | *CAGggatccATTTCAATAGGCCCAGAG* |
| | *OMTN2* | *CAGgaattcATGGAGCAGCATCAGG* | *CAGggatccACAGTAATCGCTACTTCG* |
| | *OMTN3* | *CAGgaattcTCGATGTCGATGATGAGCTT* | *CAGggatccCAACGCAAACCTCTACTTCTG* |
| | *OMTN4* | *GCTcatatgGAGCAGCATCGGTAGTCG* | *CAGctcgagTCCTCAATTCGCAACA* |
| | *OMTN6* | *CAGgaattcACCATTAGCTTGCTATTCTC* | *TTggatccGTGTGGCACTGTGAAATA* |
| Construction of the mutated forms of the *OMTNs* | *OMTN1*△TS | AGGAACTGGCCCTCCAGCGCTGGCGGGAGCGCG CACGGGG | GCGCTGGAGGGCCAGTTCCT |
| | *OMTN2*△TS | AAGAACTGGCCCTCCAGCGCCGTCGTCGCCGGG TCGACGG | GCGCTGGAGGGCCAGTTCTT |
| | *OMTN3*△TS | CTGGCAATGCTGTTGCTGAAAAGTGCGGCGACG GCGGCGA | TTCAGCAACAGCATTGCCAG |
| | *OMTN4*△TS | GATGGCTGTTGAGAGGGATTATAACCCTCTAGG TTTTGCA | AATCCCTCTCAACAGCCATC |
| | *OMTN6*△TS | CATTGGCAATGCCGGCAGTCGTAGCTCCCGACC ATC | CTGCCGGCATTGCCAAT |

| Purpose | Gene | Sense primer (5'→3') | Anti-sense primer (5'→3') |
|---|---|---|---|
| Construction of the mutated forms of the *OMTNs* | *OMTN1*SDM | CCGCCAG*CGCACGTGACgTGtTTtTCgA*ACGCGCTGGAGGGCCAG | CTGGCCCTCCAGCGCGT*TcGAaAAaCAcGTCACGTGCG*CTGGCGG |
| | *OMTN2*SDM | CGACGACGG*CGCACGTGACgTGtTTtTCgA*ACGCGCTGGAGGGCC | GGCCCTCCAGCGCGT*TcGAaAAaCAcGTCACGTGCG*CCGTCGTCG |
| | *OMTN3*SDM | CACTTG*AGCAAGTGCCgTGtTTtTCgA*GCTTCAGCAACAGCATTG | CAATGCTGTTGCTGAAGC*TcGAaAAaCAcGGCACTTGCT*CAAGTG |
| | *OMTN4*SDM | GGTTATG*AGCAAGTGCCgTGtTTtTCgA*ATAATCCCTCTCAACAG | CTGTTGAGAGGGATTAT*TcGAaAAaCAcGGCACTTGCT*CATAACC |
| | *OMTN6*SDM | GGAGCTACG*AGCAAGTGTCgTGtTTtTCgG*GCCTGCCGGCATTGC | GCAATGCCGGCAGGC*CcGAaAAaCAcGACACTTGCT*CGTAGCTCC |

**Supplementary Table S3. General information of the miR164-targeted genes in rice.**

| TIGR locus ID[a] | Sysmatic nomenclature[b] | Annotation | Name | Arabidopsis homolog | Target site | References[c] |
|---|---|---|---|---|---|---|
| LOC_Os02g36880 | ONAC027 | NAC | OMTN1 | At5g61430/ANAC100 | CDS | (Wu *et al.*, 2009; Li *et al.*, 2010) |
| LOC_Os04g38720 | ONAC004 | NAC | OMTN2 | At5g07680/ANAC080 | CDS | (Wu *et al.*, 2009; Li *et al.*, 2010) |
| LOC_Os12g41680 | ONAC060 | NAC | OMTN3 | At1g56010/ANAC022/NAC1 | CDS | (Wu *et al.*, 2009; Li *et al.*, 2010; Zhou *et al.*, 2010) |
| LOC_Os06g46270 | ONAC011 | NAC | OMTN4 | At1g56010/ANAC022/NAC1 | CDS | (Wu *et al.*, 2009; Li *et al.*, 2010) |
| LOC_Os06g23650 | ONAC092 | NAC | OMTN5 | At5g53950/ANAC098/CUC2 | CDS | (Wu *et al.*, 2009; Zhou et al., 2010) |
| LOC_Os08g10080 | ONAC104 | NAC | OMTN6 | At1g56010/ANAC022/NAC1 | CDS | (Li *et al.*, 2010) |
| LOC_Os03g50040 | | Phytanoyl-CoA dioxygenase | OMT7 | | 3′-UTR | (Li *et al.*, 2010) |
| LOC_Os05g39650 | | Phytanoyl-CoA dioxygenase | OMT8 | | 3′-UTR | (Li *et al.*, 2010) |
| LOC_Os12g05260 | | Phytosulfokines precursor | OMT9 | | CDS | (Wu *et al.*, 2009; Zhou *et al.*, 2010) |

[a]The locus ID were download from Rice Annotation Project (http://rice.plantbiology.msu.edu/).

[b]The systematic nomenclatures followed the nomenclatures in our previous publication (Fang *et al.*, 2008).

[c]References:

**Li YF, Zheng Y, Addo-Quaye C, Zhang L, Saini A, Jagadeeswaran G, Axtell MJ, Zhang W, Sunkar R**. 2010. Transcriptome-wide identification of microRNA targets in rice. *Plant J* **62**, 742-759.

**Wu L, Zhang Q, Zhou H, Ni F, Wu X, Qi Y. 2009.** Rice MicroRNA effector complexes and targets. *Plant Cell* **21**, 3421-3435.

**Zhou M, Gu L, Li P, Song X, Wei L, Chen Z, Cao X.** 2010. Degradome sequencing reveals endogenous small RNA targets in rice (*Oryza sativa* L. ssp. *indica*). *Frontiers in Biology* **5**, 67-90.

**Supplementary Table S4. Up- and down-regulated genes in the transgenic rice plants overexpressing *OMTN2*.**

| ProbeSetID | *OMTN2* -OE[a] | Stress response[b] | | | TIGR Locus ID[c] | Annotation |
|---|---|---|---|---|---|---|
| | | Drought | Salt | Cold | | |
| Os.21638.1.S1_at | 56.522 | 0.195 | 0.342 | 0.837 | LOC_Os01g46860 | major facilitator superfamily protein, expressed |
| Os.4463.1.S1_s_at | 17.729 | 18.958 | 3.674 | 8.821 | LOC_Os06g03670 | AP2 domain containing protein, expressed |
| Os.12498.2.S1_at | 17.030 | 8.186 | 13.129 | 1.328 | LOC_Os01g72360 | expressed protein |
| Os.6645.1.S1_at | 15.115 | 0.426 | 0.493 | 1.103 | LOC_Os07g25050 | thionin-like peptide, putative, expressed |
| Os.37969.2.S1_at | 13.867 | 0.936 | 0.186 | 4.805 | LOC_Os01g66860 | ankyrin-kinase, putative, expressed |
| Os.15829.1.S1_at | 12.744 | 0.382 | 1.711 | 0.307 | LOC_Os02g10120 | Lipoxygenase 2.3, chloroplast precursor, putative, expressed |
| Os.3710.1.S1_at | 11.783 | 1.230 | 1.677 | 5.611 | LOC_Os02g35440 | E3 ubiquitin ligase EL5, putative |
| Os.14105.1.S1_at | 11.729 | 6.569 | 13.264 | 12.204 | LOC_Os11g05380 | Cytochrome P450 family protein, expressed |
| Os.11365.1.S1_at | 10.263 | 3.342 | 1.305 | 2.895 | LOC_Os02g52210 | Zinc finger, C3HC4 type family protein, expressed |
| Os.4766.1.S1_at | 9.927 | 1.589 | 1.274 | 1.156 | LOC_Os10g28350 | 1,2-dihydroxy-3-keto-5-methylthiopentene dioxygenase, putative, expressed |
| Os.50053.1.A1_at | 9.335 | 220.836 | 66.154 | 3.686 | LOC_Os07g05940 | viviparous-14, putative, expressed |
| Os.56922.1.S1_at | 8.666 | 17.509 | 6.035 | 0.675 | LOC_Os02g26430 | WRKY DNA binding domain containing protein, expressed |
| Os.8088.1.S1_at | 8.187 | 2.006 | 0.947 | 10.718 | LOC_Os03g08310 | ZIM motif family protein, expressed |
| Os.49931.1.S1_at | 8.059 | 1.301 | 0.939 | 1.679 | LOC_Os11g43250 | Leucine Rich Repeat family protein, expressed |
| OsAffx.12799.1.S1_s_at | 8.057 | 16.089 | 12.316 | 2.156 | LOC_Os03g09170 | AP2 domain containing protein, expressed |
| Os.3808.4.S1_x_at | 7.265 | 2.061 | 1.227 | 5.266 | LOC_Os01g09220 | transposon protein, putative, CACTA, En/Spm sub-class, expressed |
| Os.56119.1.S1_at | 7.186 | 0.638 | 1.704 | 1.797 | LOC_Os02g15290 | VQ motif family protein, expressed |
| Os.18955.1.S1_at | 6.934 | 1.569 | 2.075 | 0.311 | LOC_Os06g51260 | myb-like DNA-binding domain, SHAQKYF class family protein, expressed |
| Os.51078.1.S1_at | 6.734 | 0.533 | 0.084 | 4.462 | LOC_Os02g45450 | AP2 domain containing protein, expressed |
| Os.46849.1.S1_at | 6.725 | 5.601 | 6.351 | 5.057 | LOC_Os10g25230 | ZIM motif family protein, expressed |
| OsAffx.28294.2.S1_at | 6.468 | 1.828 | 1.609 | 1.500 | LOC_Os07g05840 | expressed protein |
| Os.34982.1.A1_at | 6.415 | 0.348 | 0.828 | 0.751 | LOC_Os04g17660 | Rhodanese-like domain containing protein, expressed |
| OsAffx.24138.1.S1_s_at | 6.254 | 9.413 | 7.076 | 1.195 | LOC_Os02g06710 | expressed protein |

| ProbeSetID | *OMTN2* -OE[a] | Stress response[b] | | | TIGR Locus ID[c] | Annotation |
|---|---|---|---|---|---|---|
| | | Drought | Salt | Cold | | |
| Os.8823.1.S1_at | 5.943 | 0.948 | 0.474 | 0.351 | LOC_Os06g15430 | expressed protein |
| Os.34471.1.S1_at | 5.942 | 0.536 | 0.294 | 4.520 | LOC_Os01g64310 | NAC-domain containing protein 90, putative, expressed |
| Os.46956.1.S1_at | 5.593 | 31.068 | 5.481 | 3.056 | LOC_Os01g50940 | Helix-loop-helix DNA-binding domain containing protein, expressed |
| Os.26884.1.S1_a_at | 5.535 | 0.831 | 0.818 | 6.372 | LOC_Os01g67810 | transposon protein, putative, unclassified, expressed |
| Os.30575.1.A1_at | 5.531 | 4.360 | 0.880 | 3.560 | LOC_Os02g12470 | hypothetical protein |
| Os.57563.1.S1_s_at | 5.462 | 0.671 | 0.982 | 0.709 | LOC_Os04g53160 | NB-ARC domain containing protein, expressed |
| Os.11707.1.A1_at | 5.369 | 0.143 | 1.445 | 0.298 | LOC_Os03g54130 | Papain family cysteine protease containing protein, expressed |
| Os.140.3.S1_x_at | 5.339 | 2.249 | 2.320 | 10.973 | LOC_Os04g58810 | CAF1 family ribonuclease containing protein, expressed |
| Os.56966.1.S1_at | 5.187 | 0.149 | 0.238 | 1.015 | LOC_Os05g50390 | expressed protein |
| Os.32602.1.S1_at | 5.148 | 5.418 | 2.760 | 1.852 | LOC_Os01g74370 | expressed protein |
| Os.17916.1.S1_at | 5.029 | 0.780 | 0.990 | 0.951 | LOC_Os05g12630 | expressed protein |
| OsAffx.26826.1.S1_at | 4.943 | 0.062 | 0.152 | 1.096 | LOC_Os05g11210 | expressed protein |
| Os.37621.3.S1_at | 4.930 | 2.801 | 4.276 | 0.386 | LOC_Os01g02340 | Thaumatin family protein, expressed |
| Os.32366.1.S1_at | 4.863 | 9.541 | 26.752 | 28.954 | LOC_Os01g50420 | Protein kinase domain containing protein, expressed |
| Os.26695.1.S1_at | 4.725 | 5.053 | 3.805 | 3.669 | LOC_Os03g60080 | NAC-domain containing protein 19, putative, expressed |
| Os.51783.1.S1_at | 4.723 | 1.273 | 0.938 | 2.354 | LOC_Os05g37080 | NAC-domain containing protein 90, putative, expressed |
| Os.53403.1.S1_at | 4.694 | 0.628 | 1.307 | 1.300 | LOC_Os08g39830 | ETHYLENE-INSENSITIVE3-like 3 protein, putative, expressed |
| OsAffx.24280.2.S1_at | 4.651 | 0.496 | 0.821 | 0.379 | LOC_Os07g08150 | early light-inducible protein ELIP, putative, expressed |
| OsAffx.31986.1.S1_at | 4.645 | 38.019 | 5.287 | 3.341 | LOC_Os12g32610 | expressed protein |
| Os.47946.1.S1_s_at | 4.644 | 1.931 | 0.914 | 1.462 | LOC_Os07g33910 | Glucose-6-phosphate/phosphate translocator 2, chloroplast precursor, putative, expressed |
| Os.9923.1.S1_at | 4.583 | 2.859 | 2.641 | 7.944 | LOC_Os03g08330 | ZIM motif family protein, expressed |
| Os.5816.1.S1_at | 4.441 | 0.683 | 0.276 | 12.233 | LOC_Os09g35010 | AP2 domain containing protein, expressed |
| Os.10933.1.S1_at | 4.418 | 70.348 | 28.913 | 0.964 | LOC_Os01g50910 | Late embryogenesis abundant protein, expressed |

| ProbeSetID | *OMTN2* -OE[a] | Stress response[b] | | | TIGR Locus ID[c] | Annotation |
|---|---|---|---|---|---|---|
| | | Drought | Salt | Cold | | |
| Os.34873.1.S1_at | 4.418 | 0.890 | 0.539 | 0.437 | LOC_Os07g46510 | expressed protein |
| Os.10280.1.A1_at | 4.403 | 0.388 | 0.593 | 0.474 | LOC_Os02g04510 | NmrA-like family protein |
| Os.50015.1.S1_at | 4.383 | 5.523 | 21.830 | 5.004 | LOC_Os06g44010 | WRKY2 protein, putative, expressed |
| Os.10266.1.S1_at | 4.308 | 0.167 | 0.362 | 0.630 | LOC_Os03g43100 | expressed protein |
| Os.8045.1.S1_at | 4.291 | 0.138 | 0.936 | 0.922 | LOC_Os01g08440 | Indole-3-acetate beta-glucosyltransferase, putative, expressed |
| Os.57343.1.S1_at | 4.220 | 23.101 | 7.290 | 2.743 | LOC_Os02g50110 | expressed protein |
| Os.37376.1.S1_at | 4.172 | 3.195 | 0.609 | 1.233 | LOC_Os01g70820 | expressed protein |
| Os.26802.1.S1_at | 4.169 | 2.597 | 2.129 | 2.282 | LOC_Os12g08130 | amino acid transporter, putative, expressed |
| Os.12145.1.S1_at | 4.150 | 2.597 | 2.129 | 2.282 | LOC_Os02g47470 | Cytochrome P450 family protein, expressed |
| Os.39876.1.S1_at | 4.092 | 14.721 | 11.445 | 0.495 | LOC_Os04g48030 | HSF-type DNA-binding domain containing protein, expressed |
| Os.15849.1.S1_s_at | 4.092 | 1.795 | 5.921 | 5.572 | LOC_Os08g36920 | AP2 domain containing protein, expressed |
| Os.44475.1.S1_at | 4.076 | 24.155 | 14.608 | 3.051 | LOC_Os07g37730 | Rotenone-insensitive NADH-ubiquinone oxidoreductase, mitochondrial precursor, putative, expressed |
| Os.4184.1.S1_at | 4.072 | 0.343 | 1.364 | 0.904 | LOC_Os02g15350 | Dof domain, zinc finger family protein, expressed |
| Os.46725.1.S1_at | 4.026 | 0.888 | 0.634 | 0.322 | LOC_Os10g05130 | expressed protein |
| Os.7664.1.S1_at | 4.015 | 6.372 | 3.011 | 0.925 | LOC_Os02g56900 | expressed protein |
| Os.41109.1.S1_at | 4.000 | 3.906 | 6.748 | 0.367 | LOC_Os07g43740 | Zinc finger, C3HC4 type family protein, expressed |
| OsAffx.3652.1.S1_at | 3.989 | 14.125 | 5.769 | 4.019 | LOC_Os03g60210 | expressed protein |
| Os.4773.1.S1_at | 3.955 | 6.292 | 2.635 | 2.744 | LOC_Os06g04240 | expressed protein |
| OsAffx.27093.1.S1_at | 3.926 | 0.077 | 0.346 | 0.267 | LOC_Os05g31040 | Cytokinin dehydrogenase 1 precursor, putative, expressed |
| Os.6269.1.S1_at | 3.889 | 1.635 | 0.880 | 0.676 | LOC_Os03g19090 | expressed protein |
| Os.11570.1.S1_at | 3.764 | 5.185 | 2.134 | 1.343 | LOC_Os05g41590 | Glycerol-3-phosphate dehydrogenase, putative, expressed |
| Os.55984.1.S1_at | 3.763 | 3.634 | 2.166 | 2.055 | LOC_Os05g46840 | expressed protein |
| Os.57569.2.A1_s_at | 3.744 | 0.468 | 0.141 | 0.478 | LOC_Os08g04500 | Terpene synthase family, metal binding domain containing protein, expressed |

| ProbeSetID | *OMTN2* -OE[a] | Stress response[b] | | | TIGR Locus ID[c] | Annotation |
|---|---|---|---|---|---|---|
| | | Drought | Salt | Cold | | |
| Os.51764.1.S1_at | 3.722 | 0.950 | 0.848 | 1.143 | LOC_Os09g21290 | expressed protein |
| Os.606.1.S1_at | 3.674 | 9.617 | 11.730 | 5.006 | LOC_Os06g04220 | expressed protein |
| Os.6376.1.S1_at | 3.669 | 14.447 | 3.975 | 1.809 | LOC_Os06g10210 | expressed protein |
| Os.7902.1.S1_at | 3.647 | 2.454 | 1.608 | 2.546 | LOC_Os06g46950 | EF-hand Ca2+-binding protein CCD1, putative, expressed |
| Os.17047.1.A1_at | 3.627 | 0.564 | 1.205 | 0.910 | LOC_Os07g18230 | Protein kinase domain containing protein, expressed |
| Os.5047.1.S1_at | 3.612 | 1.232 | 1.265 | 0.679 | LOC_Os07g08830 | expressed protein |
| Os.12112.1.S1_at | 3.599 | 0.299 | 0.617 | 0.152 | LOC_Os01g60830 | expressed protein |
| OsAffx.25073.1.S1_x_at | 3.598 | 0.202 | 0.607 | 0.286 | LOC_Os03g12700 | expressed protein |
| Os.27484.1.S1_at | 3.595 | 2.221 | 1.585 | 0.987 | LOC_Os02g06930 | Protein kinase domain containing protein, expressed |
| Os.51479.1.S1_at | 3.522 | 0.994 | 1.228 | 0.189 | LOC_Os09g25720 | expressed protein |
| Os.9212.1.S1_at | 3.411 | 0.565 | 0.756 | 0.722 | LOC_Os07g22930 | Granule-bound starch synthase 1b, chloroplast precursor, putative, expressed |
| Os.11952.1.S1_at | 3.397 | 6.661 | 3.982 | 4.394 | LOC_Os02g11070 | Fatty acid elongase, putative, expressed |
| OsAffx.31235.1.S1_at | 3.365 | 1.047 | 0.567 | 1.150 | LOC_Os11g35300 | hypothetical protein |
| Os.7475.1.S1_at | 3.332 | 3.277 | 2.881 | 0.486 | LOC_Os01g74450 | Aquaporin TIP1.1, putative, expressed |
| Os.49787.1.S1_at | 3.317 | 18.028 | 4.606 | 3.199 | LOC_Os01g64360 | Myb-like DNA-binding domain containing protein, expressed |
| Os.5044.1.S1_at | 3.295 | 11.539 | 15.448 | 13.380 | LOC_Os01g50410 | Protein kinase domain containing protein, expressed |
| Os.37067.1.S1_at | 3.295 | 1.400 | 0.516 | 5.539 | LOC_Os10g37760 | Rhomboid family protein, expressed |
| OsAffx.29084.2.S1_x_at | 3.292 | 4.260 | 2.040 | 0.105 | LOC_Os08g07630 | expressed protein |
| OsAffx.25720.1.S1_at | 3.292 | 0.814 | 1.029 | 0.886 | LOC_Os03g55460 | expressed protein |
| Os.56943.1.S1_at | 3.291 | 1.140 | 0.835 | 1.191 | LOC_Os08g01330 | no apical meristem, putative, expressed |
| Os.7309.1.S1_at | 3.290 | 0.166 | 0.294 | 0.631 | LOC_Os12g01360 | expressed protein |
| Os.3388.2.S1_a_at | 3.269 | 2.702 | 4.005 | 7.389 | LOC_Os04g43680 | Myb-related protein Myb4, putative, expressed |
| Os.30568.1.S1_at | 3.259 | 2.103 | 0.825 | 4.850 | LOC_Os01g60640 | WRKY DNA binding domain containing protein, expressed |
| Os.7665.1.S1_at | 3.255 | 0.086 | 0.470 | 0.307 | LOC_Os04g56240 | Lipase family protein, expressed |

| ProbeSetID | *OMTN2*-OE[a] | Stress response[b] | | | TIGR Locus ID[c] | Annotation |
|---|---|---|---|---|---|---|
| | | Drought | Salt | Cold | | |
| Os.5698.1.S1_s_at | 3.251 | 7.643 | 4.452 | 1.392 | LOC_Os05g33700 | 4F5 protein family protein |
| Os.11804.1.S1_at | 3.248 | 0.237 | 0.393 | 0.215 | LOC_Os03g14910 | Tryptophan/tyrosine permease family protein, expressed |
| Os.11573.2.A2_a_at | 3.247 | 0.613 | 0.881 | 0.935 | LOC_Os11g47970 | Ribulose bisphosphate carboxylase/oxygenase activase, chloroplast precursor, putative, expressed |
| Os.32177.1.S1_at | 3.232 | 1.371 | 1.276 | 0.777 | LOC_Os05g50930 | Sigma-70, region 4 family protein, expressed |
| OsAffx.24831.1.S1_s_at | 3.230 | 0.421 | 0.600 | 0.606 | LOC_Os02g52270 | Chaperone protein dnaJ, putative, expressed |
| Os.10091.1.S1_at | 3.226 | 1.053 | 0.635 | 17.566 | LOC_Os03g19070 | long cell-linked locus protein, putative, expressed |
| Os.38806.1.A1_x_at | 3.203 | 12.732 | 5.207 | 6.969 | LOC_Os06g04230 | expressed protein |
| Os.31521.1.S1_at | 3.197 | 4.993 | 7.850 | 2.371 | LOC_Os01g61080 | DNA-binding protein, putative, expressed |
| Os.56080.1.S1_at | 3.194 | 2.705 | 5.518 | 2.543 | LOC_Os03g47280 | VQ motif family protein, expressed |
| Os.9103.1.S1_at | 3.193 | 8.130 | 3.924 | 1.551 | LOC_Os09g09930 | heavy metal-associated domain containing protein, expressed |
| Os.8480.1.S1_at | 3.175 | 0.243 | 0.186 | 2.271 | LOC_Os05g30700 | expressed protein |
| Os.16903.1.A1_at | 3.165 | 0.341 | 0.341 | 0.306 | LOC_Os05g06920 | RelA/SpoT containing protein, expressed |
| OsAffx.14373.1.S1_s_at | 3.105 | 4.043 | 2.684 | 3.111 | LOC_Os04g52090 | AP2 domain containing protein, expressed |
| Os.536.1.S1_at | 3.095 | 0.464 | 0.610 | 0.701 | LOC_Os01g05840 | oxidoreductase, short chain dehydrogenase/reductase family protein, expressed |
| Os.14921.1.S1_at | 3.093 | 0.118 | 0.464 | 0.089 | LOC_Os03g56930 | expressed protein |
| Os.22651.1.S1_at | 3.091 | 0.125 | 0.288 | 0.280 | LOC_Os04g56230 | Farnesyl pyrophosphate synthetase 2, putative, expressed |
| Os.10556.1.S1_at | 3.081 | 8.593 | 9.683 | 0.495 | LOC_Os02g15860 | expressed protein |
| Os.6244.1.S1_x_at | 3.066 | 9.191 | 2.324 | 3.018 | LOC_Os03g13740 | U-box domain containing protein, expressed |
| Os.22485.1.A1_at | 3.038 | 0.273 | 0.324 | 1.008 | LOC_Os11g29720 | Cytochrome P450 family protein, expressed |
| Os.4384.1.S1_at | 3.036 | 5.323 | 5.230 | 4.279 | LOC_Os01g60020 | No apical meristem protein, expressed |
| Os.45018.1.S1_x_at | 3.026 | 0.273 | 0.456 | 0.447 | LOC_Os03g25500 | Cytochrome P450 72A1, putative, expressed |
| Os.2915.1.S1_at | 3.025 | 3.667 | 2.459 | 2.441 | LOC_Os01g21250 | Late embryogenesis abundant protein |
| Os.24911.1.S1_at | 3.024 | 0.450 | 0.449 | 0.540 | LOC_Os03g62170 | Streptomyces cyclase/dehydrase family protein, expressed |

| ProbeSetID | *OMTN2* -OE[a] | Stress response[b] | | | TIGR Locus ID[c] | Annotation |
|---|---|---|---|---|---|---|
| | | Drought | Salt | Cold | | |
| Os.53473.1.S1_at | 3.023 | 1.487 | 1.148 | 1.809 | LOC_Os03g05460 | expressed protein |
| OsAffx.21073.1.S1_at | 3.022 | 0.559 | 0.401 | 0.494 | LOC_Os07g02620 | NB-ARC domain containing protein |
| OsAffx.12415.1.S1_at | 3.019 | 1.102 | 0.455 | 0.611 | LOC_Os02g39510 | hypothetical protein |
| Os.52597.1.S1_at | 3.018 | 88.118 | 55.049 | 0.267 | LOC_Os04g59540 | Phosphatidylinositol-4-phosphate 5-Kinase family protein, expressed |
| Os.34373.1.S1_at | 3.006 | 1.987 | 1.137 | 0.883 | LOC_Os12g12580 | NADP-dependent oxidoreductase P2, putative, expressed |
| Os.20169.1.S1_at | 2.998 | 0.421 | 0.527 | 0.578 | LOC_Os06g34730 | expressed protein |
| Os.50470.1.S1_at | 2.996 | 3.270 | 4.170 | 0.793 | LOC_Os01g71310 | Cytokinin dehydrogenase 1 precursor, putative, expressed |
| Os.15908.1.S1_s_at | 2.958 | 1.366 | 0.755 | 0.689 | LOC_Os01g72330 | Two-component response regulator ARR8, putative, expressed |
| Os.11474.1.S1_at | 2.957 | 3.998 | 2.172 | 1.975 | LOC_Os02g51350 | kelch repeat-containing F-box family protein, putative, expressed |
| OsAffx.24157.1.S1_s_at | 2.953 | 3.713 | 3.360 | 2.047 | LOC_Os02g07930 | B-box zinc finger family protein, expressed |
| OsAffx.19886.1.S1_at | 2.930 | 0.447 | 0.673 | 1.014 | LOC_Os12g29680 | hypothetical protein |
| Os.54440.1.S1_at | 2.914 | 0.392 | 0.420 | 0.987 | LOC_Os04g53180 | expressed protein |
| Os.6516.1.S1_at | 2.913 | 10.697 | 2.074 | 1.328 | LOC_Os07g33280 | expressed protein |
| Os.5591.1.S1_at | 2.910 | 1.110 | 1.037 | 1.696 | LOC_Os07g02790 | expressed protein |
| Os.49619.1.S1_at | 2.886 | 1.561 | 2.457 | 7.958 | LOC_Os03g20090 | Myb-like DNA-binding domain containing protein, expressed |
| Os.9191.1.S1_s_at | 2.875 | 2.661 | 1.507 | 0.139 | LOC_Os01g14410 | Early light-induced protein, chloroplast precursor, putative, expressed |
| OsAffx.26673.1.S1_at | 2.873 | 0.763 | 0.852 | 0.754 | LOC_Os05g01010 | hypothetical protein |
| Os.17889.1.S1_at | 2.869 | 7.274 | 2.504 | 1.464 | LOC_Os05g46340 | expressed protein |
| Os.23606.1.S1_at | 2.852 | 1.896 | 1.474 | 2.933 | LOC_Os01g29330 | expressed protein |
| OsAffx.26443.1.S1_s_at | 2.851 | 2.395 | 1.695 | 5.653 | LOC_Os04g43440 | NB-ARC domain containing protein, expressed |
| Os.4020.1.S1_at | 2.844 | 0.523 | 0.862 | 0.839 | LOC_Os10g02380 | NADH-dependent oxidoreductase 1, putative, expressed |
| Os.8961.1.S1_s_at | 2.840 | 3.207 | 4.229 | 4.041 | LOC_Os05g46020 | WRKY DNA binding domain containing protein, expressed |
| Os.7991.1.S1_at | 2.840 | 3.486 | 1.811 | 1.730 | LOC_Os04g41620 | Endochitinase A precursor, putative, expressed |
| Os.10751.1.S1_at | 2.836 | 1.064 | 1.182 | 0.886 | LOC_Os10g41100 | CCT motif family protein, expressed |

| ProbeSetID | *OMTN2* -OE[a] | Stress response[b] | | | TIGR Locus ID[c] | Annotation |
|---|---|---|---|---|---|---|
| | | Drought | Salt | Cold | | |
| Os.28290.1.S1_at | 2.832 | 0.096 | 0.405 | 0.223 | LOC_Os02g47510 | nine-cis-epoxycarotenoid dioxygenase4, putative, expressed |
| Os.6671.2.S1_x_at | 2.827 | 6.565 | 4.878 | 2.961 | LOC_Os05g39930 | U-box domain containing protein, expressed |
| Os.11941.2.S1_at | 2.823 | 10.409 | 8.796 | 0.409 | LOC_Os09g35790 | HSF-type DNA-binding domain containing protein, expressed |
| Os.40021.1.S1_a_at | 2.822 | 0.249 | 0.500 | 0.259 | LOC_Os03g06630 | Heat shock factor protein 1, putative, expressed |
| Os.49999.1.S1_x_at | 2.819 | 1.434 | 0.796 | 2.529 | LOC_Os03g18910 | COBRA-like protein 7 precursor, putative, expressed |
| Os.52264.1.S1_at | 2.817 | 1.882 | 1.706 | 1.706 | LOC_Os05g25370 | Protein kinase domain containing protein, expressed |
| Os.34459.1.S1_at | 2.817 | 0.305 | 0.467 | 0.426 | LOC_Os10g28360 | 1,2-dihydroxy-3-keto-5-methylthiopentene dioxygenase, putative, expressed |
| Os.9592.1.S1_at | 2.815 | 1.380 | 1.291 | 1.504 | LOC_Os07g41310 | COBRA-like protein 2 precursor, putative, expressed |
| Os.53660.1.S1_at | 2.811 | 3.266 | 7.603 | 5.744 | LOC_Os02g43790 | AP2 domain containing protein, expressed |
| Os.35808.1.S1_at | 2.809 | 9.547 | 3.532 | 6.845 | LOC_Os01g42190 | DnaJ domain containing protein, expressed |
| Os.14971.1.S1_at | 2.803 | 0.648 | 0.363 | 1.566 | LOC_Os08g40850 | Mitochondrial carrier protein, expressed |
| Os.49711.1.S1_at | 2.772 | 0.672 | 0.887 | 2.310 | LOC_Os08g35110 | auxin-responsive protein, putative, expressed |
| Os.1316.1.S2_at | 2.759 | 72.211 | 19.117 | 0.781 | LOC_Os02g04780 | expressed protein |
| Os.51937.1.S1_a_at | 2.757 | 3.690 | 2.051 | 0.980 | LOC_Os07g09670 | galactosyltransferase family protein, putative, expressed |
| Os.9880.1.S1_a_at | 2.752 | 0.278 | 0.623 | 0.507 | LOC_Os01g68480 | Thioredoxin F-type 2, chloroplast precursor, putative, expressed |
| Os.15142.1.S1_at | 2.751 | 1.031 | 1.591 | 0.992 | LOC_Os04g49350 | pentatricopeptide, putative, expressed |
| Os.7115.1.S1_a_at | 2.750 | 1.020 | 1.417 | 3.359 | LOC_Os04g57200 | heavy metal-associated domain containing protein, expressed |
| Os.53946.1.S1_at | 2.727 | 1.383 | 0.742 | 1.392 | LOC_Os08g41440 | NAD dependent epimerase/dehydratase family protein, expressed |
| Os.50590.2.A1_at | 2.714 | 0.307 | 0.696 | 1.025 | LOC_Os02g07830 | Cation transport protein, expressed |
| Os.53795.1.S1_at | 2.710 | 4.364 | 1.556 | 1.650 | LOC_Os03g08840 | expressed protein |
| Os.39075.1.S1_at | 2.699 | 1.862 | 0.693 | 1.058 | LOC_Os03g12990 | Phytosulfokine precursor protein containing protein, expressed |
| Os.8117.1.S1_at | 2.689 | 0.408 | 0.444 | 0.682 | LOC_Os04g36070 | Response regulator receiver domain containing protein, expressed |
| Os.57301.1.S1_at | 2.689 | 4.080 | 1.051 | 4.960 | LOC_Os02g52170 | expressed protein |
| Os.12257.1.S1_at | 2.687 | 4.935 | 6.673 | 1.336 | LOC_Os02g52150 | Hsp20/alpha crystallin family protein, expressed |

| ProbeSetID | *OMTN2* -OE[a] | Stress response[b] | | | TIGR Locus ID[c] | Annotation |
|---|---|---|---|---|---|---|
| | | Drought | Salt | Cold | | |
| Os.16422.1.S1_s_at | 2.684 | 3.893 | 4.204 | 0.541 | LOC_Os08g15050 | CCT motif family protein, expressed |
| Os.47778.1.A1_s_at | 2.677 | 7.513 | 0.887 | 1.244 | LOC_Os04g33640 | Glycosyl hydrolases family 17 protein, expressed |
| Os.52678.1.S1_at | 2.674 | 1.101 | 1.023 | 1.110 | LOC_Os04g41130 | FLOWERING LOCUS T protein, putative, expressed |
| Os.8868.1.S1_at | 2.674 | 1.110 | 1.678 | 0.118 | LOC_Os10g38610 | glutathione S-transferase GSTU6, putative, expressed |
| Os.20355.1.S1_at | 2.668 | 0.345 | 0.465 | 1.992 | LOC_Os01g72100 | Polcalcin, putative, expressed |
| OsAffx.12542.1.S1_at | 2.664 | 0.286 | 0.400 | 0.571 | LOC_Os02g47920 | exonuclease family protein |
| Os.8118.1.S1_at | 2.643 | 1.059 | 1.799 | 0.592 | LOC_Os07g26630 | Aquaporin PIP2.2, putative, expressed |
| Os.11023.1.S2_a_at | 2.637 | 6.643 | 1.000 | 2.000 | LOC_Os01g51890 | inositol polyphosphate 5-phosphatase, putative, expressed |
| Os.9677.2.S1_x_at | 2.616 | 10.300 | 3.226 | 1.647 | LOC_Os01g62670 | Avr9/Cf-9 rapidly elicited protein 137, putative, expressed |
| Os.54336.1.S1_at | 2.611 | 2.493 | 0.932 | 1.927 | LOC_Os02g03420 | PPR868-14, putative, expressed |
| Os.51529.1.S1_at | 2.597 | 2.037 | 1.598 | 1.825 | LOC_Os12g07550 | expressed protein |
| Os.55575.1.S1_at | 2.589 | 1.099 | 3.508 | 0.557 | LOC_Os05g02200 | 33 kDa secretory protein, putative, expressed |
| Os.41637.1.S1_at | 2.587 | 0.323 | 0.546 | 0.354 | LOC_Os01g50080 | ABC transporter family protein, expressed |
| Os.5549.1.S1_at | 2.577 | 1.355 | 3.672 | 4.001 | LOC_Os07g12340 | NAC-domain containing protein 2, putative, expressed |
| OsAffx.9584.1.S1_x_at | 2.574 | 2.183 | 0.550 | 3.354 | LOC_Os01g60600 | WRKY DNA binding domain containing protein, expressed |
| OsAffx.24166.1.S1_at | 2.559 | 5.067 | 5.892 | 7.203 | LOC_Os02g08440 | WRKY transcription factor, putative, expressed |
| OsAffx.22270.1.S1_x_at | 2.555 | 1.013 | 1.172 | 1.286 | LOC_Os03g08940 | hypothetical protein |
| Os.11766.1.S1_at | 2.536 | 5.054 | 2.341 | 1.992 | LOC_Os07g36170 | Chitin-inducible gibberellin-responsive protein 1, putative, expressed |
| OsAffx.12052.1.S1_at | 2.532 | 0.242 | 1.220 | 1.406 | LOC_Os02g15280 | VQ motif family protein |
| Os.26965.1.S1_at | 2.531 | 0.278 | 0.504 | 0.760 | LOC_Os01g61120 | expressed protein |
| Os.17036.1.S1_x_at | 2.515 | 3.236 | 3.255 | 1.315 | LOC_Os05g03620 | protein kinase family protein, putative, expressed |
| Os.27628.1.S1_at | 2.514 | 0.191 | 0.288 | 0.552 | LOC_Os03g62670 | Thiopurine S-methyltransferase family protein, expressed |
| Os.9301.1.S1_at | 2.506 | 0.299 | 0.608 | 1.258 | LOC_Os08g28670 | ripening induced protein, putative, expressed |
| Os.27497.1.S1_at | 2.506 | 0.191 | 0.121 | 0.681 | LOC_Os12g12390 | transposon protein, putative, CACTA, En/Spm sub-class, expressed |

| ProbeSetID | *OMTN2* -OE[a] | Stress response[b] | | | TIGR Locus ID[c] | Annotation |
|---|---|---|---|---|---|---|
| | | Drought | Salt | Cold | | |
| Os.30866.1.S1_at | 2.500 | 6.833 | 5.345 | 2.740 | LOC_Os07g02460 | expressed protein |
| Os.10003.1.S1_at | 2.486 | 0.636 | 1.071 | 0.977 | LOC_Os01g13390 | expressed protein |
| Os.2426.1.A1_at | 2.484 | 7.719 | 20.711 | 14.222 | LOC_Os11g10470 | expressed protein |
| Os.8112.1.S1_at | 2.483 | 0.537 | 0.650 | 0.587 | LOC_Os07g04990 | oxidoreductase, aldo/keto reductase family protein, expressed |
| Os.31191.1.S1_at | 2.480 | 0.592 | 1.470 | 0.685 | LOC_Os07g26660 | expressed protein |
| Os.4604.1.S1_at | 2.478 | 1.178 | 1.023 | 0.841 | LOC_Os03g15460 | expressed protein |
| Os.27682.1.S1_at | 2.475 | 1.129 | 0.772 | 0.667 | LOC_Os08g14570 | NADPH-cytochrome P450 reductase, putative, expressed |
| Os.16997.1.S1_at | 2.466 | 0.848 | 0.540 | 0.985 | LOC_Os03g07960 | expressed protein |
| Os.6085.1.S1_at | 2.463 | 9.775 | 4.825 | 13.602 | LOC_Os05g46760 | Protein kinase domain containing protein, expressed |
| Os.43040.1.S1_at | 2.460 | 2.777 | 1.123 | 1.309 | LOC_Os01g19940 | Negatively light-regulated protein, putative, expressed |
| Os.23136.1.A1_s_at | 2.459 | 0.602 | 1.064 | 0.687 | LOC_Os11g42930 | expressed protein |
| Os.55479.1.S1_at | 2.457 | 71.204 | 43.680 | 1.540 | LOC_Os05g41490 | circadian clock coupling factor ZGT, putative, expressed |
| Os.47625.1.A1_s_at | 2.451 | 0.956 | 0.614 | 1.078 | LOC_Os05g04500 | Peroxidase family protein, expressed |
| Os.34387.1.S1_at | 2.448 | 0.802 | 0.193 | 0.760 | LOC_Os01g24790 | Transferase family protein, expressed |
| Os.26472.1.S1_at | 2.438 | 1.382 | 1.117 | 2.121 | LOC_Os04g40630 | TAZ zinc finger family protein, expressed |
| Os.15692.1.S1_at | 2.436 | 0.854 | 0.586 | 0.467 | LOC_Os03g39760 | Cytochrome P450 family protein, expressed |
| Os.6043.1.S1_at | 2.432 | 2.123 | 3.429 | 2.395 | LOC_Os04g23550 | Helix-loop-helix DNA-binding domain containing protein, expressed |
| Os.14145.1.A1_at | 2.426 | 0.363 | 0.899 | 0.605 | LOC_Os04g36720 | ferric reductase-like transmembrane component family protein, putative, expressed |
| Os.49765.1.S1_at | 2.425 | 1.196 | 0.879 | 0.803 | LOC_Os04g45860 | transposon protein, putative, unclassified, expressed |
| Os.8569.1.S1_at | 2.425 | 0.430 | 0.657 | 0.864 | LOC_Os04g48880 | beta-carotene hydroxylase, putative, expressed |
| Os.9073.1.S1_at | 2.425 | 3.551 | 2.269 | 4.206 | LOC_Os01g57470 | EF hand family protein, expressed |
| OsAffx.15712.1.S1_at | 2.413 | 0.681 | 1.327 | 0.460 | LOC_Os06g33420 | transposase, putative |
| Os.51734.2.S1_at | 2.410 | 0.925 | 0.710 | 0.615 | LOC_Os02g47980 | uncharacterized plant-specific domain TIGR01570 family protein, expressed |

| ProbeSetID | *OMTN2* -OE[a] | Stress response[b] | | | TIGR Locus ID[c] | Annotation |
|---|---|---|---|---|---|---|
| | | Drought | Salt | Cold | | |
| Os.18305.1.S1_at | 2.404 | 0.201 | 0.492 | 0.424 | LOC_Os05g50550 | Polyprenyl synthetase family protein, expressed |
| Os.18922.2.S1_at | 2.403 | 17.632 | 7.684 | 9.289 | LOC_Os01g72530 | EF hand family protein, expressed |
| Os.17677.1.S1_at | 2.400 | 3.101 | 1.985 | 3.218 | LOC_Os06g03810 | expressed protein |
| Os.9022.1.S1_at | 2.393 | 103.999 | 55.190 | 0.862 | LOC_Os09g15670 | Protein phosphatase 2C, putative, expressed |
| Os.23113.1.S1_at | 2.391 | 1.314 | 1.140 | 0.630 | LOC_Os04g34610 | expressed protein |
| Os.15283.1.S1_at | 2.387 | 1.121 | 0.840 | 0.841 | LOC_Os04g57310 | Uncharacterized conserved protein, putative, expressed |
| Os.49763.2.S1_at | 2.381 | 1.269 | 1.096 | 1.070 | LOC_Os03g17230 | NAD-dependent epimerase/dehydratase family protein, putative, expressed |
| Os.15284.1.S1_at | 2.379 | 1.365 | 0.498 | 3.293 | LOC_Os07g48280 | expressed protein |
| Os.12119.1.S1_at | 2.378 | 3.009 | 0.912 | 5.715 | LOC_Os01g64470 | Harpin-induced protein 1 containing protein, expressed |
| Os.34191.1.S1_at | 2.373 | 0.675 | 0.932 | 0.574 | LOC_Os07g46630 | adenosine/AMP deaminase family protein, putative, expressed |
| Os.1385.1.S1_at | 2.370 | 1.305 | 1.446 | 1.301 | LOC_Os01g71670 | Glucan endo-1,3-beta-glucosidase GII precursor, putative, expressed |
| OsAffx.27219.1.S1_at | 2.369 | 50.407 | 27.327 | 1.124 | LOC_Os05g38290 | Protein phosphatase 2C, putative, expressed |
| OsAffx.17361.1.S1_at | 2.369 | 0.159 | 0.220 | 0.732 | LOC_Os08g36680 | Eukaryotic-type carbonic anhydrase family protein, expressed |
| Os.46160.1.S1_at | 2.367 | 0.161 | 0.290 | 1.613 | LOC_Os10g30790 | phosphate:H+ symporter family protein, expressed |
| Os.50476.1.S1_at | 2.362 | 0.724 | 0.835 | 0.394 | LOC_Os04g44930 | expressed protein |
| Os.9996.1.S1_at | 2.359 | 0.155 | 0.853 | 0.799 | LOC_Os11g10990 | DNAJ heat shock N-terminal domain-containing protein, putative, expressed |
| Os.10099.1.S1_at | 2.348 | 4.615 | 2.089 | 0.498 | LOC_Os03g13870 | expressed protein |
| Os.10410.1.S1_at | 2.345 | 0.118 | 0.468 | 0.302 | LOC_Os08g02210 | expressed protein |
| Os.5160.1.S1_at | 2.345 | 0.256 | 0.307 | 0.919 | LOC_Os07g46330 | expressed protein |
| Os.23163.1.S1_at | 2.333 | 0.757 | 0.853 | 0.756 | LOC_Os04g53310 | Soluble starch synthase 3, chloroplast precursor, putative, expressed |
| Os.31022.1.S1_at | 2.330 | 84.798 | 13.899 | 1.023 | LOC_Os06g02040 | Late embryogenesis abundant group 1 family protein, expressed |
| Os.14115.1.S1_at | 2.330 | 0.618 | 0.720 | 0.771 | LOC_Os01g51980 | Allinase, C-terminal domain containing protein, expressed |
| Os.11065.1.A1_at | 2.329 | 1.191 | 0.512 | 1.153 | LOC_Os03g19600 | retrotransposon protein, putative, Ty3-gypsy subclass, expressed |
| Os.51026.1.S1_x_at | 2.321 | 0.624 | 0.611 | 0.797 | LOC_Os07g30620 | UDP-glucoronosyl and UDP-glucosyl transferase family protein, expressed |

| ProbeSetID | *OMTN2* -OE[a] | Stress response[b] | | | TIGR Locus ID[c] | Annotation |
|---|---|---|---|---|---|---|
| | | Drought | Salt | Cold | | |
| Os.24873.1.S1_at | 2.321 | 0.486 | 0.538 | 1.368 | LOC_Os05g30250 | Glycosyl hydrolase family 1 protein, expressed |
| Os.23103.1.S1_at | 2.318 | 2.238 | 1.173 | 2.251 | LOC_Os05g41780 | AP2 domain containing protein, expressed |
| Os.52493.1.A1_s_at | 2.313 | 0.510 | 1.189 | 1.083 | LOC_Os03g22210 | expressed protein |
| Os.50975.1.S1_at | 2.312 | 0.314 | 0.567 | 1.112 | LOC_Os05g49370 | expressed protein |
| Os.34438.1.S1_x_at | 2.311 | 54.833 | 9.800 | 2.383 | LOC_Os07g39740 | Esterase precursor, putative, expressed |
| OsAffx.16913.1.S1_x_at | 2.311 | 0.092 | 0.294 | 0.342 | LOC_Os08g09310 | expressed protein |
| Os.49816.1.S1_at | 2.304 | 2.137 | 1.860 | 0.726 | LOC_Os04g51040 | wall-associated kinase 1, putative, expressed |
| Os.32454.1.S1_at | 2.300 | 0.545 | 0.782 | 1.234 | LOC_Os01g01650 | Isoflavone reductase homolog IRL, putative, expressed |
| Os.7130.2.S1_x_at | 2.298 | 2.349 | 1.300 | 1.405 | LOC_Os10g42690 | transcription factor jumonji, putative, expressed |
| Os.27836.1.A1_s_at | 2.296 | 0.484 | 0.514 | 0.713 | LOC_Os05g43650 | expressed protein |
| Os.7028.1.S1_at | 2.292 | 0.599 | 0.683 | 1.715 | LOC_Os02g45710 | Zinc finger, C3HC4 type family protein, expressed |
| Os.51753.1.A1_at | 2.282 | 3.741 | 0.719 | 1.086 | LOC_Os12g35340 | Glutaredoxin-like family protein, expressed |
| Os.5365.1.S1_at | 2.279 | 2.674 | 2.404 | 2.535 | LOC_Os02g21040 | Eukaryotic aspartyl protease family protein, expressed |
| OsAffx.15538.1.S1_at | 2.278 | 0.108 | 0.111 | 0.525 | LOC_Os06g21570 | Glycosyl hydrolase family 1 protein, expressed |
| Os.53074.1.S1_at | 2.266 | 1.341 | 5.349 | 8.467 | LOC_Os03g20330 | VQ motif family protein, expressed |
| OsAffx.15533.1.S1_at | 2.262 | 0.858 | 1.435 | 0.773 | LOC_Os06g21140 | Glycine rich protein family protein, expressed |
| Os.30528.1.S1_at | 2.260 | 52.308 | 18.077 | 0.923 | LOC_Os08g31860 | expressed protein |
| Os.6189.1.S1_s_at | 2.260 | 0.894 | 0.801 | 0.711 | LOC_Os09g27060 | chromatin complex subunit A101, putative, expressed |
| Os.5431.1.S1_at | 2.259 | 0.931 | 0.808 | 1.594 | LOC_Os04g49690 | protein kinase family protein, putative, expressed |
| OsAffx.7464.1.S1_s_at | 2.259 | 0.521 | 0.301 | 0.572 | LOC_Os12g02750 | expressed protein |
| Os.51630.1.A1_at | 2.246 | 1.376 | 0.942 | 1.313 | LOC_Os02g03430 | expressed protein |
| Os.32072.1.S1_at | 2.244 | 0.154 | 0.468 | 0.246 | LOC_Os08g33540 | ATP-dependent Clp protease adaptor protein ClpS containing protein, expressed |
| Os.26932.1.S1_at | 2.244 | 9.738 | 3.410 | 4.802 | LOC_Os07g37320 | Glucose transporter, putative, expressed |

| ProbeSetID | *OMTN2* -OE[a] | Stress response[b] | | | TIGR Locus ID[c] | Annotation |
|---|---|---|---|---|---|---|
| | | Drought | Salt | Cold | | |
| Os.27080.1.S1_at | 2.237 | 0.643 | 1.341 | 0.567 | LOC_Os02g36450 | Sugar transporter family protein, expressed |
| OsAffx.6057.1.S1_s_at | 2.236 | 0.926 | 1.426 | 1.648 | LOC_Os08g37370 | Mitochondrial carrier protein, expressed |
| Os.5390.1.S1_at | 2.234 | 1.123 | 1.416 | 2.828 | LOC_Os12g33130 | expressed protein |
| OsAffx.25749.1.S1_s_at | 2.234 | 26.820 | 6.830 | 0.981 | LOC_Os03g57880 | Glucan endo-1,3-beta-glucosidase 5 precursor, putative, expressed |
| Os.55674.1.S1_at | 2.233 | 3.286 | 1.114 | 1.829 | LOC_Os05g46830 | expressed protein |
| Os.50268.1.S1_at | 2.232 | 2.130 | 1.294 | 1.173 | LOC_Os05g45440 | expressed protein |
| Os.21300.2.S1_at | 2.212 | 0.841 | 0.609 | 0.963 | LOC_Os01g42690 | Phospholipase/Carboxylesterase family protein, expressed |
| Os.20187.2.S1_at | 2.211 | 0.936 | 1.058 | 0.514 | LOC_Os01g61010 | Nodulin-like family protein, expressed |
| Os.9201.2.S1_x_at | 2.211 | 0.261 | 0.621 | 0.990 | LOC_Os02g40010 | Adenine phosphoribosyltransferase 2, putative, expressed |
| Os.52987.1.S1_at | 2.209 | 2.100 | 1.714 | 0.858 | LOC_Os09g28620 | esterase, putative, expressed |
| Os.50329.1.S1_s_at | 2.209 | 10.860 | 7.000 | 1.119 | LOC_Os08g36860 | Cytochrome P450 family protein, expressed |
| Os.17814.2.S1_x_at | 2.209 | 0.321 | 0.684 | 0.599 | LOC_Os07g43700 | Malate dehydrogenase, glyoxysomal precursor, putative, expressed |
| Os.51439.1.S1_x_at | 2.207 | 4.281 | 1.075 | 2.889 | LOC_Os04g30420 | Chloroplastic quinone-oxidoreductase homolog, putative, expressed |
| Os.6092.1.S1_at | 2.206 | 1.773 | 0.494 | 7.560 | LOC_Os02g44230 | trehalose-phosphatase family protein, expressed |
| Os.46864.1.S1_at | 2.205 | 2.214 | 3.143 | 2.690 | LOC_Os10g07080 | expressed protein |
| Os.24876.1.S1_s_at | 2.203 | 0.360 | 0.555 | 0.508 | LOC_Os06g14350 | Calcium binding EF-hand protein, putative, expressed |
| Os.4324.1.S1_at | 2.202 | 0.258 | 0.391 | 0.585 | LOC_Os03g03670 | expressed protein |
| Os.28798.1.S1_at | 2.197 | 6.746 | 6.432 | 4.386 | LOC_Os01g58310 | expressed protein |
| Os.10025.1.S1_a_at | 2.195 | 0.667 | 0.506 | 0.631 | LOC_Os12g43130 | Phytoene synthase, chloroplast precursor, putative, expressed |
| Os.18913.1.S1_x_at | 2.195 | 0.621 | 0.555 | 1.032 | LOC_Os05g39800 | Nodulin-like family protein, expressed |
| Os.12025.1.S1_a_at | 2.195 | 0.266 | 0.523 | 0.646 | LOC_Os04g55180 | alpha/beta hydrolase fold PF\|00561 containing protein, putative, expressed |
| Os.11771.1.S1_at | 2.191 | 0.479 | 0.505 | 0.468 | LOC_Os06g12320 | Transmembrane amino acid transporter protein, expressed |
| Os.27805.1.S1_at | 2.185 | 0.742 | 0.899 | 0.979 | LOC_Os03g48000 | magnesium transporter CorA-like family protein, putative, expressed |
| Os.7016.1.S1_at | 2.183 | 1.418 | 0.958 | 0.838 | LOC_Os03g19220 | negatively light-regulated protein, putative, expressed |

| ProbeSetID | *OMTN2* -OE[a] | Stress response[b] | | | TIGR Locus ID[c] | Annotation |
|---|---|---|---|---|---|---|
| | | Drought | Salt | Cold | | |
| Os.5648.1.S1_at | 2.180 | 0.261 | 0.684 | 0.249 | LOC_Os01g01160 | Chaperone protein dnaJ 20, chloroplast precursor, putative, expressed |
| Os.52768.1.S1_at | 2.176 | 1.285 | 0.648 | 1.075 | LOC_Os02g49510 | amino acid transporter family protein, putative, expressed |
| Os.17136.1.S1_at | 2.173 | 49.828 | 19.557 | 1.942 | LOC_Os03g51920 | expressed protein |
| Os.3340.1.S1_a_at | 2.170 | 0.240 | 0.637 | 0.544 | LOC_Os06g24070 | myb-like DNA-binding domain, SHAQKYF class family protein, expressed |
| Os.36901.1.S1_at | 2.167 | 1.886 | 1.475 | 1.056 | LOC_Os01g58150 | expressed protein |
| Os.12261.1.S1_a_at | 2.157 | 1.085 | 0.804 | 1.059 | LOC_Os03g20380 | CIPK-like protein 1, putative, expressed |
| Os.24952.1.S1_at | 2.155 | 2.535 | 0.995 | 1.864 | LOC_Os04g57720 | Two-component response regulator ARR3, putative, expressed |
| Os.38045.1.S1_at | 2.155 | 0.557 | 0.460 | 0.699 | LOC_Os12g08810 | VTC2, putative, expressed |
| Os.7625.1.A1_s_at | 2.150 | 2.824 | 1.676 | 2.238 | LOC_Os06g51360 | LysM domain containing protein, expressed |
| Os.27613.1.A1_at | 2.146 | 0.171 | 0.483 | 0.259 | LOC_Os05g07870 | Triose phosphate/phosphate translocator, non-green plastid, chloroplast precursor, putative, expressed |
| Os.4417.1.S1_at | 2.145 | 0.334 | 0.332 | 0.638 | LOC_Os09g12290 | Bifunctional aspartokinase/homoserine dehydrogenase 2, chloroplast precursor, putative, expressed |
| Os.16966.1.S1_at | 2.142 | 1.030 | 1.070 | 0.832 | LOC_Os11g13850 | Rieske domain containing protein, expressed |
| Os.10524.2.S1_at | 2.141 | 0.124 | 0.431 | 0.426 | LOC_Os12g03070 | FHA domain containing protein, expressed |
| Os.27659.1.S1_x_at | 2.136 | 0.382 | 0.570 | 0.150 | LOC_Os01g08460 | TMS membrane family protein, putative, expressed |
| Os.53004.1.S1_at | 2.132 | 0.868 | 0.253 | 2.011 | LOC_Os12g12560 | NADP-dependent oxidoreductase P2, putative, expressed |
| Os.8785.1.S1_at | 2.129 | 0.813 | 0.697 | 0.467 | LOC_Os03g40100 | ACT domain containing protein, putative, expressed |
| Os.19375.1.S1_at | 2.124 | 2.105 | 1.032 | 0.837 | LOC_Os08g34790 | 4-coumarate-CoA ligase 2, putative, expressed |
| Os.51345.1.S1_at | 2.124 | 1.131 | 2.485 | 2.068 | LOC_Os05g08860 | expressed protein |
| Os.27580.1.S1_at | 2.121 | 0.357 | 0.197 | 0.380 | LOC_Os07g37230 | PAP-specific phosphatase, mitochondrial precursor, putative, expressed |
| Os.28255.1.S1_at | 2.121 | 0.499 | 0.585 | 0.600 | LOC_Os04g54790 | ABC1 family protein, expressed |
| Os.5697.1.S1_at | 2.120 | 2.600 | 4.335 | 2.146 | LOC_Os07g33270 | expressed protein |
| Os.46498.1.S1_at | 2.118 | 0.754 | 1.343 | 0.397 | LOC_Os10g11310 | expressed protein |

| ProbeSetID | *OMTN2* -OE[a] | Stress response[b] | | | TIGR Locus ID[c] | Annotation |
|---|---|---|---|---|---|---|
| | | Drought | Salt | Cold | | |
| Os.7676.1.S1_at | 2.117 | 0.412 | 0.683 | 0.953 | LOC_Os02g49680 | calcium sensing receptor, putative, expressed |
| Os.4801.1.S1_x_at | 2.117 | 0.793 | 0.818 | 0.881 | LOC_Os01g52010 | Allinase, C-terminal domain containing protein, expressed |
| Os.11602.1.S1_at | 2.112 | 0.154 | 0.882 | 1.170 | LOC_Os09g38090 | expressed protein |
| Os.12012.1.S1_at | 2.111 | 5.059 | 2.298 | 3.413 | LOC_Os10g25290 | ZIM motif family protein, expressed |
| OsAffx.24128.1.S1_s_at | 2.106 | 1.540 | 1.003 | 1.059 | LOC_Os02g06330 | AP2 domain containing protein, expressed |
| Os.11331.1.S1_at | 2.106 | 13.367 | 6.247 | 6.773 | LOC_Os08g10500 | expressed protein |
| Os.9585.1.S1_s_at | 2.102 | 3.911 | 1.441 | 1.289 | LOC_Os12g41110 | Calmodulin, putative, expressed |
| OsAffx.22181.1.S1_s_at | 2.102 | 3.956 | 1.588 | 3.195 | LOC_Os01g17050 | VQ motif family protein, expressed |
| Os.8098.1.S1_at | 2.101 | 0.325 | 1.475 | 0.605 | LOC_Os11g18570 | Cytochrome P450 family protein, expressed |
| Os.5661.1.S1_s_at | 2.097 | 2.240 | 4.280 | 0.572 | LOC_Os08g39640 | Cytochrome P450 family protein |
| Os.7347.1.S1_at | 2.096 | 0.380 | 0.564 | 0.798 | LOC_Os03g62160 | expressed protein |
| Os.35463.1.S1_at | 2.088 | 8.504 | 4.835 | 0.815 | LOC_Os05g48810 | DnaJ domain containing protein, expressed |
| Os.9494.1.S1_s_at | 2.087 | 0.473 | 0.795 | 0.754 | LOC_Os04g45000 | oxidoreductase, short chain dehydrogenase/reductase family protein, expressed |
| Os.21228.1.S1_at | 2.085 | 0.534 | 0.674 | 0.698 | LOC_Os04g33060 | NAD dependent epimerase/dehydratase family protein, expressed |
| Os.12639.1.A1_at | 2.084 | 1.186 | 0.884 | 0.757 | LOC_Os11g10710 | Protein kinase domain containing protein, expressed |
| Os.27807.1.S1_at | 2.079 | 7.933 | 4.242 | 6.426 | LOC_Os03g01740 | expressed protein |
| Os.11802.1.S1_at | 2.077 | 1.699 | 1.645 | 1.460 | LOC_Os03g51650 | expressed protein |
| Os.55193.1.S1_at | 2.076 | 111.992 | 19.437 | 1.487 | LOC_Os12g42280 | viviparous-14, putative, expressed |
| Os.2488.1.S1_at | 2.075 | 1.595 | 0.933 | 0.709 | LOC_Os03g12840 | inositol 1, 3, 4-trisphosphate 5/6-kinase, putative, expressed |
| Os.5817.1.S1_at | 2.075 | 17.361 | 10.204 | 0.986 | LOC_Os06g09560 | DnaJ domain containing protein, expressed |
| OsAffx.32170.1.S1_at | 2.068 | 2.386 | 1.087 | 1.204 | LOC_Os12g43450 | P21 protein, putative, expressed |
| Os.48273.1.A1_s_at | 2.067 | 1.022 | 0.769 | 0.728 | LOC_Os01g51530 | expressed protein |
| OsAffx.3569.1.S1_x_at | 2.066 | 2.529 | 2.926 | 0.898 | LOC_Os03g50960 | Protease inhibitor/seed storage/LTP family protein, expressed |
| OsAffx.5568.1.S1_at | 2.066 | 0.914 | 0.771 | 1.686 | LOC_Os07g38630 | hypothetical protein |

| ProbeSetID | *OMTN2* -OE[a] | Stress response[b] | | | TIGR Locus ID[c] | Annotation |
|---|---|---|---|---|---|---|
| | | Drought | Salt | Cold | | |
| Os.51003.1.S1_at | 2.064 | 0.228 | 0.588 | 0.437 | LOC_Os05g42280 | expressed protein |
| Os.50995.1.S1_at | 2.059 | 0.538 | 0.552 | 1.783 | LOC_Os01g50170 | Eukaryotic aspartyl protease family protein, expressed |
| Os.27592.1.A1_at | 2.052 | 0.349 | 0.464 | 0.558 | LOC_Os11g42490 | retrotransposon protein, putative, unclassified, expressed |
| Os.54430.1.S1_at | 2.052 | 0.568 | 0.733 | 0.677 | LOC_Os02g37200 | expressed protein |
| Os.22076.1.S1_a_at | 2.050 | 2.895 | 1.435 | 5.330 | LOC_Os01g62430 | Elicitor-responsive protein 1, putative, expressed |
| Os.50612.1.A1_at | 2.047 | 0.712 | 0.817 | 1.000 | LOC_Os08g08140 | D-mannose binding lectin family protein, expressed |
| Os.7831.1.S1_at | 2.046 | 6.984 | 3.402 | 0.823 | LOC_Os06g11980 | expressed protein |
| Os.51546.1.S1_at | 2.044 | 0.960 | 3.024 | 3.429 | LOC_Os03g08520 | expressed protein |
| Os.11330.1.S1_a_at | 2.043 | 1.474 | 1.248 | 0.973 | LOC_Os07g26690 | aquaporin PIP2.4, putative, expressed |
| Os.33102.1.S1_at | 2.041 | 2.600 | 28.282 | 2.553 | LOC_Os01g43740 | Cytochrome P450 family protein, expressed |
| Os.53178.1.S1_s_at | 2.039 | 1.838 | 0.298 | 0.249 | LOC_Os05g47840 | IPP transferase family protein, expressed |
| Os.7116.1.S1_at | 2.037 | 1.636 | 2.565 | 2.856 | LOC_Os03g53020 | Helix-loop-helix DNA-binding domain containing protein, expressed |
| Os.21280.1.S1_at | 2.030 | 0.745 | 0.583 | 1.109 | LOC_Os12g25490 | O-methyltransferase ZRP4, putative, expressed |
| Os.11946.1.S1_at | 2.026 | 3.066 | 3.438 | 0.212 | LOC_Os06g21380 | carboxyl-terminal protease family protein, expressed |
| Os.767.1.S1_at | 2.025 | 0.744 | 1.503 | 1.205 | LOC_Os01g43700 | Cytochrome P450 family protein, expressed |
| Os.12043.1.S1_at | 2.024 | 2.868 | 6.642 | 0.974 | LOC_Os01g44120 | expressed protein |
| OsAffx.2403.1.S1_at | 2.021 | 0.955 | 0.736 | 2.201 | LOC_Os01g64440 | expressed protein |
| Os.20221.1.S1_at | 2.019 | 4.874 | 2.418 | 3.576 | LOC_Os09g21710 | AN1-like Zinc finger family protein, expressed |
| Os.51595.1.S1_at | 2.017 | 1.916 | 2.158 | 1.395 | LOC_Os01g46370 | lipase class 3 family protein, putative, expressed |
| Os.12550.1.S1_s_at | 2.011 | 1.038 | 2.351 | 0.614 | LOC_Os02g09510 | UDP-glucoronosyl and UDP-glucosyl transferase family protein, expressed |
| Os.51323.1.S1_at | 2.009 | 0.256 | 1.793 | 1.220 | LOC_Os12g14540 | expressed protein |
| OsAffx.30176.1.S1_at | 2.009 | 0.375 | 0.331 | 0.520 | LOC_Os09g37710 | RWP-RK domain containing protein, expressed |
| Os.1479.1.S1_at | 2.006 | 0.356 | 0.414 | 0.639 | LOC_Os07g48980 | Nicotianamine synthase 3, putative, expressed |
| Os.14831.2.S1_at | 2.004 | 0.142 | 0.452 | 1.418 | LOC_Os09g04440 | DNA-binding protein, putative, expressed |

| ProbeSetID | *OMTN2* -OE[a] | Stress response[b] | | | TIGR Locus ID[c] | Annotation |
|---|---|---|---|---|---|---|
| | | Drought | Salt | Cold | | |
| Os.32022.1.S1_at | 2.001 | 13.189 | 2.104 | 2.252 | LOC_Os07g37620 | Cotton fibre expressed protein, expressed |
| Os.2371.1.S1_at | 0.072 | 2.412 | 1.820 | 1.596 | LOC_Os12g26290 | alpha-dioxygenase, putative, expressed |
| Os.36283.1.S1_at | 0.088 | 1.166 | 1.408 | 0.410 | LOC_Os12g25090 | expressed protein |
| OsAffx.7606.1.S1_at | 0.119 | 1.419 | 4.132 | 1.940 | LOC_Os12g15680 | Multicopper oxidase family protein, expressed |
| Os.5349.1.S1_at | 0.120 | 0.791 | 2.229 | 1.812 | LOC_Os11g37700 | ABC transporter, putative, expressed |
| Os.11327.1.S1_at | 0.134 | 3.669 | 2.953 | 1.370 | LOC_Os03g18130 | Asparagine synthetase, putative, expressed |
| Os.27688.1.A1_at | 0.136 | 0.957 | 0.731 | 9.416 | LOC_Os04g21350 | flowering promoting factor-like 1, putative, expressed |
| Os.57456.1.S1_x_at | 0.150 | 2.707 | 2.378 | 1.608 | LOC_Os01g24710 | Salt stress-induced protein, putative, expressed |
| Os.11287.1.S1_at | 0.153 | 3.770 | 2.584 | 1.577 | LOC_Os06g50930 | Senescence-associated protein DIN1, putative, expressed |
| Os.28200.1.S1_x_at | 0.156 | 24.253 | 27.402 | 8.330 | LOC_Os03g61160 | expressed protein |
| Os.27545.1.S1_at | 0.156 | 1.307 | 2.693 | 1.805 | LOC_Os04g38540 | Aldose 1-epimerase family protein, expressed |
| Os.32455.1.S1_at | 0.159 | 1.276 | 0.980 | 1.156 | LOC_Os01g54670 | expressed protein |
| Os.27507.1.S1_at | 0.159 | 0.899 | 3.222 | 1.949 | LOC_Os06g35700 | Reticuline oxidase precursor, putative, expressed |
| Os.49322.1.S1_at | 0.159 | 0.511 | 0.660 | 2.426 | LOC_Os09g15050 | Ent-kaurene synthase A, chloroplast precursor, putative, expressed |
| Os.46840.1.S1_x_at | 0.170 | 0.338 | 0.425 | 0.884 | LOC_Os10g34700 | expressed protein |
| OsAffx.3920.1.S1_s_at | 0.173 | 0.214 | 1.854 | 1.961 | LOC_Os04g27670 | Terpene synthase family, metal binding domain containing protein, expressed |
| Os.33316.1.S1_at | 0.175 | 3.624 | 0.960 | 1.115 | LOC_Os01g34560 | very-long-chain fatty acid condensing enzyme, putative, expressed |
| Os.27420.2.A1_s_at | 0.175 | 0.317 | 0.899 | 1.096 | LOC_Os05g17810 | SHR5-receptor-like kinase, putative |
| Os.12551.1.S1_s_at | 0.176 | 52.177 | 31.401 | 0.940 | LOC_Os05g46480 | Late embryogenesis abundant protein, expressed |
| Os.22594.1.S1_at | 0.177 | 8.122 | 4.913 | 1.160 | LOC_Os01g03390 | Bowman-Birk type bran trypsin inhibitor precursor, putative, expressed |
| OsAffx.15812.1.S1_at | 0.178 | 1.455 | 1.000 | 1.182 | LOC_Os06g38120 | expressed protein |
| Os.19472.1.S1_at | 0.179 | 7.458 | 5.282 | 0.656 | LOC_Os01g03330 | Bowman-Birk type bran trypsin inhibitor precursor, putative, expressed |
| Os.23518.1.A1_at | 0.179 | 0.151 | 0.473 | 0.710 | LOC_Os02g36190 | Cytochrome P450 family protein, expressed |
| Os.39652.1.S1_at | 0.179 | 0.403 | 1.035 | 0.584 | LOC_Os09g37330 | auxin induced protein, putative, expressed |

| ProbeSetID | *OMTN2* -OE[a] | Stress response[b] | | | TIGR Locus ID[c] | Annotation |
|---|---|---|---|---|---|---|
| | | Drought | Salt | Cold | | |
| Os.7281.1.S1_at | 0.183 | 0.433 | 0.831 | 1.292 | LOC_Os01g68730 | expressed protein |
| Os.30909.1.S1_at | 0.187 | 0.504 | 1.065 | 0.187 | LOC_Os01g09190 | expressed protein |
| Os.28301.1.S1_at | 0.188 | 0.225 | 0.520 | 1.377 | LOC_Os07g05360 | Photosystem II 10 kDa polypeptide, chloroplast precursor, putative, expressed |
| Os.27143.1.S1_at | 0.189 | 0.755 | 1.183 | 0.928 | LOC_Os07g48060 | Cationic peroxidase 1 precursor, putative, expressed |
| Os.17918.1.S1_at | 0.193 | 0.172 | 1.654 | 1.158 | LOC_Os03g16950 | 33 kDa secretory protein, putative, expressed |
| Os.20482.1.S1_at | 0.194 | 0.633 | 0.627 | 1.841 | LOC_Os03g02550 | F-box family protein, putative, expressed |
| Os.23008.1.S1_at | 0.196 | 0.746 | 1.288 | 1.907 | LOC_Os10g39710 | Strictosidine synthase family protein, expressed |
| OsAffx.23641.1.S1_at | 0.197 | 0.162 | 0.413 | 3.379 | LOC_Os01g43230 | expressed protein |
| Os.7612.1.S1_at | 0.198 | 1.746 | 1.710 | 2.558 | LOC_Os03g60840 | Bowman-Birk serine protease inhibitor family protein, expressed |
| OsAffx.29832.2.S1_at | 0.204 | 0.615 | 0.214 | 0.957 | LOC_Os09g13440 | expressed protein |
| OsAffx.31963.1.S1_at | 0.204 | 0.329 | 0.553 | 0.824 | LOC_Os12g31540 | expressed protein |
| Os.27019.1.S1_at | 0.205 | 3.466 | 2.619 | 0.967 | LOC_Os04g43410 | Glycosyl hydrolase family 1 protein, expressed |
| OsAffx.24782.1.S1_s_at | 0.207 | 3.917 | 1.965 | 2.304 | LOC_Os02g48320 | DNA-binding protein, putative, expressed |
| Os.52377.1.S1_s_at | 0.207 | 0.357 | 3.907 | 1.712 | LOC_Os02g13370 | expressed protein |
| Os.2821.1.A1_at | 0.208 | 1.653 | 0.305 | 1.017 | LOC_Os02g02210 | aminotransferase, class III family protein, expressed |
| OsAffx.14345.1.S1_at | 0.213 | 0.808 | 0.636 | 1.190 | LOC_Os04g49490 | protein phosphatase 2C family protein, putative, expressed |
| Os.26511.1.S1_at | 0.214 | 2.808 | 2.127 | 5.175 | LOC_Os04g49370 | expressed protein |
| Os.2211.1.S1_at | 0.215 | 0.568 | 0.573 | 1.727 | LOC_Os08g09010 | Germin-like protein subfamily 1 member 11 precursor, putative, expressed |
| Os.15537.1.S1_at | 0.218 | 2.509 | 1.547 | 0.509 | LOC_Os08g39660 | Cytochrome P450 family protein, expressed |
| Os.50825.1.S1_s_at | 0.219 | 1.097 | 0.433 | 0.270 | LOC_Os05g39990 | Alpha-expansin 1 precursor, putative, expressed |
| Os.7125.1.S1_at | 0.221 | 0.315 | 0.808 | 1.280 | LOC_Os09g36730 | Myb-related protein Hv1, putative, expressed |
| Os.2321.1.S1_at | 0.222 | 0.659 | 0.543 | 1.943 | LOC_Os08g09060 | Germin-like protein subfamily 1 member 11 precursor, putative, expressed |
| Os.5335.1.S1_at | 0.226 | 11.598 | 2.485 | 17.068 | LOC_Os04g49450 | myb-like DNA-binding domain, SHAQKYF class family protein, expressed |
| Os.12234.1.S1_s_at | 0.229 | 0.784 | 0.340 | 0.481 | LOC_Os10g40730 | Beta-expansin 1a precursor, putative, expressed |

| ProbeSetID | *OMTN2* -OE[a] | Stress response[b] | | | TIGR Locus ID[c] | Annotation |
|---|---|---|---|---|---|---|
| | | Drought | Salt | Cold | | |
| Os.35433.1.S1_at | 0.233 | 0.402 | 0.165 | 14.371 | LOC_Os04g39320 | expressed protein |
| Os.4453.1.S1_at | 0.234 | 0.276 | 0.891 | 0.682 | LOC_Os06g03520 | expressed protein |
| Os.51757.1.S1_at | 0.234 | 1.405 | 1.042 | 0.813 | LOC_Os06g26270 | expressed protein |
| Os.11786.2.S1_at | 0.234 | 7.064 | 5.694 | 17.799 | LOC_Os01g15900 | Dof domain, zinc finger family protein, expressed |
| Os.27011.1.A1_s_at | 0.235 | 0.551 | 0.802 | 1.551 | LOC_Os01g04550 | serine/threonine protein kinase, putative, expressed |
| Os.56815.1.S1_at | 0.237 | 0.212 | 0.297 | 1.331 | LOC_Os06g18960 | expressed protein |
| OsAffx.2919.1.S1_at | 0.237 | 1.784 | 2.892 | 3.162 | LOC_Os02g39660 | Leucine Rich Repeat family protein, expressed |
| Os.38856.1.A1_s_at | 0.237 | 2.874 | 0.619 | 1.877 | LOC_Os01g67950 | ubiquitin family protein, putative, expressed |
| Os.38309.1.S1_at | 0.238 | 2.361 | 2.725 | 3.040 | LOC_Os06g36390 | expressed protein |
| Os.51657.1.S1_at | 0.240 | 0.203 | 0.581 | 0.700 | LOC_Os08g44830 | zinc finger family protein, putative, expressed |
| Os.43963.1.S1_at | 0.241 | 1.395 | 2.535 | 3.326 | LOC_Os07g33580 | Cytochrome P450 family protein, expressed |
| Os.17758.1.A1_at | 0.243 | 1.171 | 0.857 | 1.000 | LOC_Os08g15710 | expressed protein |
| Os.50572.1.S1_at | 0.243 | 1.092 | 0.941 | 1.701 | LOC_Os12g36920 | calmodulin-binding protein, putative, expressed |
| Os.802.1.S1_at | 0.245 | 0.537 | 0.989 | 0.687 | LOC_Os01g01430 | NAM, putative, expressed |
| Os.12201.2.S1_at | 0.246 | 2.702 | 2.693 | 0.977 | LOC_Os09g27820 | 1-aminocyclopropane-1-carboxylate oxidase 1, putative, expressed |
| OsAffx.11788.1.S1_at | 0.246 | 0.376 | 0.313 | 0.649 | LOC_Os01g68460 | DC1 domain-containing protein, putative, expressed |
| Os.21319.1.S1_at | 0.246 | 3.877 | 4.500 | 4.794 | LOC_Os09g06770 | Zinc finger, C3HC4 type family protein, expressed |
| OsAffx.3768.1.S1_at | 0.246 | 1.667 | 4.583 | 0.917 | LOC_Os04g11780 | resistance protein LR10, putative |
| Os.40049.1.S1_at | 0.246 | 0.713 | 0.489 | 0.628 | LOC_Os06g46160 | expressed protein |
| Os.55740.1.S1_at | 0.246 | 1.588 | 1.306 | 0.541 | LOC_Os06g45960 | Cytochrome P450 family protein, expressed |
| Os.49634.1.S1_x_at | 0.247 | 2.255 | 3.515 | 5.648 | LOC_Os01g55510 | Dynein light chain type 1 family protein, expressed |
| Os.51339.1.S1_at | 0.250 | 2.469 | 0.906 | 4.750 | LOC_Os04g22120 | Protein kinase domain containing protein, expressed |
| Os.12977.1.S1_at | 0.251 | 2.675 | 1.197 | 3.089 | LOC_Os06g19430 | expressed protein |
| Os.17271.1.S1_at | 0.253 | 0.322 | 0.590 | 1.225 | LOC_Os12g07310 | Citrate binding protein precursor, putative, expressed |

| ProbeSetID | *OMTN2* -OE[a] | Stress response[b] | | | TIGR Locus ID[c] | Annotation |
|---|---|---|---|---|---|---|
| | | Drought | Salt | Cold | | |
| Os.22277.1.S1_at | 0.253 | 11.799 | 24.132 | 1.002 | LOC_Os07g34520 | Isocitrate lyase, putative, expressed |
| Os.53561.1.S1_at | 0.257 | 0.493 | 1.189 | 0.899 | LOC_Os07g26110 | plant integral membrane protein TIGR01569 containing protein, expressed |
| OsAffx.8290.1.S1_at | 0.257 | 1.580 | 1.037 | 0.370 | LOC_Os10g17960 | receptor-like protein kinase homolog RK20-1, putative, expressed |
| Os.11252.1.S1_at | 0.258 | 0.453 | 3.110 | 1.455 | LOC_Os02g37160 | heavy metal-associated domain containing protein, expressed |
| Os.6542.1.S1_at | 0.258 | 2.620 | 5.965 | 3.089 | LOC_Os03g08630 | dihydrokaempferol 4-reductase, putative, expressed |
| OsAffx.31316.1.S1_at | 0.258 | 0.308 | 0.159 | 0.224 | LOC_Os11g35850 | Leucine Rich Repeat family protein, expressed |
| Os.19861.1.S1_at | 0.258 | 0.697 | 1.294 | 1.731 | LOC_Os07g03730 | Pathogenesis-related protein 1 precursor, putative, expressed |
| Os.50881.1.S1_x_at | 0.261 | 0.608 | 1.184 | 1.543 | LOC_Os09g39650 | Protein kinase domain containing protein, expressed |
| OsAffx.10980.1.S1_at | 0.261 | 1.235 | 1.330 | 3.191 | LOC_Os01g09150 | hypothetical protein |
| Os.15943.1.S1_at | 0.264 | 0.533 | 0.961 | 1.654 | LOC_Os01g67310 | Patatin-like phospholipase family protein, expressed |
| Os.5941.1.S1_at | 0.264 | 0.745 | 1.641 | 1.629 | LOC_Os01g53040 | WRKY DNA binding domain containing protein, expressed |
| Os.30572.1.S1_at | 0.265 | 1.216 | 0.811 | 0.634 | LOC_Os01g50610 | SAM dependent carboxyl methyltransferase family protein, expressed |
| Os.5031.1.S1_at | 0.268 | 0.382 | 1.135 | 1.325 | LOC_Os12g36830 | pathogenesis-related protein 10, putative, expressed |
| Os.51601.1.S1_at | 0.269 | 0.185 | 0.595 | 1.823 | LOC_Os07g40300 | Zinc finger protein 7, putative, expressed |
| Os.55343.1.S1_at | 0.271 | 3.947 | 4.715 | 0.896 | LOC_Os04g56030 | expressed protein |
| Os.23405.1.S1_at | 0.272 | 5.964 | 3.302 | 1.516 | LOC_Os07g14150 | Guanine deaminase, putative, expressed |
| Os.25952.1.S1_at | 0.273 | 6.165 | 3.003 | 10.106 | LOC_Os05g27780 | expressed protein |
| Os.24580.2.A1_at | 0.273 | 0.894 | 0.850 | 2.324 | LOC_Os02g05950 | Leucine Rich Repeat family protein, expressed |
| Os.33131.1.A1_at | 0.274 | 2.056 | 1.751 | 0.540 | LOC_Os03g20550 | WRKY DNA binding containing protein, expressed |
| Os.30063.1.S1_at | 0.274 | 1.558 | 2.404 | 1.131 | LOC_Os05g45450 | nuclear protein, putative, expressed |
| Os.5905.1.S1_at | 0.275 | 0.897 | 0.223 | 0.877 | LOC_Os06g32990 | Peroxidase family protein, expressed |
| Os.52228.1.S1_at | 0.275 | 0.224 | 0.400 | 0.903 | LOC_Os11g36000 | Leucine Rich Repeat family protein, expressed |
| Os.32357.1.S1_at | 0.276 | 5.540 | 6.431 | 1.308 | LOC_Os07g46950 | transposon protein, putative, unclassified, expressed |
| Os.5812.1.S1_at | 0.276 | 0.795 | 0.938 | 2.129 | LOC_Os03g20500 | F-box domain containing protein, expressed |

| ProbeSetID | *OMTN2* -OE[a] | Stress response[b] | | | TIGR Locus ID[c] | Annotation |
|---|---|---|---|---|---|---|
| | | Drought | Salt | Cold | | |
| Os.53763.1.A1_at | 0.276 | 2.714 | 0.229 | 0.314 | LOC_Os06g38340 | Leucine Rich Repeat family protein, expressed |
| Os.55511.1.S1_at | 0.276 | 0.544 | 2.694 | 0.549 | LOC_Os04g45520 | Integral membrane protein, expressed |
| OsAffx.31475.1.S1_at | 0.279 | 0.927 | 1.232 | 1.277 | LOC_Os11g44630 | hypothetical protein |
| Os.28124.1.S1_at | 0.279 | 0.357 | 0.634 | 2.404 | LOC_Os10g28080 | Glycosyl hydrolases family 18 protein, expressed |
| OsAffx.12733.1.S1_at | 0.280 | 1.472 | 1.239 | 3.033 | LOC_Os03g04740 | expressed protein |
| Os.27494.1.S1_at | 0.280 | 0.474 | 0.665 | 1.051 | LOC_Os07g41200 | expressed protein |
| Os.19374.1.S1_at | 0.281 | 1.579 | 1.035 | 3.561 | LOC_Os01g48440 | Glycosyltransferase family 43 protein, expressed |
| OsAffx.17348.1.S1_at | 0.281 | 0.699 | 0.928 | 2.325 | LOC_Os08g35590 | hypothetical protein |
| OsAffx.30833.1.S1_at | 0.281 | 0.356 | 0.406 | 0.979 | LOC_Os11g05800 | TB2/DP1, HVA22 family protein, expressed |
| Os.6042.1.S1_at | 0.281 | 4.173 | 1.419 | 0.716 | LOC_Os07g25810 | transposon protein, putative, CACTA, En/Spm sub-class, expressed |
| Os.50399.1.S1_at | 0.282 | 74.437 | 31.901 | 1.586 | LOC_Os06g05420 | expressed protein |
| Os.10675.1.A1_at | 0.284 | 0.734 | 1.709 | 1.969 | LOC_Os01g68140 | expressed protein |
| OsAffx.14697.1.S1_at | 0.285 | 8.414 | 6.759 | 1.931 | LOC_Os05g14820 | lysine and histidine specific transporter, putative, expressed |
| OsAffx.14201.1.S1_at | 0.286 | 0.337 | 0.136 | 0.472 | LOC_Os04g39360 | heavy metal-associated domain containing protein, expressed |
| OsAffx.19285.1.S1_at | 0.286 | 0.808 | 2.512 | 1.146 | LOC_Os11g40810 | Leucine Rich Repeat family protein, expressed |
| Os.46618.1.S1_at | 0.288 | 29.232 | 12.826 | 6.346 | LOC_Os10g41550 | Glycosyl hydrolase family 14 protein, expressed |
| Os.11711.1.S1_at | 0.289 | 0.693 | 0.757 | 1.444 | LOC_Os12g17880 | U-box domain containing protein, expressed |
| Os.47445.1.S1_at | 0.291 | 1.503 | 1.340 | 1.357 | LOC_Os02g26210 | flowering promoting factor-like 1, putative, expressed |
| Os.2320.1.S1_at | 0.292 | 0.629 | 0.602 | 1.514 | LOC_Os08g09080 | Germin-like protein subfamily 1 member 11 precursor, putative, expressed |
| Os.6009.3.S1_a_at | 0.293 | 0.870 | 1.054 | 1.050 | LOC_Os03g08470 | AP2 domain containing protein, expressed |
| OsAffx.26389.1.S1_at | 0.298 | 0.619 | 0.619 | 0.952 | LOC_Os04g39300 | heavy metal-associated domain containing protein, expressed |
| Os.51369.1.S1_at | 0.299 | 1.129 | 1.364 | 0.787 | LOC_Os02g51740 | VQ motif family protein, expressed |
| Os.7727.1.S1_at | 0.299 | 4.679 | 3.170 | 0.994 | LOC_Os05g11910 | GDSL-like Lipase/Acylhydrolase family protein, expressed |
| Os.22731.1.S1_at | 0.299 | 9.240 | 14.323 | 0.888 | LOC_Os03g16020 | 17.4 kDa class I heat shock protein, putative, expressed |

| ProbeSetID | *OMTN2* -OE[a] | Stress response[b] | | | TIGR Locus ID[c] | Annotation |
|---|---|---|---|---|---|---|
| | | Drought | Salt | Cold | | |
| OsAffx.7414.1.S1_at | 0.300 | 0.129 | 0.259 | 0.345 | LOC_Os11g44700 | expressed protein |
| OsAffx.24063.1.S1_at | 0.300 | 0.917 | 4.792 | 0.958 | LOC_Os02g02230 | Cytochrome P450 51, putative, expressed |
| Os.6345.1.S1_at | 0.301 | 0.701 | 0.583 | 1.223 | LOC_Os06g36560 | Inositol oxygenase, putative, expressed |
| OsAffx.30966.4.S1_at | 0.302 | 2.529 | 2.118 | 3.353 | LOC_Os07g46060 | expressed protein |
| Os.7938.1.S1_at | 0.303 | 3.543 | 1.221 | 0.757 | LOC_Os04g59190 | Peroxidase family protein, expressed |
| Os.51904.1.S1_at | 0.304 | 0.674 | 1.289 | 0.622 | LOC_Os05g13970 | Phosphorylase family protein, expressed |
| OsAffx.30765.1.S1_at | 0.304 | 0.544 | 0.888 | 0.878 | LOC_Os11g01480 | myb-like DNA-binding domain, SHAQKYF class family protein, expressed |
| OsAffx.19473.1.S1_at | 0.305 | 1.539 | 1.309 | 1.893 | LOC_Os12g03740 | F-box domain containing protein, expressed |
| Os.50152.1.S1_at | 0.306 | 0.955 | 1.468 | 2.766 | LOC_Os03g59770 | EF hand family protein, expressed |
| Os.40030.1.S1_s_at | 0.307 | 0.293 | 0.743 | 0.678 | LOC_Os05g16420 | SHR5-receptor-like kinase, putative, expressed |
| Os.11120.1.S1_at | 0.308 | 0.558 | 0.758 | 1.483 | LOC_Os03g08460 | AP2 domain containing protein, expressed |
| Os.26687.1.S1_at | 0.309 | 8.789 | 7.301 | 1.073 | LOC_Os04g40990 | Malate synthase, glyoxysomal, putative, expressed |
| Os.12633.1.S1_at | 0.310 | 256.935 | 143.57 7 | 1.561 | LOC_Os11g26790 | Dehydrin family protein, expressed |
| Os.7339.1.S1_at | 0.311 | 1.839 | 1.648 | 1.933 | LOC_Os10g31850 | CHY zinc finger family protein, expressed |
| OsAffx.5948.1.S1_at | 0.312 | 0.981 | 0.858 | 1.317 | LOC_Os08g27170 | calmodulin-binding protein, putative |
| Os.56048.1.S1_s_at | 0.312 | 0.246 | 0.507 | 1.014 | LOC_Os01g68740 | expressed protein |
| Os.23327.2.S1_a_at | 0.313 | 0.291 | 0.602 | 2.617 | LOC_Os10g10130 | Calcium binding EGF domain containing protein, expressed |
| Os.51787.1.S1_at | 0.316 | 0.435 | 0.444 | 1.350 | LOC_Os12g24320 | Cell Division Protein AAA ATPase family, putative, expressed |
| Os.21349.1.S1_at | 0.316 | 2.482 | 1.820 | 0.909 | LOC_Os03g04020 | Rare lipoprotein A like double-psi beta-barrel containing protein, expressed |
| Os.37773.1.S1_at | 0.317 | 1.643 | 7.595 | 2.434 | LOC_Os03g16030 | 17.4 kDa class I heat shock protein, putative, expressed |
| Os.16036.1.S1_at | 0.317 | 1.069 | 1.249 | 0.867 | LOC_Os11g09180 | expressed protein |
| OsAffx.17220.1.S1_at | 0.318 | 3.300 | 3.450 | 0.550 | LOC_Os08g29040 | Protein kinase domain containing protein |
| Os.50246.1.S1_at | 0.319 | 0.549 | 0.358 | 9.954 | LOC_Os02g49880 | CCT motif family protein, expressed |

| ProbeSetID | *OMTN2* -OE[a] | Stress response[b] | | | TIGR Locus ID[c] | Annotation |
|---|---|---|---|---|---|---|
| | | Drought | Salt | Cold | | |
| Os.20067.1.S1_at | 0.320 | 0.728 | 0.869 | 0.998 | LOC_Os04g38530 | Aldose 1-epimerase family protein, expressed |
| Os.51616.1.S1_at | 0.320 | 0.629 | 0.748 | 0.841 | LOC_Os03g16600 | expressed protein |
| Os.51246.1.S1_at | 0.321 | 0.193 | 0.193 | 1.209 | LOC_Os11g11780 | serine/threonine protein kinase, putative, expressed |
| Os.55630.1.S1_at | 0.321 | 0.864 | 1.163 | 1.767 | LOC_Os06g06470 | U-box domain containing protein, expressed |
| Os.6325.1.A1_x_at | 0.321 | 0.292 | 0.747 | 0.970 | LOC_Os01g39770 | Calcineurin B-like protein 10, putative, expressed |
| Os.18717.2.S1_at | 0.322 | 1.557 | 3.307 | 0.634 | LOC_Os09g28180 | D-mannose binding lectin family protein, expressed |
| Os.11322.1.S1_at | 0.323 | 1.168 | 0.685 | 1.311 | LOC_Os04g50950 | POT family protein, expressed |
| Os.48074.1.A1_at | 0.325 | 0.390 | 0.414 | 1.008 | LOC_Os05g19910 | Transferase family protein, expressed |
| Os.10411.1.S1_at | 0.325 | 12.553 | 12.171 | 3.888 | LOC_Os01g62190 | Zinc finger, C2H2 type family protein, expressed |
| Os.26810.1.A1_s_at | 0.326 | 0.333 | 0.619 | 1.059 | LOC_Os02g16940 | Subtilisin N-terminal Region family protein, expressed |
| OsAffx.29831.1.S1_s_at | 0.327 | 0.790 | 0.242 | 1.669 | LOC_Os09g13400 | expressed protein |
| Os.10635.1.S1_s_at | 0.327 | 0.833 | 1.438 | 1.710 | LOC_Os04g51880 | GHMP kinases putative ATP-binding protein, expressed |
| Os.2081.1.S1_at | 0.327 | 1.129 | 0.870 | 1.362 | LOC_Os01g19260 | ATPase, AAA family protein, expressed |
| Os.35343.1.A1_at | 0.328 | 1.812 | 5.787 | 4.001 | LOC_Os11g03370 | NAC-domain containing protein 21/22, putative, expressed |
| Os.54736.1.S1_at | 0.328 | 2.424 | 2.051 | 3.822 | LOC_Os02g52910 | hydroxyproline-rich glycoprotein family protein, putative, expressed |
| Os.14076.1.S1_s_at | 0.328 | 2.119 | 1.534 | 0.916 | LOC_Os02g44820 | expressed protein |
| Os.18742.1.A1_at | 0.329 | 0.304 | 0.424 | 0.736 | LOC_Os05g30010 | expressed protein |
| OsAffx.6270.1.S1_at | 0.329 | 0.318 | 0.435 | 0.368 | LOC_Os09g14450 | NB-ARC domain containing protein, expressed |
| Os.55829.1.S1_at | 0.330 | 3.196 | 1.379 | 2.648 | LOC_Os05g30760 | hydrolase, alpha/beta fold family protein, expressed |
| Os.10245.1.S1_at | 0.330 | 1.135 | 2.508 | 1.681 | LOC_Os04g37700 | expressed protein |
| Os.50346.1.S1_at | 0.331 | 3.223 | 1.628 | 1.876 | LOC_Os05g04680 | expressed protein |
| Os.52150.1.S1_x_at | 0.333 | 3.175 | 3.493 | 9.809 | LOC_Os04g45970 | Glutamate dehydrogenase, putative, expressed |
| Os.12452.1.S1_s_at | 0.333 | 7.189 | 4.123 | 1.011 | LOC_Os08g36910 | Alpha-amylase isozyme 3D precursor, putative, expressed |
| Os.27506.1.A1_at | 0.335 | 0.168 | 0.863 | 0.273 | LOC_Os11g15340 | Jasmonate O-methyltransferase, putative, expressed |

| ProbeSetID | *OMTN2* -OE[a] | Stress response[b] | | | TIGR Locus ID[c] | Annotation |
|---|---|---|---|---|---|---|
| | | Drought | Salt | Cold | | |
| OsAffx.12326.1.S1_at | 0.336 | 0.295 | 1.691 | 0.623 | LOC_Os02g33060 | expressed protein |
| Os.46881.1.S1_at | 0.337 | 1.726 | 0.637 | 0.500 | LOC_Os10g39770 | Zinc finger, C3HC4 type family protein, expressed |
| Os.27330.1.A1_at | 0.337 | 1.318 | 1.713 | 0.677 | LOC_Os04g49160 | Zinc finger, C3HC4 type family protein, expressed |
| Os.7699.1.S1_at | 0.338 | 3.428 | 2.658 | 1.919 | LOC_Os10g22050 | expressed protein |
| Os.27290.1.A1_at | 0.338 | 2.278 | 4.622 | 1.090 | LOC_Os06g07600 | alkaline alpha galactosidase 2, putative, expressed |
| Os.10839.1.S1_at | 0.340 | 7.774 | 3.990 | 2.181 | LOC_Os03g16350 | DNA-binding protein, putative, expressed |
| Os.49501.1.A1_at | 0.340 | 2.798 | 2.460 | 2.123 | LOC_Os06g12230 | TCP family transcription factor containing protein, expressed |
| Os.51127.1.S1_a_at | 0.340 | 0.282 | 0.686 | 0.568 | LOC_Os04g37820 | Cytokinin-O-glucosyltransferase 2, putative, expressed |
| Os.37717.1.A1_s_at | 0.341 | 5.040 | 3.255 | 1.736 | LOC_Os05g15770 | Xylanase inhibitor protein 2 precursor, putative, expressed |
| Os.10174.1.S1_at | 0.341 | 0.246 | 0.315 | 0.733 | LOC_Os11g47460 | myb family transcription factor, putative, expressed |
| Os.50349.2.S1_at | 0.342 | 2.187 | 2.284 | 0.887 | LOC_Os04g09570 | expressed protein |
| Os.52661.1.S1_at | 0.343 | 1.003 | 0.490 | 3.697 | LOC_Os11g44680 | expressed protein |
| Os.16218.1.S1_at | 0.344 | 13.061 | 9.137 | 0.876 | LOC_Os06g30370 | MOTHER of FT and TF1 protein, putative, expressed |
| Os.53009.1.A1_x_at | 0.344 | 0.078 | 0.444 | 0.883 | LOC_Os02g54640 | Receptor family ligand binding region containing protein, expressed |
| Os.28515.1.S1_at | 0.345 | 0.474 | 0.438 | 0.632 | LOC_Os03g39690 | Cytochrome P450 family protein, expressed |
| Os.46941.1.S1_s_at | 0.347 | 2.784 | 3.364 | 1.773 | LOC_Os06g48500 | expressed protein |
| OsAffx.2268.1.S1_at | 0.347 | 0.338 | 0.916 | 0.297 | LOC_Os01g47820 | S-locus-like receptor protein kinase, putative, expressed |
| Os.55085.1.S1_at | 0.347 | 0.322 | 1.373 | 2.271 | LOC_Os09g01580 | hypothetical protein |
| Os.18395.1.S1_s_at | 0.348 | 1.088 | 0.581 | 0.567 | LOC_Os06g15620 | GAST1 protein precursor, putative, expressed |
| OsAffx.5776.1.S1_at | 0.349 | 0.643 | 0.315 | 0.153 | LOC_Os08g10290 | SHR5-receptor-like kinase, putative, expressed |
| Os.23578.1.S1_at | 0.350 | 4.510 | 5.158 | 1.632 | LOC_Os06g05450 | expressed protein |
| Os.27767.1.A1_s_at | 0.350 | 0.107 | 0.663 | 0.826 | LOC_Os04g29680 | Calcium binding EGF domain containing protein, expressed |
| Os.26059.1.S1_at | 0.350 | 0.637 | 0.912 | 1.557 | LOC_Os03g20730 | DnaJ domain containing protein, expressed |
| OsAffx.17691.1.S1_at | 0.350 | 0.237 | 0.547 | 0.864 | LOC_Os09g14490 | NB-ARC domain containing protein, expressed |

| ProbeSetID | *OMTN2* -OE[a] | Stress response[b] | | | TIGR Locus ID[c] | Annotation |
|---|---|---|---|---|---|---|
| | | Drought | Salt | Cold | | |
| OsAffx.13993.1.S1_at | 0.351 | 1.238 | 1.103 | 1.270 | LOC_Os04g27100 | WRKY transcription factor 19, putative |
| Os.39576.1.A1_s_at | 0.353 | 0.891 | 1.032 | 1.198 | LOC_Os03g02240 | expressed protein |
| Os.52699.1.S1_at | 0.353 | 0.928 | 0.737 | 1.267 | LOC_Os04g55420 | Leucine Rich Repeat family protein, expressed |
| Os.19401.1.S1_at | 0.353 | 3.329 | 2.897 | 2.103 | LOC_Os03g04100 | expressed protein |
| Os.17273.1.S1_at | 0.353 | 1.177 | 1.829 | 0.761 | LOC_Os05g27010 | POT family protein, expressed |
| Os.46566.1.S1_at | 0.354 | 2.732 | 4.268 | 0.687 | LOC_Os10g17940 | F-box domain containing protein |
| Os.16453.1.S1_at | 0.355 | 12.735 | 26.621 | 2.599 | LOC_Os12g39400 | Zinc finger, C2H2 type family protein, expressed |
| Os.10379.1.S1_at | 0.357 | 2.792 | 2.111 | 0.841 | LOC_Os02g49860 | ABA induced plasma membrane protein PM 19, putative |
| OsAffx.12018.1.S1_at | 0.357 | 0.789 | 0.582 | 1.084 | LOC_Os02g13460 | Leucine Rich Repeat family protein |
| Os.48695.1.S1_x_at | 0.358 | 0.412 | 0.543 | 0.765 | LOC_Os07g35390 | Protein kinase domain containing protein, expressed |
| Os.8741.1.S1_at | 0.358 | 20.865 | 8.908 | 2.680 | LOC_Os03g12820 | Poly polymerase catalytic domain containing protein, expressed |
| Os.26517.1.S1_at | 0.358 | 28.947 | 20.369 | 4.124 | LOC_Os02g44990 | F-box domain containing protein, expressed |
| Os.15501.1.S1_at | 0.359 | 1.036 | 0.953 | 1.911 | LOC_Os05g41310 | NB-ARC domain containing protein, expressed |
| Os.27810.1.S1_at | 0.360 | 2.580 | 1.165 | 1.061 | LOC_Os07g06850 | PrMC3, putative, expressed |
| Os.17112.1.S1_at | 0.360 | 15.064 | 12.497 | 1.767 | LOC_Os03g18030 | oxidoreductase, 2OG-Fe oxygenase family protein, expressed |
| Os.6149.1.S1_at | 0.361 | 2.271 | 2.626 | 0.516 | LOC_Os01g04580 | Protein kinase domain containing protein, expressed |
| Os.5238.1.S1_at | 0.361 | 0.483 | 0.520 | 1.160 | LOC_Os12g41710 | receptor-like serine/threonine kinase, putative, expressed |
| Os.53357.1.S1_at | 0.363 | 1.191 | 1.579 | 1.355 | LOC_Os02g34940 | Protein disulfide-isomerase precursor, putative, expressed |
| OsAffx.15455.1.S1_at | 0.364 | 0.387 | 0.219 | 1.190 | LOC_Os06g16040 | hypothetical protein |
| Os.158.1.S1_at | 0.365 | 0.295 | 0.632 | 0.850 | LOC_Os10g02070 | Peroxidase N precursor, putative, expressed |
| Os.14951.1.S1_at | 0.365 | 8.699 | 10.099 | 2.665 | LOC_Os08g39730 | Cytochrome P450 family protein, expressed |
| Os.21932.1.S1_at | 0.367 | 3.514 | 3.361 | 0.843 | LOC_Os10g42130 | no apical meristem, putative, expressed |
| Os.37213.1.S1_at | 0.367 | 0.469 | 0.502 | 1.640 | LOC_Os07g29310 | Auxin responsive protein, expressed |
| OsAffx.17899.1.S1_at | 0.369 | 0.870 | 1.141 | 1.167 | LOC_Os09g25930 | Ligand-gated ion channel family protein |

| ProbeSetID | *OMTN2* -OE[a] | Stress response[b] | | | TIGR Locus ID[c] | Annotation |
|---|---|---|---|---|---|---|
| | | Drought | Salt | Cold | | |
| Os.20230.1.S1_at | 0.369 | 5.830 | 4.452 | 1.517 | LOC_Os11g37950 | Barwin, putative, expressed |
| Os.12664.1.S1_at | 0.370 | 0.531 | 0.499 | 0.874 | LOC_Os07g35810 | Protein kinase domain containing protein, expressed |
| Os.12501.1.S1_at | 0.370 | 0.868 | 4.215 | 0.585 | LOC_Os01g55940 | indole-3-acetic acid-amido synthetase GH3.1, putative, expressed |
| Os.32141.1.S1_at | 0.370 | 1.144 | 0.862 | 1.241 | LOC_Os03g49610 | Glycosyl hydrolase family 1 protein, expressed |
| Os.22910.1.S1_at | 0.371 | 0.713 | 0.743 | 0.631 | LOC_Os04g39880 | Non-cyanogenic beta-glucosidase precursor, putative, expressed |
| Os.17014.1.S1_at | 0.371 | 1.126 | 1.888 | 2.135 | LOC_Os04g53850 | NAD dependent epimerase/dehydratase family protein, expressed |
| Os.18448.1.S1_at | 0.372 | 0.786 | 1.347 | 1.115 | LOC_Os12g01490 | myb-like DNA-binding domain, SHAQKYF class family protein, expressed |
| OsAffx.18423.1.S1_at | 0.373 | 0.527 | 0.872 | 1.115 | LOC_Os10g27430 | expressed protein |
| OsAffx.27946.1.S1_at | 0.373 | 0.258 | 0.339 | 1.036 | LOC_Os06g35060 | heavy metal-associated domain containing protein, expressed |
| Os.4971.1.S1_at | 0.373 | 0.481 | 0.616 | 0.820 | LOC_Os02g10500 | hypothetical protein |
| Os.32577.1.S1_at | 0.374 | 0.404 | 0.343 | 1.071 | LOC_Os01g05870 | Leucine Rich Repeat family protein, expressed |
| OsAffx.27662.1.S1_at | 0.375 | 0.303 | 0.711 | 0.807 | LOC_Os06g16030 | Amidase, putative |
| Os.24878.1.A1_at | 0.375 | 0.738 | 1.013 | 1.470 | LOC_Os01g41750 | Leucine Rich Repeat family protein, expressed |
| Os.7629.1.S1_at | 0.375 | 6.527 | 2.751 | 2.285 | LOC_Os09g30160 | Zinc finger, C3HC4 type family protein, expressed |
| Os.31171.1.S1_at | 0.376 | 0.538 | 0.992 | 1.506 | LOC_Os01g40290 | expressed protein |
| Os.9543.1.S1_at | 0.376 | 0.926 | 0.995 | 0.945 | LOC_Os08g26350 | expressed protein |
| Os.54851.1.S1_at | 0.379 | 1.553 | 1.335 | 0.456 | LOC_Os05g46050 | F-box domain containing protein, expressed |
| OsAffx.20014.1.S1_at | 0.379 | 0.439 | 0.815 | 0.739 | LOC_Os12g38770 | diphosphonucleotide phosphatase 1, putative, expressed |
| Os.15516.1.S1_at | 0.380 | 0.963 | 0.821 | 1.189 | LOC_Os04g30240 | Protein kinase domain containing protein, expressed |
| OsAffx.29016.1.S1_at | 0.380 | 1.325 | 1.152 | 1.424 | LOC_Os08g03600 | magnesium transporter CorA, putative, expressed |
| Os.26907.1.S1_at | 0.381 | 0.188 | 0.306 | 0.836 | LOC_Os02g17710 | Leucine Rich Repeat family protein, expressed |
| Os.8263.1.S1_s_at | 0.381 | 1.629 | 1.100 | 0.846 | LOC_Os04g32030 | heavy metal-associated domain containing protein, expressed |
| Os.40417.1.A1_at | 0.381 | 0.550 | 0.322 | 1.073 | LOC_Os07g14740 | Harpin-induced protein 1 containing protein, expressed |
| OsAffx.17158.1.S1_at | 0.384 | 0.459 | 0.293 | 0.122 | LOC_Os08g25850 | expressed protein |

| ProbeSetID | *OMTN2* -OE[a] | Stress response[b] | | | TIGR Locus ID[c] | Annotation |
|---|---|---|---|---|---|---|
| | | Drought | Salt | Cold | | |
| Os.19350.1.S1_at | 0.385 | 0.283 | 0.539 | 1.356 | LOC_Os01g68720 | expressed protein |
| Os.34496.1.S1_at | 0.386 | 8.392 | 3.355 | 0.979 | LOC_Os03g61500 | PGP224, putative, expressed |
| Os.15092.1.S1_at | 0.387 | 2.942 | 2.627 | 1.499 | LOC_Os09g31130 | sodium/dicarboxylate cotransporter, putative, expressed |
| OsAffx.15137.1.S1_at | 0.387 | 6.618 | 10.735 | 2.117 | LOC_Os05g47650 | DNA-binding protein RAV1, putative, expressed |
| Os.15701.1.S1_x_at | 0.387 | 0.893 | 1.201 | 1.459 | LOC_Os07g47350 | Potassium transporter 7, putative, expressed |
| Os.1443.1.S1_a_at | 0.387 | 2.977 | 4.172 | 1.712 | LOC_Os01g06640 | Helix-loop-helix DNA-binding domain containing protein, expressed |
| Os.8202.1.S1_at | 0.387 | 2.181 | 1.704 | 1.680 | LOC_Os12g37560 | phospholipase C, putative, expressed |
| Os.54453.1.S1_at | 0.388 | 0.375 | 0.219 | 1.231 | LOC_Os08g44220 | expressed protein |
| Os.12363.1.S1_at | 0.389 | 0.389 | 0.495 | 1.859 | LOC_Os04g58200 | Protochlorophyllide reductase A, chloroplast precursor, putative, expressed |
| Os.3400.1.S1_s_at | 0.389 | 1.614 | 1.312 | 2.535 | LOC_Os02g49230 | CCT motif family protein, expressed |
| OsAffx.31524.1.S1_at | 0.390 | 1.130 | 2.474 | 1.389 | LOC_Os11g47180 | Leucine Rich Repeat family protein, expressed |
| Os.47706.2.A1_at | 0.390 | 0.755 | 0.934 | 1.635 | LOC_Os10g03570 | NB-ARC domain containing protein |
| Os.52993.1.S1_at | 0.391 | 1.339 | 2.735 | 0.481 | LOC_Os08g02180 | expressed protein |
| Os.49582.1.S1_at | 0.391 | 0.485 | 1.209 | 0.914 | LOC_Os02g45930 | expressed protein |
| Os.18819.1.S1_at | 0.393 | 0.555 | 0.436 | 1.149 | LOC_Os05g06660 | Serine carboxypeptidase II precursor, putative, expressed |
| Os.43417.1.S1_at | 0.393 | 29.609 | 13.435 | 4.391 | LOC_Os01g12760 | Cytochrome P450 family protein, expressed |
| Os.171.1.S1_at | 0.394 | 0.797 | 1.090 | 1.302 | LOC_Os01g47070 | Acidic endochitinase precursor, putative, expressed |
| Os.15607.1.S1_at | 0.395 | 1.042 | 2.231 | 2.316 | LOC_Os04g31120 | beta scruin, putative, expressed |
| Os.11418.1.S1_at | 0.395 | 0.630 | 0.671 | 1.049 | LOC_Os12g34350 | expressed protein |
| OsAffx.31250.1.S1_at | 0.397 | 0.953 | 0.868 | 0.791 | LOC_Os11g31660 | expressed protein |
| Os.15426.1.S1_at | 0.397 | 0.510 | 0.440 | 1.109 | LOC_Os07g35370 | Protein kinase domain containing protein, expressed |
| OsAffx.17563.1.S1_at | 0.398 | 0.241 | 0.725 | 0.128 | LOC_Os09g04430 | hypothetical protein |
| OsAffx.18129.1.S1_at | 0.399 | 1.107 | 0.821 | 1.643 | LOC_Os10g04490 | hypothetical protein |
| Os.25128.1.S1_at | 0.399 | 11.003 | 9.193 | 3.137 | LOC_Os02g09810 | amino acid transporter family protein, putative, expressed |

| ProbeSetID | *OMTN2* -OE[a] | Stress response[b] | | | TIGR Locus ID[c] | Annotation |
|---|---|---|---|---|---|---|
| | | Drought | Salt | Cold | | |
| Os.10293.1.S1_at | 0.400 | 1.343 | 1.369 | 1.269 | LOC_Os07g42510 | AP2 domain containing protein, expressed |
| Os.11552.1.S2_at | 0.400 | 1.042 | 1.049 | 0.653 | LOC_Os04g59150 | Peroxidase 12 precursor, putative, expressed |
| OsAffx.15825.1.S1_at | 0.400 | 0.644 | 1.184 | 2.080 | LOC_Os06g38830 | Leucine Rich Repeat family protein, expressed |
| Os.55490.1.S1_at | 0.401 | 1.573 | 3.203 | 1.506 | LOC_Os06g10000 | expressed protein |
| Os.57007.1.S1_at | 0.401 | 0.061 | 0.488 | 0.219 | LOC_Os04g55440 | Leucine Rich Repeat family protein, expressed |
| Os.57091.1.S1_at | 0.402 | 0.837 | 0.568 | 0.963 | LOC_Os08g01950 | HCBT-like putative defense response protein, putative, expressed |
| Os.51241.1.S1_at | 0.404 | 1.620 | 1.082 | 0.788 | LOC_Os11g35310 | Phytosulfokine precursor protein containing protein |
| Os.53456.1.S1_at | 0.404 | 2.455 | 3.130 | 0.786 | LOC_Os01g32120 | EF hand family protein, expressed |
| Os.16435.1.S1_at | 0.404 | 3.766 | 10.863 | 5.077 | LOC_Os02g53410 | expressed protein |
| Os.50121.1.S1_at | 0.404 | 0.653 | 0.874 | 0.723 | LOC_Os06g41100 | bZIP transcription factor family protein, expressed |
| OsAffx.3006.1.S1_at | 0.404 | 1.024 | 1.121 | 2.205 | LOC_Os02g49240 | expressed protein |
| Os.54467.1.S1_at | 0.406 | 4.638 | 3.268 | 2.110 | LOC_Os03g21710 | WRKY DNA binding domain containing protein |
| Os.27059.1.S1_at | 0.406 | 1.113 | 0.941 | 1.091 | LOC_Os08g10070 | Protein kinase domain containing protein, expressed |
| Os.9700.1.S1_at | 0.406 | 1.165 | 0.836 | 0.872 | LOC_Os08g28970 | expressed protein |
| Os.46777.1.S1_at | 0.408 | 0.421 | 0.363 | 0.888 | LOC_Os04g29580 | Calcium binding EGF domain containing protein, expressed |
| Os.49337.1.S1_at | 0.408 | 0.380 | 0.628 | 0.798 | LOC_Os02g39140 | bHLH transcription factor GBOF-1, putative, expressed |
| OsAffx.26367.1.S1_at | 0.408 | 0.683 | 1.331 | 0.971 | LOC_Os04g37760 | expressed protein |
| Os.9336.1.S1_at | 0.408 | 0.825 | 1.634 | 0.617 | LOC_Os08g43550 | Myb-related protein Zm38, putative, expressed |
| OsAffx.16444.1.S1_at | 0.409 | 1.426 | 1.771 | 0.438 | LOC_Os07g27670 | WRKY DNA binding domain containing protein |
| Os.9086.1.S1_at | 0.410 | 10.092 | 5.127 | 1.148 | LOC_Os03g10210 | Homeobox domain containing protein, expressed |
| OsAffx.4329.1.S1_at | 0.410 | 2.508 | 4.446 | 1.385 | LOC_Os05g13770 | RNA polymerase Rpb7, N-terminal domain containing protein |
| OsAffx.26937.3.S1_at | 0.410 | 0.507 | 0.496 | 1.341 | LOC_Os05g16930 | Protein kinase domain containing protein, expressed |
| Os.11897.1.S1_at | 0.410 | 14.239 | 10.240 | 1.103 | LOC_Os03g61150 | expressed protein |
| OsAffx.23947.1.S1_at | 0.410 | 0.753 | 0.384 | 1.465 | LOC_Os01g66740 | Leucine Rich Repeat family protein |

| ProbeSetID | *OMTN2* -OE[a] | Stress response[b] | | | TIGR Locus ID[c] | Annotation |
|---|---|---|---|---|---|---|
| | | Drought | Salt | Cold | | |
| OsAffx.16075.1.S1_s_at | 0.411 | 1.553 | 0.825 | 2.193 | LOC_Os07g04130 | lectin receptor kinase 7, putative |
| OsAffx.8988.1.S1_at | 0.411 | 0.354 | 0.292 | 1.083 | LOC_Os01g02550 | Protein kinase domain containing protein, expressed |
| Os.46574.1.S1_at | 0.413 | 0.180 | 0.527 | 1.105 | LOC_Os10g42040 | expressed protein |
| OsAffx.28169.1.S1_at | 0.413 | 0.771 | 1.311 | 1.529 | LOC_Os06g48250 | ATPase, AAA family protein, expressed |
| Os.53407.1.S1_at | 0.414 | 3.573 | 1.288 | 0.763 | LOC_Os08g06210 | expressed protein |
| Os.27799.1.S1_at | 0.414 | 0.743 | 0.619 | 1.254 | LOC_Os04g52190 | Vacuolar sorting receptor 7 precursor, putative, expressed |
| OsAffx.17800.2.S1_at | 0.415 | 0.359 | 0.463 | 1.288 | LOC_Os09g20500 | hypothetical protein |
| Os.49329.1.S1_at | 0.415 | 0.758 | 0.337 | 1.255 | LOC_Os02g35490 | MLO-like protein 1, putative, expressed |
| Os.49410.1.A1_at | 0.415 | 3.582 | 3.653 | 1.563 | LOC_Os04g33240 | oxidoreductase, short chain dehydrogenase/reductase family protein, expressed |
| Os.30886.1.S1_x_at | 0.416 | 2.914 | 3.315 | 1.260 | LOC_Os03g61360 | hydrolase, alpha/beta fold family protein, expressed |
| Os.52189.1.S1_at | 0.417 | 0.265 | 0.489 | 0.906 | LOC_Os06g19260 | expressed protein |
| Os.26755.1.S1_at | 0.417 | 0.280 | 0.700 | 0.626 | LOC_Os01g48610 | expressed protein |
| Os.26983.1.S1_s_at | 0.417 | 0.263 | 0.403 | 0.524 | LOC_Os01g04570 | Protein kinase domain containing protein, expressed |
| OsAffx.3951.1.S1_at | 0.418 | 0.702 | 0.226 | 0.806 | LOC_Os04g30340 | wall-associated kinase 2, putative |
| OsAffx.29638.1.S1_at | 0.418 | 1.086 | 0.293 | 1.000 | LOC_Os08g42840 | Leucine Rich Repeat family protein |
| Os.55501.1.S1_at | 0.419 | 6.750 | 13.467 | 2.400 | LOC_Os02g07170 | myb-like DNA-binding domain, SHAQKYF class family protein, expressed |
| Os.15996.1.S1_at | 0.419 | 0.548 | 0.409 | 1.000 | LOC_Os07g38250 | Protein kinase domain containing protein, expressed |
| Os.316.1.S1_at | 0.420 | 0.735 | 1.436 | 0.817 | LOC_Os07g01560 | Glucose transporter, putative, expressed |
| Os.25556.1.S1_x_at | 0.420 | 0.398 | 0.448 | 0.856 | LOC_Os08g28400 | expressed protein |
| Os.50127.1.S1_at | 0.420 | 0.877 | 1.261 | 0.433 | LOC_Os04g30030 | Protein kinase domain containing protein |
| Os.54385.1.S1_at | 0.420 | 0.889 | 0.715 | 1.847 | LOC_Os04g01320 | D-mannose binding lectin family protein, expressed |
| Os.6786.1.S1_a_at | 0.420 | 0.268 | 0.332 | 0.780 | LOC_Os10g40720 | Beta-expansin 1a precursor, putative, expressed |
| Os.10150.1.S1_at | 0.421 | 3.820 | 3.981 | 2.319 | LOC_Os03g12890 | Branched-chain-amino-acid aminotransferase 5, chloroplast precursor, putative, expressed |

| ProbeSetID | *OMTN2* -OE[a] | Stress response[b] | | | TIGR Locus ID[c] | Annotation |
|---|---|---|---|---|---|---|
| | | Drought | Salt | Cold | | |
| Os.18463.1.S1_at | 0.421 | 9.807 | 2.097 | 5.040 | LOC_Os05g37660 | AER, putative, expressed |
| Os.57134.1.S1_at | 0.421 | 1.723 | 1.541 | 1.959 | LOC_Os02g39890 | RNA recognition motif family protein, expressed |
| Os.53614.1.S1_at | 0.423 | 4.912 | 1.703 | 0.580 | LOC_Os02g51930 | Cytokinin-O-glucosyltransferase 2, putative, expressed |
| Os.44751.1.S1_at | 0.423 | 0.959 | 0.901 | 1.390 | LOC_Os06g06760 | U-box domain containing protein, expressed |
| Os.30258.1.S1_at | 0.423 | 0.260 | 0.688 | 1.125 | LOC_Os01g58240 | Subtilisin N-terminal Region family protein, expressed |
| Os.32630.1.S1_at | 0.423 | 0.366 | 0.428 | 1.553 | LOC_Os10g32700 | hypersensitive-induced response protein, putative, expressed |
| Os.7065.1.S1_at | 0.424 | 0.474 | 0.723 | 0.607 | LOC_Os02g42240 | expressed protein |
| Os.27067.2.A1_at | 0.424 | 0.472 | 2.505 | 0.865 | LOC_Os08g02230 | plant-specific FAD-dependent oxidoreductase family protein, expressed |
| Os.49074.1.A1_at | 0.425 | 3.432 | 0.719 | 0.502 | LOC_Os07g37290 | hypothetical protein |
| OsAffx.2270.1.S1_at | 0.425 | 1.521 | 1.619 | 1.250 | LOC_Os01g48020 | D-mannose binding lectin family protein, expressed |
| Os.15825.1.S1_at | 0.426 | 1.553 | 0.757 | 1.454 | LOC_Os04g53720 | SNF2 domain-containing protein, putative, expressed |
| Os.37641.1.S1_at | 0.426 | 1.004 | 0.347 | 1.114 | LOC_Os01g43050 | CENP-C1, putative, expressed |
| Os.10251.1.S1_at | 0.426 | 0.236 | 18.621 | 0.946 | LOC_Os10g33370 | beta-ketoacyl-CoA synthase family protein, putative, expressed |
| OsAffx.21092.2.S1_at | 0.426 | 0.326 | 0.874 | 0.407 | LOC_Os01g26310 | expressed protein |
| Os.8531.1.S1_at | 0.427 | 0.588 | 0.627 | 1.391 | LOC_Os04g15690 | DSBA-like thioredoxin domain containing protein, expressed |
| OsAffx.15371.1.S1_at | 0.427 | 0.713 | 0.323 | 0.717 | LOC_Os06g11520 | LMBR1 integral membrane family protein, putative, expressed |
| Os.49400.1.S2_s_at | 0.428 | 1.133 | 0.830 | 0.786 | LOC_Os11g11960 | NBS-LRR type disease resistance protein, putative, expressed |
| Os.47945.1.A1_at | 0.428 | 0.810 | 0.490 | 0.792 | LOC_Os06g37510 | senescence-associated protein, putative, expressed |
| Os.15000.1.S1_a_at | 0.428 | 0.727 | 0.883 | 1.197 | LOC_Os03g41350 | transposon protein, putative, Mutator sub-class, expressed |
| Os.9524.1.S1_at | 0.428 | 1.555 | 1.232 | 0.839 | LOC_Os01g46350 | expressed protein |
| Os.56191.1.S1_at | 0.429 | 0.975 | 3.814 | 1.606 | LOC_Os09g13890 | calmodulin-binding protein, putative, expressed |
| Os.53779.1.S1_x_at | 0.429 | 0.964 | 1.297 | 0.989 | LOC_Os06g48680 | expressed protein |
| Os.27279.1.A1_at | 0.429 | 26.757 | 6.295 | 1.570 | LOC_Os03g26870 | WD-40 repeat family protein, putative, expressed |
| Os.4862.1.S1_at | 0.430 | 10.023 | 7.398 | 1.558 | LOC_Os02g50350 | Dihydroorotate dehydrogenase family protein, expressed |

| ProbeSetID | *OMTN2* -OE[a] | Stress response[b] | | | TIGR Locus ID[c] | Annotation |
|---|---|---|---|---|---|---|
| | | Drought | Salt | Cold | | |
| Os.52868.1.S1_at | 0.430 | 4.743 | 2.178 | 2.663 | LOC_Os02g54590 | serine threonine kinase 1, putative, expressed |
| OsAffx.25832.1.S1_at | 0.430 | 0.484 | 1.325 | 1.302 | LOC_Os04g02030 | NB-ARC domain containing protein |
| Os.57191.1.S1_at | 0.430 | 16.608 | 9.364 | 4.114 | LOC_Os06g05470 | expressed protein |
| OsAffx.30204.1.S1_at | 0.431 | 1.521 | 0.827 | 1.311 | LOC_Os09g39190 | Copine family protein, expressed |
| Os.39087.1.S1_at | 0.432 | 1.762 | 1.344 | 1.987 | LOC_Os01g14550 | Pathogen-related protein, putative, expressed |
| Os.55823.1.S1_at | 0.433 | 0.718 | 1.641 | 2.370 | LOC_Os12g24490 | Zinc finger, C3HC4 type family protein, expressed |
| Os.11321.1.S1_at | 0.434 | 0.607 | 0.665 | 1.192 | LOC_Os07g48260 | WRKY DNA binding domain containing protein, expressed |
| OsAffx.12774.1.S1_s_at | 0.435 | 2.893 | 1.850 | 1.146 | LOC_Os03g07890 | mitochondrial carrier, putative, expressed |
| OsAffx.23406.1.S1_at | 0.435 | 0.836 | 0.954 | 1.289 | LOC_Os01g26300 | wall-associated kinase 1, putative, expressed |
| Os.53726.1.S1_at | 0.435 | 5.651 | 4.368 | 0.765 | LOC_Os07g05370 | protein kinase family protein, putative, expressed |
| Os.9859.1.S1_at | 0.436 | 0.551 | 0.507 | 0.353 | LOC_Os01g08000 | Histone-lysine N-methyltransferase, H3 lysine-9 specific 5, putative, expressed |
| Os.17652.1.S1_s_at | 0.436 | 3.169 | 5.366 | 1.230 | LOC_Os03g43720 | major facilitator superfamily protein, expressed |
| Os.51747.1.S1_at | 0.437 | 0.592 | 0.973 | 2.006 | LOC_Os03g29250 | SPX domain-containing protein, putative, expressed |
| Os.9764.1.S1_at | 0.437 | 0.942 | 1.040 | 1.823 | LOC_Os12g36940 | calmodulin-binding protein, putative, expressed |
| Os.8851.1.S1_at | 0.437 | 2.663 | 6.091 | 2.317 | LOC_Os08g35630 | MTD1, putative, expressed |
| Os.11997.1.S1_at | 0.438 | 2.079 | 1.556 | 1.168 | LOC_Os07g48510 | Thioredoxin-like 1, putative, expressed |
| Os.6114.1.S1_at | 0.438 | 1.929 | 1.544 | 1.475 | LOC_Os05g09500 | Hexokinase 1, putative, expressed |
| Os.56252.1.S1_at | 0.439 | 4.063 | 1.063 | 0.719 | LOC_Os01g57540 | Serine/threonine-protein kinase receptor precursor, putative, expressed |
| OsAffx.19409.1.S1_at | 0.439 | 0.798 | 1.074 | 4.479 | LOC_Os09g11780 | hypothetical protein |
| Os.7539.1.S1_at | 0.439 | 1.086 | 0.825 | 1.218 | LOC_Os03g03320 | expressed protein |
| OsAffx.6930.1.S1_at | 0.440 | 0.601 | 0.731 | 0.939 | LOC_Os10g40810 | GATA zinc finger family protein, expressed |
| Os.36104.1.S1_at | 0.440 | 0.397 | 0.405 | 1.280 | LOC_Os02g31030 | glycerophosphoryl diester phosphodiesterase family protein, putative, expressed |
| Os.38099.1.S1_at | 0.441 | 34.297 | 14.997 | 0.629 | LOC_Os03g61270 | endo-beta-mannanase, putative, expressed |

| ProbeSetID | *OMTN2* -OE[a] | Stress response[b] | | | TIGR Locus ID[c] | Annotation |
|---|---|---|---|---|---|---|
| | | Drought | Salt | Cold | | |
| Os.40189.1.S1_at | 0.441 | 0.269 | 0.643 | 1.494 | LOC_Os07g23340 | expressed protein |
| Os.27474.1.S1_at | 0.441 | 0.496 | 1.155 | 1.217 | LOC_Os05g16430 | SHR5-receptor-like kinase, putative, expressed |
| Os.34283.2.S1_x_at | 0.441 | 0.796 | 0.874 | 1.026 | LOC_Os01g12160 | GH3 auxin-responsive promoter family protein, expressed |
| OsAffx.11789.1.S1_s_at | 0.441 | 0.092 | 0.083 | 0.712 | LOC_Os01g68470 | hypothetical protein |
| OsAffx.12740.1.S1_s_at | 0.442 | 1.102 | 0.834 | 1.145 | LOC_Os03g05520 | expressed protein |
| Os.56988.1.S1_at | 0.442 | 1.338 | 0.632 | 1.368 | LOC_Os03g53730 | flavodoxin family protein, expressed |
| OsAffx.22380.1.S1_at | 0.442 | 1.580 | 0.910 | 3.608 | LOC_Os07g39680 | XYPPX repeat family protein, expressed |
| Os.10830.1.S1_at | 0.442 | 0.487 | 0.660 | 0.884 | LOC_Os08g41320 | Helix-loop-helix DNA-binding domain containing protein, expressed |
| Os.17286.1.S1_at | 0.442 | 7.004 | 4.069 | 0.763 | LOC_Os03g21060 | NAC domain transcription factor, putative, expressed |
| Os.4458.1.S1_at | 0.443 | 1.265 | 1.362 | 2.028 | LOC_Os06g03580 | Zinc finger, C3HC4 type family protein, expressed |
| OsAffx.32186.1.S1_at | 0.443 | 0.883 | 1.508 | 0.917 | LOC_Os12g44250 | Synaptobrevin family protein, expressed |
| Os.7826.1.S1_a_at | 0.444 | 0.714 | 0.955 | 1.008 | LOC_Os05g10740 | Pollen-specific protein SF21, putative, expressed |
| Os.19843.1.S1_at | 0.444 | 6.086 | 2.346 | 0.962 | LOC_Os01g55100 | TCP1 protein, putative, expressed |
| Os.5853.2.S1_x_at | 0.444 | 0.313 | 0.384 | 0.762 | LOC_Os12g44230 | HIPL1 protein precursor, putative, expressed |
| Os.26698.1.S1_a_at | 0.444 | 2.153 | 3.198 | 1.527 | LOC_Os01g19820 | universal stress protein family protein, expressed |
| Os.2423.1.S1_at | 0.446 | 0.552 | 1.786 | 1.560 | LOC_Os12g36850 | Pathogenesis-related protein Bet v I family protein, expressed |
| Os.8618.1.S1_at | 0.446 | 3.417 | 1.755 | 0.926 | LOC_Os05g44810 | AUX/IAA family protein, expressed |
| OsAffx.12626.1.S1_at | 0.446 | 0.189 | 0.222 | 0.751 | LOC_Os02g53620 | CCAAT-binding transcription factor subunit B family protein, expressed |
| Os.54660.1.S1_at | 0.447 | 0.723 | 0.556 | 1.323 | LOC_Os03g44540 | CCAAT-binding transcription factor subunit B family protein, expressed |
| Os.15982.1.S1_s_at | 0.449 | 0.760 | 1.046 | 0.699 | LOC_Os02g30150 | NB-ARC domain containing protein, expressed |
| Os.37603.1.S1_at | 0.449 | 1.837 | 4.689 | 1.265 | LOC_Os01g63480 | AER, putative, expressed |
| Os.55380.1.S1_at | 0.449 | 107.958 | 64.986 | 2.032 | LOC_Os06g46740 | Plastocyanin-like domain containing protein, expressed |
| OsAffx.28152.2.S1_at | 0.450 | 2.281 | 3.026 | 1.104 | LOC_Os06g47470 | Protein kinase domain containing protein, expressed |
| Os.35858.1.S1_at | 0.450 | 0.730 | 0.850 | 0.904 | LOC_Os01g44110 | Protein kinase domain containing protein, expressed |

| ProbeSetID | *OMTN2* -OE[a] | Stress response[b] | | | TIGR Locus ID[c] | Annotation |
|---|---|---|---|---|---|---|
| | | Drought | Salt | Cold | | |
| Os.15191.1.S1_s_at | 0.450 | 1.967 | 2.532 | 2.035 | LOC_Os05g41220 | SNF1-related protein kinase regulatory beta subunit 1, putative, expressed |
| OsAffx.5290.1.S1_at | 0.451 | 0.171 | 0.286 | 0.634 | LOC_Os07g10970 | expressed protein |
| Os.53422.1.S1_at | 0.451 | 0.398 | 1.102 | 0.766 | LOC_Os02g15460 | expressed protein |
| OsAffx.14380.1.S1_s_at | 0.451 | 1.907 | 1.642 | 3.609 | LOC_Os04g52670 | Auxin responsive protein, expressed |
| Os.32668.1.S1_at | 0.451 | 0.553 | 1.513 | 1.695 | LOC_Os01g08090 | Flavonol-3-O-glycoside-7-O-glucosyltransferase 1, putative, expressed |
| Os.27517.1.A1_s_at | 0.451 | 0.979 | 1.746 | 1.203 | LOC_Os11g14140 | Kelch motif family protein, expressed |
| Os.49112.1.S1_at | 0.452 | 5.459 | 4.156 | 1.317 | LOC_Os02g37180 | expressed protein |
| OsAffx.17900.1.S1_at | 0.452 | 1.243 | 0.655 | 0.508 | LOC_Os09g25940 | Receptor family ligand binding region containing protein |
| Os.5594.1.S1_at | 0.452 | 3.823 | 2.911 | 1.173 | LOC_Os05g48340 | proteasome, putative, expressed |
| OsAffx.12887.2.S1_s_at | 0.453 | 25.013 | 17.562 | 1.386 | LOC_Os03g16920 | Heat shock cognate 70 kDa protein, putative, expressed |
| OsAffx.18875.1.S1_at | 0.453 | 0.697 | 0.860 | 0.947 | LOC_Os11g15700 | NB-ARC domain containing protein, expressed |
| OsAffx.24812.1.S1_s_at | 0.453 | 1.044 | 4.093 | 0.044 | LOC_Os02g50460 | U-box domain containing protein, expressed |
| Os.38192.1.S1_at | 0.453 | 0.620 | 0.852 | 1.205 | LOC_Os07g14160 | Polygalacturonase family protein, expressed |
| Os.37834.1.S1_a_at | 0.453 | 4.016 | 3.305 | 2.766 | LOC_Os04g58280 | Stem-specific protein TSJT1, putative, expressed |
| Os.52931.1.S1_at | 0.454 | 3.269 | 3.936 | 1.235 | LOC_Os02g38040 | Leucine Rich Repeat family protein, expressed |
| Os.15914.1.S1_at | 0.454 | 2.818 | 2.593 | 1.345 | LOC_Os09g23350 | glycosyl transferase family 20 protein, putative, expressed |
| Os.19277.1.S1_at | 0.455 | 0.291 | 0.964 | 0.845 | LOC_Os07g04150 | expressed protein |
| Os.38006.1.S1_at | 0.455 | 1.724 | 1.549 | 1.349 | LOC_Os03g20530 | expressed protein |
| Os.40406.1.A1_at | 0.455 | 0.305 | 1.024 | 0.951 | LOC_Os06g38110 | expressed protein |
| Os.19369.1.S1_at | 0.455 | 1.610 | 1.080 | 0.969 | LOC_Os12g05990 | No apical meristem protein, expressed |
| Os.27765.1.S1_at | 0.456 | 0.280 | 1.206 | 0.335 | LOC_Os06g50080 | expressed protein |
| OsAffx.28651.1.S1_x_at | 0.456 | 1.429 | 1.527 | 2.905 | LOC_Os07g28850 | Piwi domain containing protein, expressed |
| Os.54961.1.S1_at | 0.456 | 5.200 | 1.663 | 2.109 | LOC_Os05g46350 | IQ calmodulin-binding motif family protein, expressed |
| Os.46839.1.S1_at | 0.456 | 0.277 | 0.562 | 1.052 | LOC_Os10g02300 | Protein of unknown function, DUF614 containing protein, expressed |

| ProbeSetID | *OMTN2* -OE[a] | Stress response[b] | | | TIGR Locus ID[c] | Annotation |
|---|---|---|---|---|---|---|
| | | Drought | Salt | Cold | | |
| Os.30376.1.S1_at | 0.457 | 1.004 | 1.603 | 1.411 | LOC_Os01g02130 | expressed protein |
| Os.50359.1.S1_at | 0.457 | 1.828 | 1.706 | 2.206 | LOC_Os09g26460 | zinc finger family protein, putative, expressed |
| Os.19393.1.S1_at | 0.457 | 0.166 | 0.569 | 0.782 | LOC_Os02g36110 | Cytochrome P450 76C2, putative, expressed |
| Os.49496.1.S1_at | 0.457 | 0.474 | 0.540 | 0.970 | LOC_Os06g46500 | L-ascorbate oxidase homolog precursor, putative, expressed |
| Os.5434.1.S1_at | 0.457 | 0.594 | 0.509 | 0.785 | LOC_Os08g26850 | expressed protein |
| OsAffx.18131.3.S1_at | 0.458 | 0.271 | 0.602 | 0.161 | LOC_Os10g04570 | NB-ARC domain containing protein |
| Os.46812.1.S1_at | 0.458 | 0.778 | 1.400 | 2.600 | LOC_Os10g38450 | Leucine Rich Repeat family protein, expressed |
| Os.55827.1.S1_at | 0.458 | 6.560 | 7.058 | 1.370 | LOC_Os11g29870 | WRKY DNA binding domain containing protein, expressed |
| Os.35935.2.S1_at | 0.459 | 4.341 | 1.545 | 4.909 | LOC_Os07g31250 | Protein kinase domain containing protein, expressed |
| Os.10870.1.S1_at | 0.460 | 4.511 | 2.599 | 1.896 | LOC_Os02g51890 | RNA recognition motif family protein, expressed |
| Os.18595.1.A1_at | 0.460 | 3.019 | 5.054 | 1.253 | LOC_Os07g48450 | NAC-domain containing protein 18, putative, expressed |
| Os.10862.1.S1_at | 0.460 | 0.859 | 6.572 | 1.364 | LOC_Os06g05410 | expressed protein |
| Os.4763.1.S2_at | 0.460 | 1.608 | 1.025 | 2.304 | LOC_Os01g04650 | PB1 domain containing protein, expressed |
| Os.9344.1.S1_x_at | 0.460 | 1.944 | 1.581 | 1.546 | LOC_Os03g53800 | Glycosyl hydrolase family 3 N terminal domain containing protein, expressed |
| Os.10185.1.S1_at | 0.460 | 1.212 | 0.952 | 0.221 | LOC_Os11g31470 | expressed protein |
| Os.51029.1.S1_at | 0.461 | 3.780 | 5.736 | 1.363 | LOC_Os09g02770 | expressed protein |
| Os.32212.1.A1_at | 0.461 | 2.478 | 0.723 | 1.031 | LOC_Os01g10130 | RNA-dependent RNA polymerase family protein, putative, expressed |
| Os.50019.1.S1_at | 0.461 | 761.382 | 105.602 | 4.463 | LOC_Os03g04080 | expressed protein |
| Os.15706.1.S1_a_at | 0.461 | 1.408 | 0.692 | 1.753 | LOC_Os01g47580 | Lipid phosphate phosphatase 2, putative, expressed |
| Os.3426.2.S1_x_at | 0.462 | 0.467 | 0.933 | 0.486 | LOC_Os03g07880 | CCAAT-binding transcription factor subunit B family protein, expressed |
| Os.50494.1.A1_at | 0.462 | 1.795 | 1.476 | 1.457 | LOC_Os06g50180 | Non-imprinted in Prader-Willi/Angelman syndrome region protein 2homolog, putative, expressed |
| OsAffx.6449.1.S1_at | 0.462 | 1.795 | 1.476 | 1.457 | LOC_Os09g30170 | F-box protein interaction domain containing protein, expressed |

| ProbeSetID | *OMTN2* -OE[a] | Stress response[b] | | | TIGR Locus ID[c] | Annotation |
|---|---|---|---|---|---|---|
| | | Drought | Salt | Cold | | |
| Os.49615.1.S1_at | 0.463 | 0.566 | 0.848 | 1.216 | LOC_Os03g45960 | P21 protein, putative, expressed |
| Os.3625.1.S1_at | 0.463 | 0.845 | 1.067 | 1.151 | LOC_Os01g18660 | Isopenicillin N epimerase, putative, expressed |
| Os.1563.1.S1_at | 0.463 | 1.144 | 1.108 | 0.994 | LOC_Os01g22370 | Peroxidase family protein, expressed |
| Os.15185.1.S1_s_at | 0.464 | 0.404 | 0.687 | 1.006 | LOC_Os05g48390 | Ubiquitin-conjugating enzyme family protein, expressed |
| Os.49354.1.S1_at | 0.464 | 0.404 | 0.687 | 1.006 | LOC_Os07g48730 | serine/threonine-protein kinase NAK, putative, expressed |
| Os.49973.1.S1_at | 0.465 | 0.879 | 0.436 | 1.258 | LOC_Os03g20210 | Eukaryotic aspartyl protease family protein, expressed |
| Os.15152.2.S1_at | 0.465 | 2.649 | 2.715 | 1.111 | LOC_Os05g45350 | DnaJ domain containing protein, expressed |
| Os.4780.1.S1_at | 0.465 | 3.321 | 2.573 | 1.080 | LOC_Os03g55290 | Gibberellin-regulated protein 2 precursor, putative, expressed |
| Os.10784.1.S1_at | 0.465 | 2.745 | 1.943 | 1.799 | LOC_Os02g26700 | ChaC-like protein, expressed |
| Os.27738.1.S1_at | 0.465 | 0.372 | 0.552 | 0.738 | LOC_Os07g05620 | CIPK-like protein 1, putative, expressed |
| Os.27875.1.S1_at | 0.465 | 0.724 | 2.518 | 1.782 | LOC_Os10g28120 | Chitinase 1 precursor, putative, expressed |
| OsAffx.23671.2.S1_x_at | 0.467 | 0.496 | 0.552 | 1.090 | LOC_Os01g45520 | D-mannose binding lectin family protein |
| Os.17102.1.S1_at | 0.467 | 1.519 | 1.488 | 1.507 | LOC_Os01g66130 | arm repeat-containing protein, putative, expressed |
| OsAffx.7086.1.S1_at | 0.467 | 0.442 | 0.653 | 0.923 | LOC_Os11g11970 | expressed protein |
| Os.17189.1.S1_at | 0.468 | 0.272 | 0.517 | 1.246 | LOC_Os05g44070 | Ras-related protein RIC2, putative, expressed |
| Os.54153.1.S1_at | 0.468 | 0.889 | 0.631 | 0.438 | LOC_Os08g24310 | Leucine Rich Repeat family protein, expressed |
| Os.33210.1.S1_at | 0.468 | 1.869 | 1.445 | 0.759 | LOC_Os01g68570 | expressed protein |
| Os.46544.2.S1_x_at | 0.468 | 0.355 | 0.273 | 0.556 | LOC_Os10g25060 | expressed protein |
| Os.42318.2.S1_x_at | 0.468 | 0.743 | 1.965 | 0.776 | LOC_Os01g57870 | NB-ARC domain containing protein, expressed |
| Os.33229.1.S1_at | 0.469 | 1.004 | 1.553 | 0.940 | LOC_Os06g06450 | U-box domain containing protein, expressed |
| Os.53055.1.S1_at | 0.470 | 0.712 | 0.798 | 0.963 | LOC_Os11g31450 | expressed protein |
| Os.56907.1.S1_at | 0.470 | 0.783 | 0.317 | 1.116 | LOC_Os09g27510 | Cytochrome P450 family protein, expressed |
| Os.55254.1.S1_at | 0.470 | 1.036 | 1.313 | 1.133 | LOC_Os06g10130 | expressed protein |
| OsAffx.28409.2.S1_s_at | 0.470 | 0.807 | 0.880 | 2.089 | LOC_Os07g12240 | EF hand family protein |

| ProbeSetID | *OMTN2* -OE[a] | Stress response[b] | | | TIGR Locus ID[c] | Annotation |
|---|---|---|---|---|---|---|
| | | Drought | Salt | Cold | | |
| Os.27727.1.S1_s_at | 0.470 | 0.776 | 1.017 | 1.135 | LOC_Os04g02510 | Zinc finger, C2H2 type family protein, expressed |
| Os.56006.1.S1_at | 0.470 | 1.641 | 0.862 | 0.655 | LOC_Os06g08610 | Transferase family protein, expressed |
| Os.47914.1.S1_at | 0.471 | 0.642 | 0.982 | 0.971 | LOC_Os06g11210 | 12-oxophytodienoate reductase 2, putative, expressed |
| Os.14052.1.S1_at | 0.471 | 5.155 | 5.618 | 1.500 | LOC_Os03g45210 | expressed protein |
| OsAffx.24300.1.S1_at | 0.472 | 1.306 | 1.125 | 1.444 | LOC_Os02g18080 | NB-ARC domain containing protein, expressed |
| OsAffx.26624.1.S1_at | 0.472 | 0.590 | 1.361 | 2.082 | LOC_Os04g56040 | Glycine rich protein family protein, expressed |
| Os.17554.1.S1_at | 0.472 | 1.741 | 3.722 | 0.937 | LOC_Os08g37760 | Zinc finger, C3HC4 type family protein, expressed |
| OsAffx.28689.1.S1_at | 0.472 | 0.444 | 0.435 | 0.287 | LOC_Os07g31190 | expressed protein |
| Os.13596.1.S1_at | 0.473 | 3.308 | 2.756 | 0.812 | LOC_Os03g10320 | expressed protein |
| Os.18230.1.S1_at | 0.473 | 3.308 | 2.756 | 0.812 | LOC_Os08g40990 | Leucine Rich Repeat family protein, expressed |
| Os.54936.1.S1_at | 0.473 | 34.517 | 27.457 | 2.241 | LOC_Os03g57640 | PrMC3, putative, expressed |
| Os.10855.1.S1_at | 0.474 | 4.595 | 1.533 | 1.105 | LOC_Os03g56060 | glycosyl transferase, group 2 family protein, expressed |
| Os.49023.1.S1_x_at | 0.474 | 1.067 | 1.645 | 0.914 | LOC_Os03g22680 | CHY zinc finger family protein, expressed |
| Os.20572.2.S1_at | 0.474 | 0.118 | 0.471 | 0.702 | LOC_Os03g22620 | Terpene synthase family, metal binding domain containing protein, expressed |
| Os.26811.1.A1_at | 0.475 | 1.721 | 1.918 | 1.934 | LOC_Os06g39780 | Cytochrome P450 family protein, expressed |
| OsAffx.17460.1.S1_at | 0.475 | 0.762 | 0.830 | 1.358 | LOC_Os08g41590 | POT family protein, expressed |
| Os.55076.1.S1_at | 0.476 | 0.470 | 1.608 | 0.822 | LOC_Os11g05820 | transposon protein, putative, Mutator sub-class, expressed |
| Os.5768.1.S1_at | 0.476 | 2.041 | 2.326 | 1.237 | LOC_Os09g15320 | Ubiquitin-conjugating enzyme E2 M, putative, expressed |
| OsAffx.18050.2.S1_at | 0.476 | 0.392 | 0.174 | 0.571 | LOC_Os02g56370 | wall-associated kinase-like 1, putative, expressed |
| Os.8136.1.A1_at | 0.476 | 0.921 | 2.511 | 1.132 | LOC_Os06g40030 | D-mannose binding lectin family protein, expressed |
| Os.27793.1.S1_at | 0.476 | 0.583 | 0.764 | 0.556 | LOC_Os02g14430 | Peroxidase 52 precursor, putative, expressed |
| Os.17446.1.S1_at | 0.476 | 7.075 | 4.708 | 1.690 | LOC_Os04g48270 | F-box family protein, putative, expressed |
| Os.21024.1.S1_at | 0.477 | 0.630 | 0.861 | 0.665 | LOC_Os03g63900 | oxidoreductase, 2OG-Fe oxygenase family protein, expressed |
| OsAffx.24724.1.S1_x_at | 0.477 | 0.483 | 0.637 | 1.210 | LOC_Os02g44600 | U-box domain containing protein, expressed |

| ProbeSetID | *OMTN2* -OE[a] | Stress response[b] | | | TIGR Locus ID[c] | Annotation |
|---|---|---|---|---|---|---|
| | | Drought | Salt | Cold | | |
| Os.18023.1.S1_at | 0.478 | 0.669 | 0.633 | 1.171 | LOC_Os11g36030 | AT hook motif family protein, expressed |
| Os.55444.1.S1_at | 0.478 | 1.589 | 1.217 | 0.832 | LOC_Os03g47270 | expressed protein |
| Os.2617.2.S1_a_at | 0.478 | 0.319 | 0.830 | 0.276 | LOC_Os06g48810 | high-affinity sodium transporter, putative, expressed |
| Os.26938.1.A1_at | 0.479 | 0.149 | 0.409 | 0.701 | LOC_Os02g42150 | Calcium binding EGF domain containing protein, expressed |
| Os.7217.1.S1_a_at | 0.479 | 0.621 | 0.814 | 1.123 | LOC_Os01g07140 | Kelch motif family protein, expressed |
| Os.24008.1.S2_at | 0.479 | 0.830 | 0.831 | 0.766 | LOC_Os05g32170 | Protein kinase domain containing protein, expressed |
| Os.49577.1.S1_at | 0.479 | 9.844 | 6.365 | 1.102 | LOC_Os02g50340 | MAC/Perforin domain containing protein, expressed |
| Os.55612.1.S1_at | 0.479 | 0.239 | 0.142 | 1.034 | LOC_Os03g18600 | Streptomyces cyclase/dehydrase family protein, expressed |
| Os.23977.1.S1_at | 0.480 | 0.378 | 0.538 | 0.763 | LOC_Os03g43510 | expressed protein |
| Os.773.1.S1_s_at | 0.480 | 1.132 | 1.687 | 1.154 | LOC_Os01g43750 | Cytochrome P450 family protein, expressed |
| Os.54351.1.S1_at | 0.480 | 3.776 | 3.215 | 3.694 | LOC_Os08g31250 | C1-like domain containing protein, expressed |
| Os.17437.1.S1_at | 0.480 | 0.265 | 1.358 | 1.169 | LOC_Os01g53390 | Anthocyanidin 5,3-O-glucosyltransferase, putative, expressed |
| OsAffx.12561.1.S1_s_at | 0.481 | 0.508 | 0.490 | 0.754 | LOC_Os02g49160 | AUX/IAA family protein, expressed |
| Os.41723.1.S1_at | 0.481 | 0.653 | 1.069 | 1.210 | LOC_Os01g48620 | expressed protein |
| OsAffx.18807.1.S1_at | 0.481 | 0.947 | 0.579 | 1.026 | LOC_Os11g11770 | NB-ARC domain containing protein, expressed |
| OsAffx.30194.1.S1_x_at | 0.481 | 1.372 | 1.937 | 0.679 | LOC_Os09g38840 | wall-associated kinase-like 1, putative, expressed |
| Os.44516.1.S1_x_at | 0.482 | 0.632 | 1.264 | 1.042 | LOC_Os07g05010 | Helix-loop-helix DNA-binding domain containing protein, expressed |
| Os.4683.2.S1_at | 0.483 | 0.925 | 0.695 | 0.888 | LOC_Os01g42520 | expressed protein |
| Os.38984.1.S1_at | 0.483 | 1.139 | 0.861 | 0.877 | LOC_Os01g23580 | Pyrophosphate-energized vacuolar membrane proton pump, putative, expressed |
| OsAffx.2598.1.S1_at | 0.483 | 1.379 | 3.621 | 6.172 | LOC_Os02g13430 | Leucine Rich Repeat family protein, expressed |
| Os.6125.1.S1_at | 0.484 | 0.723 | 0.785 | 1.460 | LOC_Os03g29190 | PDI, putative, expressed |
| OsAffx.29181.1.S1_at | 0.484 | 2.395 | 3.342 | 3.237 | LOC_Os08g13870 | D-mannose binding lectin family protein |
| OsAffx.20724.2.S1_s_at | 0.484 | 1.834 | 2.700 | 0.748 | LOC_Os10g07010 | senescence-associated protein 15, putative |

| ProbeSetID | *OMTN2* -OE[a] | Stress response[b] | | | TIGR Locus ID[c] | Annotation |
|---|---|---|---|---|---|---|
| | | Drought | Salt | Cold | | |
| Os.17181.1.S1_at | 0.484 | 1.834 | 2.700 | 0.748 | LOC_Os07g32010 | UDP-glucoronosyl and UDP-glucosyl transferase family protein, expressed |
| Os.35583.1.S1_at | 0.484 | 0.180 | 0.535 | 0.499 | LOC_Os01g48950 | expressed protein |
| OsAffx.32110.1.S1_at | 0.484 | 0.389 | 0.793 | 0.605 | LOC_Os12g40010 | expressed protein |
| Os.17479.1.S1_at | 0.485 | 0.854 | 0.526 | 1.884 | LOC_Os01g49320 | Acidic endochitinase precursor, putative, expressed |
| OsAffx.12065.1.S1_at | 0.485 | 0.485 | 1.971 | 0.250 | LOC_Os02g16060 | NBS-LRR disease resistance protein, putative, expressed |
| Os.26377.1.S1_at | 0.485 | 1.575 | 2.351 | 1.597 | LOC_Os01g60860 | U-box domain containing protein, expressed |
| OsAffx.2542.1.S1_at | 0.485 | 1.289 | 1.758 | 0.828 | LOC_Os02g07730 | haloacid dehalogenase-like hydrolase family protein, putative, expressed |
| Os.11194.1.S1_at | 0.486 | 0.983 | 1.412 | 1.588 | LOC_Os08g35190 | Dormancy/auxin associated protein, expressed |
| Os.45405.1.A1_at | 0.486 | 0.443 | 0.574 | 1.098 | LOC_Os01g04750 | DNA-binding protein RAV1, putative, expressed |
| Os.22839.1.S1_at | 0.486 | 5.327 | 3.747 | 2.828 | LOC_Os06g48200 | xyloglucan endotransglucosylase/hydrolase protein 15precursor, putative, expressed |
| Os.662.1.S1_at | 0.486 | 0.503 | 0.630 | 0.740 | LOC_Os01g43220 | hypothetical protein |
| Os.37006.1.S1_at | 0.486 | 4.111 | 3.023 | 1.686 | LOC_Os06g11660 | Phosphate-induced protein 1 conserved region containing protein, expressed |
| Os.35049.1.S1_a_at | 0.486 | 0.385 | 0.625 | 1.192 | LOC_Os01g59350 | Transcription factor HBP-1b, putative, expressed |
| Os.17506.1.S1_at | 0.487 | 5.690 | 4.602 | 1.476 | LOC_Os10g32680 | Uncharacterized conserved protein, putative, expressed |
| Os.56369.1.S1_at | 0.487 | 0.748 | 1.433 | 0.344 | LOC_Os09g23780 | expressed protein |
| Os.57316.1.S1_at | 0.487 | 1.357 | 2.739 | 0.792 | LOC_Os05g38940 | expressed protein |
| Os.27864.1.S1_at | 0.487 | 1.437 | 1.343 | 1.322 | LOC_Os04g59200 | Peroxidase family protein, expressed |
| Os.38848.1.S1_at | 0.487 | 0.099 | 0.993 | 2.712 | LOC_Os06g15330 | CCT motif family protein, expressed |
| Os.55746.1.S1_at | 0.487 | 3.472 | 0.778 | 0.778 | LOC_Os11g28530 | Terpene synthase family, metal binding domain containing protein, expressed |
| Os.56111.1.S1_at | 0.488 | 0.425 | 0.941 | 0.757 | LOC_Os04g56120 | Protein kinase domain containing protein, expressed |
| Os.32292.1.S1_at | 0.489 | 1.110 | 1.066 | 0.958 | LOC_Os01g36240 | Peroxidase 72 precursor, putative, expressed |
| Os.51307.1.S1_at | 0.490 | 0.747 | 1.349 | 2.941 | LOC_Os11g47630 | Zinc finger, C2H2 type family protein, expressed |
| OsAffx.17004.1.S1_at | 0.490 | 2.268 | 1.512 | 1.146 | LOC_Os08g15090 | Diacylglycerol kinase accessory domain family protein, expressed |

| ProbeSetID | *OMTN2*-OE[a] | Stress response[b] | | | TIGR Locus ID[c] | Annotation |
|---|---|---|---|---|---|---|
| | | Drought | Salt | Cold | | |
| OsAffx.28664.1.S1_at | 0.490 | 1.150 | 1.264 | 1.388 | LOC_Os07g29810 | NB-ARC domain containing protein |
| Os.27520.4.S1_a_at | 0.491 | 1.932 | 1.526 | 1.672 | LOC_Os10g03850 | F-box domain containing protein, expressed |
| Os.6170.1.S1_at | 0.491 | 0.610 | 0.605 | 1.748 | LOC_Os02g51060 | glycosyl transferase, group 2 family protein, expressed |
| Os.7806.1.S1_at | 0.491 | 3.534 | 5.258 | 3.332 | LOC_Os04g39980 | oxidoreductase, 2OG-Fe oxygenase family protein, expressed |
| Os.8570.4.S1_at | 0.492 | 2.884 | 2.078 | 2.699 | LOC_Os09g32330 | expressed protein |
| OsAffx.5923.1.S1_at | 0.492 | 1.179 | 1.953 | 0.812 | LOC_Os08g25380 | Serine/threonine-protein kinase BRI1-like 1 precursor, putative, expressed |
| Os.12738.1.S1_a_at | 0.492 | 3.013 | 3.234 | 3.053 | LOC_Os01g48960 | Glutamate synthase, chloroplast precursor, putative, expressed |
| Os.20817.1.S2_at | 0.492 | 0.300 | 0.122 | 0.589 | LOC_Os07g03870 | lectin receptor kinase 7, putative, expressed |
| Os.28525.2.S1_at | 0.492 | 0.710 | 1.131 | 0.579 | LOC_Os01g49630 | expressed protein |
| OsAffx.4160.1.S1_at | 0.492 | 0.886 | 2.252 | 0.835 | LOC_Os04g52810 | No apical meristem protein, expressed |
| Os.11408.1.S2_at | 0.492 | 6.034 | 3.263 | 1.221 | LOC_Os09g37100 | Phospholipase D delta, putative, expressed |
| Os.5095.1.S1_at | 0.492 | 0.521 | 0.643 | 0.778 | LOC_Os07g40130 | transposon protein, putative, CACTA, En/Spm sub-class |
| OsAffx.24745.1.S1_at | 0.493 | 0.708 | 0.587 | 0.908 | LOC_Os02g45940 | Histone H4, putative, expressed |
| Os.7678.1.S1_at | 0.493 | 2.222 | 2.997 | 6.049 | LOC_Os03g12500 | Cytochrome P450 74A2, putative, expressed |
| Os.38447.1.S1_s_at | 0.493 | 12.621 | 7.669 | 1.119 | LOC_Os06g07030 | AP2 domain containing protein, expressed |
| OsAffx.4277.1.S1_s_at | 0.494 | 3.532 | 2.605 | 1.421 | LOC_Os05g08750 | cold-induced glucosyl transferase, putative, expressed |
| Os.1503.1.S1_at | 0.495 | 0.792 | 0.612 | 1.478 | LOC_Os06g45140 | bZIP transcription factor family protein, expressed |
| Os.10440.1.S1_a_at | 0.495 | 0.538 | 0.660 | 1.774 | LOC_Os03g29760 | CCAAT-box transcription factor complex WHAP3, putative, expressed |
| OsAffx.12022.1.S1_s_at | 0.495 | 7.202 | 6.607 | 0.893 | LOC_Os02g13600 | expressed protein |
| Os.29008.1.S1_x_at | 0.495 | 1.732 | 2.485 | 0.676 | LOC_Os01g08370 | symbiosis-related disease resistance protein, putative, expressed |
| Os.31375.1.S1_at | 0.495 | 0.785 | 1.002 | 0.844 | LOC_Os01g73410 | Chromosome condensation factor, putative, expressed |
| Os.459.1.S1_at | 0.495 | 0.870 | 1.445 | 1.269 | LOC_Os03g46070 | P21 protein, putative, expressed |
| Os.1189.1.S1_at | 0.496 | 1.511 | 1.239 | 1.212 | LOC_Os06g16370 | Hd1, putative, expressed |
| Os.15679.1.S1_s_at | 0.496 | 0.376 | 0.352 | 0.685 | LOC_Os08g35210 | Ferric reductase like transmembrane component family protein, expressed |

| ProbeSetID | *OMTN2*-OE[a] | Stress response[b] | | | TIGR Locus ID[c] | Annotation |
|---|---|---|---|---|---|---|
| | | Drought | Salt | Cold | | |
| Os.26809.1.S1_at | 0.497 | 0.750 | 0.870 | 2.500 | LOC_Os07g35700 | Protein kinase domain containing protein, expressed |
| Os.50870.2.S1_at | 0.497 | 0.497 | 0.367 | 0.735 | LOC_Os11g10550 | Leucine Rich Repeat family protein, expressed |
| Os.26928.1.S2_at | 0.497 | 1.738 | 3.714 | 2.143 | LOC_Os08g29020 | wall-associated kinase 2, putative, expressed |
| OsAffx.7243.1.S1_s_at | 0.498 | 0.412 | 0.319 | 0.926 | LOC_Os11g29210 | expressed protein |
| Os.35253.1.S1_at | 0.498 | 0.768 | 1.898 | 0.972 | LOC_Os07g03820 | lectin receptor kinase 7, putative, expressed |
| Os.31431.1.S1_at | 0.498 | 0.819 | 1.397 | 1.087 | LOC_Os01g40580 | hypersensitive-induced response protein, putative, expressed |
| Os.9021.4.S1_at | 0.499 | 2.217 | 0.261 | 2.261 | LOC_Os11g08530 | expressed protein |
| Os.54530.1.S1_at | 0.499 | 0.945 | 0.691 | 0.602 | LOC_Os06g49100 | leucine-rich repeat family protein, putative, expressed |
| Os.53236.1.S1_at | 0.500 | 1.354 | 0.593 | 1.832 | LOC_Os02g43170 | B-box zinc finger family protein, expressed |

NOTE: All values in the table are expression level change folds (transgenic/WT, or stress/normal)(mean of the repeats).The folds higher than 2 were indicated by red colour, and the folds lower than 0.5 were indicated by green colour.

[a]Expression level change folds in the *OMTN2*-OE plants.

[b]Expression profile of the genes under drought, salt and cold stress was download from the GEO database (http://www.ncbi.nlm.nih.gov/geo/, accession number: GSE6901).

[c]The locus ID was download from Rice Annotation Project (http://rice.plantbiology.msu.edu/).

**Supplementary Table S5. Up- and down-regulated genes in the transgenic rice plants overexpressing *OMTN3*.**

| ProbeSetID | *OMTN3* -OE[a] | Stress response[b] | | | TIGR Locus ID[c] | Annotation |
|---|---|---|---|---|---|---|
| | | Drought | Salt | Cold | | |
| Os.4184.1.S1_at | 17.441 | 0.343 | 1.364 | 0.904 | LOC_Os02g15350 | Dof domain, zinc finger family protein, expressed |
| Os.4766.1.S1_at | 8.254 | 1.589 | 1.274 | 1.156 | LOC_Os10g28350 | 1,2-dihydroxy-3-keto-5-methylthiopentene dioxygenase, putative, expressed |
| Os.11065.1.A1_at | 7.766 | 1.191 | 0.512 | 1.153 | LOC_Os03g19600 | retrotransposon protein, putative, Ty3-gypsy subclass, expressed |
| Os.18955.1.S1_at | 7.749 | 1.569 | 2.075 | 0.311 | LOC_Os06g51260 | myb-like DNA-binding domain, SHAQKYF class family protein, expressed |
| Os.10266.1.S1_at | 7.733 | 0.167 | 0.362 | 0.630 | LOC_Os03g43100 | expressed protein |
| Os.15829.1.S1_at | 7.718 | 0.382 | 1.711 | 0.307 | LOC_Os02g10120 | Lipoxygenase 2.3, chloroplast precursor, putative, expressed |
| Os.41109.1.S1_at | 6.472 | 3.906 | 6.748 | 0.367 | LOC_Os07g43740 | Zinc finger, C3HC4 type family protein, expressed |
| Os.5698.1.S1_s_at | 5.613 | 7.643 | 4.452 | 1.392 | LOC_Os05g33700 | 4F5 protein family protein |
| Os.17047.1.A1_at | 5.360 | 0.564 | 1.205 | 0.910 | LOC_Os07g18230 | Protein kinase domain containing protein, expressed |
| Os.11941.2.S1_at | 5.031 | 10.409 | 8.796 | 0.409 | LOC_Os09g35790 | HSF-type DNA-binding domain containing protein, expressed |
| Os.12257.1.S1_at | 4.979 | 4.935 | 6.673 | 1.336 | LOC_Os02g52150 | Hsp20/alpha crystallin family protein, expressed |
| Os.39876.1.S1_at | 4.632 | 14.721 | 11.445 | 0.495 | LOC_Os04g48030 | HSF-type DNA-binding domain containing protein, expressed |
| OsAffx.19886.1.S1_at | 4.314 | 0.447 | 0.673 | 1.014 | LOC_Os12g29680 | hypothetical protein |
| Os.30575.1.A1_at | 4.232 | 4.360 | 0.880 | 3.560 | LOC_Os02g12470 | hypothetical protein |
| Os.11707.1.A1_at | 4.141 | 0.143 | 1.445 | 0.298 | LOC_Os03g54130 | Papain family cysteine protease containing protein, expressed |
| Os.40021.1.S1_a_at | 3.860 | 0.249 | 0.500 | 0.259 | LOC_Os03g06630 | Heat shock factor protein 1, putative, expressed |
| Os.47946.1.S1_s_at | 3.860 | 1.931 | 0.914 | 1.462 | LOC_Os07g33910 | Glucose-6-phosphate/phosphate translocator 2, chloroplast precursor, putative, expressed |
| OsAffx.21073.1.S1_at | 3.826 | 0.559 | 0.401 | 0.494 | LOC_Os07g02620 | NB-ARC domain containing protein |
| Os.9103.1.S1_at | 3.801 | 8.130 | 3.924 | 1.551 | LOC_Os09g09930 | heavy metal-associated domain containing protein, expressed |
| Os.50470.1.S1_at | 3.611 | 3.270 | 4.170 | 0.793 | LOC_Os01g71310 | Cytokinin dehydrogenase 1 precursor, putative, expressed |
| Os.34982.1.A1_at | 3.611 | 0.348 | 0.828 | 0.751 | LOC_Os04g17660 | Rhodanese-like domain containing protein, expressed |
| OsAffx.24831.1.S1_s_at | 3.523 | 0.421 | 0.600 | 0.606 | LOC_Os02g52270 | Chaperone protein dnaJ, putative, expressed |

| ProbeSetID | *OMTN3* -OE[a] | Stress response[b] | | | TIGR Locus ID[c] | Annotation |
|---|---|---|---|---|---|---|
| | | Drought | Salt | Cold | | |
| Os.10556.1.S1_at | 3.495 | 8.593 | 9.683 | 0.495 | LOC_Os02g15860 | expressed protein |
| Os.8481.2.S1_at | 3.480 | 2.081 | 2.120 | 1.173 | LOC_Os03g13450 | expressed protein |
| Os.6269.1.S1_at | 3.416 | 1.635 | 0.880 | 0.676 | LOC_Os03g19090 | expressed protein |
| Os.22485.1.A1_at | 3.401 | 0.273 | 0.324 | 1.008 | LOC_Os11g29720 | Cytochrome P450 family protein, expressed |
| Os.35463.1.S1_at | 3.358 | 8.504 | 4.835 | 0.815 | LOC_Os05g48810 | DnaJ domain containing protein, expressed |
| Os.8045.1.S1_at | 3.337 | 0.138 | 0.936 | 0.922 | LOC_Os01g08440 | Indole-3-acetate beta-glucosyltransferase, putative, expressed |
| Os.49627.1.S1_at | 3.166 | 1.651 | 2.649 | 0.534 | LOC_Os06g37150 | L-ascorbate oxidase, putative, expressed |
| Os.17916.1.S1_at | 3.108 | 0.780 | 0.990 | 0.951 | LOC_Os05g12630 | expressed protein |
| Os.7664.1.S1_at | 3.099 | 6.372 | 3.011 | 0.925 | LOC_Os02g56900 | expressed protein |
| Os.8868.1.S1_at | 3.083 | 1.110 | 1.678 | 0.118 | LOC_Os10g38610 | glutathione S-transferase GSTU6, putative, expressed |
| OsAffx.26826.1.S1_at | 3.070 | 0.062 | 0.152 | 1.096 | LOC_Os05g11210 | expressed protein |
| Os.9191.1.S1_s_at | 3.016 | 2.661 | 1.507 | 0.139 | LOC_Os01g14410 | Early light-induced protein, chloroplast precursor, putative, expressed |
| Os.54440.1.S1_at | 2.970 | 0.392 | 0.420 | 0.987 | LOC_Os04g53180 | expressed protein |
| OsAffx.31235.1.S1_at | 2.942 | 1.047 | 0.567 | 1.150 | LOC_Os11g35300 | hypothetical protein |
| OsAffx.12508.1.S1_at | 2.920 | 0.201 | 0.377 | 0.374 | LOC_Os02g45520 | SPX domain-containing protein, putative, expressed |
| OsAffx.24280.2.S1_at | 2.898 | 0.496 | 0.821 | 0.379 | LOC_Os07g08150 | early light-inducible protein ELIP, putative, expressed |
| OsAffx.25073.1.S1_x_at | 2.840 | 0.202 | 0.607 | 0.286 | LOC_Os03g12700 | expressed protein |
| Os.51066.1.S1_at | 2.816 | 0.531 | 1.428 | 1.117 | LOC_Os06g43320 | Cytochrome P450 family protein, expressed |
| OsAffx.14605.1.S1_at | 2.793 | 1.501 | 1.918 | 3.028 | LOC_Os05g09020 | WRKY transcription factor 50, putative, expressed |
| Os.57569.2.A1_s_at | 2.790 | 0.468 | 0.141 | 0.478 | LOC_Os08g04500 | Terpene synthase family, metal binding domain containing protein, expressed |
| Os.49931.1.S1_at | 2.788 | 1.301 | 0.939 | 1.679 | LOC_Os11g43250 | Leucine Rich Repeat family protein, expressed |
| OsAffx.27093.1.S1_at | 2.732 | 0.077 | 0.346 | 0.267 | LOC_Os05g31040 | Cytokinin dehydrogenase 1 precursor, putative, expressed |
| Os.35061.1.S1_at | 2.727 | 0.708 | 0.354 | 1.393 | LOC_Os01g42690 | Phospholipase/Carboxylesterase family protein, expressed |
| Os.52331.1.A1_at | 2.706 | 1.126 | 0.867 | 1.000 | LOC_Os07g49390 | prephenate dehydratase family protein, expressed |

| ProbeSetID | *OMTN3* -OE[a] | Stress response[b] | | | TIGR Locus ID[c] | Annotation |
|---|---|---|---|---|---|---|
| | | Drought | Salt | Cold | | |
| Os.11804.1.S1_at | 2.694 | 0.237 | 0.393 | 0.215 | LOC_Os03g14910 | Tryptophan/tyrosine permease family protein, expressed |
| Os.8118.1.S1_at | 2.687 | 1.059 | 1.799 | 0.592 | LOC_Os07g26630 | Aquaporin PIP2.2, putative, expressed |
| Os.49648.1.S1_s_at | 2.618 | 21.581 | 20.046 | 0.419 | LOC_Os02g54140 | Hsp20/alpha crystallin family protein, expressed |
| Os.9212.1.S1_at | 2.611 | 0.565 | 0.756 | 0.722 | LOC_Os07g22930 | Granule-bound starch synthase 1b, chloroplast precursor, putative, expressed |
| Os.4801.1.S1_x_at | 2.601 | 0.793 | 0.818 | 0.881 | LOC_Os01g52010 | Allinase, C-terminal domain containing protein, expressed |
| Os.51026.1.S1_at | 2.580 | 0.598 | 0.655 | 0.897 | LOC_Os07g30620 | UDP-glucoronosyl and UDP-glucosyl transferase family protein, expressed |
| Os.10126.1.S1_at | 2.575 | 1.860 | 1.927 | 3.544 | LOC_Os09g24580 | EF hand family protein, expressed |
| Os.4775.1.S1_at | 2.537 | 1.187 | 2.645 | 2.658 | LOC_Os01g04380 | 16.9 kDa class I heat shock protein, putative, expressed |
| Os.5047.1.S1_at | 2.531 | 1.232 | 1.265 | 0.679 | LOC_Os07g08830 | expressed protein |
| Os.11771.1.S1_at | 2.521 | 0.479 | 0.505 | 0.468 | LOC_Os06g12320 | Transmembrane amino acid transporter protein, expressed |
| Os.15908.1.S1_s_at | 2.511 | 1.366 | 0.755 | 0.689 | LOC_Os01g72330 | Two-component response regulator ARR8, putative, expressed |
| Os.37295.1.S1_at | 2.503 | 0.472 | 0.450 | 0.857 | LOC_Os11g02440 | Chalcone-flavanone isomerase family protein, expressed |
| Os.34459.1.S1_at | 2.486 | 0.305 | 0.467 | 0.426 | LOC_Os10g28360 | 1,2-dihydroxy-3-keto-5-methylthiopentene dioxygenase, putative, expressed |
| Os.46725.1.S1_at | 2.486 | 0.888 | 0.634 | 0.322 | LOC_Os10g05130 | expressed protein |
| Os.9107.2.S1_x_at | 2.479 | 1.974 | 4.485 | 0.908 | LOC_Os09g27260 | Plant viral-response family protein, expressed |
| Os.54927.1.S1_at | 2.468 | 1.374 | 1.922 | 0.922 | LOC_Os08g44590 | oxidoreductase, 2OG-Fe oxygenase family protein, expressed |
| Os.12705.2.S1_x_at | 2.457 | 3.074 | 2.942 | 0.743 | LOC_Os03g15890 | RNA recognition motif family protein, expressed |
| Os.1385.1.S1_at | 2.448 | 1.305 | 1.446 | 1.301 | LOC_Os01g71670 | Glucan endo-1,3-beta-glucosidase GII precursor, putative, expressed |
| Os.16422.1.S1_s_at | 2.435 | 3.893 | 4.204 | 0.541 | LOC_Os08g15050 | CCT motif family protein, expressed |
| Os.27497.1.S1_at | 2.432 | 0.191 | 0.121 | 0.681 | LOC_Os12g12390 | transposon protein, putative, CACTA, En/Spm sub-class, expressed |
| Os.50590.2.A1_at | 2.425 | 0.307 | 0.696 | 1.025 | LOC_Os02g07830 | Cation transport protein, expressed |
| Os.7831.1.S1_at | 2.389 | 6.984 | 3.402 | 0.823 | LOC_Os06g11980 | expressed protein |
| Os.5817.1.S1_at | 2.375 | 17.361 | 10.204 | 0.986 | LOC_Os06g09560 | DnaJ domain containing protein, expressed |
| Os.7991.1.S1_at | 2.373 | 3.486 | 1.811 | 1.730 | LOC_Os04g41620 | Endochitinase A precursor, putative, expressed |

| ProbeSetID | *OMTN3* -OE[a] | Stress response[b] | | | TIGR Locus ID[c] | Annotation |
|---|---|---|---|---|---|---|
| | | Drought | Salt | Cold | | |
| Os.30866.1.S1_at | 2.355 | 6.833 | 5.345 | 2.740 | LOC_Os07g02460 | expressed protein |
| Os.11040.1.S1_x_at | 2.351 | 2.689 | 2.236 | 0.960 | LOC_Os10g31320 | transposon protein, putative, CACTA, En/Spm sub-class, expressed |
| Os.12693.3.S1_at | 2.347 | 3.632 | 3.838 | 1.825 | LOC_Os07g49400 | L-ascorbate peroxidase 2, cytosolic, putative, expressed |
| Os.10280.1.A1_at | 2.329 | 0.388 | 0.593 | 0.474 | LOC_Os02g04510 | NmrA-like family protein |
| OsAffx.32321.1.A1_at | 2.318 | 0.765 | 0.247 | 1.747 | LOC_Os06g16390 | Polycomb protein EZ1, putative, expressed |
| Os.50015.1.S1_at | 2.311 | 5.523 | 21.830 | 5.004 | LOC_Os06g44010 | WRKY2 protein, putative, expressed |
| OsAffx.12542.1.S1_at | 2.310 | 0.286 | 0.400 | 0.571 | LOC_Os02g47920 | exonuclease family protein |
| Os.53403.1.S1_at | 2.303 | 0.628 | 1.307 | 1.300 | LOC_Os08g39830 | ETHYLENE-INSENSITIVE3-like 3 protein, putative, expressed |
| Os.31191.1.S1_at | 2.297 | 0.592 | 1.470 | 0.685 | LOC_Os07g26660 | expressed protein |
| Os.35808.1.S1_at | 2.282 | 9.547 | 3.532 | 6.845 | LOC_Os01g42190 | DnaJ domain containing protein, expressed |
| OsAffx.24138.1.S1_s_at | 2.275 | 9.413 | 7.076 | 1.195 | LOC_Os02g06710 | expressed protein |
| Os.11573.2.A2_a_at | 2.256 | 0.613 | 0.881 | 0.935 | LOC_Os11g47970 | Ribulose bisphosphate carboxylase/oxygenase activase, chloroplast precursor, putative, expressed |
| Os.7772.2.A1_at | 2.255 | 1.064 | 0.946 | 1.205 | LOC_Os06g08840 | RNA recognition motif family protein, expressed |
| Os.33102.1.S1_at | 2.252 | 2.600 | 28.282 | 2.553 | LOC_Os01g43740 | Cytochrome P450 family protein, expressed |
| Os.15142.1.S1_at | 2.247 | 1.031 | 1.591 | 0.992 | LOC_Os04g49350 | pentatricopeptide, putative, expressed |
| Os.32123.1.S1_at | 2.240 | 1.901 | 2.949 | 0.648 | LOC_Os01g02930 | glycosyltransferase, putative, expressed |
| Os.12323.2.S1_at | 2.220 | 0.491 | 0.451 | 0.621 | LOC_Os04g49650 | expressed protein |
| Os.7665.1.S1_at | 2.201 | 0.086 | 0.470 | 0.307 | LOC_Os04g56240 | Lipase family protein, expressed |
| Os.12112.1.S1_at | 2.198 | 0.299 | 0.617 | 0.152 | LOC_Os01g60830 | expressed protein |
| Os.32177.1.S1_at | 2.196 | 1.371 | 1.276 | 0.777 | LOC_Os05g50930 | Sigma-70, region 4 family protein, expressed |
| Os.47598.1.S1_at | 2.168 | 0.862 | 0.703 | 1.082 | LOC_Os01g12190 | expressed protein |
| Os.37969.1.S1_at | 2.159 | 1.573 | 0.171 | 4.067 | LOC_Os01g66860 | ankyrin-kinase, putative, expressed |
| Os.8922.1.S1_at | 2.153 | 0.399 | 0.608 | 0.596 | LOC_Os04g05010 | CBS domain containing protein, expressed |

| ProbeSetID | *OMTN3* -OE[a] | Stress response[b] | | | TIGR Locus ID[c] | Annotation |
|---|---|---|---|---|---|---|
| | | Drought | Salt | Cold | | |
| Os.7475.1.S1_at | 2.147 | 3.277 | 2.881 | 0.486 | LOC_Os01g74450 | Aquaporin TIP1.1, putative, expressed |
| Os.27393.1.S1_a_at | 2.144 | 0.428 | 0.510 | 0.502 | LOC_Os05g04610 | ABC transporter family protein, expressed |
| Os.30528.1.S1_at | 2.142 | 52.308 | 18.077 | 0.923 | LOC_Os08g31860 | expressed protein |
| Os.6372.1.S1_at | 2.141 | 18.720 | 1.593 | 1.071 | LOC_Os03g10110 | Cupin family protein, expressed |
| Os.53074.1.S1_at | 2.137 | 1.341 | 5.349 | 8.467 | LOC_Os03g20330 | VQ motif family protein, expressed |
| Os.27080.1.S1_at | 2.128 | 0.643 | 1.341 | 0.567 | LOC_Os02g36450 | Sugar transporter family protein, expressed |
| OsAffx.3569.1.S1_at | 2.125 | 2.547 | 2.755 | 0.886 | LOC_Os03g50960 | Protease inhibitor/seed storage/LTP family protein, expressed |
| Os.8468.1.S1_at | 2.117 | 0.121 | 0.550 | 1.021 | LOC_Os12g25180 | expressed protein |
| Os.12253.1.S1_at | 2.109 | 3.418 | 2.225 | 2.280 | LOC_Os01g45110 | UDP-glucoronosyl and UDP-glucosyl transferase family protein, expressed |
| OsAffx.5196.1.S1_at | 2.102 | 0.449 | 0.764 | 0.584 | LOC_Os07g03900 | Lectin receptor-type protein kinase, putative |
| Os.9880.1.S1_a_at | 2.088 | 0.278 | 0.623 | 0.507 | LOC_Os01g68480 | Thioredoxin F-type 2, chloroplast precursor, putative, expressed |
| Os.24911.1.S1_at | 2.081 | 0.450 | 0.449 | 0.540 | LOC_Os03g62170 | Streptomyces cyclase/dehydrase family protein, expressed |
| Os.22651.1.S1_at | 2.081 | 0.125 | 0.288 | 0.280 | LOC_Os04g56230 | Farnesyl pyrophosphate synthetase 2, putative, expressed |
| Os.3710.1.S1_at | 2.077 | 1.230 | 1.677 | 5.611 | LOC_Os02g35440 | E3 ubiquitin ligase EL5, putative |
| Os.12550.1.S1_s_at | 2.075 | 1.038 | 2.351 | 0.614 | LOC_Os02g09510 | UDP-glucoronosyl and UDP-glucosyl transferase family protein, expressed |
| Os.34438.1.S1_x_at | 2.071 | 54.833 | 9.800 | 2.383 | LOC_Os07g39740 | Esterase precursor, putative, expressed |
| Os.28290.1.S1_at | 2.064 | 0.096 | 0.405 | 0.223 | LOC_Os02g47510 | nine-cis-epoxycarotenoid dioxygenase4, putative, expressed |
| Os.49826.1.S1_at | 2.064 | 1.949 | 2.900 | 1.073 | LOC_Os04g31790 | expressed protein |
| Os.53622.1.S1_at | 2.056 | 1.780 | 4.531 | 1.627 | LOC_Os04g44100 | expressed protein |
| Os.19523.1.S1_at | 2.045 | 0.331 | 0.682 | 0.364 | LOC_Os03g51930 | expressed protein |
| Os.45887.1.S1_at | 2.045 | 2.906 | 1.462 | 0.688 | LOC_Os01g12750 | Cytochrome P450 family protein, expressed |
| OsAffx.19309.1.S1_at | 2.032 | 0.405 | 0.750 | 0.712 | LOC_Os11g41500 | NC domain-containing protein, putative |
| Os.53004.1.S1_at | 2.024 | 0.868 | 0.253 | 2.011 | LOC_Os12g12560 | NADP-dependent oxidoreductase P2, putative, expressed |
| OsAffx.13348.1.S1_s_at | 2.021 | 0.346 | 0.894 | 0.598 | LOC_Os03g44740 | Cytochrome P450 family protein, expressed |

| ProbeSetID | *OMTN3* -OE[a] | Stress response[b] | | | TIGR Locus ID[c] | Annotation |
|---|---|---|---|---|---|---|
| | | Drought | Salt | Cold | | |
| Os.11029.1.S1_at | 2.018 | 3.039 | 2.038 | 0.923 | LOC_Os03g16860 | Heat shock cognate 70 kDa protein, putative, expressed |
| Os.8480.1.S1_at | 2.015 | 0.243 | 0.186 | 2.271 | LOC_Os05g30700 | expressed protein |
| Os.50973.1.S1_at | 2.008 | 1.182 | 0.947 | 0.954 | LOC_Os02g20040 | transposon protein, putative, CACTA, En/Spm sub-class, expressed |
| Os.7075.1.S1_at | 2.004 | 0.921 | 1.268 | 0.897 | LOC_Os03g27230 | Phospho-2-dehydro-3-deoxyheptonate aldolase 1, chloroplast precursor, putative, expressed |
| Os.2371.1.S1_at | 0.082 | 2.412 | 1.820 | 1.596 | LOC_Os12g26290 | alpha-dioxygenase, putative, expressed |
| OsAffx.11788.1.S1_at | 0.089 | 0.376 | 0.313 | 0.649 | LOC_Os01g68460 | DC1 domain-containing protein, putative, expressed |
| Os.49634.1.S1_x_at | 0.131 | 2.255 | 3.515 | 5.648 | LOC_Os01g55510 | Dynein light chain type 1 family protein, expressed |
| Os.12452.1.S1_s_at | 0.145 | 7.189 | 4.123 | 1.011 | LOC_Os08g36910 | Alpha-amylase isozyme 3D precursor, putative, expressed |
| OsAffx.15812.1.S1_at | 0.148 | 1.455 | 1.000 | 1.182 | LOC_Os06g38120 | expressed protein |
| OsAffx.26389.1.S1_at | 0.153 | 0.619 | 0.619 | 0.952 | LOC_Os04g39300 | heavy metal-associated domain containing protein, expressed |
| Os.49322.1.S1_at | 0.166 | 0.511 | 0.660 | 2.426 | LOC_Os09g15050 | Ent-kaurene synthase A, chloroplast precursor, putative, expressed |
| Os.17918.1.S1_at | 0.171 | 0.172 | 1.654 | 1.158 | LOC_Os03g16950 | 33 kDa secretory protein, putative, expressed |
| Os.5335.1.S1_at | 0.180 | 11.598 | 2.485 | 17.068 | LOC_Os04g49450 | myb-like DNA-binding domain, SHAQKYF class family protein, expressed |
| Os.54961.1.S1_at | 0.182 | 5.200 | 1.663 | 2.109 | LOC_Os05g46350 | IQ calmodulin-binding motif family protein, expressed |
| Os.18463.1.S1_at | 0.183 | 9.807 | 2.097 | 5.040 | LOC_Os05g37660 | AER, putative, expressed |
| Os.32455.1.S1_at | 0.184 | 1.276 | 0.980 | 1.156 | LOC_Os01g54670 | expressed protein |
| Os.36283.1.S1_at | 0.187 | 1.166 | 1.408 | 0.410 | LOC_Os12g25090 | expressed protein |
| OsAffx.17348.1.S1_at | 0.197 | 0.699 | 0.928 | 2.325 | LOC_Os08g35590 | hypothetical protein |
| Os.27494.1.S1_at | 0.201 | 0.474 | 0.665 | 1.051 | LOC_Os07g41200 | expressed protein |
| Os.27019.1.S1_at | 0.201 | 3.466 | 2.619 | 0.967 | LOC_Os04g43410 | Glycosyl hydrolase family 1 protein, expressed |
| Os.12201.2.S1_at | 0.208 | 2.702 | 2.693 | 0.977 | LOC_Os09g27820 | 1-aminocyclopropane-1-carboxylate oxidase 1, putative, expressed |
| OsAffx.14201.1.S1_at | 0.209 | 0.337 | 0.136 | 0.472 | LOC_Os04g39360 | heavy metal-associated domain containing protein, expressed |
| OsAffx.12733.1.S1_at | 0.212 | 1.472 | 1.239 | 3.033 | LOC_Os03g04740 | expressed protein |

| ProbeSetID | *OMTN3* -OE[a] | Stress response[b] | | | TIGR Locus ID[c] | Annotation |
|---|---|---|---|---|---|---|
| | | Drought | Salt | Cold | | |
| OsAffx.3920.1.S1_s_at | 0.212 | 0.214 | 1.854 | 1.961 | LOC_Os04g27670 | Terpene synthase family, metal binding domain containing protein, expressed |
| Os.27688.1.A1_at | 0.214 | 0.957 | 0.731 | 9.416 | LOC_Os04g21350 | flowering promoting factor-like 1, putative, expressed |
| OsAffx.24782.1.S1_s_at | 0.215 | 3.917 | 1.965 | 2.304 | LOC_Os02g48320 | DNA-binding protein, putative, expressed |
| Os.19472.1.S1_at | 0.215 | 7.458 | 5.282 | 0.656 | LOC_Os01g03330 | Bowman-Birk type bran trypsin inhibitor precursor, putative, expressed |
| Os.51616.1.S1_at | 0.220 | 0.629 | 0.748 | 0.841 | LOC_Os03g16600 | expressed protein |
| Os.2211.1.S1_at | 0.221 | 0.568 | 0.573 | 1.727 | LOC_Os08g09010 | Germin-like protein subfamily 1 member 11 precursor, putative, expressed |
| Os.2321.1.S1_at | 0.226 | 0.659 | 0.543 | 1.943 | LOC_Os08g09060 | Germin-like protein subfamily 1 member 11 precursor, putative, expressed |
| OsAffx.7606.1.S1_at | 0.227 | 1.419 | 4.132 | 1.940 | LOC_Os12g15680 | Multicopper oxidase family protein, expressed |
| Os.50572.1.S1_at | 0.234 | 1.092 | 0.941 | 1.701 | LOC_Os12g36920 | calmodulin-binding protein, putative, expressed |
| OsAffx.23641.1.S1_at | 0.236 | 0.162 | 0.413 | 3.379 | LOC_Os01g43230 | expressed protein |
| Os.27143.1.S1_at | 0.236 | 0.755 | 1.183 | 0.928 | LOC_Os07g48060 | Cationic peroxidase 1 precursor, putative, expressed |
| Os.15701.1.S1_x_at | 0.239 | 0.893 | 1.201 | 1.459 | LOC_Os07g47350 | Potassium transporter 7, putative, expressed |
| Os.28301.1.S1_at | 0.242 | 0.225 | 0.520 | 1.377 | LOC_Os07g05360 | Photosystem II 10 kDa polypeptide, chloroplast precursor, putative, expressed |
| Os.52661.1.S1_at | 0.245 | 1.003 | 0.490 | 3.697 | LOC_Os11g44680 | expressed protein |
| Os.53407.1.S1_at | 0.246 | 3.573 | 1.288 | 0.763 | LOC_Os08g06210 | expressed protein |
| Os.2320.1.S1_at | 0.247 | 0.629 | 0.602 | 1.514 | LOC_Os08g09080 | Germin-like protein subfamily 1 member 11 precursor, putative, expressed |
| Os.54944.1.S1_at | 0.247 | 28.688 | 19.029 | 10.857 | LOC_Os02g52670 | AP2 domain containing protein, expressed |
| Os.54453.1.S1_at | 0.257 | 0.375 | 0.219 | 1.231 | LOC_Os08g44220 | expressed protein |
| Os.14413.1.S1_at | 0.264 | 0.348 | 0.366 | 1.939 | LOC_Os07g48340 | Calcium-binding protein CAST, putative, expressed |
| Os.15706.1.S1_a_at | 0.266 | 1.408 | 0.692 | 1.753 | LOC_Os01g47580 | Lipid phosphate phosphatase 2, putative, expressed |
| OsAffx.19473.1.S1_at | 0.267 | 1.539 | 1.309 | 1.893 | LOC_Os12g03740 | F-box domain containing protein, expressed |
| Os.27011.1.A1_s_at | 0.268 | 0.551 | 0.802 | 1.551 | LOC_Os01g04550 | serine/threonine protein kinase, putative, expressed |
| Os.17887.1.S1_at | 0.269 | 3.302 | 1.690 | 4.523 | LOC_Os12g36910 | calmodulin-binding protein, putative, expressed |
| OsAffx.31475.1.S1_at | 0.271 | 0.927 | 1.232 | 1.277 | LOC_Os11g44630 | hypothetical protein |

| ProbeSetID | *OMTN3* -OE[a] | Stress response[b] | | | TIGR Locus ID[c] | Annotation |
|---|---|---|---|---|---|---|
| | | Drought | Salt | Cold | | |
| Os.50449.1.S1_at | 0.271 | 2.270 | 0.885 | 1.655 | LOC_Os03g10640 | Calcium-transporting ATPase 2, plasma membrane-type, putative, expressed |
| Os.10675.1.A1_at | 0.271 | 0.734 | 1.709 | 1.969 | LOC_Os01g68140 | expressed protein |
| Os.11287.1.S1_at | 0.272 | 3.770 | 2.584 | 1.577 | LOC_Os06g50930 | Senescence-associated protein DIN1, putative, expressed |
| OsAffx.7506.1.S1_at | 0.275 | 1.073 | 0.938 | 1.741 | LOC_Os12g06780 | expressed protein |
| OsAffx.23406.1.S1_at | 0.276 | 0.836 | 0.954 | 1.289 | LOC_Os01g26300 | wall-associated kinase 1, putative, expressed |
| Os.27705.1.S1_at | 0.278 | 1.884 | 1.927 | 1.096 | LOC_Os01g18290 | Helix-loop-helix DNA-binding domain containing protein, expressed |
| Os.57456.1.S1_x_at | 0.278 | 2.707 | 2.378 | 1.608 | LOC_Os01g24710 | Salt stress-induced protein, putative, expressed |
| Os.30000.1.S1_at | 0.286 | 10.395 | 5.383 | 0.794 | LOC_Os07g36560 | Transferase family protein, expressed |
| Os.30909.1.S1_at | 0.288 | 0.504 | 1.065 | 0.187 | LOC_Os01g09190 | expressed protein |
| Os.46840.1.S1_x_at | 0.288 | 0.338 | 0.425 | 0.884 | LOC_Os10g34700 | expressed protein |
| Os.26511.1.S1_at | 0.289 | 2.808 | 2.127 | 5.175 | LOC_Os04g49370 | expressed protein |
| Os.46618.1.S1_at | 0.289 | 29.232 | 12.826 | 6.346 | LOC_Os10g41550 | Glycosyl hydrolase family 14 protein, expressed |
| Os.37603.1.S1_at | 0.291 | 1.837 | 4.689 | 1.265 | LOC_Os01g63480 | AER, putative, expressed |
| Os.19861.1.S1_at | 0.291 | 0.697 | 1.294 | 1.731 | LOC_Os07g03730 | Pathogenesis-related protein 1 precursor, putative, expressed |
| Os.6345.1.S1_at | 0.292 | 0.701 | 0.583 | 1.223 | LOC_Os06g36560 | Inositol oxygenase, putative, expressed |
| OsAffx.22469.1.S1_x_at | 0.295 | 3.843 | 2.093 | 2.284 | LOC_Os07g07040 | expressed protein |
| Os.50825.1.S1_s_at | 0.296 | 1.097 | 0.433 | 0.270 | LOC_Os05g39990 | Alpha-expansin 1 precursor, putative, expressed |
| Os.55408.1.S1_at | 0.297 | 88.632 | 13.552 | 9.601 | LOC_Os04g52750 | expressed protein |
| Os.27507.1.S1_at | 0.299 | 0.899 | 3.222 | 1.949 | LOC_Os06g35700 | Reticuline oxidase precursor, putative, expressed |
| Os.54385.1.S1_at | 0.299 | 0.889 | 0.715 | 1.847 | LOC_Os04g01320 | D-mannose binding lectin family protein, expressed |
| Os.17900.1.S1_s_at | 0.300 | 0.738 | 0.940 | 0.174 | LOC_Os05g35690 | Gibberellin-regulated protein 2 precursor, putative, expressed |
| Os.11354.1.S1_at | 0.302 | 1.361 | 0.188 | 7.684 | LOC_Os04g51460 | xyloglucan endotransglucosylase/hydrolase protein 15precursor, putative, expressed |
| Os.33316.1.S1_at | 0.302 | 3.624 | 0.960 | 1.115 | LOC_Os01g34560 | very-long-chain fatty acid condensing enzyme, putative, expressed |

| ProbeSetID | *OMTN3* -OE[a] | Stress response[b] | | | TIGR Locus ID[c] | Annotation |
|---|---|---|---|---|---|---|
| | | Drought | Salt | Cold | | |
| OsAffx.2919.1.S1_at | 0.303 | 1.784 | 2.892 | 3.162 | LOC_Os02g39660 | Leucine Rich Repeat family protein, expressed |
| Os.52228.1.S1_at | 0.303 | 0.224 | 0.400 | 0.903 | LOC_Os11g36000 | Leucine Rich Repeat family protein, expressed |
| Os.57191.1.S1_at | 0.305 | 16.608 | 9.364 | 4.114 | LOC_Os06g05470 | expressed protein |
| Os.31858.1.S1_at | 0.305 | 4.131 | 1.885 | 2.555 | LOC_Os07g35340 | Protein kinase domain containing protein, expressed |
| OsAffx.3275.1.S1_at | 0.306 | 2.265 | 1.807 | 1.867 | LOC_Os03g19400 | expressed protein |
| Os.11786.2.S1_at | 0.307 | 7.064 | 5.694 | 17.799 | LOC_Os01g15900 | Dof domain, zinc finger family protein, expressed |
| Os.15633.1.S1_at | 0.308 | 4.191 | 1.939 | 1.984 | LOC_Os10g35460 | COBRA-like protein 2 precursor, putative, expressed |
| Os.20206.1.S1_at | 0.311 | 24.261 | 12.171 | 3.913 | LOC_Os02g41840 | expressed protein |
| Os.21319.1.S1_at | 0.311 | 3.877 | 4.500 | 4.794 | LOC_Os09g06770 | Zinc finger, C3HC4 type family protein, expressed |
| Os.7727.1.S1_at | 0.311 | 4.679 | 3.170 | 0.994 | LOC_Os05g11910 | GDSL-like Lipase/Acylhydrolase family protein, expressed |
| Os.37773.1.S1_at | 0.312 | 1.643 | 7.595 | 2.434 | LOC_Os03g16030 | 17.4 kDa class I heat shock protein, putative, expressed |
| OsAffx.27743.1.S1_at | 0.312 | 0.219 | 0.094 | 3.413 | LOC_Os06g20900 | hypothetical protein |
| OsAffx.11789.1.S1_s_at | 0.313 | 0.092 | 0.083 | 0.712 | LOC_Os01g68470 | hypothetical protein |
| Os.53009.1.A1_x_at | 0.317 | 0.078 | 0.444 | 0.883 | LOC_Os02g54640 | Receptor family ligand binding region containing protein, expressed |
| Os.11309.1.S1_x_at | 0.317 | 1.181 | 0.961 | 0.901 | LOC_Os05g04490 | Peroxidase family protein, expressed |
| OsAffx.24613.1.S1_at | 0.321 | 0.550 | 1.750 | 0.500 | LOC_Os02g37320 | heavy metal-associated domain containing protein, expressed |
| OsAffx.14345.1.S1_at | 0.322 | 0.808 | 0.636 | 1.190 | LOC_Os04g49490 | protein phosphatase 2C family protein, putative, expressed |
| Os.5905.1.S1_at | 0.325 | 0.897 | 0.223 | 0.877 | LOC_Os06g32990 | Peroxidase family protein, expressed |
| Os.7938.1.S1_at | 0.328 | 3.543 | 1.221 | 0.757 | LOC_Os04g59190 | Peroxidase family protein, expressed |
| OsAffx.17220.1.S1_at | 0.328 | 3.300 | 3.450 | 0.550 | LOC_Os08g29040 | Protein kinase domain containing protein |
| Os.7678.1.S1_at | 0.329 | 2.222 | 2.997 | 6.049 | LOC_Os03g12500 | Cytochrome P450 74A2, putative, expressed |
| Os.27793.1.S1_at | 0.330 | 0.583 | 0.764 | 0.556 | LOC_Os02g14430 | Peroxidase 52 precursor, putative, expressed |
| Os.1978.1.S1_at | 0.332 | 1.649 | 3.001 | 1.492 | LOC_Os01g02290 | Ser/Thr receptor-like kinase, putative, expressed |
| OsAffx.24726.1.S1_s_at | 0.332 | 0.405 | 0.882 | 3.131 | LOC_Os02g44710 | expressed protein |

| ProbeSetID | *OMTN3* -OE[a] | Stress response[b] | | | TIGR Locus ID[c] | Annotation |
|---|---|---|---|---|---|---|
| | | Drought | Salt | Cold | | |
| Os.32801.1.S1_x_at | 0.333 | 1.697 | 0.949 | 2.881 | LOC_Os01g54700 | transposon protein, putative, CACTA, En/Spm sub-class, expressed |
| OsAffx.12721.1.S1_at | 0.333 | 2.570 | 1.367 | 1.457 | LOC_Os03g03510 | CIPK-like protein 1, putative, expressed |
| Os.12234.1.S1_s_at | 0.335 | 0.784 | 0.340 | 0.481 | LOC_Os10g40730 | Beta-expansin 1a precursor, putative, expressed |
| Os.53887.2.S1_x_at | 0.338 | 6.939 | 1.310 | 1.432 | LOC_Os07g49480 | expressed protein |
| OsAffx.6035.1.S1_at | 0.340 | 6.939 | 1.310 | 1.432 | LOC_Os08g35580 | expressed protein |
| Os.20482.1.S1_at | 0.340 | 0.633 | 0.627 | 1.841 | LOC_Os03g02550 | F-box family protein, putative, expressed |
| Os.26810.1.A1_s_at | 0.343 | 0.333 | 0.619 | 1.059 | LOC_Os02g16940 | Subtilisin N-terminal Region family protein, expressed |
| Os.6042.1.S1_at | 0.343 | 4.173 | 1.419 | 0.716 | LOC_Os07g25810 | transposon protein, putative, CACTA, En/Spm sub-class, expressed |
| Os.9154.1.S1_at | 0.344 | 2.221 | 2.133 | 2.876 | LOC_Os01g56220 | expressed protein |
| Os.11322.1.S1_at | 0.345 | 1.168 | 0.685 | 1.311 | LOC_Os04g50950 | POT family protein, expressed |
| Os.38856.1.A1_s_at | 0.346 | 2.874 | 0.619 | 1.877 | LOC_Os01g67950 | ubiquitin family protein, putative, expressed |
| Os.10635.1.S1_s_at | 0.346 | 0.833 | 1.438 | 1.710 | LOC_Os04g51880 | GHMP kinases putative ATP-binding protein, expressed |
| Os.5213.1.S1_at | 0.348 | 0.213 | 0.432 | 6.455 | LOC_Os02g44720 | expressed protein |
| Os.5594.1.S1_at | 0.348 | 3.823 | 2.911 | 1.173 | LOC_Os05g48340 | proteasome, putative, expressed |
| Os.4453.1.S1_at | 0.349 | 0.276 | 0.891 | 0.682 | LOC_Os06g03520 | expressed protein |
| Os.28200.1.S1_x_at | 0.349 | 24.253 | 27.402 | 8.330 | LOC_Os03g61160 | expressed protein |
| OsAffx.27815.1.S1_s_at | 0.350 | 6.433 | 5.255 | 1.739 | LOC_Os06g24990 | Xylanase inhibitor protein 1 precursor, putative, expressed |
| Os.52699.1.S1_at | 0.352 | 0.928 | 0.737 | 1.267 | LOC_Os04g55420 | Leucine Rich Repeat family protein, expressed |
| Os.25316.2.S1_x_at | 0.352 | 1.367 | 1.161 | 0.853 | LOC_Os03g56260 | Kinesin motor domain containing protein, expressed |
| Os.28030.1.S1_s_at | 0.353 | 0.920 | 1.569 | 2.386 | LOC_Os06g48160 | xyloglucan endotransglucosylase/hydrolase protein 15precursor, putative, expressed |
| Os.25124.1.A1_at | 0.354 | 4.082 | 3.733 | 1.772 | LOC_Os12g36630 | fiber protein Fb19, putative, expressed |
| Os.23327.2.S1_a_at | 0.354 | 0.291 | 0.602 | 2.617 | LOC_Os10g10130 | Calcium binding EGF domain containing protein, expressed |
| Os.11552.1.S2_at | 0.355 | 1.042 | 1.049 | 0.653 | LOC_Os04g59150 | Peroxidase 12 precursor, putative, expressed |

| ProbeSetID | *OMTN3* -OE[a] | Stress response[b] | | | TIGR Locus ID[c] | Annotation |
|---|---|---|---|---|---|---|
| | | Drought | Salt | Cold | | |
| OsAffx.7414.1.S1_at | 0.357 | 0.129 | 0.259 | 0.345 | LOC_Os11g44700 | expressed protein |
| Os.50575.1.S1_at | 0.358 | 2.252 | 1.239 | 1.780 | LOC_Os04g40700 | expressed protein |
| Os.30376.1.S1_at | 0.358 | 1.004 | 1.603 | 1.411 | LOC_Os01g02130 | expressed protein |
| Os.37213.1.S1_at | 0.358 | 0.469 | 0.502 | 1.640 | LOC_Os07g29310 | Auxin responsive protein, expressed |
| OsAffx.2891.1.S1_s_at | 0.360 | 0.822 | 1.243 | 2.211 | LOC_Os02g37330 | heavy metal-associated domain containing protein, expressed |
| Os.12363.1.S1_at | 0.361 | 0.389 | 0.495 | 1.859 | LOC_Os04g58200 | Protochlorophyllide reductase A, chloroplast precursor, putative, expressed |
| Os.56988.1.S1_at | 0.361 | 1.338 | 0.632 | 1.368 | LOC_Os03g53730 | flavodoxin family protein, expressed |
| OsAffx.5120.1.S1_x_at | 0.361 | 1.279 | 0.721 | 0.410 | LOC_Os06g45960 | Cytochrome P450 family protein, expressed |
| Os.38309.1.S1_at | 0.362 | 2.361 | 2.725 | 3.040 | LOC_Os06g36390 | expressed protein |
| Os.46941.1.S1_s_at | 0.366 | 2.784 | 3.364 | 1.773 | LOC_Os06g48500 | expressed protein |
| Os.51029.1.S1_at | 0.366 | 3.780 | 5.736 | 1.363 | LOC_Os09g02770 | expressed protein |
| Os.5812.1.S1_at | 0.367 | 0.795 | 0.938 | 2.129 | LOC_Os03g20500 | F-box domain containing protein, expressed |
| OsAffx.29016.1.S1_at | 0.367 | 1.325 | 1.152 | 1.424 | LOC_Os08g03600 | magnesium transporter CorA, putative, expressed |
| Os.56815.1.S1_at | 0.367 | 0.212 | 0.297 | 1.331 | LOC_Os06g18960 | expressed protein |
| Os.9021.4.S1_at | 0.369 | 2.217 | 0.261 | 2.261 | LOC_Os11g08530 | expressed protein |
| Os.6075.1.S1_at | 0.371 | 3.281 | 1.502 | 1.893 | LOC_Os04g06520 | expressed protein |
| OsAffx.21092.2.S1_at | 0.371 | 0.326 | 0.874 | 0.407 | LOC_Os01g26310 | expressed protein |
| Os.9199.1.S1_at | 0.371 | 4.709 | 1.450 | 1.368 | LOC_Os06g50330 | senescence/dehydration-associated protein, putative, expressed |
| Os.50399.1.S1_at | 0.372 | 74.437 | 31.901 | 1.586 | LOC_Os06g05420 | expressed protein |
| OsAffx.13360.1.S1_at | 0.372 | 3.190 | 1.047 | 3.493 | LOC_Os03g45280 | Dehydrin family protein, expressed |
| Os.8202.1.S1_at | 0.372 | 2.181 | 1.704 | 1.680 | LOC_Os12g37560 | phospholipase C, putative, expressed |
| Os.17758.1.A1_at | 0.374 | 1.171 | 0.857 | 1.000 | LOC_Os08g15710 | expressed protein |
| Os.46627.1.S1_at | 0.374 | 2.347 | 1.432 | 1.300 | LOC_Os10g40640 | Glycogenin, putative, expressed |
| Os.9344.1.S1_x_at | 0.374 | 1.944 | 1.581 | 1.546 | LOC_Os03g53800 | Glycosyl hydrolase family 3 N terminal domain containing protein, expressed |

| ProbeSetID | *OMTN3* -OE[a] | Stress response[b] | | | TIGR Locus ID[c] | Annotation |
|---|---|---|---|---|---|---|
| | | Drought | Salt | Cold | | |
| Os.51601.1.S1_at | 0.375 | 0.185 | 0.595 | 1.823 | LOC_Os07g40300 | Zinc finger protein 7, putative, expressed |
| Os.10245.1.S1_at | 0.375 | 1.135 | 2.508 | 1.681 | LOC_Os04g37700 | expressed protein |
| OsAffx.14202.1.S1_at | 0.376 | 0.158 | 0.157 | 0.266 | LOC_Os04g39380 | expressed protein |
| Os.17479.1.S1_at | 0.376 | 0.854 | 0.526 | 1.884 | LOC_Os01g49320 | Acidic endochitinase precursor, putative, expressed |
| Os.54463.1.S1_at | 0.376 | 1.321 | 3.216 | 0.667 | LOC_Os05g48680 | expressed protein |
| Os.7539.1.S1_at | 0.377 | 1.086 | 0.825 | 1.218 | LOC_Os03g03320 | expressed protein |
| OsAffx.24812.1.S1_s_at | 0.377 | 1.044 | 4.093 | 0.044 | LOC_Os02g50460 | U-box domain containing protein, expressed |
| Os.49384.1.A1_at | 0.378 | 1.002 | 1.194 | 1.289 | LOC_Os02g53040 | Protein kinase domain containing protein, expressed |
| Os.47945.1.A1_at | 0.378 | 0.810 | 0.490 | 0.792 | LOC_Os06g37510 | senescence-associated protein, putative, expressed |
| OsAffx.18423.1.S1_at | 0.378 | 0.527 | 0.872 | 1.115 | LOC_Os10g27430 | expressed protein |
| Os.53683.1.S1_at | 0.378 | 3.210 | 1.390 | 1.691 | LOC_Os09g26160 | Receptor family ligand binding region containing protein, expressed |
| Os.55829.1.S1_at | 0.378 | 3.196 | 1.379 | 2.648 | LOC_Os05g30760 | hydrolase, alpha/beta fold family protein, expressed |
| Os.36767.1.S1_at | 0.379 | 4.575 | 2.087 | 2.917 | LOC_Os01g04330 | EF hand family protein, expressed |
| OsAffx.15455.1.S1_at | 0.380 | 0.387 | 0.219 | 1.190 | LOC_Os06g16040 | hypothetical protein |
| Os.27382.2.S1_at | 0.380 | 0.116 | 0.266 | 1.171 | LOC_Os02g02120 | wall-associated kinase 3, putative, expressed |
| Os.56018.1.S1_at | 0.380 | 3.679 | 1.363 | 3.818 | LOC_Os05g44060 | expressed protein |
| Os.11639.1.S1_at | 0.380 | 2.396 | 1.256 | 1.777 | LOC_Os06g05130 | Myristoyl-acyl carrier protein thioesterase, chloroplast precursor, putative, expressed |
| Os.19369.1.S1_at | 0.380 | 1.610 | 1.080 | 0.969 | LOC_Os12g05990 | No apical meristem protein, expressed |
| OsAffx.15485.1.S1_at | 0.383 | 0.729 | 0.585 | 1.585 | LOC_Os06g18900 | embryogenesis transmembrane protein, putative, expressed |
| OsAffx.31524.1.S1_at | 0.384 | 1.130 | 2.474 | 1.389 | LOC_Os11g47180 | Leucine Rich Repeat family protein, expressed |
| Os.12977.1.S1_at | 0.384 | 2.675 | 1.197 | 3.089 | LOC_Os06g19430 | expressed protein |
| Os.171.1.S1_at | 0.386 | 0.797 | 1.090 | 1.302 | LOC_Os01g47070 | Acidic endochitinase precursor, putative, expressed |
| Os.53148.1.S1_at | 0.387 | 1.119 | 0.648 | 2.101 | LOC_Os03g52720 | magnesium-dependent phosphatase-1 family protein, expressed |

| ProbeSetID | *OMTN3* -OE[a] | Stress response[b] | | | TIGR Locus ID[c] | Annotation |
|---|---|---|---|---|---|---|
| | | Drought | Salt | Cold | | |
| OsAffx.5948.1.S1_at | 0.387 | 0.981 | 0.858 | 1.317 | LOC_Os08g27170 | calmodulin-binding protein, putative |
| Os.22594.1.S1_at | 0.388 | 8.122 | 4.913 | 1.160 | LOC_Os01g03390 | Bowman-Birk type bran trypsin inhibitor precursor, putative, expressed |
| Os.49973.1.S1_at | 0.388 | 0.879 | 0.436 | 1.258 | LOC_Os03g20210 | Eukaryotic aspartyl protease family protein, expressed |
| Os.7612.1.S1_at | 0.388 | 1.746 | 1.710 | 2.558 | LOC_Os03g60840 | Bowman-Birk serine protease inhibitor family protein, expressed |
| Os.8957.1.S1_at | 0.388 | 8.261 | 2.533 | 2.136 | LOC_Os01g63060 | phosphatidic acid phosphatase, putative, expressed |
| OsAffx.10980.1.S1_at | 0.388 | 1.235 | 1.330 | 3.191 | LOC_Os01g09150 | hypothetical protein |
| Os.30572.1.S1_at | 0.389 | 1.216 | 0.811 | 0.634 | LOC_Os01g50610 | SAM dependent carboxyl methyltransferase family protein, expressed |
| Os.11657.1.S1_at | 0.390 | 4.788 | 3.002 | 2.751 | LOC_Os01g47760 | glutaredoxin family protein, putative, expressed |
| Os.17112.1.S1_at | 0.390 | 15.064 | 12.497 | 1.767 | LOC_Os03g18030 | oxidoreductase, 2OG-Fe oxygenase family protein, expressed |
| Os.35433.1.S1_at | 0.390 | 0.402 | 0.165 | 14.371 | LOC_Os04g39320 | expressed protein |
| Os.7862.1.S1_at | 0.390 | 7.040 | 1.999 | 4.019 | LOC_Os03g13840 | Senescence-associated protein, expressed |
| Os.28124.1.S1_at | 0.390 | 0.357 | 0.634 | 2.404 | LOC_Os10g28080 | Glycosyl hydrolases family 18 protein, expressed |
| OsAffx.30833.1.S1_at | 0.391 | 0.356 | 0.406 | 0.979 | LOC_Os11g05800 | TB2/DP1, HVA22 family protein, expressed |
| Os.23977.1.S1_at | 0.391 | 0.378 | 0.538 | 0.763 | LOC_Os03g43510 | expressed protein |
| Os.50346.1.S1_at | 0.392 | 3.223 | 1.628 | 1.876 | LOC_Os05g04680 | expressed protein |
| Os.48082.1.S1_at | 0.392 | 1.241 | 4.215 | 1.427 | LOC_Os09g25070 | WRKY DNA binding domain containing protein, expressed |
| Os.51866.1.S1_at | 0.393 | 9.248 | 5.017 | 2.709 | LOC_Os11g10770 | NB-ARC domain containing protein, expressed |
| Os.10861.1.S1_at | 0.394 | 2.865 | 1.730 | 3.323 | LOC_Os11g08100 | Eukaryotic aspartyl protease family protein, expressed |
| Os.57316.1.S1_at | 0.395 | 1.357 | 2.739 | 0.792 | LOC_Os05g38940 | expressed protein |
| Os.7314.1.S1_at | 0.396 | 5.184 | 5.391 | 1.126 | LOC_Os10g22520 | Cellulase containing protein, expressed |
| Os.26382.1.S1_at | 0.396 | 0.869 | 0.811 | 1.059 | LOC_Os05g12680 | Protein kinase domain containing protein, expressed |
| Os.27545.1.S1_at | 0.396 | 1.307 | 2.693 | 1.805 | LOC_Os04g38540 | Aldose 1-epimerase family protein, expressed |
| Os.20557.1.S1_s_at | 0.397 | 8.690 | 2.881 | 1.250 | LOC_Os11g10760 | Leucine Rich Repeat family protein, expressed |
| OsAffx.7406.1.S1_at | 0.398 | 0.116 | 0.353 | 0.340 | LOC_Os11g44300 | hypothetical protein |

| ProbeSetID | *OMTN3* -OE[a] | Stress response[b] | | | TIGR Locus ID[c] | Annotation |
|---|---|---|---|---|---|---|
| | | Drought | Salt | Cold | | |
| OsAffx.22380.1.S1_at | 0.398 | 1.580 | 0.910 | 3.608 | LOC_Os07g39680 | XYPPX repeat family protein, expressed |
| Os.609.3.S1_a_at | 0.398 | 2.656 | 1.656 | 1.838 | LOC_Os08g37670 | Plastocyanin-like domain containing protein, expressed |
| Os.50771.1.S1_at | 0.400 | 1.204 | 1.360 | 0.664 | LOC_Os06g44320 | expressed protein |
| Os.31171.1.S1_at | 0.400 | 0.538 | 0.992 | 1.506 | LOC_Os01g40290 | expressed protein |
| Os.27509.1.S1_at | 0.401 | 1.075 | 1.417 | 1.319 | LOC_Os01g53790 | expressed protein |
| Os.10384.1.S1_at | 0.402 | 3.570 | 2.101 | 2.041 | LOC_Os03g10950 | protein phosphatase 2C family protein, putative, expressed |
| Os.20548.1.S1_at | 0.402 | 6.117 | 10.887 | 7.483 | LOC_Os04g51680 | hypothetical protein |
| Os.12421.1.S1_at | 0.402 | 0.378 | 0.265 | 0.724 | LOC_Os11g42960 | plant integral membrane protein TIGR01569 containing protein, expressed |
| Os.40417.1.A1_at | 0.403 | 0.550 | 0.322 | 1.073 | LOC_Os07g14740 | Harpin-induced protein 1 containing protein, expressed |
| Os.11575.1.S1_a_at | 0.403 | 0.777 | 0.597 | 1.260 | LOC_Os03g46440 | BTB/POZ domain containing protein, expressed |
| Os.15295.2.S1_x_at | 0.406 | 1.727 | 1.105 | 3.192 | LOC_Os08g19670 | expressed protein |
| Os.8508.1.S1_at | 0.406 | 0.601 | 0.326 | 0.201 | LOC_Os10g38880 | uncharacterized plant-specific domain TIGR01568 family protein, expressed |
| Os.50175.2.S1_at | 0.407 | 1.590 | 2.039 | 0.585 | LOC_Os04g51820 | Cation transport protein, expressed |
| Os.8741.1.S1_at | 0.407 | 20.865 | 8.908 | 2.680 | LOC_Os03g12820 | Poly polymerase catalytic domain containing protein, expressed |
| Os.11711.1.S1_at | 0.407 | 0.693 | 0.757 | 1.444 | LOC_Os12g17880 | U-box domain containing protein, expressed |
| Os.23560.1.S1_at | 0.407 | 3.406 | 3.210 | 4.810 | LOC_Os04g45970 | Glutamate dehydrogenase, putative, expressed |
| OsAffx.24063.1.S1_at | 0.407 | 0.917 | 4.792 | 0.958 | LOC_Os02g02230 | Cytochrome P450 51, putative, expressed |
| OsAffx.19579.1.S1_at | 0.408 | 5.257 | 4.009 | 1.092 | LOC_Os12g09640 | Protein phosphatase 2C containing protein, expressed |
| Os.46287.1.S1_a_at | 0.408 | 2.099 | 1.490 | 2.182 | LOC_Os05g40770 | Protein kinase domain containing protein, expressed |
| Os.20230.1.S1_at | 0.408 | 5.830 | 4.452 | 1.517 | LOC_Os11g37950 | Barwin, putative, expressed |
| Os.12664.1.S1_at | 0.408 | 0.531 | 0.499 | 0.874 | LOC_Os07g35810 | Protein kinase domain containing protein, expressed |
| Os.53236.1.S1_at | 0.408 | 1.354 | 0.593 | 1.832 | LOC_Os02g43170 | B-box zinc finger family protein, expressed |
| Os.6321.1.S1_at | 0.408 | 3.679 | 4.107 | 7.535 | LOC_Os01g63690 | Nematode-resistance protein, putative, expressed |
| Os.10358.1.A1_at | 0.409 | 0.277 | 0.575 | 0.586 | LOC_Os10g04270 | Jacalin-like lectin domain containing protein, expressed |

| ProbeSetID | *OMTN3* -OE[a] | Stress response[b] | | | TIGR Locus ID[c] | Annotation |
|---|---|---|---|---|---|---|
| | | Drought | Salt | Cold | | |
| Os.9086.1.S1_at | 0.410 | 10.092 | 5.127 | 1.148 | LOC_Os03g10210 | Homeobox domain containing protein, expressed |
| Os.52147.1.S1_at | 0.411 | 3.759 | 2.718 | 0.658 | LOC_Os11g30760 | expressed protein |
| Os.11465.1.S1_at | 0.412 | 1.392 | 0.683 | 1.075 | LOC_Os11g33270 | xyloglucan endotransglucosylase/hydrolase precursor, putative, expressed |
| Os.7629.1.S1_at | 0.413 | 6.527 | 2.751 | 2.285 | LOC_Os09g30160 | Zinc finger, C3HC4 type family protein, expressed |
| OsAffx.23947.1.S1_at | 0.413 | 0.753 | 0.384 | 1.465 | LOC_Os01g66740 | Leucine Rich Repeat family protein |
| OsAffx.29638.1.S1_at | 0.414 | 1.086 | 0.293 | 1.000 | LOC_Os08g42840 | Leucine Rich Repeat family protein |
| Os.19374.1.S1_at | 0.414 | 1.579 | 1.035 | 3.561 | LOC_Os01g48440 | Glycosyltransferase family 43 protein, expressed |
| Os.14360.1.S1_x_at | 0.414 | 0.709 | 0.645 | 0.932 | LOC_Os07g09140 | expressed protein |
| Os.55991.1.S1_at | 0.416 | 5.346 | 3.803 | 2.449 | LOC_Os10g39090 | expressed protein |
| Os.48695.1.S1_x_at | 0.416 | 0.412 | 0.543 | 0.765 | LOC_Os07g35390 | Protein kinase domain containing protein, expressed |
| Os.21349.1.S1_at | 0.417 | 2.482 | 1.820 | 0.909 | LOC_Os03g04020 | Rare lipoprotein A like double-psi beta-barrel containing protein, expressed |
| Os.53763.1.A1_at | 0.417 | 2.714 | 0.229 | 0.314 | LOC_Os06g38340 | Leucine Rich Repeat family protein, expressed |
| Os.56891.1.S1_at | 0.417 | 1.031 | 0.811 | 1.338 | LOC_Os03g29930 | expressed protein |
| Os.49400.1.S2_s_at | 0.418 | 1.133 | 0.830 | 0.786 | LOC_Os11g11960 | NBS-LRR type disease resistance protein, putative, expressed |
| Os.52189.1.S1_at | 0.419 | 0.265 | 0.489 | 0.906 | LOC_Os06g19260 | expressed protein |
| OsAffx.1083.1.S1_at | 0.419 | 1.281 | 0.439 | 1.302 | LOC_Os01g66840 | Pectinacetylesterase family protein, expressed |
| Os.53726.1.S1_at | 0.420 | 5.651 | 4.368 | 0.765 | LOC_Os07g05370 | protein kinase family protein, putative, expressed |
| OsAffx.30258.1.S1_at | 0.420 | 1.655 | 1.266 | 1.195 | LOC_Os10g03570 | NB-ARC domain containing protein |
| Os.10050.1.S1_at | 0.421 | 2.490 | 1.447 | 1.645 | LOC_Os01g62660 | myb-like DNA-binding domain, SHAQKYF class family protein, expressed |
| Os.18717.2.S1_at | 0.422 | 1.557 | 3.307 | 0.634 | LOC_Os09g28180 | D-mannose binding lectin family protein, expressed |
| Os.26543.1.S1_at | 0.422 | 0.891 | 0.397 | 1.177 | LOC_Os03g58110 | Leucine Rich Repeat family protein, expressed |
| Os.158.1.S1_at | 0.422 | 0.295 | 0.632 | 0.850 | LOC_Os10g02070 | Peroxidase N precursor, putative, expressed |
| Os.159.1.S1_s_at | 0.422 | 0.144 | 0.545 | 0.515 | LOC_Os03g13210 | Peroxidase N precursor, putative, expressed |
| Os.45929.1.S1_at | 0.423 | 0.631 | 0.442 | 1.019 | LOC_Os05g34170 | Tubulin beta-6 chain, putative, expressed |

| ProbeSetID | *OMTN3* -OE[a] | Stress response[b] | | | TIGR Locus ID[c] | Annotation |
|---|---|---|---|---|---|---|
| | | Drought | Salt | Cold | | |
| Os.52377.1.S1_s_at | 0.423 | 0.357 | 3.907 | 1.712 | LOC_Os02g13370 | expressed protein |
| Os.25606.1.S1_at | 0.424 | 1.476 | 0.793 | 1.850 | LOC_Os09g25060 | WRKY DNA binding domain containing protein, expressed |
| Os.49501.1.A1_at | 0.424 | 2.798 | 2.460 | 2.123 | LOC_Os06g12230 | TCP family transcription factor containing protein, expressed |
| Os.37717.1.A1_s_at | 0.425 | 5.040 | 3.255 | 1.736 | LOC_Os05g15770 | Xylanase inhibitor protein 2 precursor, putative, expressed |
| Os.49337.1.S1_at | 0.425 | 0.380 | 0.628 | 0.798 | LOC_Os02g39140 | bHLH transcription factor GBOF-1, putative, expressed |
| Os.26755.1.S1_at | 0.425 | 0.280 | 0.700 | 0.626 | LOC_Os01g48610 | expressed protein |
| Os.2821.1.A1_s_at | 0.425 | 1.278 | 0.722 | 2.028 | LOC_Os02g02210 | aminotransferase, class III family protein, expressed |
| Os.25556.1.S1_x_at | 0.427 | 0.398 | 0.448 | 0.856 | LOC_Os08g28400 | expressed protein |
| Os.15872.1.S1_at | 0.428 | 1.094 | 0.920 | 1.450 | LOC_Os05g50710 | Late embryogenesis abundant protein Lea14-A, putative, expressed |
| Os.19277.1.S1_at | 0.428 | 0.291 | 0.964 | 0.845 | LOC_Os07g04150 | expressed protein |
| Os.27799.1.S1_at | 0.430 | 0.743 | 0.619 | 1.254 | LOC_Os04g52190 | Vacuolar sorting receptor 7 precursor, putative, expressed |
| Os.12551.1.S1_s_at | 0.431 | 52.177 | 31.401 | 0.940 | LOC_Os05g46480 | Late embryogenesis abundant protein, expressed |
| Os.49074.1.A1_at | 0.432 | 3.432 | 2.505 | 0.502 | LOC_Os07g37290 | hypothetical protein |
| OsAffx.5776.1.S1_at | 0.432 | 0.643 | 0.315 | 0.153 | LOC_Os08g10290 | SHR5-receptor-like kinase, putative, expressed |
| Os.25952.1.S1_at | 0.433 | 6.165 | 3.003 | 10.106 | LOC_Os05g27780 | expressed protein |
| OsAffx.4329.1.S1_at | 0.433 | 2.508 | 4.446 | 1.385 | LOC_Os05g13770 | RNA polymerase Rpb7, N-terminal domain containing protein |
| Os.12038.1.S1_at | 0.434 | 1.198 | 1.136 | 0.730 | LOC_Os01g65700 | expressed protein |
| OsAffx.21802.1.S1_at | 0.434 | 0.579 | 0.706 | 0.720 | LOC_Os01g66820 | receptor-like protein kinase, putative |
| OsAffx.3951.1.S1_at | 0.434 | 0.702 | 0.226 | 0.806 | LOC_Os04g30340 | wall-associated kinase 2, putative |
| Os.46160.1.S1_at | 0.435 | 0.161 | 0.290 | 1.613 | LOC_Os10g30790 | phosphate:H+ symporter family protein, expressed |
| Os.7339.1.S1_at | 0.437 | 1.839 | 1.648 | 1.933 | LOC_Os10g31850 | CHY zinc finger family protein, expressed |
| Os.26938.1.A1_at | 0.437 | 0.149 | 0.409 | 0.701 | LOC_Os02g42150 | Calcium binding EGF domain containing protein, expressed |
| Os.18395.1.S1_s_at | 0.440 | 1.088 | 0.581 | 0.567 | LOC_Os06g15620 | GAST1 protein precursor, putative, expressed |
| Os.9841.1.S1_at | 0.441 | 2.585 | 1.060 | 1.328 | LOC_Os03g10390 | expressed protein |

| ProbeSetID | *OMTN3* -OE[a] | Stress response[b] | | | TIGR Locus ID[c] | Annotation |
|---|---|---|---|---|---|---|
| | | Drought | Salt | Cold | | |
| Os.53581.1.S1_at | 0.441 | 4.552 | 4.348 | 5.345 | LOC_Os09g37080 | expressed protein |
| Os.15943.1.S1_at | 0.442 | 0.533 | 0.961 | 1.654 | LOC_Os01g67310 | Patatin-like phospholipase family protein, expressed |
| Os.27553.2.S1_at | 0.442 | 0.950 | 0.489 | 0.211 | LOC_Os07g27300 | RNA-binding protein Luc7-like 1, putative, expressed |
| Os.10784.1.S1_at | 0.442 | 2.745 | 1.943 | 1.799 | LOC_Os02g26700 | ChaC-like protein, expressed |
| OsAffx.13993.1.S1_at | 0.442 | 1.238 | 1.103 | 1.270 | LOC_Os04g27100 | WRKY transcription factor 19, putative |
| Os.14951.1.S1_at | 0.443 | 8.699 | 10.099 | 2.665 | LOC_Os08g39730 | Cytochrome P450 family protein, expressed |
| Os.47445.1.S1_at | 0.443 | 1.503 | 1.340 | 1.357 | LOC_Os02g26210 | flowering promoting factor-like 1, putative, expressed |
| Os.14125.1.S1_at | 0.443 | 0.644 | 1.600 | 7.577 | LOC_Os09g35030 | DREB1A protein, putative, expressed |
| Os.51757.1.S1_at | 0.443 | 1.405 | 1.042 | 0.813 | LOC_Os06g26270 | expressed protein |
| Os.51787.1.S1_at | 0.443 | 0.435 | 0.444 | 1.350 | LOC_Os12g24320 | Cell Division Protein AAA ATPase family, putative, expressed |
| Os.12633.1.S1_at | 0.443 | 256.935 | 143.577 | 1.561 | LOC_Os11g26790 | Dehydrin family protein, expressed |
| OsAffx.31316.1.S1_at | 0.443 | 0.308 | 0.159 | 0.224 | LOC_Os11g35850 | Leucine Rich Repeat family protein, expressed |
| Os.15426.1.S1_at | 0.444 | 0.510 | 0.440 | 1.109 | LOC_Os07g35370 | Protein kinase domain containing protein, expressed |
| Os.34779.1.S1_at | 0.444 | 0.839 | 2.607 | 0.607 | LOC_Os01g06520 | Leucine Rich Repeat family protein, expressed |
| Os.55254.1.S1_at | 0.444 | 1.036 | 1.313 | 1.133 | LOC_Os06g10130 | expressed protein |
| Os.53337.1.S1_at | 0.445 | 1.609 | 0.203 | 1.563 | LOC_Os02g17090 | Subtilisin N-terminal Region family protein, expressed |
| Os.9276.1.S1_s_at | 0.446 | 0.113 | 0.315 | 0.808 | LOC_Os07g07900 | expressed protein |
| OsAffx.15371.1.S1_at | 0.446 | 0.713 | 0.323 | 0.717 | LOC_Os06g11520 | LMBR1 integral membrane family protein, putative, expressed |
| Os.27386.1.S1_at | 0.446 | 0.375 | 0.505 | 1.154 | LOC_Os07g41250 | POT family protein, expressed |
| Os.26698.1.S1_a_at | 0.448 | 2.153 | 3.198 | 1.527 | LOC_Os01g19820 | universal stress protein family protein, expressed |
| Os.53174.1.A1_s_at | 0.448 | 0.541 | 0.841 | 1.116 | LOC_Os02g48350 | Diacylglycerol acyltransferase family protein, expressed |
| OsAffx.14098.1.S1_at | 0.449 | 2.370 | 1.008 | 1.451 | LOC_Os04g32590 | expressed protein |
| OsAffx.30204.1.S1_at | 0.449 | 1.521 | 0.827 | 1.311 | LOC_Os09g39190 | Copine family protein, expressed |

| ProbeSetID | *OMTN3* -OE[a] | Stress response[b] | | | TIGR Locus ID[c] | Annotation |
|---|---|---|---|---|---|---|
| | | Drought | Salt | Cold | | |
| OsAffx.17279.1.S1_at | 0.449 | 1.515 | 3.061 | 2.303 | LOC_Os08g31770 | NB-ARC domain containing protein, expressed |
| Os.33210.1.S1_at | 0.450 | 1.869 | 1.445 | 0.759 | LOC_Os01g68570 | expressed protein |
| Os.2677.1.S1_at | 0.450 | 41.625 | 18.023 | 2.220 | LOC_Os07g48830 | galactinol synthase 3, putative, expressed |
| Os.7281.1.S1_at | 0.451 | 0.433 | 0.831 | 1.292 | LOC_Os01g68730 | expressed protein |
| Os.51063.1.S1_at | 0.451 | 22.692 | 10.647 | 4.776 | LOC_Os09g28210 | Helix-loop-helix DNA-binding domain containing protein, expressed |
| Os.22086.1.S1_at | 0.452 | 0.129 | 0.779 | 1.147 | LOC_Os07g48020 | Peroxidase 2 precursor, putative, expressed |
| Os.27279.1.A1_at | 0.452 | 26.757 | 6.295 | 1.570 | LOC_Os03g26870 | WD-40 repeat family protein, putative, expressed |
| Os.49615.1.S1_at | 0.452 | 0.566 | 0.848 | 1.216 | LOC_Os03g45960 | P21 protein, putative, expressed |
| Os.26983.1.S1_s_at | 0.453 | 0.263 | 0.403 | 0.524 | LOC_Os01g04570 | Protein kinase domain containing protein, expressed |
| Os.10500.1.S1_at | 0.453 | 1.171 | 0.831 | 1.687 | LOC_Os05g39610 | Exo70 exocyst complex subunit family protein, expressed |
| Os.50019.1.S1_at | 0.453 | 761.382 | 105.602 | 4.463 | LOC_Os03g04080 | expressed protein |
| Os.11565.1.S1_at | 0.454 | 1.304 | 1.684 | 0.755 | LOC_Os10g02040 | Peroxidase 53 precursor, putative, expressed |
| Os.6035.1.S1_at | 0.454 | 0.391 | 0.690 | 0.662 | LOC_Os07g46350 | Serine carboxypeptidase II-3 precursor, putative, expressed |
| Os.39087.1.S1_at | 0.454 | 1.762 | 1.344 | 1.987 | LOC_Os01g14550 | Pathogen-related protein, putative, expressed |
| Os.10293.1.S1_at | 0.456 | 1.343 | 1.369 | 1.269 | LOC_Os07g42510 | AP2 domain containing protein, expressed |
| Os.7756.1.S1_at | 0.456 | 0.126 | 0.221 | 1.315 | LOC_Os10g36500 | pectinesterase inhibitor domain containing protein, expressed |
| Os.27797.1.A1_at | 0.456 | 2.723 | 2.578 | 1.053 | LOC_Os03g06570 | IQ calmodulin-binding motif family protein, expressed |
| Os.27755.1.S1_at | 0.457 | 0.306 | 0.344 | 0.583 | LOC_Os07g35560 | Glycosyl hydrolases family 17 protein, expressed |
| Os.19401.1.S1_at | 0.458 | 3.329 | 2.897 | 2.103 | LOC_Os03g04100 | expressed protein |
| OsAffx.13523.1.S1_at | 0.458 | 1.984 | 0.889 | 1.238 | LOC_Os03g57400 | hypothetical protein |
| Os.10855.1.S1_at | 0.458 | 4.595 | 1.533 | 1.105 | LOC_Os03g56060 | glycosyl transferase, group 2 family protein, expressed |
| Os.27112.1.S1_at | 0.459 | 1.923 | 2.318 | 1.247 | LOC_Os01g09800 | Regulatory protein NPR1, putative, expressed |
| Os.11176.1.S1_at | 0.459 | 3.087 | 1.923 | 0.957 | LOC_Os02g57400 | 2-phosphoglycerate kinase, putative, expressed |

| ProbeSetID | *OMTN3* -OE[a] | Stress response[b] | | | TIGR Locus ID[c] | Annotation |
|---|---|---|---|---|---|---|
| | | Drought | Salt | Cold | | |
| Os.22731.1.S1_at | 0.459 | 9.240 | 14.323 | 0.888 | LOC_Os03g16020 | 17.4 kDa class I heat shock protein, putative, expressed |
| Os.6542.1.S1_at | 0.459 | 2.620 | 5.965 | 3.089 | LOC_Os03g08630 | dihydrokaempferol 4-reductase, putative, expressed |
| OsAffx.15825.1.S1_at | 0.459 | 0.644 | 1.184 | 2.080 | LOC_Os06g38830 | Leucine Rich Repeat family protein, expressed |
| OsAffx.8290.1.S1_at | 0.460 | 1.580 | 1.037 | 0.370 | LOC_Os10g17960 | receptor-like protein kinase homolog RK20-1, putative, expressed |
| Os.11408.1.S2_at | 0.460 | 6.034 | 3.263 | 1.221 | LOC_Os09g37100 | Phospholipase D delta, putative, expressed |
| Os.7217.1.S1_a_at | 0.461 | 0.621 | 0.814 | 1.123 | LOC_Os01g07140 | Kelch motif family protein, expressed |
| Os.51359.1.S1_at | 0.461 | 1.153 | 2.855 | 0.591 | LOC_Os07g48680 | Zinc finger, C3HC4 type family protein, expressed |
| Os.46566.1.S1_at | 0.462 | 2.732 | 4.268 | 0.687 | LOC_Os10g17940 | F-box domain containing protein |
| Os.7335.1.S1_at | 0.463 | 2.551 | 1.397 | 2.497 | LOC_Os02g36340 | Riboflavin biosynthesis protein ribA, chloroplast precursor, putative, expressed |
| Os.53208.1.S1_at | 0.463 | 0.307 | 0.667 | 0.470 | LOC_Os09g29600 | Calcium binding EGF domain containing protein, expressed |
| Os.52868.1.S1_at | 0.464 | 4.743 | 2.178 | 2.663 | LOC_Os02g54590 | serine threonine kinase 1, putative, expressed |
| Os.12724.1.S1_a_at | 0.464 | 2.123 | 2.034 | 1.030 | LOC_Os08g36320 | Glutamate decarboxylase, putative, expressed |
| Os.19497.1.S1_at | 0.465 | 0.977 | 0.629 | 1.161 | LOC_Os01g72380 | expressed protein |
| Os.3391.1.S1_at | 0.465 | 4.220 | 4.324 | 0.544 | LOC_Os09g23620 | typical P-type R2R3 Myb protein, putative, expressed |
| Os.45991.1.S1_x_at | 0.466 | 4.011 | 3.827 | 1.880 | LOC_Os01g10440 | glycosyltransferase family 14 protein, putative, expressed |
| Os.54493.1.S1_at | 0.467 | 0.898 | 0.671 | 1.993 | LOC_Os06g14490 | calmodulin-binding heat-shock protein, putative, expressed |
| Os.17102.1.S1_at | 0.467 | 1.519 | 1.488 | 1.507 | LOC_Os01g66130 | arm repeat-containing protein, putative, expressed |
| Os.52483.1.S1_at | 0.468 | 0.240 | 0.567 | 0.680 | LOC_Os06g19380 | transporter, putative, expressed |
| Os.20572.2.S1_at | 0.469 | 0.118 | 0.471 | 0.702 | LOC_Os03g22620 | Terpene synthase family, metal binding domain containing protein, expressed |
| Os.51246.1.S1_at | 0.470 | 0.193 | 0.193 | 1.209 | LOC_Os11g11780 | serine/threonine protein kinase, putative, expressed |
| Os.17766.1.S1_at | 0.470 | 1.397 | 0.962 | 1.429 | LOC_Os06g12120 | BRASSINOSTEROID INSENSITIVE 1-associated receptor kinase 1 precursor, putative, expressed |
| OsAffx.12515.1.S1_at | 0.470 | 1.107 | 1.275 | 2.486 | LOC_Os02g46090 | Calcium-dependent protein kinase, isoform 11, putative, expressed |
| Os.31375.1.S1_at | 0.470 | 0.785 | 1.002 | 0.844 | LOC_Os01g73410 | Chromosome condensation factor, putative, expressed |

| ProbeSetID | *OMTN3* -OE[a] | Stress response[b] | | | TIGR Locus ID[c] | Annotation |
|---|---|---|---|---|---|---|
| | | Drought | Salt | Cold | | |
| Os.12642.1.S1_at | 0.471 | 8.266 | 2.362 | 5.968 | LOC_Os08g42750 | Calcium-dependent protein kinase, isoform AK1, putative, expressed |
| Os.54784.1.S1_at | 0.471 | 1.397 | 0.852 | 0.949 | LOC_Os05g38460 | Myb-like DNA-binding domain containing protein, expressed |
| OsAffx.24724.1.S1_x_at | 0.473 | 0.483 | 0.637 | 1.210 | LOC_Os02g44600 | U-box domain containing protein, expressed |
| Os.4807.1.S1_at | 0.475 | 0.716 | 0.527 | 1.878 | LOC_Os04g54200 | diacylglycerol kinase, putative, expressed |
| Os.57031.1.A1_at | 0.476 | 1.104 | 0.896 | 0.338 | LOC_Os12g42630 | transposon protein, putative, Mutator sub-class, expressed |
| OsAffx.5110.1.S1_at | 0.476 | 1.872 | 1.678 | 1.254 | LOC_Os06g45020 | Leucine Rich Repeat family protein, expressed |
| OsAffx.17460.1.S1_at | 0.476 | 0.762 | 0.830 | 1.358 | LOC_Os08g41590 | POT family protein, expressed |
| Os.50359.1.S1_at | 0.478 | 0.281 | 0.493 | 1.097 | LOC_Os09g26460 | zinc finger family protein, putative, expressed |
| OsAffx.12065.1.S1_at | 0.478 | 0.485 | 1.971 | 0.250 | LOC_Os02g16060 | NBS-LRR disease resistance protein, putative, expressed |
| Os.56048.1.S1_s_at | 0.478 | 0.246 | 0.507 | 1.014 | LOC_Os01g68740 | expressed protein |
| Os.12793.1.S1_x_at | 0.478 | 1.095 | 1.226 | 2.415 | LOC_Os01g54340 | uncharacterized plant-specific domain TIGR01615 family protein, expressed |
| Os.46777.1.S1_at | 0.479 | 0.421 | 0.363 | 0.888 | LOC_Os04g29580 | Calcium binding EGF domain containing protein, expressed |
| Os.51127.1.S1_a_at | 0.479 | 0.282 | 0.686 | 0.568 | LOC_Os04g37820 | Cytokinin-O-glucosyltransferase 2, putative, expressed |
| Os.19001.1.S1_at | 0.479 | 1.622 | 1.076 | 1.040 | LOC_Os05g31890 | expressed protein |
| Os.26892.1.A1_at | 0.479 | 0.475 | 0.784 | 1.008 | LOC_Os03g30890 | D-mannose binding lectin family protein, expressed |
| Os.6683.1.S1_at | 0.479 | 17.377 | 5.512 | 11.082 | LOC_Os01g58130 | expressed protein |
| Os.19385.1.S1_at | 0.480 | 14.141 | 12.023 | 4.867 | LOC_Os01g52730 | expressed protein |
| Os.22676.1.S1_at | 0.480 | 1.807 | 1.840 | 2.441 | LOC_Os02g53750 | serine/threonine-protein kinase NAK, putative, expressed |
| Os.38006.1.S1_at | 0.481 | 1.724 | 1.549 | 1.349 | LOC_Os03g20530 | expressed protein |
| OsAffx.17563.1.S1_at | 0.481 | 0.241 | 0.725 | 0.128 | LOC_Os09g04430 | hypothetical protein |
| Os.54269.1.S1_s_at | 0.482 | 2.176 | 1.297 | 1.255 | LOC_Os05g48060 | Phosphatidyl serine synthase family protein, expressed |
| OsAffx.12774.1.S1_s_at | 0.482 | 2.893 | 1.850 | 1.146 | LOC_Os03g07890 | mitochondrial carrier, putative, expressed |
| OsAffx.30194.1.S1_x_at | 0.482 | 1.372 | 1.937 | 0.679 | LOC_Os09g38840 | wall-associated kinase-like 1, putative, expressed |
| Os.15607.1.S1_at | 0.482 | 1.042 | 2.231 | 2.316 | LOC_Os04g31120 | beta scruin, putative, expressed |

| ProbeSetID | *OMTN3* -OE[a] | Stress response[b] | | | TIGR Locus ID[c] | Annotation |
|---|---|---|---|---|---|---|
| | | Drought | Salt | Cold | | |
| Os.51307.1.S1_at | 0.482 | 0.747 | 1.349 | 2.941 | LOC_Os11g47630 | Zinc finger, C2H2 type family protein, expressed |
| Os.5194.1.S1_x_at | 0.483 | 0.462 | 0.573 | 0.925 | LOC_Os01g66110 | dehydration-responsive protein, putative, expressed |
| Os.49329.1.S1_at | 0.483 | 0.758 | 0.337 | 1.255 | LOC_Os02g35490 | MLO-like protein 1, putative, expressed |
| Os.53673.1.S1_at | 0.483 | 2.416 | 2.377 | 2.022 | LOC_Os04g01310 | D-mannose binding lectin family protein, expressed |
| Os.10591.1.S1_at | 0.484 | 2.406 | 1.002 | 0.904 | LOC_Os04g50790 | Aldose reductase-related protein, putative, expressed |
| Os.17199.1.S1_at | 0.485 | 1.618 | 1.612 | 0.698 | LOC_Os07g31650 | expressed protein |
| Os.49527.1.S1_at | 0.485 | 0.143 | 0.069 | 0.916 | LOC_Os03g03790 | acyl-activating enzyme 11, putative, expressed |
| Os.6125.1.S1_at | 0.485 | 0.723 | 0.785 | 1.460 | LOC_Os03g29190 | PDI, putative, expressed |
| Os.46881.1.S1_at | 0.485 | 1.726 | 0.637 | 0.500 | LOC_Os10g39770 | Zinc finger, C3HC4 type family protein, expressed |
| Os.6662.1.S1_at | 0.485 | 8.889 | 4.342 | 1.814 | LOC_Os05g44900 | expressed protein |
| Os.14285.2.S1_at | 0.486 | 0.406 | 0.511 | 1.465 | LOC_Os04g31550 | expressed protein |
| OsAffx.31710.1.S1_x_at | 0.486 | 5.188 | 2.823 | 3.467 | LOC_Os12g12260 | Diacylglycerol kinase 1, putative, expressed |
| OsAffx.23877.2.S1_at | 0.486 | 0.594 | 0.769 | 1.327 | LOC_Os01g60800 | expressed protein |
| Os.14076.1.S1_s_at | 0.486 | 2.119 | 1.534 | 0.916 | LOC_Os02g44820 | expressed protein |
| Os.23008.1.S1_at | 0.486 | 0.746 | 1.288 | 1.907 | LOC_Os10g39710 | Strictosidine synthase family protein, expressed |
| Os.648.1.S1_at | 0.487 | 7.494 | 3.226 | 2.188 | LOC_Os01g43480 | ATPase, AAA family protein, expressed |
| Os.21932.1.S1_at | 0.487 | 3.514 | 3.361 | 0.843 | LOC_Os10g42130 | no apical meristem, putative, expressed |
| Os.50287.1.S1_at | 0.487 | 4.973 | 2.965 | 5.186 | LOC_Os04g39010 | heavy metal-associated domain containing protein, expressed |
| Os.50971.1.S1_at | 0.487 | 0.292 | 0.568 | 1.042 | LOC_Os11g42970 | plant integral membrane protein TIGR01569 containing protein, expressed |
| Os.55696.1.S1_at | 0.487 | 0.662 | 0.853 | 0.472 | LOC_Os12g39100 | expressed protein |
| Os.11327.1.S1_at | 0.488 | 3.669 | 2.953 | 1.370 | LOC_Os03g18130 | Asparagine synthetase, putative, expressed |
| Os.50198.1.S1_at | 0.488 | 6.481 | 7.130 | 2.858 | LOC_Os12g40180 | expressed protein |
| Os.49208.1.S1_at | 0.489 | 0.595 | 0.243 | 1.681 | LOC_Os04g34270 | D-mannose binding lectin family protein, expressed |
| Os.6501.1.A1_at | 0.489 | 2.210 | 1.577 | 1.026 | LOC_Os08g43180 | expressed protein |

| ProbeSetID | *OMTN3* -OE[a] | Stress response[b] | | | TIGR Locus ID[c] | Annotation |
|---|---|---|---|---|---|---|
| | | Drought | Salt | Cold | | |
| Os.5334.2.S1_a_at | 0.489 | 2.788 | 1.301 | 1.963 | LOC_Os01g65780 | secondary cell wall-related glycosyltransferase family 8, putative, expressed |
| Os.35583.1.S1_at | 0.490 | 0.180 | 0.535 | 0.499 | LOC_Os01g48950 | expressed protein |
| Os.27466.1.S1_at | 0.490 | 0.405 | 0.756 | 1.191 | LOC_Os11g30910 | Sulfotransferase domain containing protein, expressed |
| Os.3721.1.S1_at | 0.491 | 1.451 | 1.140 | 1.466 | LOC_Os01g03980 | expressed protein |
| Os.44751.1.S1_at | 0.491 | 0.959 | 0.901 | 1.390 | LOC_Os06g06760 | U-box domain containing protein, expressed |
| OsAffx.28714.1.S1_x_at | 0.491 | 0.959 | 0.901 | 1.390 | LOC_Os07g32570 | 5'-adenylylsulfate reductase 2, chloroplast precursor, putative, expressed |
| Os.37872.1.S1_at | 0.491 | 6.281 | 2.762 | 1.102 | LOC_Os06g08280 | Protein kinase domain containing protein, expressed |
| Os.21634.1.S1_at | 0.491 | 9.253 | 5.817 | 2.315 | LOC_Os01g55240 | Gibberellin 2-beta-dioxygenase, putative, expressed |
| OsAffx.17899.1.S1_at | 0.492 | 0.870 | 1.141 | 1.167 | LOC_Os09g25930 | Ligand-gated ion channel family protein |
| Os.9511.1.S1_at | 0.492 | 0.904 | 0.686 | 1.889 | LOC_Os03g40670 | Glycerophosphoryl diester phosphodiesterase family protein, expressed |
| Os.17805.1.S1_at | 0.492 | 8.455 | 3.544 | 1.548 | LOC_Os01g63930 | Cytochrome P450 family protein, expressed |
| Os.24878.1.A1_at | 0.492 | 0.738 | 1.013 | 1.470 | LOC_Os01g41750 | Leucine Rich Repeat family protein, expressed |
| Os.40423.2.S1_x_at | 0.492 | 1.376 | 0.282 | 0.792 | LOC_Os01g41780 | Leucine Rich Repeat family protein, expressed |
| Os.49577.1.S1_at | 0.492 | 9.844 | 6.365 | 1.102 | LOC_Os02g50340 | MAC/Perforin domain containing protein, expressed |
| Os.14616.1.S1_at | 0.493 | 3.049 | 0.953 | 1.947 | LOC_Os07g34260 | Chalcone and stilbene synthases, N-terminal domain containing protein, expressed |
| Os.26517.1.S1_at | 0.493 | 28.947 | 20.369 | 4.124 | LOC_Os02g44990 | F-box domain containing protein, expressed |
| Os.55343.1.S1_at | 0.493 | 3.947 | 4.715 | 0.896 | LOC_Os04g56030 | expressed protein |
| OsAffx.31963.1.S1_at | 0.493 | 0.329 | 0.553 | 0.824 | LOC_Os12g31540 | expressed protein |
| Os.7699.1.S1_at | 0.493 | 3.428 | 2.658 | 1.919 | LOC_Os10g22050 | expressed protein |
| OsAffx.30738.1.S1_at | 0.493 | 2.028 | 0.958 | 1.347 | LOC_Os10g40650 | expressed protein |
| OsAffx.2268.1.S1_at | 0.494 | 0.338 | 0.916 | 0.297 | LOC_Os01g47820 | S-locus-like receptor protein kinase, putative, expressed |
| Os.10054.1.S1_at | 0.494 | 2.876 | 0.768 | 1.099 | LOC_Os02g46650 | Ubiquitin carboxyl-terminal hydrolase family protein, expressed |
| Os.51657.1.S1_at | 0.495 | 0.203 | 0.581 | 0.700 | LOC_Os08g44830 | zinc finger family protein, putative, expressed |

| ProbeSetID | *OMTN3* -OE[a] | Stress response[b] | | | TIGR Locus ID[c] | Annotation |
|---|---|---|---|---|---|---|
| | | Drought | Salt | Cold | | |
| Os.6170.1.S1_at | 0.495 | 0.610 | 0.605 | 1.748 | LOC_Os02g51060 | glycosyl transferase, group 2 family protein, expressed |
| OsAffx.29832.2.S1_at | 0.495 | 0.615 | 0.214 | 0.957 | LOC_Os09g13440 | expressed protein |
| Os.27986.1.A1_at | 0.495 | 0.730 | 0.835 | 0.984 | LOC_Os09g32550 | Glycosyl hydrolases family 17 protein, expressed |
| Os.55064.1.S1_at | 0.495 | 0.905 | 0.855 | 0.742 | LOC_Os06g25250 | RNase3 domain containing protein, expressed |
| Os.7125.1.S1_at | 0.496 | 0.315 | 0.808 | 1.280 | LOC_Os09g36730 | Myb-related protein Hv1, putative, expressed |
| Os.54501.1.S1_at | 0.496 | 5.083 | 1.100 | 0.769 | LOC_Os11g12650 | PHD-finger family protein, expressed |
| Os.24971.1.A1_s_at | 0.496 | 0.477 | 0.565 | 0.322 | LOC_Os04g29960 | Calcium binding EGF domain containing protein, expressed |
| Os.31670.2.S1_at | 0.497 | 1.196 | 0.901 | 0.994 | LOC_Os01g43330 | expressed protein |
| Os.51809.1.S1_at | 0.497 | 1.842 | 2.377 | 1.091 | LOC_Os11g06780 | Protein kinase domain containing protein, expressed |
| Os.47984.1.A1_at | 0.497 | 0.322 | 0.611 | 0.706 | LOC_Os02g51950 | expressed protein |
| Os.26928.1.S2_at | 0.498 | 1.738 | 3.714 | 2.143 | LOC_Os08g29020 | wall-associated kinase 2, putative, expressed |
| Os.7870.1.S1_at | 0.498 | 2.106 | 1.266 | 1.506 | LOC_Os02g39850 | Transferase family protein, expressed |
| OsAffx.28651.1.S1_x_at | 0.498 | 1.429 | 1.527 | 2.905 | LOC_Os07g28850 | Piwi domain containing protein, expressed |
| Os.32022.1.S1_x_at | 0.498 | 14.641 | 2.360 | 2.761 | LOC_Os07g37620 | Cotton fibre expressed protein, expressed |
| Os.17271.1.S1_at | 0.499 | 0.322 | 0.590 | 1.225 | LOC_Os12g07310 | Citrate binding protein precursor, putative, expressed |
| OsAffx.27816.1.S1_at | 0.499 | 8.545 | 7.280 | 1.922 | LOC_Os06g25010 | Xylanase inhibitor protein 1 precursor, putative, expressed |
| Os.3655.1.S1_at | 0.499 | 0.357 | 0.247 | 0.523 | LOC_Os01g18730 | hypothetical protein |

NOTE: All values in the table are expression level change folds (transgenic/WT, or stress/normal)(mean of the repeats).The folds higher than 2 were indicated by red colour, and the folds lower than 0.5 were indicated by green colour.

[a]Expression level change folds in the *OMTN3*-OE plants.

[b]Expression profile of the genes under drought, salt and cold stress was download from the GEO database (http://www.ncbi.nlm.nih.gov/geo/, accession number: GSE6901).

[c]The locus ID was download from Rice Annotation Project (http://rice.plantbiology.msu.edu/).

**Supplementary Table S6. Up- and down-regulated genes in the transgenic rice plants overexpressing *OMTN4*.**

| ProbeSetID | *OMTN4* -OE[a] | Stress response[b] | | | TIGR Locus ID[c] | Annotation |
|---|---|---|---|---|---|---|
| | | Drought | Salt | Cold | | |
| Os.10266.1.S1_at | 7.489 | 0.167 | 0.362 | 0.630 | LOC_Os03g43100 | expressed protein |
| Os.31908.1.S1_at | 7.456 | 0.991 | 1.047 | 0.635 | LOC_Os01g12440 | AP2 domain containing protein, expressed |
| Os.17047.1.A1_at | 6.827 | 0.564 | 1.205 | 0.910 | LOC_Os07g18230 | Protein kinase domain containing protein, expressed |
| Os.5816.1.S1_at | 5.155 | 0.683 | 0.276 | 12.233 | LOC_Os09g35010 | AP2 domain containing protein, expressed |
| Os.6372.1.S1_at | 4.793 | 18.720 | 1.593 | 1.071 | LOC_Os03g10110 | Cupin family protein, expressed |
| Os.7991.1.S1_at | 4.348 | 3.486 | 1.811 | 1.730 | LOC_Os04g41620 | Endochitinase A precursor, putative, expressed |
| Os.38806.1.A1_x_at | 3.884 | 12.732 | 5.207 | 6.969 | LOC_Os06g04230 | expressed protein |
| OsAffx.21073.1.S1_at | 3.856 | 0.559 | 0.401 | 0.494 | LOC_Os07g02620 | NB-ARC domain containing protein |
| Os.27497.1.S1_at | 3.781 | 0.191 | 0.121 | 0.681 | LOC_Os12g12390 | transposon protein, putative, CACTA, En/Spm sub-class, expressed |
| Os.26868.1.A1_at | 3.748 | 0.210 | 1.040 | 1.078 | LOC_Os03g10100 | Sugar transporter family protein, expressed |
| OsAffx.31235.1.S1_at | 3.702 | 1.047 | 0.567 | 1.150 | LOC_Os11g35300 | hypothetical protein |
| Os.3808.4.S1_x_at | 3.621 | 2.061 | 1.227 | 5.266 | LOC_Os01g09220 | transposon protein, putative, CACTA, En/Spm sub-class, expressed |
| Os.17677.1.S1_at | 3.537 | 3.101 | 1.985 | 3.218 | LOC_Os06g03810 | expressed protein |
| Os.34982.1.A1_at | 3.517 | 0.348 | 0.828 | 0.751 | LOC_Os04g17660 | Rhodanese-like domain containing protein, expressed |
| Os.51979.1.S1_at | 3.509 | 0.867 | 2.778 | 2.111 | LOC_Os03g37140 | expressed protein |
| Os.10126.1.S1_at | 3.403 | 1.860 | 1.927 | 3.544 | LOC_Os09g24580 | EF hand family protein, expressed |
| Os.8480.1.S1_at | 3.351 | 0.243 | 0.186 | 2.271 | LOC_Os05g30700 | expressed protein |
| Os.3710.1.S1_at | 3.195 | 1.230 | 1.677 | 5.611 | LOC_Os02g35440 | E3 ubiquitin ligase EL5, putative |
| Os.53074.1.S1_at | 3.194 | 1.341 | 5.349 | 8.467 | LOC_Os03g20330 | VQ motif family protein, expressed |
| Os.7902.1.S1_at | 2.936 | 2.454 | 1.608 | 2.546 | LOC_Os06g46950 | EF-hand Ca2+-binding protein CCD1, putative, expressed |
| OsAffx.32170.1.S1_at | 2.879 | 2.386 | 1.087 | 1.204 | LOC_Os12g43450 | P21 protein, putative, expressed |
| Os.18922.2.S1_at | 2.695 | 17.632 | 7.684 | 9.289 | LOC_Os01g72530 | EF hand family protein, expressed |
| Os.140.3.S1_x_at | 2.693 | 2.249 | 2.320 | 10.973 | LOC_Os04g58810 | CAF1 family ribonuclease containing protein, expressed |

| ProbeSetID | *OMTN4* -OE[a] | Stress response[b] | | | TIGR Locus ID[c] | Annotation |
|---|---|---|---|---|---|---|
| | | Drought | Salt | Cold | | |
| Os.37996.1.S1_at | 2.684 | 0.993 | 1.413 | 1.756 | LOC_Os07g43800 | EF hand family protein, expressed |
| Os.17158.1.S1_at | 2.665 | 1.042 | 1.382 | 1.592 | LOC_Os01g70490 | Potassium transporter 5, putative, expressed |
| Os.15829.1.S1_at | 2.663 | 0.382 | 1.711 | 0.307 | LOC_Os02g10120 | Lipoxygenase 2.3, chloroplast precursor, putative, expressed |
| Os.159.1.S1_s_at | 2.615 | 0.144 | 0.545 | 0.515 | LOC_Os03g13210 | Peroxidase N precursor, putative, expressed |
| Os.10556.1.S1_at | 2.490 | 8.593 | 9.683 | 0.495 | LOC_Os02g15860 | expressed protein |
| Os.51345.1.S1_at | 2.463 | 1.131 | 2.485 | 2.068 | LOC_Os05g08860 | expressed protein |
| Os.52974.1.S1_at | 2.450 | 2.827 | 3.373 | 1.569 | LOC_Os04g50940 | POT family protein, expressed |
| Os.7028.1.S1_at | 2.448 | 0.599 | 0.683 | 1.715 | LOC_Os02g45710 | Zinc finger, C3HC4 type family protein, expressed |
| Os.26822.1.S1_at | 2.437 | 0.494 | 0.456 | 0.629 | LOC_Os04g35030 | cellulose synthase family protein, putative, expressed |
| Os.12728.1.S1_at | 2.419 | 0.826 | 0.587 | 1.242 | LOC_Os03g12290 | Glutamine synthetase root isozyme 5, putative, expressed |
| OsAffx.14605.1.S1_at | 2.417 | 1.501 | 1.918 | 3.028 | LOC_Os05g09020 | WRKY transcription factor 50, putative, expressed |
| OsAffx.19103.1.S1_at | 2.406 | 0.756 | 2.556 | 3.244 | LOC_Os11g31530 | BRASSINOSTEROID INSENSITIVE 1-associated receptor kinase 1 precursor, putative |
| Os.35642.2.S1_x_at | 2.404 | 0.560 | 0.763 | 1.633 | LOC_Os01g58280 | Subtilisin N-terminal Region family protein, expressed |
| Os.5390.1.S1_at | 2.397 | 1.123 | 1.416 | 2.828 | LOC_Os12g33130 | expressed protein |
| Os.30528.1.S1_at | 2.393 | 52.308 | 18.077 | 0.923 | LOC_Os08g31860 | expressed protein |
| Os.5606.1.S1_at | 2.390 | 1.667 | 4.500 | 1.583 | LOC_Os07g37690 | UDP-glucoronosyl and UDP-glucosyl transferase family protein, expressed |
| Os.22485.1.A1_at | 2.367 | 0.273 | 0.324 | 1.008 | LOC_Os11g29720 | Cytochrome P450 family protein, expressed |
| OsAffx.5196.1.S1_at | 2.366 | 0.449 | 0.764 | 0.584 | LOC_Os07g03900 | Lectin receptor-type protein kinase, putative |
| Os.11707.1.A1_at | 2.359 | 0.143 | 1.445 | 0.298 | LOC_Os03g54130 | Papain family cysteine protease containing protein, expressed |
| Os.56922.1.S1_at | 2.345 | 17.509 | 6.035 | 0.675 | LOC_Os02g26430 | WRKY DNA binding domain containing protein, expressed |
| Os.1385.1.S1_at | 2.328 | 1.305 | 1.446 | 1.301 | LOC_Os01g71670 | Glucan endo-1,3-beta-glucosidase GII precursor, putative, expressed |
| Os.3406.1.S1_at | 2.321 | 4.373 | 7.963 | 2.410 | LOC_Os04g51160 | transposon protein, putative, unclassified, expressed |
| Os.54768.1.S1_at | 2.315 | 1.184 | 1.159 | 1.599 | LOC_Os12g43970 | hydrolase, alpha/beta fold family protein, expressed |

| ProbeSetID | *OMTN4* -OE[a] | Stress response[b] | | | TIGR Locus ID[c] | Annotation |
|---|---|---|---|---|---|---|
| | | Drought | Salt | Cold | | |
| Os.9103.1.S1_at | 2.302 | 8.130 | 3.924 | 1.551 | LOC_Os09g09930 | heavy metal-associated domain containing protein, expressed |
| Os.54927.1.S1_at | 2.267 | 1.374 | 1.922 | 0.922 | LOC_Os08g44590 | oxidoreductase, 2OG-Fe oxygenase family protein, expressed |
| Os.26922.1.S1_at | 2.266 | 0.897 | 1.236 | 0.792 | LOC_Os02g04170 | L-aspartate oxidase family protein, expressed |
| Os.51741.1.S1_at | 2.264 | 12.469 | 3.845 | 2.271 | LOC_Os05g25920 | hypothetical protein |
| Os.48076.1.S1_at | 2.256 | 1.432 | 1.176 | 0.762 | LOC_Os12g36750 | expressed protein |
| Os.28011.1.S1_at | 2.236 | 0.187 | 0.528 | 1.247 | LOC_Os06g29730 | expressed protein |
| Os.25171.1.A1_at | 2.232 | 1.295 | 0.534 | 0.370 | LOC_Os11g47140 | Protein kinase domain containing protein, expressed |
| Os.51323.1.S1_at | 2.224 | 0.256 | 1.793 | 1.220 | LOC_Os12g14540 | expressed protein |
| Os.50015.1.S1_at | 2.208 | 5.523 | 21.830 | 5.004 | LOC_Os06g44010 | WRKY2 protein, putative, expressed |
| Os.4773.1.S1_at | 2.207 | 6.292 | 2.635 | 2.744 | LOC_Os06g04240 | expressed protein |
| OsAffx.13358.1.S1_at | 2.169 | 6.082 | 2.242 | 3.731 | LOC_Os03g45180 | expressed protein |
| Os.53660.1.S1_at | 2.159 | 3.266 | 7.603 | 5.744 | LOC_Os02g43790 | AP2 domain containing protein, expressed |
| Os.54370.1.S1_at | 2.144 | 3.608 | 3.452 | 1.166 | LOC_Os03g48750 | Oxalate oxidase GF-2.8 precursor, putative, expressed |
| Os.11065.1.A1_at | 2.142 | 1.191 | 0.512 | 1.153 | LOC_Os03g19600 | retrotransposon protein, putative, Ty3-gypsy subclass, expressed |
| Os.29825.1.S1_a_at | 2.137 | 1.318 | 2.346 | 1.197 | LOC_Os08g38710 | Raffinose synthase or seed imbibition protein Sip1 containing protein, expressed |
| Os.50470.1.S1_at | 2.136 | 3.270 | 4.170 | 0.793 | LOC_Os01g71310 | Cytokinin dehydrogenase 1 precursor, putative, expressed |
| Os.12286.1.S1_at | 2.135 | 0.642 | 1.025 | 1.633 | LOC_Os03g52690 | CBS domain containing protein, expressed |
| Os.22967.1.S1_s_at | 2.134 | 1.012 | 1.124 | 1.000 | LOC_Os03g02070 | heavy-metal-associated domain-containing protein, putative, expressed |
| OsAffx.20788.1.S1_at | 2.116 | 0.296 | 1.775 | 0.676 | LOC_Os10g02970 | Leucine Rich Repeat family protein |
| Os.50590.2.A1_at | 2.115 | 0.307 | 0.696 | 1.025 | LOC_Os02g07830 | Cation transport protein, expressed |
| OsAffx.16322.1.S1_at | 2.097 | 2.255 | 0.436 | 4.782 | LOC_Os07g18240 | lectin protein kinase, putative, expressed |
| Os.606.1.S1_at | 2.097 | 9.617 | 11.730 | 5.006 | LOC_Os06g04220 | expressed protein |
| Os.40473.1.S1_at | 2.083 | 0.484 | 0.444 | 1.173 | LOC_Os07g02660 | NB-ARC domain containing protein |

| ProbeSetID | *OMTN4* -OE[a] | Stress response[b] | | | TIGR Locus ID[c] | Annotation |
|---|---|---|---|---|---|---|
| | | Drought | Salt | Cold | | |
| OsAffx.25720.1.S1_at | 2.077 | 0.814 | 1.029 | 0.886 | LOC_Os03g55460 | expressed protein |
| OsAffx.21914.2.S1_at | 2.074 | 0.802 | 1.372 | 0.570 | LOC_Os01g08630 | expressed protein |
| OsAffx.29181.1.S1_at | 2.064 | 2.395 | 3.342 | 3.237 | LOC_Os08g13870 | D-mannose binding lectin family protein |
| Os.6269.1.S1_at | 2.044 | 1.635 | 0.880 | 0.676 | LOC_Os03g19090 | expressed protein |
| Os.15152.2.S1_at | 2.039 | 2.649 | 2.715 | 1.111 | LOC_Os05g45350 | DnaJ domain containing protein, expressed |
| Os.4801.1.S1_x_at | 2.012 | 0.793 | 0.818 | 0.881 | LOC_Os01g52010 | Allinase, C-terminal domain containing protein, expressed |
| Os.12043.1.S1_at | 2.005 | 2.868 | 6.642 | 0.974 | LOC_Os01g44120 | expressed protein |
| OsAffx.11788.1.S1_at | 0.072 | 0.376 | 0.313 | 0.649 | LOC_Os01g68460 | DC1 domain-containing protein, putative, expressed |
| OsAffx.17366.1.S1_at | 0.090 | 2.606 | 5.273 | 4.973 | LOC_Os08g36920 | AP2 domain containing protein, expressed |
| Os.54961.1.S1_at | 0.120 | 5.200 | 1.663 | 2.109 | LOC_Os05g46350 | IQ calmodulin-binding motif family protein, expressed |
| Os.12452.1.S1_s_at | 0.138 | 7.189 | 4.123 | 1.011 | LOC_Os08g36910 | Alpha-amylase isozyme 3D precursor, putative, expressed |
| Os.18463.1.S1_at | 0.139 | 9.807 | 2.097 | 5.040 | LOC_Os05g37660 | AER, putative, expressed |
| Os.6043.1.S1_at | 0.143 | 2.123 | 3.429 | 2.395 | LOC_Os04g23550 | Helix-loop-helix DNA-binding domain containing protein, expressed |
| Os.49634.1.S1_x_at | 0.145 | 2.255 | 3.515 | 5.648 | LOC_Os01g55510 | Dynein light chain type 1 family protein, expressed |
| Os.54944.1.S1_at | 0.153 | 28.688 | 19.029 | 10.857 | LOC_Os02g52670 | AP2 domain containing protein, expressed |
| Os.15633.1.S1_at | 0.160 | 4.191 | 1.939 | 1.984 | LOC_Os10g35460 | COBRA-like protein 2 precursor, putative, expressed |
| OsAffx.17348.1.S1_at | 0.162 | 0.699 | 0.928 | 2.325 | LOC_Os08g35590 | hypothetical protein |
| Os.53407.1.S1_at | 0.168 | 3.573 | 1.288 | 0.763 | LOC_Os08g06210 | expressed protein |
| Os.15706.1.S1_at | 0.168 | 1.538 | 0.523 | 2.396 | LOC_Os01g47580 | Lipid phosphate phosphatase 2, putative, expressed |
| OsAffx.12733.1.S1_at | 0.172 | 1.472 | 1.239 | 3.033 | LOC_Os03g04740 | expressed protein |
| Os.11812.1.S1_at | 0.172 | 8.155 | 0.733 | 1.271 | LOC_Os02g48770 | SAM dependent carboxyl methyltransferase family protein, expressed |
| Os.12201.2.S1_at | 0.175 | 2.702 | 2.693 | 0.977 | LOC_Os09g27820 | 1-aminocyclopropane-1-carboxylate oxidase 1, putative, expressed |
| Os.14616.1.S1_at | 0.177 | 3.049 | 0.953 | 1.947 | LOC_Os07g34260 | Chalcone and stilbene synthases, N-terminal domain containing protein, expressed |

| ProbeSetID | *OMTN4* -OE[a] | Stress response[b] | | | TIGR Locus ID[c] | Annotation |
|---|---|---|---|---|---|---|
| | | Drought | Salt | Cold | | |
| OsAffx.24782.1.S1_s_at | 0.179 | 3.917 | 1.965 | 2.304 | LOC_Os02g48320 | DNA-binding protein, putative, expressed |
| OsAffx.14201.1.S1_at | 0.183 | 0.337 | 0.136 | 0.472 | LOC_Os04g39360 | heavy metal-associated domain containing protein, expressed |
| Os.50449.1.S1_at | 0.188 | 2.270 | 0.885 | 1.655 | LOC_Os03g10640 | Calcium-transporting ATPase 2, plasma membrane-type, putative, expressed |
| Os.56018.1.S1_at | 0.190 | 3.679 | 1.363 | 3.818 | LOC_Os05g44060 | expressed protein |
| Os.50175.2.S1_at | 0.199 | 1.590 | 2.039 | 0.585 | LOC_Os04g51820 | Cation transport protein, expressed |
| Os.32455.1.S1_at | 0.202 | 1.276 | 0.980 | 1.156 | LOC_Os01g54670 | expressed protein |
| Os.51757.1.S1_at | 0.203 | 1.405 | 1.042 | 0.813 | LOC_Os06g26270 | expressed protein |
| Os.11639.1.S1_at | 0.206 | 2.396 | 1.256 | 1.777 | LOC_Os06g05130 | Myristoyl-acyl carrier protein thioesterase, chloroplast precursor, putative, expressed |
| Os.15701.1.S1_x_at | 0.207 | 0.893 | 1.201 | 1.459 | LOC_Os07g47350 | Potassium transporter 7, putative, expressed |
| OsAffx.26389.1.S1_at | 0.208 | 0.619 | 0.619 | 0.952 | LOC_Os04g39300 | heavy metal-associated domain containing protein, expressed |
| OsAffx.22469.1.S1_x_at | 0.214 | 3.843 | 2.093 | 2.284 | LOC_Os07g07040 | expressed protein |
| Os.52661.1.S1_at | 0.217 | 1.003 | 0.490 | 3.697 | LOC_Os11g44680 | expressed protein |
| Os.27494.1.S1_at | 0.217 | 0.474 | 0.665 | 1.051 | LOC_Os07g41200 | expressed protein |
| Os.15706.1.S1_a_at | 0.220 | 1.408 | 0.692 | 1.753 | LOC_Os01g47580 | Lipid phosphate phosphatase 2, putative, expressed |
| Os.28301.1.S1_at | 0.221 | 0.225 | 0.520 | 1.377 | LOC_Os07g05360 | Photosystem II 10 kDa polypeptide, chloroplast precursor, putative, expressed |
| Os.14413.1.S1_at | 0.222 | 0.348 | 0.366 | 1.939 | LOC_Os07g48340 | Calcium-binding protein CAST, putative, expressed |
| Os.10358.1.A1_at | 0.230 | 0.277 | 0.575 | 0.586 | LOC_Os10g04270 | Jacalin-like lectin domain containing protein, expressed |
| Os.27688.1.A1_at | 0.231 | 0.957 | 0.731 | 9.416 | LOC_Os04g21350 | flowering promoting factor-like 1, putative, expressed |
| Os.49322.1.S1_at | 0.231 | 0.511 | 0.660 | 2.426 | LOC_Os09g15050 | Ent-kaurene synthase A, chloroplast precursor, putative, expressed |
| OsAffx.3275.1.S1_at | 0.235 | 2.265 | 1.807 | 1.867 | LOC_Os03g19400 | expressed protein |
| Os.54453.1.S1_at | 0.237 | 0.375 | 0.219 | 1.231 | LOC_Os08g44220 | expressed protein |
| OsAffx.13360.1.S1_at | 0.254 | 3.190 | 1.047 | 3.493 | LOC_Os03g45280 | Dehydrin family protein, expressed |
| Os.8508.1.S1_at | 0.256 | 0.601 | 0.326 | 0.201 | LOC_Os10g38880 | uncharacterized plant-specific domain TIGR01568 family protein, expressed |

| ProbeSetID | *OMTN4* -OE[a] | Stress response[b] | | | TIGR Locus ID[c] | Annotation |
|---|---|---|---|---|---|---|
| | | Drought | Salt | Cold | | |
| Os.12724.1.S1_a_at | 0.256 | 2.123 | 2.034 | 1.030 | LOC_Os08g36320 | Glutamate decarboxylase, putative, expressed |
| Os.55408.1.S1_at | 0.257 | 88.632 | 13.552 | 9.601 | LOC_Os04g52750 | expressed protein |
| Os.27019.1.S1_at | 0.258 | 3.466 | 2.619 | 0.967 | LOC_Os04g43410 | Glycosyl hydrolase family 1 protein, expressed |
| Os.22731.1.S1_at | 0.259 | 9.240 | 14.323 | 0.888 | LOC_Os03g16020 | 17.4 kDa class I heat shock protein, putative, expressed |
| Os.2371.1.S1_at | 0.262 | 2.412 | 1.820 | 1.596 | LOC_Os12g26290 | alpha-dioxygenase, putative, expressed |
| Os.27011.1.A1_s_at | 0.263 | 0.551 | 0.802 | 1.551 | LOC_Os01g04550 | serine/threonine protein kinase, putative, expressed |
| OsAffx.3920.1.S1_s_at | 0.263 | 0.214 | 1.854 | 1.961 | LOC_Os04g27670 | Terpene synthase family, metal binding domain containing protein, expressed |
| OsAffx.7506.1.S1_at | 0.265 | 1.073 | 0.938 | 1.741 | LOC_Os12g06780 | expressed protein |
| Os.17805.1.S1_at | 0.266 | 8.455 | 3.544 | 1.548 | LOC_Os01g63930 | Cytochrome P450 family protein, expressed |
| Os.9199.1.S1_at | 0.266 | 4.709 | 1.450 | 1.368 | LOC_Os06g50330 | senescence/dehydration-associated protein, putative, expressed |
| Os.25606.1.S1_at | 0.267 | 1.476 | 0.793 | 1.850 | LOC_Os09g25060 | WRKY DNA binding domain containing protein, expressed |
| Os.53422.1.S1_at | 0.267 | 0.398 | 1.102 | 0.766 | LOC_Os02g15460 | expressed protein |
| Os.2617.2.S1_a_at | 0.267 | 0.319 | 0.830 | 0.276 | LOC_Os06g48810 | high-affinity sodium transporter, putative, expressed |
| Os.12642.1.S1_at | 0.276 | 8.266 | 2.362 | 5.968 | LOC_Os08g42750 | Calcium-dependent protein kinase, isoform AK1, putative, expressed |
| Os.7862.1.S1_at | 0.277 | 7.040 | 1.999 | 4.019 | LOC_Os03g13840 | Senescence-associated protein, expressed |
| OsAffx.6035.1.S1_at | 0.283 | 1.662 | 0.441 | 13.993 | LOC_Os08g35580 | expressed protein |
| OsAffx.12721.1.S1_at | 0.286 | 2.570 | 1.367 | 1.457 | LOC_Os03g03510 | CIPK-like protein 1, putative, expressed |
| Os.14938.1.S1_at | 0.287 | 2.039 | 0.863 | 1.882 | LOC_Os05g01140 | SAM dependent carboxyl methyltransferase family protein, expressed |
| Os.55991.1.S1_at | 0.287 | 5.346 | 3.803 | 2.449 | LOC_Os10g39090 | expressed protein |
| OsAffx.23406.1.S1_at | 0.294 | 0.836 | 0.954 | 1.289 | LOC_Os01g26300 | wall-associated kinase 1, putative, expressed |
| Os.28030.1.S1_s_at | 0.295 | 0.920 | 1.569 | 2.386 | LOC_Os06g48160 | xyloglucan endotransglucosylase/hydrolase protein 15precursor, putative, expressed |
| Os.9154.1.S1_at | 0.296 | 2.221 | 2.133 | 2.876 | LOC_Os01g56220 | expressed protein |
| Os.50575.1.S1_at | 0.306 | 2.252 | 1.239 | 1.780 | LOC_Os04g40700 | expressed protein |

| ProbeSetID | *OMTN4* -OE[a] | Stress response[b] | | | TIGR Locus ID[c] | Annotation |
|---|---|---|---|---|---|---|
| | | Drought | Salt | Cold | | |
| OsAffx.7606.1.S1_at | 0.307 | 1.419 | 4.132 | 1.940 | LOC_Os12g15680 | Multicopper oxidase family protein, expressed |
| Os.37213.1.S1_at | 0.307 | 0.469 | 0.502 | 1.640 | LOC_Os07g29310 | Auxin responsive protein, expressed |
| Os.57426.1.S1_x_at | 0.311 | 2.820 | 1.613 | 1.743 | LOC_Os05g13940 | retrotransposon protein, putative, Ty3-gypsy subclass, expressed |
| Os.8957.1.S1_at | 0.312 | 8.261 | 2.533 | 2.136 | LOC_Os01g63060 | phosphatidic acid phosphatase, putative, expressed |
| Os.36767.1.S1_at | 0.313 | 4.575 | 2.087 | 2.917 | LOC_Os01g04330 | EF hand family protein, expressed |
| Os.47945.1.A1_at | 0.314 | 0.810 | 0.490 | 0.792 | LOC_Os06g37510 | senescence-associated protein, putative, expressed |
| OsAffx.14202.1.S1_at | 0.315 | 0.158 | 0.157 | 0.266 | LOC_Os04g39380 | expressed protein |
| Os.11309.1.S1_x_at | 0.317 | 1.181 | 0.961 | 0.901 | LOC_Os05g04490 | Peroxidase family protein, expressed |
| Os.37773.1.S1_at | 0.318 | 1.643 | 7.595 | 2.434 | LOC_Os03g16030 | 17.4 kDa class I heat shock protein, putative, expressed |
| Os.2225.1.S1_at | 0.321 | 2.697 | 1.320 | 1.242 | LOC_Os04g44870 | Elicitor-responsive protein 3, putative, expressed |
| Os.14285.2.S1_at | 0.323 | 0.406 | 0.511 | 1.465 | LOC_Os04g31550 | expressed protein |
| Os.56988.1.S1_at | 0.326 | 1.338 | 0.632 | 1.368 | LOC_Os03g53730 | flavodoxin family protein, expressed |
| Os.54385.1.S1_at | 0.326 | 0.889 | 0.715 | 1.847 | LOC_Os04g01320 | D-mannose binding lectin family protein, expressed |
| Os.10416.1.S1_at | 0.327 | 0.924 | 0.725 | 2.495 | LOC_Os01g61850 | expressed protein |
| Os.17900.1.S1_s_at | 0.329 | 0.738 | 0.940 | 0.174 | LOC_Os05g35690 | Gibberellin-regulated protein 2 precursor, putative, expressed |
| Os.13708.1.S1_at | 0.330 | 1.034 | 0.643 | 1.287 | LOC_Os03g14250 | expressed protein |
| Os.53009.1.A1_x_at | 0.331 | 0.078 | 0.444 | 0.883 | LOC_Os02g54640 | Receptor family ligand binding region containing protein, expressed |
| Os.820.1.S1_s_at | 0.332 | 1.069 | 0.868 | 1.220 | LOC_Os01g19020 | Peroxidase family protein, expressed |
| Os.2211.1.S1_at | 0.335 | 0.568 | 0.573 | 1.727 | LOC_Os08g09010 | Germin-like protein subfamily 1 member 11 precursor, putative, expressed |
| OsAffx.14888.1.S1_at | 0.335 | 1.859 | 0.313 | 1.868 | LOC_Os05g30500 | expressed protein |
| Os.37798.1.S1_at | 0.337 | 2.318 | 2.725 | 1.274 | LOC_Os01g11460 | Zinc finger, C3HC4 type family protein, expressed |
| Os.11287.1.S1_at | 0.337 | 3.770 | 2.584 | 1.577 | LOC_Os06g50930 | Senescence-associated protein DIN1, putative, expressed |
| Os.25316.2.S1_x_at | 0.337 | 1.367 | 1.161 | 0.853 | LOC_Os03g56260 | Kinesin motor domain containing protein, expressed |
| OsAffx.11789.1.S1_s_at | 0.337 | 0.092 | 0.083 | 0.712 | LOC_Os01g68470 | hypothetical protein |

| ProbeSetID | *OMTN4* -OE[a] | Stress response[b] | | | TIGR Locus ID[c] | Annotation |
|---|---|---|---|---|---|---|
| | | Drought | Salt | Cold | | |
| Os.6345.1.S1_at | 0.338 | 0.701 | 0.583 | 1.223 | LOC_Os06g36560 | Inositol oxygenase, putative, expressed |
| OsAffx.15485.1.S1_at | 0.338 | 0.729 | 0.585 | 1.585 | LOC_Os06g18900 | embryogenesis transmembrane protein, putative, expressed |
| Os.9456.1.S1_at | 0.340 | 4.517 | 1.775 | 1.354 | LOC_Os02g28170 | Transferase family protein, expressed |
| OsAffx.3951.1.S1_at | 0.341 | 0.702 | 0.226 | 0.806 | LOC_Os04g30340 | wall-associated kinase 2, putative |
| Os.55254.1.S1_at | 0.341 | 1.036 | 1.313 | 1.133 | LOC_Os06g10130 | expressed protein |
| OsAffx.19473.1.S1_at | 0.341 | 1.539 | 1.309 | 1.893 | LOC_Os12g03740 | F-box domain containing protein, expressed |
| Os.5905.1.S1_at | 0.343 | 0.897 | 0.223 | 0.877 | LOC_Os06g32990 | Peroxidase family protein, expressed |
| Os.26543.1.S1_at | 0.344 | 0.891 | 0.397 | 1.177 | LOC_Os03g58110 | Leucine Rich Repeat family protein, expressed |
| Os.26938.1.A1_at | 0.346 | 0.149 | 0.409 | 0.701 | LOC_Os02g42150 | Calcium binding EGF domain containing protein, expressed |
| Os.7348.1.S1_at | 0.347 | 3.093 | 1.127 | 6.184 | LOC_Os08g38910 | O-methyltransferase family protein, expressed |
| Os.32801.1.S1_x_at | 0.347 | 1.697 | 0.949 | 2.881 | LOC_Os01g54700 | transposon protein, putative, CACTA, En/Spm sub-class, expressed |
| Os.27553.2.S1_at | 0.348 | 0.950 | 0.489 | 0.211 | LOC_Os07g27300 | RNA-binding protein Luc7-like 1, putative, expressed |
| Os.6092.1.S1_at | 0.349 | 1.773 | 0.494 | 7.560 | LOC_Os02g44230 | trehalose-phosphatase family protein, expressed |
| Os.25556.1.S1_x_at | 0.349 | 0.398 | 0.448 | 0.856 | LOC_Os08g28400 | expressed protein |
| Os.51546.1.S1_at | 0.352 | 0.960 | 3.024 | 3.429 | LOC_Os03g08520 | expressed protein |
| Os.1978.1.S1_at | 0.353 | 1.649 | 3.001 | 1.492 | LOC_Os01g02290 | Ser/Thr receptor-like kinase, putative, expressed |
| Os.32267.1.S1_at | 0.353 | 1.583 | 0.967 | 1.346 | LOC_Os01g56330 | protein kinase family protein, putative, expressed |
| Os.54269.1.S1_s_at | 0.355 | 2.176 | 1.297 | 1.255 | LOC_Os05g48060 | Phosphatidyl serine synthase family protein, expressed |
| Os.6042.1.S1_at | 0.357 | 4.173 | 1.419 | 0.716 | LOC_Os07g25810 | transposon protein, putative, CACTA, En/Spm sub-class, expressed |
| Os.26411.1.A1_at | 0.357 | 38.364 | 20.293 | 2.357 | LOC_Os08g28710 | Protein kinase domain containing protein, expressed |
| Os.33316.1.S1_at | 0.357 | 3.624 | 0.960 | 1.115 | LOC_Os01g34560 | very-long-chain fatty acid condensing enzyme, putative, expressed |
| Os.17112.1.S1_at | 0.359 | 15.064 | 12.497 | 1.767 | LOC_Os03g18030 | oxidoreductase, 2OG-Fe oxygenase family protein, expressed |
| Os.9514.1.S1_at | 0.359 | 0.526 | 0.736 | 1.181 | LOC_Os05g04820 | Myb-like DNA-binding domain containing protein, expressed |
| Os.9533.1.S1_at | 0.361 | 0.949 | 1.477 | 1.911 | LOC_Os03g02750 | Subtilisin N-terminal Region family protein, expressed |

| ProbeSetID | *OMTN4* -OE[a] | Stress response[b] | | | TIGR Locus ID[c] | Annotation |
|---|---|---|---|---|---|---|
| | | Drought | Salt | Cold | | |
| Os.51246.1.S1_at | 0.362 | 0.193 | 0.193 | 1.209 | LOC_Os11g11780 | serine/threonine protein kinase, putative, expressed |
| OsAffx.1083.1.S1_at | 0.365 | 1.281 | 0.439 | 1.302 | LOC_Os01g66840 | Pectinacetylesterase family protein, expressed |
| OsAffx.2803.1.S1_at | 0.366 | 0.439 | 1.314 | 1.029 | LOC_Os02g30190 | hypothetical protein |
| Os.50346.1.S1_at | 0.366 | 3.223 | 1.628 | 1.876 | LOC_Os05g04680 | expressed protein |
| OsAffx.15812.1.S1_at | 0.366 | 1.455 | 1.000 | 1.182 | LOC_Os06g38120 | expressed protein |
| OsAffx.30204.1.S1_at | 0.366 | 1.521 | 0.827 | 1.311 | LOC_Os09g39190 | Copine family protein, expressed |
| OsAffx.29016.1.S1_at | 0.367 | 1.325 | 1.152 | 1.424 | LOC_Os08g03600 | magnesium transporter CorA, putative, expressed |
| Os.8202.1.S1_at | 0.367 | 2.181 | 1.704 | 1.680 | LOC_Os12g37560 | phospholipase C, putative, expressed |
| Os.39087.1.S1_at | 0.368 | 1.762 | 1.344 | 1.987 | LOC_Os01g14550 | Pathogen-related protein, putative, expressed |
| Os.51227.1.S1_x_at | 0.368 | 1.487 | 0.690 | 1.205 | LOC_Os04g27790 | Terpene synthase family, metal binding domain containing protein, expressed |
| Os.8597.1.S1_at | 0.370 | 0.479 | 0.516 | 1.271 | LOC_Os03g02040 | Remorin, C-terminal region family protein, expressed |
| Os.50455.1.S1_at | 0.371 | 3.019 | 0.924 | 3.068 | LOC_Os06g40170 | Phospholipase D alpha 2, putative, expressed |
| Os.53887.2.S1_x_at | 0.373 | 6.939 | 1.310 | 1.432 | LOC_Os07g49480 | expressed protein |
| Os.30909.1.S1_at | 0.374 | 0.504 | 1.065 | 0.187 | LOC_Os01g09190 | expressed protein |
| Os.26382.1.S1_at | 0.374 | 0.869 | 0.811 | 1.059 | LOC_Os05g12680 | Protein kinase domain containing protein, expressed |
| Os.10245.1.S1_at | 0.375 | 1.135 | 2.508 | 1.681 | LOC_Os04g37700 | expressed protein |
| Os.3721.1.S1_at | 0.375 | 1.451 | 1.140 | 1.466 | LOC_Os01g03980 | expressed protein |
| OsAffx.17220.1.S1_at | 0.377 | 3.300 | 3.450 | 0.550 | LOC_Os08g29040 | Protein kinase domain containing protein |
| Os.30572.1.S1_at | 0.378 | 1.216 | 0.811 | 0.634 | LOC_Os01g50610 | SAM dependent carboxyl methyltransferase family protein, expressed |
| Os.50399.1.S1_at | 0.380 | 74.437 | 31.901 | 1.586 | LOC_Os06g05420 | expressed protein |
| Os.23977.1.S1_at | 0.381 | 0.378 | 0.538 | 0.763 | LOC_Os03g43510 | expressed protein |
| Os.48082.1.S1_at | 0.382 | 1.241 | 4.215 | 1.427 | LOC_Os09g25070 | WRKY DNA binding domain containing protein, expressed |
| Os.36283.1.S1_at | 0.382 | 1.166 | 1.408 | 0.410 | LOC_Os12g25090 | expressed protein |
| Os.12387.1.S1_at | 0.382 | 1.465 | 1.074 | 1.064 | LOC_Os04g39150 | Pathogenesis-related protein Bet v I family protein, expressed |

| ProbeSetID | *OMTN4* -OE[a] | Stress response[b] | | | TIGR Locus ID[c] | Annotation |
|---|---|---|---|---|---|---|
| | | Drought | Salt | Cold | | |
| Os.7507.1.S1_at | 0.383 | 3.691 | 4.096 | 0.682 | LOC_Os10g02880 | O-methyltransferase family protein, expressed |
| Os.12421.1.S1_at | 0.385 | 0.378 | 0.265 | 0.724 | LOC_Os11g42960 | plant integral membrane protein TIGR01569 containing protein, expressed |
| Os.28200.1.S1_x_at | 0.386 | 24.253 | 27.402 | 8.330 | LOC_Os03g61160 | expressed protein |
| OsAffx.13276.1.S1_at | 0.386 | 0.724 | 0.761 | 1.192 | LOC_Os03g40930 | Lipase, putative, expressed |
| Os.20557.1.S1_s_at | 0.386 | 8.690 | 2.881 | 1.250 | LOC_Os11g10760 | Leucine Rich Repeat family protein, expressed |
| Os.51866.1.S1_at | 0.387 | 9.248 | 5.017 | 2.709 | LOC_Os11g10770 | NB-ARC domain containing protein, expressed |
| Os.57191.1.S1_at | 0.387 | 16.608 | 9.364 | 4.114 | LOC_Os06g05470 | expressed protein |
| Os.57316.1.S1_at | 0.387 | 1.357 | 2.739 | 0.792 | LOC_Os05g38940 | expressed protein |
| Os.9344.1.S1_x_at | 0.387 | 1.944 | 1.581 | 1.546 | LOC_Os03g53800 | Glycosyl hydrolase family 3 N terminal domain containing protein, expressed |
| OsAffx.7406.1.S1_at | 0.391 | 0.116 | 0.353 | 0.340 | LOC_Os11g44300 | hypothetical protein |
| OsAffx.22380.1.S1_at | 0.392 | 1.580 | 0.910 | 3.608 | LOC_Os07g39680 | XYPPX repeat family protein, expressed |
| Os.52699.1.S1_at | 0.393 | 0.928 | 0.737 | 1.267 | LOC_Os04g55420 | Leucine Rich Repeat family protein, expressed |
| Os.12201.1.S1_at | 0.393 | 2.447 | 1.470 | 1.403 | LOC_Os09g27750 | 1-aminocyclopropane-1-carboxylate oxidase 1, putative, expressed |
| Os.10391.1.S1_a_at | 0.394 | 3.638 | 1.705 | 1.249 | LOC_Os05g51630 | early-responsive to dehydration protein, putative, expressed |
| Os.47625.1.A1_s_at | 0.397 | 0.956 | 0.614 | 1.078 | LOC_Os05g04500 | Peroxidase family protein, expressed |
| Os.22839.1.S1_at | 0.397 | 5.327 | 3.747 | 2.828 | LOC_Os06g48200 | xyloglucan endotransglucosylase/hydrolase protein 15precursor, putative, expressed |
| Os.6418.1.S1_at | 0.399 | 9.068 | 3.843 | 0.848 | LOC_Os01g07530 | Raffinose synthase or seed imbibition protein Sip1 containing protein, expressed |
| Os.27797.1.A1_at | 0.399 | 2.723 | 2.578 | 1.053 | LOC_Os03g06570 | IQ calmodulin-binding motif family protein, expressed |
| Os.50572.1.S1_at | 0.399 | 1.092 | 0.941 | 1.701 | LOC_Os12g36920 | calmodulin-binding protein, putative, expressed |
| OsAffx.8290.1.S1_at | 0.400 | 1.580 | 1.037 | 0.370 | LOC_Os10g17960 | receptor-like protein kinase homolog RK20-1, putative, expressed |
| Os.50799.1.S1_at | 0.401 | 0.588 | 0.375 | 0.816 | LOC_Os04g48130 | membrane protein, putative, expressed |
| Os.10583.1.S1_at | 0.401 | 0.219 | 0.243 | 0.832 | LOC_Os06g04920 | Zn-finger in Ran binding protein and others containing protein, expressed |

| ProbeSetID | *OMTN4* -OE[a] | Stress response[b] | | | TIGR Locus ID[c] | Annotation |
|---|---|---|---|---|---|---|
| | | Drought | Salt | Cold | | |
| Os.15872.1.S1_at | 0.402 | 1.094 | 0.920 | 1.450 | LOC_Os05g50710 | Late embryogenesis abundant protein Lea14-A, putative, expressed |
| Os.11322.1.S1_at | 0.402 | 1.168 | 0.685 | 1.311 | LOC_Os04g50950 | POT family protein, expressed |
| Os.31858.1.S1_at | 0.403 | 4.131 | 1.885 | 2.555 | LOC_Os07g35340 | Protein kinase domain containing protein, expressed |
| Os.46849.1.S1_at | 0.403 | 5.601 | 6.351 | 5.057 | LOC_Os10g25230 | ZIM motif family protein, expressed |
| Os.56918.1.S1_at | 0.403 | 0.691 | 0.833 | 3.404 | LOC_Os03g62330 | expressed protein |
| Os.9841.1.S1_at | 0.405 | 2.585 | 1.060 | 1.328 | LOC_Os03g10390 | expressed protein |
| Os.21634.1.S1_at | 0.405 | 9.253 | 5.817 | 2.315 | LOC_Os01g55240 | Gibberellin 2-beta-dioxygenase, putative, expressed |
| OsAffx.21092.2.S1_at | 0.405 | 0.326 | 0.874 | 0.407 | LOC_Os01g26310 | expressed protein |
| OsAffx.4214.1.S1_x_at | 0.405 | 0.691 | 0.865 | 1.070 | LOC_Os04g59330 | expressed protein |
| Os.11354.1.S1_at | 0.405 | 1.361 | 0.188 | 7.684 | LOC_Os04g51460 | xyloglucan endotransglucosylase/hydrolase protein 15precursor, putative, expressed |
| Os.54555.1.S1_at | 0.406 | 1.542 | 0.910 | 1.165 | LOC_Os05g07060 | Fasciclin domain containing protein, expressed |
| Os.34779.1.S1_at | 0.406 | 0.839 | 2.607 | 0.607 | LOC_Os01g06520 | Leucine Rich Repeat family protein, expressed |
| Os.15537.1.S1_at | 0.408 | 2.509 | 1.547 | 0.509 | LOC_Os08g39660 | Cytochrome P450 family protein, expressed |
| Os.9046.1.S1_x_at | 0.408 | 2.156 | 1.676 | 0.986 | LOC_Os10g22980 | Leucine Rich Repeat family protein, expressed |
| Os.15295.2.S1_x_at | 0.408 | 1.727 | 1.105 | 3.192 | LOC_Os08g19670 | expressed protein |
| Os.11575.1.S1_a_at | 0.408 | 0.777 | 0.597 | 1.260 | LOC_Os03g46440 | BTB/POZ domain containing protein, expressed |
| Os.27420.2.A1_s_at | 0.408 | 0.317 | 0.899 | 1.096 | LOC_Os05g17810 | SHR5-receptor-like kinase, putative |
| Os.49208.1.S1_at | 0.408 | 0.595 | 0.243 | 1.681 | LOC_Os04g34270 | D-mannose binding lectin family protein, expressed |
| Os.49496.1.S1_at | 0.408 | 0.474 | 0.540 | 0.970 | LOC_Os06g46500 | L-ascorbate oxidase homolog precursor, putative, expressed |
| Os.15917.1.S1_at | 0.409 | 4.545 | 0.675 | 5.079 | LOC_Os04g41960 | NADP-dependent oxidoreductase P1, putative, expressed |
| Os.3391.1.S1_at | 0.410 | 4.220 | 4.324 | 0.544 | LOC_Os09g23620 | typical P-type R2R3 Myb protein, putative, expressed |
| Os.9511.1.S1_at | 0.410 | 0.904 | 0.686 | 1.889 | LOC_Os03g40670 | Glycerophosphoryl diester phosphodiesterase family protein, expressed |
| Os.53726.1.S1_at | 0.412 | 5.651 | 4.368 | 0.765 | LOC_Os07g05370 | protein kinase family protein, putative, expressed |

| ProbeSetID | *OMTN4* -OE[a] | Stress response[b] | | | TIGR Locus ID[c] | Annotation |
|---|---|---|---|---|---|---|
| | | Drought | Salt | Cold | | |
| Os.10054.1.S1_at | 0.412 | 2.876 | 0.768 | 1.099 | LOC_Os02g46650 | Ubiquitin carboxyl-terminal hydrolase family protein, expressed |
| Os.10546.1.S1_s_at | 0.413 | 5.328 | 0.988 | 1.402 | LOC_Os09g34230 | UDP-glucoronosyl and UDP-glucosyl transferase family protein, expressed |
| Os.5299.2.S1_a_at | 0.414 | 1.548 | 1.526 | 1.417 | LOC_Os05g37970 | universal stress protein family protein, expressed |
| OsAffx.5607.1.S1_x_at | 0.414 | 11.223 | 3.489 | 1.433 | LOC_Os07g42280 | von Willebrand factor type A domain containing protein, expressed |
| Os.12664.1.S1_at | 0.416 | 0.531 | 0.499 | 0.874 | LOC_Os07g35810 | Protein kinase domain containing protein, expressed |
| Os.27509.1.S1_at | 0.416 | 1.075 | 1.417 | 1.319 | LOC_Os01g53790 | expressed protein |
| Os.4780.1.S1_at | 0.416 | 3.321 | 2.573 | 1.080 | LOC_Os03g55290 | Gibberellin-regulated protein 2 precursor, putative, expressed |
| Os.9719.1.S1_at | 0.417 | 0.500 | 0.520 | 0.701 | LOC_Os07g47460 | expressed protein |
| Os.5812.1.S1_at | 0.417 | 0.795 | 0.938 | 2.129 | LOC_Os03g20500 | F-box domain containing protein, expressed |
| Os.6075.1.S1_at | 0.418 | 3.281 | 1.502 | 1.893 | LOC_Os04g06520 | expressed protein |
| Os.55829.1.S1_at | 0.418 | 3.196 | 1.379 | 2.648 | LOC_Os05g30760 | hydrolase, alpha/beta fold family protein, expressed |
| Os.7727.1.S1_at | 0.420 | 4.679 | 3.170 | 0.994 | LOC_Os05g11910 | GDSL-like Lipase/Acylhydrolase family protein, expressed |
| Os.53673.1.S1_at | 0.420 | 2.416 | 2.377 | 2.022 | LOC_Os04g01310 | D-mannose binding lectin family protein, expressed |
| Os.6170.1.S1_at | 0.420 | 0.610 | 0.605 | 1.748 | LOC_Os02g51060 | glycosyl transferase, group 2 family protein, expressed |
| Os.10423.1.S1_at | 0.421 | 5.160 | 2.684 | 2.631 | LOC_Os07g48630 | ETHYLENE-INSENSITIVE3-like 1 protein, putative, expressed |
| Os.52147.1.S1_at | 0.423 | 3.759 | 2.718 | 0.658 | LOC_Os11g30760 | expressed protein |
| Os.55283.1.S1_at | 0.425 | 10.076 | 4.159 | 0.830 | LOC_Os05g38660 | expressed protein |
| Os.31670.2.S1_at | 0.425 | 1.196 | 0.901 | 0.994 | LOC_Os01g43330 | expressed protein |
| Os.12851.1.S1_at | 0.425 | 0.974 | 0.733 | 1.180 | LOC_Os01g14670 | Nectarin-1 precursor, putative, expressed |
| Os.57152.1.S1_at | 0.426 | 13.554 | 1.308 | 5.344 | LOC_Os02g38890 | hypothetical protein |
| Os.9388.1.S1_at | 0.426 | 4.609 | 0.968 | 1.479 | LOC_Os04g33200 | expressed protein |
| Os.14105.1.S1_at | 0.427 | 6.569 | 13.264 | 12.204 | LOC_Os11g05380 | Cytochrome P450 family protein, expressed |
| OsAffx.21802.1.S1_at | 0.427 | 0.579 | 0.706 | 0.720 | LOC_Os01g66820 | receptor-like protein kinase, putative |
| Os.26907.1.S1_at | 0.428 | 0.188 | 0.306 | 0.836 | LOC_Os02g17710 | Leucine Rich Repeat family protein, expressed |

| ProbeSetID | *OMTN4* -OE[a] | Stress response[b] | | | TIGR Locus ID[c] | Annotation |
|---|---|---|---|---|---|---|
| | | Drought | Salt | Cold | | |
| OsAffx.23641.1.S1_at | 0.428 | 0.162 | 0.413 | 3.379 | LOC_Os01g43230 | expressed protein |
| OsAffx.27743.1.S1_at | 0.429 | 0.219 | 0.094 | 3.413 | LOC_Os06g20900 | hypothetical protein |
| Os.5095.1.S1_at | 0.430 | 0.521 | 0.643 | 0.778 | LOC_Os07g40130 | transposon protein, putative, CACTA, En/Spm sub-class |
| Os.171.1.S1_at | 0.430 | 0.797 | 1.090 | 1.302 | LOC_Os01g47070 | Acidic endochitinase precursor, putative, expressed |
| Os.27705.1.S1_a_at | 0.431 | 2.323 | 2.060 | 1.361 | LOC_Os01g18290 | Helix-loop-helix DNA-binding domain containing protein, expressed |
| Os.53174.1.A1_s_at | 0.432 | 0.541 | 0.841 | 1.116 | LOC_Os02g48350 | Diacylglycerol acyltransferase family protein, expressed |
| Os.19001.1.S1_at | 0.432 | 1.622 | 1.076 | 1.040 | LOC_Os05g31890 | expressed protein |
| Os.30059.2.S1_x_at | 0.432 | 1.027 | 0.681 | 0.829 | LOC_Os01g60420 | expressed protein |
| Os.46287.1.S1_a_at | 0.434 | 2.099 | 1.490 | 2.182 | LOC_Os05g40770 | Protein kinase domain containing protein, expressed |
| Os.34496.1.S1_at | 0.434 | 8.392 | 3.355 | 0.979 | LOC_Os03g61500 | PGP224, putative, expressed |
| Os.10855.1.S1_at | 0.435 | 4.595 | 1.533 | 1.105 | LOC_Os03g56060 | glycosyl transferase, group 2 family protein, expressed |
| Os.4377.1.S1_at | 0.435 | 0.482 | 0.557 | 0.726 | LOC_Os02g46970 | 4-coumarate-CoA ligase 2, putative, expressed |
| Os.36437.1.S1_at | 0.435 | 12.977 | 2.163 | 1.058 | LOC_Os07g37920 | NAM, putative, expressed |
| Os.30376.1.S1_at | 0.435 | 1.004 | 1.603 | 1.411 | LOC_Os01g02130 | expressed protein |
| Os.53148.1.S1_at | 0.435 | 1.119 | 0.648 | 2.101 | LOC_Os03g52720 | magnesium-dependent phosphatase-1 family protein, expressed |
| Os.8859.1.S1_s_at | 0.436 | 1.236 | 0.836 | 1.797 | LOC_Os07g07990 | Leucine Rich Repeat family protein, expressed |
| OsAffx.29230.1.S1_at | 0.436 | 0.547 | 1.955 | 0.823 | LOC_Os08g16030 | expressed protein |
| Os.27673.1.S1_at | 0.438 | 1.173 | 0.750 | 1.153 | LOC_Os03g14730 | esterase, putative, expressed |
| Os.5594.1.S1_at | 0.438 | 3.823 | 2.911 | 1.173 | LOC_Os05g48340 | proteasome, putative, expressed |
| Os.53208.1.S1_at | 0.438 | 0.307 | 0.667 | 0.470 | LOC_Os09g29600 | Calcium binding EGF domain containing protein, expressed |
| Os.10185.1.S1_at | 0.439 | 1.212 | 0.952 | 0.221 | LOC_Os11g31470 | expressed protein |
| Os.37006.1.S1_at | 0.439 | 4.111 | 3.023 | 1.686 | LOC_Os06g11660 | Phosphate-induced protein 1 conserved region containing protein, expressed |
| Os.11465.1.S1_at | 0.440 | 1.392 | 0.683 | 1.075 | LOC_Os11g33270 | xyloglucan endotransglucosylase/hydrolase precursor, putative, expressed |
| Os.15803.1.S1_at | 0.440 | 0.560 | 0.634 | 1.275 | LOC_Os02g49950 | U-box domain containing protein, expressed |

| ProbeSetID | *OMTN4* -OE[a] | Stress response[b] | | | TIGR Locus ID[c] | Annotation |
|---|---|---|---|---|---|---|
| | | Drought | Salt | Cold | | |
| Os.32736.1.S1_at | 0.441 | 0.526 | 0.456 | 0.951 | LOC_Os03g56820 | Fatty acid hydroxylase family protein, expressed |
| Os.46566.1.S1_at | 0.441 | 2.732 | 4.268 | 0.687 | LOC_Os10g17940 | F-box domain containing protein |
| Os.12167.1.S1_at | 0.444 | 8.843 | 5.536 | 2.456 | LOC_Os02g44870 | Dehydrin family protein, expressed |
| Os.47445.1.S1_at | 0.445 | 1.503 | 1.340 | 1.357 | LOC_Os02g26210 | flowering promoting factor-like 1, putative, expressed |
| Os.52228.1.S1_at | 0.445 | 0.224 | 0.400 | 0.903 | LOC_Os11g36000 | Leucine Rich Repeat family protein, expressed |
| Os.54493.1.S1_at | 0.445 | 0.898 | 0.671 | 1.993 | LOC_Os06g14490 | calmodulin-binding heat-shock protein, putative, expressed |
| Os.17479.1.S1_at | 0.445 | 0.854 | 0.526 | 1.884 | LOC_Os01g49320 | Acidic endochitinase precursor, putative, expressed |
| Os.17887.1.S1_at | 0.445 | 3.302 | 1.690 | 4.523 | LOC_Os12g36910 | calmodulin-binding protein, putative, expressed |
| Os.16401.1.S2_at | 0.446 | 1.647 | 0.432 | 1.176 | LOC_Os03g21380 | EF hand family protein, expressed |
| Os.34952.1.S1_at | 0.446 | 1.481 | 1.136 | 1.623 | LOC_Os01g55610 | POT family protein, expressed |
| Os.21774.1.S1_at | 0.446 | 1.024 | 0.829 | 1.478 | LOC_Os03g18310 | 6-phosphofructo-2-kinase family protein, expressed |
| Os.15216.1.S1_at | 0.446 | 2.200 | 1.785 | 0.884 | LOC_Os05g36190 | Tubby protein, putative, expressed |
| Os.36104.1.S1_at | 0.448 | 0.397 | 0.405 | 1.280 | LOC_Os02g31030 | glycerophosphoryl diester phosphodiesterase family protein, putative, expressed |
| Os.41841.1.S1_at | 0.451 | 9.540 | 3.658 | 0.734 | LOC_Os01g52130 | sulfate transporter 3.5, putative, expressed |
| Os.49265.1.S2_a_at | 0.451 | 0.233 | 0.714 | 0.896 | LOC_Os03g61720 | Acyltransferase family protein, expressed |
| Os.12751.1.S1_at | 0.452 | 0.676 | 0.803 | 1.181 | LOC_Os02g48470 | expressed protein |
| Os.57456.1.S1_x_at | 0.453 | 2.707 | 2.378 | 1.608 | LOC_Os01g24710 | Salt stress-induced protein, putative, expressed |
| Os.11335.1.S1_at | 0.454 | 0.338 | 0.297 | 1.070 | LOC_Os04g33450 | expressed protein |
| Os.19369.1.S1_at | 0.454 | 1.610 | 1.080 | 0.969 | LOC_Os12g05990 | No apical meristem protein, expressed |
| Os.1054.1.A1_at | 0.455 | 3.199 | 2.877 | 0.650 | LOC_Os01g02900 | HGA6, putative, expressed |
| Os.49406.1.S1_at | 0.455 | 1.239 | 0.873 | 0.576 | LOC_Os02g29510 | Non-imprinted in Prader-Willi/Angelman syndrome region protein 2homolog, putative, expressed |
| Os.4651.1.S1_at | 0.455 | 17.742 | 1.580 | 3.231 | LOC_Os07g48710 | VQ motif family protein, expressed |

| ProbeSetID | *OMTN4* -OE[a] | Stress response[b] | | | TIGR Locus ID[c] | Annotation |
|---|---|---|---|---|---|---|
| | | Drought | Salt | Cold | | |
| Os.7678.1.S1_at | 0.455 | 2.222 | 2.997 | 6.049 | LOC_Os03g12500 | Cytochrome P450 74A2, putative, expressed |
| Os.2653.1.S1_at | 0.456 | 1.160 | 0.884 | 1.839 | LOC_Os10g39520 | MLO-like protein 10, putative, expressed |
| Os.50342.1.S1_at | 0.456 | 1.618 | 2.276 | 0.727 | LOC_Os04g52780 | Leucine Rich Repeat family protein, expressed |
| Os.17766.1.S1_at | 0.456 | 1.397 | 0.962 | 1.429 | LOC_Os06g12120 | BRASSINOSTEROID INSENSITIVE 1-associated receptor kinase 1 precursor, putative, expressed |
| Os.10747.1.S1_s_at | 0.456 | 4.549 | 1.871 | 1.779 | LOC_Os08g38220 | Dof domain, zinc finger family protein, expressed |
| Os.16286.1.S1_at | 0.458 | 1.171 | 0.814 | 0.854 | LOC_Os01g36070 | MtN3/saliva family protein, expressed |
| OsAffx.31963.1.S1_at | 0.458 | 0.329 | 0.553 | 0.824 | LOC_Os12g31540 | expressed protein |
| Os.32819.1.S1_at | 0.458 | 1.246 | 1.263 | 1.045 | LOC_Os07g36590 | KI domain interacting kinase 1, putative, expressed |
| Os.4159.1.S1_at | 0.458 | 0.411 | 0.406 | 0.985 | LOC_Os01g51570 | Glucan endo-1,3-beta-glucosidase GII precursor, putative, expressed |
| Os.16903.1.A1_at | 0.458 | 0.341 | 0.341 | 0.306 | LOC_Os05g06920 | RelA/SpoT containing protein, expressed |
| Os.18717.2.S1_at | 0.459 | 1.557 | 3.307 | 0.634 | LOC_Os09g28180 | D-mannose binding lectin family protein, expressed |
| Os.5194.1.S1_x_at | 0.459 | 0.462 | 0.573 | 0.925 | LOC_Os01g66110 | dehydration-responsive protein, putative, expressed |
| Os.55059.1.S1_at | 0.460 | 4.465 | 1.370 | 0.624 | LOC_Os03g53540 | expressed protein |
| Os.46563.1.S1_at | 0.461 | 1.185 | 0.876 | 0.986 | LOC_Os10g39750 | Helix-loop-helix DNA-binding domain containing protein, expressed |
| Os.12234.1.S1_s_at | 0.461 | 0.784 | 0.340 | 0.481 | LOC_Os10g40730 | Beta-expansin 1a precursor, putative, expressed |
| Os.17918.1.S1_at | 0.462 | 0.172 | 1.654 | 1.158 | LOC_Os03g16950 | 33 kDa secretory protein, putative, expressed |
| Os.2320.1.S1_at | 0.462 | 0.629 | 0.602 | 1.514 | LOC_Os08g09080 | Germin-like protein subfamily 1 member 11 precursor, putative, expressed |
| Os.34462.1.S1_at | 0.463 | 4.930 | 1.073 | 1.361 | LOC_Os01g62950 | Ras-related protein Rab11C, putative, expressed |
| Os.12165.1.S1_at | 0.463 | 1.830 | 1.422 | 0.484 | LOC_Os07g32570 | 5'-adenylylsulfate reductase 2, chloroplast precursor, putative, expressed |
| Os.10500.1.S1_at | 0.463 | 1.171 | 0.831 | 1.687 | LOC_Os05g39610 | Exo70 exocyst complex subunit family protein, expressed |
| Os.35858.1.S1_at | 0.464 | 0.730 | 0.850 | 0.904 | LOC_Os01g44110 | Protein kinase domain containing protein, expressed |
| Os.40417.1.A1_at | 0.465 | 0.550 | 0.322 | 1.073 | LOC_Os07g14740 | Harpin-induced protein 1 containing protein, expressed |
| Os.14641.1.S1_at | 0.466 | 1.226 | 0.949 | 1.302 | LOC_Os03g54100 | calcium-activated outward-rectifying potassium channel 5,chloroplast |

| ProbeSetID | *OMTN4* -OE[a] | Stress response[b] | | | TIGR Locus ID[c] | Annotation |
|---|---|---|---|---|---|---|
| | | Drought | Salt | Cold | | |
| | | | | | | precursor, putative, expressed |
| OsAffx.26488.1.S1_at | 0.466 | 0.967 | 0.629 | 1.186 | LOC_Os04g46730 | Pollen thioesterase, putative, expressed |
| Os.56189.1.S1_at | 0.467 | 2.419 | 2.058 | 1.054 | LOC_Os07g41590 | expressed protein |
| Os.10861.1.S1_at | 0.467 | 2.865 | 1.730 | 3.323 | LOC_Os11g08100 | Eukaryotic aspartyl protease family protein, expressed |
| Os.46160.1.S1_at | 0.467 | 0.161 | 0.290 | 1.613 | LOC_Os10g30790 | phosphate:H+ symporter family protein, expressed |
| Os.53055.1.S1_at | 0.468 | 0.712 | 0.798 | 0.963 | LOC_Os11g31450 | expressed protein |
| Os.53236.1.S1_at | 0.468 | 1.354 | 0.593 | 1.832 | LOC_Os02g43170 | B-box zinc finger family protein, expressed |
| Os.6363.1.S1_at | 0.468 | 0.867 | 3.866 | 1.141 | LOC_Os10g38080 | Subtilisin N-terminal Region family protein, expressed |
| Os.27382.2.S1_at | 0.469 | 0.116 | 0.266 | 1.171 | LOC_Os02g02120 | wall-associated kinase 3, putative, expressed |
| Os.49819.1.S1_at | 0.469 | 1.462 | 2.590 | 3.825 | LOC_Os11g36200 | receptor kinase, putative, expressed |
| Os.19277.1.S1_at | 0.471 | 0.291 | 0.964 | 0.845 | LOC_Os07g04150 | expressed protein |
| Os.27793.1.S1_at | 0.471 | 0.583 | 0.764 | 0.556 | LOC_Os02g14430 | Peroxidase 52 precursor, putative, expressed |
| Os.12392.1.S1_at | 0.471 | 0.576 | 0.772 | 0.975 | LOC_Os03g59210 | hypothetical protein |
| Os.51853.1.S1_at | 0.472 | 0.834 | 0.626 | 0.863 | LOC_Os06g28480 | Leucine Rich Repeat family protein, expressed |
| Os.49400.1.S2_s_at | 0.472 | 1.133 | 0.830 | 0.786 | LOC_Os11g11960 | NBS-LRR type disease resistance protein, putative, expressed |
| Os.52509.1.S1_at | 0.472 | 1.306 | 0.981 | 0.794 | LOC_Os04g33950 | Transcription factor E2F/dimerisation partner family protein, expressed |
| Os.53337.1.S1_at | 0.472 | 1.609 | 0.203 | 1.563 | LOC_Os02g17090 | Subtilisin N-terminal Region family protein, expressed |
| Os.49973.1.S1_at | 0.473 | 0.879 | 0.436 | 1.258 | LOC_Os03g20210 | Eukaryotic aspartyl protease family protein, expressed |
| OsAffx.18807.1.S1_at | 0.473 | 0.947 | 0.579 | 1.026 | LOC_Os11g11770 | NB-ARC domain containing protein, expressed |
| Os.6023.1.S1_s_at | 0.473 | 6.043 | 2.851 | 1.358 | LOC_Os05g46460 | hydrolase, alpha/beta fold family protein, putative, expressed |
| Os.18178.1.S1_at | 0.474 | 3.665 | 1.087 | 0.947 | LOC_Os03g03550 | bZIP family transcription factor, putative, expressed |
| Os.18395.1.S1_s_at | 0.474 | 1.088 | 0.581 | 0.567 | LOC_Os06g15620 | GAST1 protein precursor, putative, expressed |
| Os.12029.1.S1_a_at | 0.475 | 1.085 | 1.789 | 0.965 | LOC_Os06g06290 | GDSL-like Lipase/Acylhydrolase family protein, expressed |
| Os.55488.1.S1_at | 0.476 | 0.537 | 0.568 | 0.992 | LOC_Os08g28790 | Dirigent-like protein, expressed |

| ProbeSetID | *OMTN4* -OE[a] | Stress response[b] | | | TIGR Locus ID[c] | Annotation |
|---|---|---|---|---|---|---|
| | | Drought | Salt | Cold | | |
| Os.37822.1.A1_a_at | 0.476 | 0.967 | 0.539 | 2.346 | LOC_Os01g07370 | CENP-E like kinetochore protein, putative, expressed |
| Os.14079.1.S1_at | 0.476 | 3.021 | 0.827 | 3.299 | LOC_Os03g58300 | Indole-3-glycerol phosphate lyase, chloroplast precursor, putative, expressed |
| Os.52189.1.S1_at | 0.476 | 0.265 | 0.489 | 0.906 | LOC_Os06g19260 | expressed protein |
| Os.6035.1.S1_at | 0.477 | 0.391 | 0.690 | 0.662 | LOC_Os07g46350 | Serine carboxypeptidase II-3 precursor, putative, expressed |
| Os.33603.1.S1_at | 0.477 | 1.075 | 1.008 | 1.254 | LOC_Os03g03500 | heavy metal-associated domain containing protein, expressed |
| Os.49501.1.A1_at | 0.477 | 2.798 | 2.460 | 2.123 | LOC_Os06g12230 | TCP family transcription factor containing protein, expressed |
| Os.54530.1.S1_at | 0.478 | 0.945 | 0.691 | 0.602 | LOC_Os06g49100 | leucine-rich repeat family protein, putative, expressed |
| Os.35433.1.S1_at | 0.478 | 0.402 | 0.165 | 14.371 | LOC_Os04g39320 | expressed protein |
| Os.52465.1.S1_at | 0.479 | 0.428 | 0.559 | 0.518 | LOC_Os07g36210 | expressed protein |
| Os.56891.1.S1_at | 0.479 | 1.031 | 0.811 | 1.338 | LOC_Os03g29930 | expressed protein |
| OsAffx.24726.1.S1_s_at | 0.479 | 0.405 | 0.882 | 3.131 | LOC_Os02g44710 | expressed protein |
| Os.24699.1.S1_at | 0.480 | 0.960 | 0.824 | 0.543 | LOC_Os02g47190 | Calcium-dependent protein kinase substrate protein, putative, expressed |
| OsAffx.12740.1.S1_s_at | 0.480 | 1.102 | 0.834 | 1.145 | LOC_Os03g05520 | expressed protein |
| Os.54232.1.S1_at | 0.481 | 4.336 | 5.508 | 0.937 | LOC_Os05g37190 | Zinc finger, C2H2 type family protein, expressed |
| Os.9211.1.S1_at | 0.481 | 0.756 | 0.668 | 1.086 | LOC_Os07g45060 | SCP-like extracellular protein, expressed |
| Os.4683.2.S1_at | 0.482 | 0.925 | 0.695 | 0.888 | LOC_Os01g42520 | expressed protein |
| Os.14411.1.S1_at | 0.482 | 0.582 | 0.462 | 1.605 | LOC_Os06g16640 | Carboxyl-terminal peptidase, putative, expressed |
| OsAffx.31710.1.S1_x_at | 0.482 | 5.188 | 2.823 | 3.467 | LOC_Os12g12260 | Diacylglycerol kinase 1, putative, expressed |
| Os.14195.1.S1_at | 0.483 | 0.669 | 1.073 | 1.587 | LOC_Os03g19200 | TPR Domain containing protein, expressed |
| Os.27507.1.S1_at | 0.483 | 0.899 | 3.222 | 1.949 | LOC_Os06g35700 | Reticuline oxidase precursor, putative, expressed |
| Os.34174.1.S1_at | 0.483 | 1.000 | 0.195 | 1.921 | LOC_Os04g38790 | expressed protein |
| OsAffx.19579.1.S1_at | 0.483 | 5.257 | 4.009 | 1.092 | LOC_Os12g09640 | Protein phosphatase 2C containing protein, expressed |
| Os.32454.1.S1_at | 0.484 | 0.545 | 0.782 | 1.234 | LOC_Os01g01650 | Isoflavone reductase homolog IRL, putative, expressed |
| Os.14830.1.S1_a_at | 0.484 | 1.661 | 2.891 | 1.334 | LOC_Os01g63190 | Multicopper oxidase family protein, expressed |

| ProbeSetID | *OMTN4* -OE[a] | Stress response[b] | | | TIGR Locus ID[c] | Annotation |
|---|---|---|---|---|---|---|
| | | Drought | Salt | Cold | | |
| Os.21066.1.S1_at | 0.484 | 1.137 | 1.242 | 1.610 | LOC_Os03g56250 | wound and phytochrome signaling involved receptor like kinase, putative, expressed |
| Os.5727.1.S1_at | 0.485 | 0.964 | 0.561 | 1.156 | LOC_Os06g05550 | GDSL-like Lipase/Acylhydrolase family protein, expressed |
| Os.16142.1.S1_at | 0.486 | 3.188 | 1.268 | 1.483 | LOC_Os03g31510 | Cystatin, putative, expressed |
| Os.17881.1.S1_at | 0.486 | 0.552 | 0.484 | 1.156 | LOC_Os04g54940 | microtubule-associated EB1 family protein, putative, expressed |
| OsAffx.6930.1.S1_at | 0.486 | 0.601 | 0.731 | 0.939 | LOC_Os10g40810 | GATA zinc finger family protein, expressed |
| Os.55266.1.S1_at | 0.486 | 1.135 | 1.332 | 1.198 | LOC_Os07g10620 | expressed protein |
| OsAffx.14381.1.S1_x_at | 0.486 | 0.489 | 0.864 | 0.736 | LOC_Os04g52700 | expressed protein |
| OsAffx.7414.1.S1_at | 0.486 | 0.129 | 0.259 | 0.345 | LOC_Os11g44700 | expressed protein |
| Os.56210.1.S1_at | 0.487 | 3.781 | 17.738 | 2.172 | LOC_Os04g42950 | Myb protein, putative, expressed |
| Os.55085.1.S1_at | 0.487 | 0.322 | 1.373 | 2.271 | LOC_Os09g01580 | hypothetical protein |
| OsAffx.5776.1.S1_at | 0.488 | 0.643 | 0.315 | 0.153 | LOC_Os08g10290 | SHR5-receptor-like kinase, putative, expressed |
| Os.10179.1.S1_at | 0.489 | 0.701 | 0.464 | 0.737 | LOC_Os06g03640 | BAG domain-containing protein, putative, expressed |
| Os.34196.1.S1_at | 0.489 | 0.989 | 0.697 | 2.237 | LOC_Os01g60110 | expressed protein |
| Os.11668.1.S1_at | 0.489 | 0.762 | 0.934 | 1.157 | LOC_Os03g19390 | Seed maturation protein PM36, putative, expressed |
| Os.27767.1.A1_s_at | 0.489 | 0.107 | 0.663 | 0.826 | LOC_Os04g29680 | Calcium binding EGF domain containing protein, expressed |
| Os.24746.1.S1_at | 0.489 | 1.460 | 0.994 | 1.519 | LOC_Os06g15690 | no apical meristem, putative, expressed |
| Os.52914.1.S1_at | 0.490 | 1.187 | 0.732 | 0.994 | LOC_Os02g14890 | expressed protein |
| Os.52282.1.S1_at | 0.490 | 0.110 | 0.155 | 0.557 | LOC_Os04g28780 | D-mannose binding lectin family protein, expressed |
| Os.5768.1.S1_at | 0.490 | 2.041 | 2.326 | 1.237 | LOC_Os09g15320 | Ubiquitin-conjugating enzyme E2 M, putative, expressed |
| Os.53258.1.S1_at | 0.490 | 6.270 | 2.597 | 1.151 | LOC_Os03g60220 | expressed protein |
| OsAffx.28957.1.S1_at | 0.491 | 0.687 | 0.603 | 0.542 | LOC_Os07g48700 | expressed protein |
| Os.11657.1.S1_at | 0.491 | 4.788 | 3.002 | 2.751 | LOC_Os01g47760 | glutaredoxin family protein, putative, expressed |
| Os.23209.1.S1_at | 0.491 | 0.383 | 0.368 | 0.787 | LOC_Os05g39350 | expressed protein |

| ProbeSetID | *OMTN4* -OE[a] | Stress response[b] | | | TIGR Locus ID[c] | Annotation |
|---|---|---|---|---|---|---|
| | | Drought | Salt | Cold | | |
| Os.10477.2.S1_x_at | 0.492 | 1.814 | 1.780 | 1.407 | LOC_Os05g11810 | oxidoreductase, 2OG-Fe oxygenase family protein, expressed |
| Os.14382.1.S1_at | 0.492 | 0.638 | 0.628 | 0.823 | LOC_Os02g31860 | expressed protein |
| Os.6210.1.S1_at | 0.493 | 0.255 | 0.440 | 0.593 | LOC_Os03g56270 | Receptor protein kinase CLAVATA1 precursor, putative, expressed |
| OsAffx.24328.1.S1_at | 0.493 | 0.377 | 0.439 | 0.580 | LOC_Os02g19530 | lectin receptor kinase 7, putative |
| Os.20313.1.S1_s_at | 0.493 | 2.126 | 1.109 | 1.435 | LOC_Os01g72290 | Germin-like protein subfamily 2 member 4 precursor, putative, expressed |
| Os.18299.1.S1_at | 0.493 | 5.026 | 2.817 | 1.002 | LOC_Os07g07410 | oxidoreductase, 2OG-Fe oxygenase family protein, expressed |
| Os.12183.1.S1_at | 0.494 | 1.071 | 1.223 | 1.763 | LOC_Os04g33030 | expressed protein |
| Os.10498.1.S1_at | 0.494 | 0.763 | 1.188 | 1.500 | LOC_Os10g35100 | expressed protein |
| Os.7539.1.S1_at | 0.494 | 1.086 | 0.825 | 1.218 | LOC_Os03g03320 | expressed protein |
| OsAffx.19285.1.S1_at | 0.494 | 0.808 | 2.512 | 1.146 | LOC_Os11g40810 | Leucine Rich Repeat family protein, expressed |
| OsAffx.10980.1.S1_at | 0.495 | 1.235 | 1.330 | 3.191 | LOC_Os01g09150 | hypothetical protein |
| Os.34139.1.S1_at | 0.495 | 10.314 | 4.954 | 1.162 | LOC_Os08g04630 | EF hand family protein, expressed |
| OsAffx.31405.1.S1_at | 0.495 | 1.727 | 6.364 | 2.364 | LOC_Os11g40410 | expressed protein |
| Os.33210.1.S1_at | 0.496 | 1.869 | 1.445 | 0.759 | LOC_Os01g68570 | expressed protein |
| Os.56999.1.S1_at | 0.496 | 2.037 | 1.361 | 1.473 | LOC_Os03g17940 | PAP2 superfamily protein, expressed |
| Os.3655.1.S1_at | 0.496 | 0.357 | 0.247 | 0.523 | LOC_Os01g18730 | hypothetical protein |
| OsAffx.13523.1.S1_at | 0.496 | 1.984 | 0.889 | 1.238 | LOC_Os03g57400 | hypothetical protein |
| OsAffx.7288.1.S1_at | 0.496 | 0.410 | 0.896 | 0.794 | LOC_Os11g33970 | expressed protein |
| Os.27386.1.S1_at | 0.496 | 0.375 | 0.505 | 1.154 | LOC_Os07g41250 | POT family protein, expressed |
| Os.31344.1.S1_at | 0.497 | 1.964 | 1.596 | 0.534 | LOC_Os01g63970 | sialyltransferase, putative, expressed |
| Os.20201.1.S1_at | 0.497 | 0.983 | 0.701 | 1.015 | LOC_Os02g54650 | diacylglycerol kinase, putative, expressed |
| OsAffx.9584.1.S1_at | 0.498 | 2.371 | 0.902 | 4.423 | LOC_Os01g60600 | WRKY DNA binding domain containing protein, expressed |
| Os.38013.1.S1_a_at | 0.498 | 0.667 | 1.078 | 0.843 | LOC_Os03g08960 | Homeobox domain containing protein, expressed |
| OsAffx.24063.1.S1_at | 0.498 | 0.917 | 4.792 | 0.958 | LOC_Os02g02230 | Cytochrome P450 51, putative, expressed |

| ProbeSetID | *OMTN4* -OE[a] | Stress response[b] | | | TIGR Locus ID[c] | Annotation |
|---|---|---|---|---|---|---|
| | | Drought | Salt | Cold | | |
| Os.25104.2.S1_at | 0.499 | 0.978 | 0.702 | 1.151 | LOC_Os02g52280 | expressed protein |
| Os.7281.1.S1_at | 0.499 | 0.433 | 0.831 | 1.292 | LOC_Os01g68730 | expressed protein |
| OsAffx.17900.1.S1_at | 0.499 | 1.243 | 0.655 | 0.508 | LOC_Os09g25940 | Receptor family ligand binding region containing protein |
| Os.609.3.S1_a_at | 0.499 | 2.656 | 1.656 | 1.838 | LOC_Os08g37670 | Plastocyanin-like domain containing protein, expressed |
| Os.46840.1.S1_x_at | 0.500 | 0.338 | 0.425 | 0.884 | LOC_Os10g34700 | expressed protein |

NOTE: All values in the table are expression level change folds (transgenic/WT, or stress/normal)(mean of the repeats).The folds higher than 2 were indicated by red colour, and the folds lower than 0.5 were indicated by green colour.

[a]Expression level change folds in the *OMTN4*-OE plants.

[b]Expression profile of the genes under drought, salt and cold stress was download from the GEO database (http://www.ncbi.nlm.nih.gov/geo/, accession number: GSE6901).

[c]The locus ID was download from Rice Annotation Project (http://rice.plantbiology.msu.edu/).

**Supplementary Table S7. Up- and down-regulated genes in the transgenic rice plants overexpressing *OMTN6*.**

| ProbeSetID | *OMTN6* -OE[a] | Stress response[b] | | | TIGR Locus ID[c] | Annotation |
|---|---|---|---|---|---|---|
| | | Drought | Salt | Cold | | |
| Os.4184.1.S1_at | 30.978 | 0.343 | 1.364 | 0.904 | LOC_Os02g15350 | Dof domain, zinc finger family protein, expressed |
| Os.56053.1.S1_at | 17.356 | 1.333 | 0.852 | 21.111 | LOC_Os04g43090 | expressed protein |
| Os.11065.1.A1_at | 12.391 | 1.191 | 0.512 | 1.153 | LOC_Os03g19600 | retrotransposon protein, putative, Ty3-gypsy subclass, expressed |
| Os.7991.1.S1_at | 8.962 | 3.486 | 1.811 | 1.730 | LOC_Os04g41620 | Endochitinase A precursor, putative, expressed |
| Os.34982.1.A1_at | 8.067 | 0.348 | 0.828 | 0.751 | LOC_Os04g17660 | Rhodanese-like domain containing protein, expressed |
| Os.30575.1.A1_at | 6.926 | 4.360 | 0.880 | 3.560 | LOC_Os02g12470 | hypothetical protein |
| OsAffx.28294.2.S1_at | 6.728 | 1.828 | 1.609 | 1.500 | LOC_Os07g05840 | expressed protein |
| Os.56966.1.S1_at | 6.444 | 0.149 | 0.238 | 1.015 | LOC_Os05g50390 | expressed protein |
| Os.48948.1.S1_at | 5.941 | 3.571 | 3.786 | 5.429 | LOC_Os10g26910 | transposon protein, putative, CACTA, En/Spm sub-class |
| Os.22967.1.S1_s_at | 5.532 | 1.012 | 1.124 | 1.000 | LOC_Os03g02070 | heavy-metal-associated domain-containing protein, putative, expressed |
| Os.50950.1.S1_x_at | 5.367 | 0.471 | 0.679 | 0.797 | LOC_Os02g44730 | tetracycline transporter protein, putative, expressed |
| Os.10266.1.S1_at | 5.037 | 0.167 | 0.362 | 0.630 | LOC_Os03g43100 | expressed protein |
| Os.10556.1.S1_at | 4.682 | 8.593 | 9.683 | 0.495 | LOC_Os02g15860 | expressed protein |
| Os.49703.1.S1_at | 4.252 | 1.493 | 0.958 | 2.099 | LOC_Os08g25260 | transposon protein, putative, unclassified |
| Os.16804.1.S1_at | 4.225 | 1.828 | 2.927 | 0.796 | LOC_Os10g23060 | expressed protein |
| Os.21300.2.S1_at | 4.171 | 0.841 | 0.609 | 0.963 | LOC_Os01g42690 | Phospholipase/Carboxylesterase family protein, expressed |
| Os.39129.1.S1_at | 3.784 | 0.206 | 1.333 | 0.310 | LOC_Os10g33760 | NAC-domain containing protein 21/22, putative, expressed |
| Os.18981.1.S1_x_at | 3.695 | 1.047 | 1.180 | 0.867 | LOC_Os07g18120 | Aldehyde oxidase 3, putative, expressed |
| Os.4766.1.S1_at | 3.601 | 1.589 | 1.274 | 1.156 | LOC_Os10g28350 | 1,2-dihydroxy-3-keto-5-methylthiopentene dioxygenase, putative, expressed |
| Os.49931.1.S1_at | 3.556 | 1.301 | 0.939 | 1.679 | LOC_Os11g43250 | Leucine Rich Repeat family protein, expressed |
| Os.13466.1.S1_at | 3.536 | 1.288 | 1.411 | 1.043 | LOC_Os02g43300 | expressed protein |
| Os.16164.1.S1_at | 3.471 | 5.690 | 4.444 | 1.412 | LOC_Os06g10530 | expressed protein |
| Os.37717.1.A2_s_at | 3.456 | 3.188 | 2.187 | 1.410 | LOC_Os05g15770 | Xylanase inhibitor protein 2 precursor, putative, expressed |

| ProbeSetID | *OMTN6* -OE[a] | Stress response[b] | | | TIGR Locus ID[c] | Annotation |
|---|---|---|---|---|---|---|
| | | Drought | Salt | Cold | | |
| OsAffx.17389.1.S1_s_at | 3.454 | 0.665 | 0.637 | 0.955 | LOC_Os08g37930 | Rare lipoprotein A like double-psi beta-barrel containing protein, expressed |
| Os.51595.1.S1_at | 3.314 | 1.916 | 2.158 | 1.395 | LOC_Os01g46370 | lipase class 3 family protein, putative, expressed |
| Os.32686.1.S1_at | 3.291 | 1.128 | 0.739 | 5.958 | LOC_Os01g65110 | POT family protein, expressed |
| OsAffx.19309.1.S1_at | 3.204 | 0.405 | 0.750 | 0.712 | LOC_Os11g41500 | NC domain-containing protein, putative |
| Os.40325.1.A1_at | 3.174 | 1.436 | 1.127 | 1.855 | LOC_Os10g09290 | expressed protein |
| Os.11040.1.S1_x_at | 3.166 | 2.689 | 2.236 | 0.960 | LOC_Os10g31320 | transposon protein, putative, CACTA, En/Spm sub-class, expressed |
| Os.1385.1.S1_at | 3.142 | 1.305 | 1.446 | 1.301 | LOC_Os01g71670 | Glucan endo-1,3-beta-glucosidase GII precursor, putative, expressed |
| OsAffx.20724.2.S1_s_at | 3.119 | 1.834 | 2.700 | 0.748 | LOC_Os10g07010 | senescence-associated protein 15, putative |
| Os.5816.1.S1_at | 3.117 | 0.683 | 0.276 | 12.233 | LOC_Os09g35010 | AP2 domain containing protein, expressed |
| OsAffx.32170.1.S1_at | 3.059 | 2.386 | 1.087 | 1.204 | LOC_Os12g43450 | P21 protein, putative, expressed |
| Os.7189.1.S1_at | 3.026 | 1.177 | 1.452 | 1.294 | LOC_Os11g04010 | latex-abundant protein, putative, expressed |
| Os.27874.2.S1_x_at | 3.024 | 1.365 | 1.571 | 0.936 | LOC_Os05g48840 | expressed protein |
| OsAffx.26673.1.S1_at | 3.005 | 0.763 | 0.852 | 0.754 | LOC_Os05g01010 | hypothetical protein |
| Os.8823.1.S1_at | 2.900 | 0.948 | 0.474 | 0.351 | LOC_Os06g15430 | expressed protein |
| Os.55213.1.S1_at | 2.850 | 0.757 | 0.982 | 0.780 | LOC_Os09g24350 | expressed protein |
| Os.46672.1.S1_at | 2.826 | 0.295 | 0.629 | 0.473 | LOC_Os10g31290 | expressed protein |
| Os.14047.1.S1_at | 2.752 | 1.821 | 1.449 | 0.529 | LOC_Os01g70110 | No apical meristem protein, expressed |
| Os.28098.1.S1_at | 2.706 | 39.630 | 15.673 | 0.834 | LOC_Os10g32810 | Glycosyl hydrolase family 14 protein, expressed |
| Os.27497.1.S1_at | 2.676 | 0.191 | 0.121 | 0.681 | LOC_Os12g12390 | transposon protein, putative, CACTA, En/Spm sub-class, expressed |
| Os.18257.1.S1_at | 2.662 | 6.371 | 5.734 | 0.915 | LOC_Os07g10840 | seed imbibition protein, putative, expressed |
| OsAffx.25720.1.S1_at | 2.651 | 0.814 | 1.029 | 0.886 | LOC_Os03g55460 | expressed protein |
| Os.12077.1.S1_at | 2.642 | 1.487 | 1.497 | 1.038 | LOC_Os08g44270 | Vignain precursor, putative, expressed |
| Os.8098.1.S1_at | 2.628 | 0.325 | 1.475 | 0.605 | LOC_Os11g18570 | Cytochrome P450 family protein, expressed |
| Os.50470.1.S1_at | 2.627 | 3.270 | 4.170 | 0.793 | LOC_Os01g71310 | Cytokinin dehydrogenase 1 precursor, putative, expressed |

| ProbeSetID | *OMTN6* -OE[a] | Stress response[b] | | | TIGR Locus ID[c] | Annotation |
|---|---|---|---|---|---|---|
| | | Drought | Salt | Cold | | |
| OsAffx.23454.1.S1_at | 2.618 | 2.292 | 2.646 | 2.954 | LOC_Os01g29870 | Multidrug resistance protein 1, putative, expressed |
| Os.44872.1.S1_at | 2.616 | 0.614 | 0.879 | 0.494 | LOC_Os06g01660 | Integral membrane protein DUF6 containing protein, expressed |
| Os.47937.2.S1_x_at | 2.597 | 1.398 | 2.140 | 1.104 | LOC_Os12g17160 | Sulfotransferase domain containing protein, expressed |
| Os.17047.1.A1_at | 2.591 | 0.564 | 1.205 | 0.910 | LOC_Os07g18230 | Protein kinase domain containing protein, expressed |
| Os.11707.1.A1_at | 2.544 | 0.143 | 1.445 | 0.298 | LOC_Os03g54130 | Papain family cysteine protease containing protein, expressed |
| OsAffx.13358.1.S1_at | 2.542 | 6.082 | 2.242 | 3.731 | LOC_Os03g45180 | expressed protein |
| Os.11831.1.S1_at | 2.530 | 2.886 | 3.593 | 2.243 | LOC_Os06g13560 | SAM dependent carboxyl methyltransferase family protein, expressed |
| Os.48076.1.S1_at | 2.517 | 1.432 | 1.176 | 0.762 | LOC_Os12g36750 | expressed protein |
| Os.13708.2.A1_at | 2.515 | 13.409 | 7.453 | 2.905 | LOC_Os03g22790 | Glycosyl hydrolase family 14 protein, expressed |
| Os.54937.1.S1_at | 2.501 | 0.672 | 0.625 | 1.484 | LOC_Os08g41780 | ab-hydrolase associated lipase region family protein, expressed |
| Os.10166.1.S1_at | 2.493 | 2.053 | 1.089 | 1.502 | LOC_Os04g41680 | Endochitinase A precursor, putative, expressed |
| Os.2821.1.A1_at | 2.477 | 1.653 | 0.305 | 1.017 | LOC_Os02g02210 | aminotransferase, class III family protein, expressed |
| Os.23156.1.S1_at | 2.461 | 1.022 | 0.881 | 1.003 | LOC_Os02g02320 | Serine carboxypeptidase III precursor, putative, expressed |
| OsAffx.24063.1.S1_at | 2.420 | 0.917 | 4.792 | 0.958 | LOC_Os02g02230 | Cytochrome P450 51, putative, expressed |
| Os.17158.1.S1_at | 2.420 | 1.042 | 1.382 | 1.592 | LOC_Os01g70490 | Potassium transporter 5, putative, expressed |
| Os.20169.1.S1_at | 2.418 | 0.421 | 0.527 | 0.578 | LOC_Os06g34730 | expressed protein |
| Os.2694.1.S1_at | 2.416 | 38.091 | 13.823 | 1.497 | LOC_Os04g43200 | caleosin 2, putative, expressed |
| Os.5390.1.S1_at | 2.379 | 1.123 | 1.416 | 2.828 | LOC_Os12g33130 | expressed protein |
| Os.7530.1.S1_at | 2.375 | 0.332 | 0.472 | 1.515 | LOC_Os09g29710 | Expansin-related protein 2 precursor, putative, expressed |
| Os.9851.1.S1_at | 2.363 | 1.029 | 0.833 | 0.871 | LOC_Os01g48130 | no apical meristem, putative, expressed |
| Os.53971.1.S1_at | 2.351 | 0.569 | 0.629 | 0.527 | LOC_Os07g37110 | equilibrative nucleoside transporter, putative, expressed |
| OsAffx.24672.1.S1_at | 2.331 | 0.276 | 0.090 | 0.541 | LOC_Os02g41770 | hypothetical protein |
| OsAffx.27289.1.S1_at | 2.323 | 0.670 | 1.144 | 0.891 | LOC_Os05g43110 | GDSL-like Lipase/Acylhydrolase family protein, expressed |
| Os.8618.1.S1_at | 2.315 | 3.417 | 1.755 | 0.926 | LOC_Os05g44810 | AUX/IAA family protein, expressed |

| ProbeSetID | *OMTN6* -OE[a] | Stress response[b] | | | TIGR Locus ID[c] | Annotation |
|---|---|---|---|---|---|---|
| | | Drought | Salt | Cold | | |
| OsAffx.21073.1.S1_at | 2.307 | 0.559 | 0.401 | 0.494 | LOC_Os07g02620 | NB-ARC domain containing protein |
| Os.6304.1.S1_at | 2.296 | 7.841 | 3.822 | 8.307 | LOC_Os08g31090 | expressed protein |
| Os.1215.1.S1_at | 2.288 | 1.309 | 1.132 | 1.010 | LOC_Os04g37500 | Glutamate decarboxylase, putative, expressed |
| Os.40473.1.S1_at | 2.286 | 0.484 | 0.444 | 1.173 | LOC_Os07g02660 | NB-ARC domain containing protein |
| Os.6864.1.S1_at | 2.259 | 1.642 | 1.864 | 1.129 | LOC_Os01g61070 | heavy metal-associated domain containing protein, expressed |
| Os.55459.1.S1_at | 2.255 | 0.887 | 0.932 | 0.865 | LOC_Os03g57990 | Protease inhibitor/seed storage/LTP family protein, expressed |
| Os.9686.1.S1_at | 2.246 | 0.706 | 0.803 | 0.782 | LOC_Os06g43620 | Haemolysin-III related family protein, expressed |
| Os.2954.1.S1_at | 2.241 | 1.028 | 1.163 | 1.135 | LOC_Os03g25440 | expressed protein |
| Os.30473.1.S1_at | 2.240 | 7.227 | 2.439 | 2.712 | LOC_Os01g11150 | oxidoreductase, 2OG-Fe oxygenase family protein, expressed |
| Os.17753.1.A1_s_at | 2.231 | 1.666 | 1.524 | 0.892 | LOC_Os04g21110 | Phosphoribulokinase/Uridine kinase family protein, expressed |
| Os.31381.1.S1_at | 2.227 | 0.618 | 1.475 | 0.531 | LOC_Os01g03720 | Myb-like DNA-binding domain containing protein, expressed |
| Os.54760.1.S1_at | 2.227 | 0.624 | 1.610 | 1.150 | LOC_Os05g49840 | Lipase family protein, expressed |
| Os.15799.1.S1_at | 2.210 | 3.710 | 3.352 | 1.216 | LOC_Os03g11420 | Glycosyl hydrolase family 1 protein, expressed |
| Os.6776.1.S1_at | 2.209 | 0.279 | 0.435 | 0.632 | LOC_Os07g35480 | Glycosyl hydrolases family 17 protein, expressed |
| OsAffx.3920.1.S1_at | 2.208 | 0.878 | 1.683 | 3.098 | LOC_Os04g27670 | Terpene synthase family, metal binding domain containing protein, expressed |
| Os.53085.1.S1_at | 2.202 | 5.860 | 1.580 | 0.631 | LOC_Os05g49900 | Fatty acid elongase, putative, expressed |
| Os.10799.1.S1_at | 2.189 | 1.309 | 1.424 | 1.098 | LOC_Os04g24600 | Cysteine proteinase 1 precursor, putative, expressed |
| Os.6645.1.S1_at | 2.181 | 0.426 | 0.493 | 1.103 | LOC_Os07g25050 | thionin-like peptide, putative, expressed |
| Os.53458.1.S1_at | 2.179 | 1.766 | 1.247 | 1.299 | LOC_Os09g17560 | O-methyltransferase family protein, expressed |
| Os.55687.1.S1_at | 2.175 | 0.361 | 0.908 | 1.273 | LOC_Os06g44220 | Uncharacterized protein family protein |
| Os.18318.1.S1_at | 2.167 | 2.201 | 1.759 | 0.883 | LOC_Os03g10240 | expressed protein |
| Os.54430.1.S1_at | 2.164 | 0.568 | 0.733 | 0.677 | LOC_Os02g37200 | expressed protein |
| Os.54535.1.S1_at | 2.161 | 4.892 | 3.244 | 1.433 | LOC_Os08g36410 | ANTH domain containing protein, expressed |
| OsAffx.14605.1.S1_at | 2.148 | 1.501 | 1.918 | 3.028 | LOC_Os05g09020 | WRKY transcription factor 50, putative, expressed |

| ProbeSetID | *OMTN6* -OE[a] | Stress response[b] | | | TIGR Locus ID[c] | Annotation |
|---|---|---|---|---|---|---|
| | | Drought | Salt | Cold | | |
| Os.51848.1.S1_x_at | 2.143 | 0.920 | 1.389 | 0.694 | LOC_Os10g31330 | transposon protein, putative, CACTA, En/Spm sub-class, expressed |
| Os.48287.1.S1_at | 2.132 | 0.331 | 0.701 | 0.595 | LOC_Os04g38570 | multidrug resistance P-glycoprotein, putative, expressed |
| Os.51369.1.S1_at | 2.132 | 1.129 | 1.364 | 0.787 | LOC_Os02g51740 | VQ motif family protein, expressed |
| Os.44806.1.A1_x_at | 2.126 | 1.157 | 1.739 | 0.944 | LOC_Os06g03800 | ankyrin repeat family protein, putative, expressed |
| Os.31816.2.S1_x_at | 2.125 | 0.881 | 0.854 | 1.166 | LOC_Os01g70220 | YDG/SRA domain containing protein, expressed |
| Os.54821.1.S1_at | 2.111 | 1.309 | 0.829 | 1.098 | LOC_Os08g10670 | Eukaryotic aspartyl protease family protein, expressed |
| Os.24065.2.S1_at | 2.103 | 0.325 | 0.849 | 0.754 | LOC_Os03g11690 | expressed protein |
| OsAffx.27339.1.S1_s_at | 2.101 | 1.354 | 1.239 | 1.140 | LOC_Os05g46440 | expressed protein |
| Os.53672.1.A1_at | 2.092 | 0.772 | 1.125 | 1.099 | LOC_Os07g27350 | expressed protein |
| Os.1740.1.S1_at | 2.078 | 3.690 | 3.067 | 0.839 | LOC_Os01g26120 | plant integral membrane protein TIGR01569 containing protein, expressed |
| Os.11682.1.S1_at | 2.074 | 1.783 | 2.299 | 0.393 | LOC_Os01g58640 | Ser/Thr protein phosphatase family protein, expressed |
| Os.5087.1.S1_at | 2.061 | 2.341 | 2.306 | 0.438 | LOC_Os11g03780 | Alpha-L-arabinofuranosidase C-terminus family protein, expressed |
| OsAffx.2939.1.S1_at | 2.046 | 1.757 | 0.795 | 0.371 | LOC_Os02g41940 | RNase H domain-containing protein, putative |
| Os.49023.1.S1_x_at | 2.041 | 1.067 | 1.645 | 0.914 | LOC_Os03g22680 | CHY zinc finger family protein, expressed |
| OsAffx.6545.1.S1_at | 2.040 | 0.646 | 0.799 | 1.005 | LOC_Os10g01500 | expressed protein |
| Os.9700.1.S1_at | 2.037 | 1.165 | 0.836 | 0.872 | LOC_Os08g28970 | expressed protein |
| Os.27875.1.S1_at | 2.007 | 0.724 | 2.518 | 1.782 | LOC_Os10g28120 | Chitinase 1 precursor, putative, expressed |
| Os.11666.1.S1_at | 2.003 | 21.785 | 10.581 | 2.143 | LOC_Os09g36200 | senescence-inducible chloroplast stay-green protein 2, putative, expressed |
| Os.29093.1.S1_at | 0.050 | 1.113 | 1.511 | 0.390 | LOC_Os07g14600 | Peptidase family M20/M25/M40 containing protein, expressed |
| OsAffx.17366.1.S1_at | 0.084 | 2.606 | 5.273 | 4.973 | LOC_Os08g36920 | AP2 domain containing protein, expressed |
| Os.14616.1.S1_at | 0.106 | 3.049 | 0.953 | 1.947 | LOC_Os07g34260 | Chalcone and stilbene synthases, N-terminal domain containing protein, expressed |
| Os.54944.1.S1_at | 0.112 | 28.688 | 19.029 | 10.857 | LOC_Os02g52670 | AP2 domain containing protein, expressed |
| Os.6043.1.S1_at | 0.113 | 2.123 | 3.429 | 2.395 | LOC_Os04g23550 | Helix-loop-helix DNA-binding domain containing protein, expressed |

| ProbeSetID | *OMTN6* -OE[a] | Stress response[b] | | | TIGR Locus ID[c] | Annotation |
|---|---|---|---|---|---|---|
| | | Drought | Salt | Cold | | |
| Os.12201.2.S1_at | 0.115 | 2.702 | 2.693 | 0.977 | LOC_Os09g27820 | 1-aminocyclopropane-1-carboxylate oxidase 1, putative, expressed |
| Os.18463.1.S1_at | 0.117 | 9.807 | 2.097 | 5.040 | LOC_Os05g37660 | AER, putative, expressed |
| Os.54961.1.S1_at | 0.121 | 5.200 | 1.663 | 2.109 | LOC_Os05g46350 | IQ calmodulin-binding motif family protein, expressed |
| OsAffx.15812.1.S1_at | 0.136 | 1.455 | 1.000 | 1.182 | LOC_Os06g38120 | expressed protein |
| Os.11812.1.S1_at | 0.152 | 8.155 | 0.733 | 1.271 | LOC_Os02g48770 | SAM dependent carboxyl methyltransferase family protein, expressed |
| OsAffx.11788.1.S1_at | 0.155 | 0.376 | 0.313 | 0.649 | LOC_Os01g68460 | DC1 domain-containing protein, putative, expressed |
| Os.53407.1.S1_at | 0.155 | 3.573 | 1.288 | 0.763 | LOC_Os08g06210 | expressed protein |
| OsAffx.14201.1.S1_at | 0.164 | 0.337 | 0.136 | 0.472 | LOC_Os04g39360 | heavy metal-associated domain containing protein, expressed |
| Os.27688.1.A1_at | 0.169 | 0.957 | 0.731 | 9.416 | LOC_Os04g21350 | flowering promoting factor-like 1, putative, expressed |
| Os.49634.1.S1_x_at | 0.175 | 2.255 | 3.515 | 5.648 | LOC_Os01g55510 | Dynein light chain type 1 family protein, expressed |
| OsAffx.17348.1.S1_at | 0.176 | 0.699 | 0.928 | 2.325 | LOC_Os08g35590 | hypothetical protein |
| Os.56018.1.S1_at | 0.186 | 3.679 | 1.363 | 3.818 | LOC_Os05g44060 | expressed protein |
| Os.25606.1.S1_at | 0.197 | 1.476 | 0.793 | 1.850 | LOC_Os09g25060 | WRKY DNA binding domain containing protein, expressed |
| OsAffx.26389.1.S1_at | 0.198 | 0.619 | 0.619 | 0.952 | LOC_Os04g39300 | heavy metal-associated domain containing protein, expressed |
| Os.27019.1.S1_at | 0.212 | 3.466 | 2.619 | 0.967 | LOC_Os04g43410 | Glycosyl hydrolase family 1 protein, expressed |
| OsAffx.12733.1.S1_at | 0.215 | 1.472 | 1.239 | 3.033 | LOC_Os03g04740 | expressed protein |
| OsAffx.22469.1.S1_x_at | 0.216 | 3.843 | 2.093 | 2.284 | LOC_Os07g07040 | expressed protein |
| Os.15633.1.S1_at | 0.216 | 4.191 | 1.939 | 1.984 | LOC_Os10g35460 | COBRA-like protein 2 precursor, putative, expressed |
| OsAffx.14888.1.S1_at | 0.221 | 1.859 | 0.313 | 1.868 | LOC_Os05g30500 | expressed protein |
| Os.51757.1.S1_at | 0.226 | 1.405 | 1.042 | 0.813 | LOC_Os06g26270 | expressed protein |
| Os.11639.1.S1_at | 0.231 | 2.396 | 1.256 | 1.777 | LOC_Os06g05130 | Myristoyl-acyl carrier protein thioesterase, chloroplast precursor, putative, expressed |
| Os.14413.1.S1_at | 0.232 | 0.348 | 0.366 | 1.939 | LOC_Os07g48340 | Calcium-binding protein CAST, putative, expressed |
| Os.28030.1.S1_s_at | 0.236 | 0.920 | 1.569 | 2.386 | LOC_Os06g48160 | xyloglucan endotransglucosylase/hydrolase protein 15precursor, putative, |

| ProbeSetID | *OMTN6* -OE[a] | Stress response[b] | | | TIGR Locus ID[c] | Annotation |
|---|---|---|---|---|---|---|
| | | Drought | Salt | Cold | | |
| | | | | | | expressed |
| Os.27751.1.S1_at | 0.242 | 0.135 | 0.695 | 1.158 | LOC_Os02g36210 | Terpene synthase, N-terminal domain containing protein, expressed |
| OsAffx.24782.1.S1_s_at | 0.244 | 3.917 | 1.965 | 2.304 | LOC_Os02g48320 | DNA-binding protein, putative, expressed |
| OsAffx.5120.1.S1_x_at | 0.245 | 1.279 | 0.721 | 0.410 | LOC_Os06g45960 | Cytochrome P450 family protein, expressed |
| Os.51546.1.S1_at | 0.252 | 0.960 | 3.024 | 3.429 | LOC_Os03g08520 | expressed protein |
| Os.15701.1.S1_x_at | 0.256 | 0.893 | 1.201 | 1.459 | LOC_Os07g47350 | Potassium transporter 7, putative, expressed |
| OsAffx.15825.1.S1_at | 0.258 | 0.644 | 1.184 | 2.080 | LOC_Os06g38830 | Leucine Rich Repeat family protein, expressed |
| Os.50449.1.S1_at | 0.262 | 2.270 | 0.885 | 1.655 | LOC_Os03g10640 | Calcium-transporting ATPase 2, plasma membrane-type, putative, expressed |
| OsAffx.7506.1.S1_at | 0.262 | 1.073 | 0.938 | 1.741 | LOC_Os12g06780 | expressed protein |
| Os.17805.1.S1_at | 0.262 | 8.455 | 3.544 | 1.548 | LOC_Os01g63930 | Cytochrome P450 family protein, expressed |
| Os.54453.1.S1_at | 0.263 | 0.375 | 0.219 | 1.231 | LOC_Os08g44220 | expressed protein |
| Os.8508.1.S1_at | 0.263 | 0.601 | 0.326 | 0.201 | LOC_Os10g38880 | uncharacterized plant-specific domain TIGR01568 family protein, expressed |
| Os.30376.1.S1_at | 0.265 | 1.004 | 1.603 | 1.411 | LOC_Os01g02130 | expressed protein |
| Os.32455.1.S1_at | 0.265 | 1.276 | 0.980 | 1.156 | LOC_Os01g54670 | expressed protein |
| Os.47445.1.S1_at | 0.265 | 1.503 | 1.340 | 1.357 | LOC_Os02g26210 | flowering promoting factor-like 1, putative, expressed |
| Os.14938.1.S1_at | 0.265 | 2.039 | 0.863 | 1.882 | LOC_Os05g01140 | SAM dependent carboxyl methyltransferase family protein, expressed |
| Os.51227.1.S1_x_at | 0.267 | 1.487 | 0.690 | 1.205 | LOC_Os04g27790 | Terpene synthase family, metal binding domain containing protein, expressed |
| Os.6042.1.S1_at | 0.267 | 4.173 | 1.419 | 0.716 | LOC_Os07g25810 | transposon protein, putative, CACTA, En/Spm sub-class, expressed |
| OsAffx.2919.1.S1_at | 0.271 | 1.784 | 2.892 | 3.162 | LOC_Os02g39660 | Leucine Rich Repeat family protein, expressed |
| Os.16903.1.A1_at | 0.271 | 0.341 | 0.341 | 0.306 | LOC_Os05g06920 | RelA/SpoT containing protein, expressed |
| Os.14105.1.S1_at | 0.273 | 6.569 | 13.264 | 12.204 | LOC_Os11g05380 | Cytochrome P450 family protein, expressed |
| Os.52661.1.S1_at | 0.278 | 1.003 | 0.490 | 3.697 | LOC_Os11g44680 | expressed protein |
| Os.15706.1.S1_a_at | 0.279 | 1.408 | 0.692 | 1.753 | LOC_Os01g47580 | Lipid phosphate phosphatase 2, putative, expressed |
| OsAffx.3275.1.S1_at | 0.279 | 2.265 | 1.807 | 1.867 | LOC_Os03g19400 | expressed protein |

| ProbeSetID | *OMTN6* -OE[a] | Stress response[b] | | | TIGR Locus ID[c] | Annotation |
|---|---|---|---|---|---|---|
| | | Drought | Salt | Cold | | |
| Os.9199.1.S1_at | 0.279 | 4.709 | 1.450 | 1.368 | LOC_Os06g50330 | senescence/dehydration-associated protein, putative, expressed |
| Os.11685.1.S1_at | 0.280 | 0.189 | 0.057 | 0.465 | LOC_Os03g41200 | transposon protein, putative, CACTA, En/Spm sub-class, expressed |
| Os.22731.1.S1_at | 0.280 | 9.240 | 14.323 | 0.888 | LOC_Os03g16020 | 17.4 kDa class I heat shock protein, putative, expressed |
| Os.47945.1.A1_at | 0.281 | 0.810 | 0.490 | 0.792 | LOC_Os06g37510 | senescence-associated protein, putative, expressed |
| OsAffx.27743.1.S1_at | 0.284 | 0.219 | 0.094 | 3.413 | LOC_Os06g20900 | hypothetical protein |
| Os.27494.1.S1_at | 0.286 | 0.474 | 0.665 | 1.051 | LOC_Os07g41200 | expressed protein |
| Os.57426.1.S1_s_at | 0.286 | 3.304 | 1.726 | 1.958 | LOC_Os05g13940 | retrotransposon protein, putative, Ty3-gypsy subclass, expressed |
| Os.6450.1.S1_at | 0.288 | 1.139 | 0.896 | 2.507 | LOC_Os07g09190 | 1-deoxy-D-xylulose-5-phosphate synthase, chloroplast precursor, putative, expressed |
| Os.33316.1.S1_at | 0.289 | 3.624 | 0.960 | 1.115 | LOC_Os01g34560 | very-long-chain fatty acid condensing enzyme, putative, expressed |
| Os.12633.1.S1_at | 0.289 | 256.935 | 143.57 7 | 1.561 | LOC_Os11g26790 | Dehydrin family protein, expressed |
| Os.9154.1.S1_at | 0.289 | 2.221 | 2.133 | 2.876 | LOC_Os01g56220 | expressed protein |
| Os.50175.2.S1_at | 0.292 | 1.590 | 2.039 | 0.585 | LOC_Os04g51820 | Cation transport protein, expressed |
| Os.56891.1.S1_at | 0.295 | 1.031 | 0.811 | 1.338 | LOC_Os03g29930 | expressed protein |
| Os.48082.1.S1_at | 0.295 | 1.241 | 4.215 | 1.427 | LOC_Os09g25070 | WRKY DNA binding domain containing protein, expressed |
| Os.53009.1.A1_x_at | 0.295 | 0.078 | 0.444 | 0.883 | LOC_Os02g54640 | Receptor family ligand binding region containing protein, expressed |
| Os.26938.1.A1_at | 0.300 | 0.149 | 0.409 | 0.701 | LOC_Os02g42150 | Calcium binding EGF domain containing protein, expressed |
| Os.53422.1.S1_at | 0.302 | 0.398 | 1.102 | 0.766 | LOC_Os02g15460 | expressed protein |
| OsAffx.24812.1.S1_s_at | 0.307 | 1.044 | 4.093 | 0.044 | LOC_Os02g50460 | U-box domain containing protein, expressed |
| Os.36767.1.S1_at | 0.308 | 4.575 | 2.087 | 2.917 | LOC_Os01g04330 | EF hand family protein, expressed |
| Os.28964.1.S1_at | 0.308 | 1.613 | 3.197 | 1.326 | LOC_Os01g42790 | Papain family cysteine protease containing protein, expressed |
| OsAffx.13360.1.S1_at | 0.309 | 3.190 | 1.047 | 3.493 | LOC_Os03g45280 | Dehydrin family protein, expressed |
| Os.53208.1.S1_at | 0.309 | 0.307 | 0.667 | 0.470 | LOC_Os09g29600 | Calcium binding EGF domain containing protein, expressed |

| ProbeSetID | *OMTN6* -OE[a] | Stress response[b] | | | TIGR Locus ID[c] | Annotation |
|---|---|---|---|---|---|---|
| | | Drought | Salt | Cold | | |
| Os.17112.1.S1_at | 0.310 | 15.064 | 12.497 | 1.767 | LOC_Os03g18030 | oxidoreductase, 2OG-Fe oxygenase family protein, expressed |
| Os.27382.2.S1_at | 0.311 | 0.116 | 0.266 | 1.171 | LOC_Os02g02120 | wall-associated kinase 3, putative, expressed |
| Os.10358.1.A1_at | 0.313 | 0.277 | 0.575 | 0.586 | LOC_Os10g04270 | Jacalin-like lectin domain containing protein, expressed |
| Os.6092.1.S1_at | 0.313 | 1.773 | 0.494 | 7.560 | LOC_Os02g44230 | trehalose-phosphatase family protein, expressed |
| Os.11465.1.S1_at | 0.314 | 1.392 | 0.683 | 1.075 | LOC_Os11g33270 | xyloglucan endotransglucosylase/hydrolase precursor, putative, expressed |
| Os.54463.1.S1_at | 0.315 | 1.321 | 3.216 | 0.667 | LOC_Os05g48680 | expressed protein |
| Os.50575.1.S1_at | 0.315 | 2.252 | 1.239 | 1.780 | LOC_Os04g40700 | expressed protein |
| Os.55991.1.S1_at | 0.317 | 5.346 | 3.803 | 2.449 | LOC_Os10g39090 | expressed protein |
| Os.49322.1.S1_at | 0.318 | 0.511 | 0.660 | 2.426 | LOC_Os09g15050 | Ent-kaurene synthase A, chloroplast precursor, putative, expressed |
| Os.7862.1.S1_at | 0.318 | 7.040 | 1.999 | 4.019 | LOC_Os03g13840 | Senescence-associated protein, expressed |
| Os.52228.1.S1_at | 0.319 | 0.224 | 0.400 | 0.903 | LOC_Os11g36000 | Leucine Rich Repeat family protein, expressed |
| OsAffx.21092.2.S1_at | 0.320 | 0.326 | 0.874 | 0.407 | LOC_Os01g26310 | expressed protein |
| OsAffx.23406.1.S1_at | 0.321 | 0.836 | 0.954 | 1.289 | LOC_Os01g26300 | wall-associated kinase 1, putative, expressed |
| Os.46849.1.S1_at | 0.322 | 5.601 | 6.351 | 5.057 | LOC_Os10g25230 | ZIM motif family protein, expressed |
| OsAffx.7606.1.S1_at | 0.323 | 1.419 | 4.132 | 1.940 | LOC_Os12g15680 | Multicopper oxidase family protein, expressed |
| OsAffx.22380.1.S1_at | 0.323 | 1.580 | 0.910 | 3.608 | LOC_Os07g39680 | XYPPX repeat family protein, expressed |
| Os.52147.1.S1_at | 0.324 | 3.759 | 2.718 | 0.658 | LOC_Os11g30760 | expressed protein |
| OsAffx.14202.1.S1_at | 0.329 | 0.158 | 0.157 | 0.266 | LOC_Os04g39380 | expressed protein |
| Os.27011.1.A1_s_at | 0.329 | 0.551 | 0.802 | 1.551 | LOC_Os01g04550 | serine/threonine protein kinase, putative, expressed |
| Os.6786.1.S1_a_at | 0.330 | 0.268 | 0.332 | 0.780 | LOC_Os10g40720 | Beta-expansin 1a precursor, putative, expressed |
| Os.4377.1.S1_at | 0.332 | 0.482 | 0.557 | 0.726 | LOC_Os02g46970 | 4-coumarate-CoA ligase 2, putative, expressed |
| Os.23008.1.S1_at | 0.332 | 0.746 | 1.288 | 1.907 | LOC_Os10g39710 | Strictosidine synthase family protein, expressed |
| Os.12452.1.S1_s_at | 0.333 | 7.189 | 4.123 | 1.011 | LOC_Os08g36910 | Alpha-amylase isozyme 3D precursor, putative, expressed |
| Os.30000.1.S1_at | 0.333 | 10.395 | 5.383 | 0.794 | LOC_Os07g36560 | Transferase family protein, expressed |

| ProbeSetID | *OMTN6* -OE[a] | Stress response[b] | | | TIGR Locus ID[c] | Annotation |
|---|---|---|---|---|---|---|
| | | Drought | Salt | Cold | | |
| Os.46160.1.S1_at | 0.333 | 0.161 | 0.290 | 1.613 | LOC_Os10g30790 | phosphate:H+ symporter family protein, expressed |
| Os.24180.1.A1_s_at | 0.338 | 0.944 | 0.767 | 1.576 | LOC_Os12g36920 | calmodulin-binding protein, putative, expressed |
| Os.17887.1.S1_at | 0.338 | 3.302 | 1.690 | 4.523 | LOC_Os12g36910 | calmodulin-binding protein, putative, expressed |
| Os.56988.1.S1_at | 0.340 | 1.338 | 0.632 | 1.368 | LOC_Os03g53730 | flavodoxin family protein, expressed |
| OsAffx.21802.1.S1_at | 0.343 | 0.579 | 0.706 | 0.720 | LOC_Os01g66820 | receptor-like protein kinase, putative |
| Os.20557.1.S1_s_at | 0.344 | 8.690 | 2.881 | 1.250 | LOC_Os11g10760 | Leucine Rich Repeat family protein, expressed |
| Os.55408.1.S1_at | 0.344 | 88.632 | 13.552 | 9.601 | LOC_Os04g52750 | expressed protein |
| Os.57506.1.S1_at | 0.345 | 1.272 | 1.540 | 1.610 | LOC_Os06g37300 | Ent-kaurene oxidase, putative, expressed |
| OsAffx.28409.2.S1_s_at | 0.345 | 0.807 | 0.880 | 2.089 | LOC_Os07g12240 | EF hand family protein |
| Os.56815.1.S1_at | 0.346 | 0.212 | 0.297 | 1.331 | LOC_Os06g18960 | expressed protein |
| Os.11575.1.S1_at | 0.347 | 0.870 | 0.497 | 1.425 | LOC_Os03g46440 | BTB/POZ domain containing protein, expressed |
| Os.5905.1.S1_at | 0.347 | 0.897 | 0.223 | 0.877 | LOC_Os06g32990 | Peroxidase family protein, expressed |
| Os.37773.1.S1_at | 0.349 | 1.643 | 7.595 | 2.434 | LOC_Os03g16030 | 17.4 kDa class I heat shock protein, putative, expressed |
| Os.1054.1.A1_at | 0.349 | 3.199 | 2.877 | 0.650 | LOC_Os01g02900 | HGA6, putative, expressed |
| Os.46486.1.S1_at | 0.350 | 0.477 | 0.152 | 1.675 | LOC_Os10g39020 | fringe protein, putative, expressed |
| Os.49413.1.A1_x_at | 0.350 | 3.198 | 5.806 | 0.586 | LOC_Os09g34150 | NB-ARC domain containing protein, expressed |
| Os.50575.2.S1_x_at | 0.350 | 2.209 | 1.371 | 1.948 | LOC_Os04g40700 | expressed protein |
| Os.50572.1.S1_at | 0.350 | 1.092 | 0.941 | 1.701 | LOC_Os12g36920 | calmodulin-binding protein, putative, expressed |
| Os.19369.1.S1_at | 0.351 | 1.610 | 1.080 | 0.969 | LOC_Os12g05990 | No apical meristem protein, expressed |
| OsAffx.1083.1.S1_at | 0.351 | 1.281 | 0.439 | 1.302 | LOC_Os01g66840 | Pectinacetylesterase family protein, expressed |
| Os.50455.1.S1_at | 0.352 | 3.019 | 0.924 | 3.068 | LOC_Os06g40170 | Phospholipase D alpha 2, putative, expressed |
| Os.54385.1.S1_at | 0.352 | 0.889 | 0.715 | 1.847 | LOC_Os04g01320 | D-mannose binding lectin family protein, expressed |
| OsAffx.6035.1.S1_at | 0.355 | 1.662 | 0.441 | 13.993 | LOC_Os08g35580 | expressed protein |
| OsAffx.7406.1.S1_at | 0.355 | 0.116 | 0.353 | 0.340 | LOC_Os11g44300 | hypothetical protein |

| ProbeSetID | *OMTN6* -OE[a] | Stress response[b] | | | TIGR Locus ID[c] | Annotation |
|---|---|---|---|---|---|---|
| | | Drought | Salt | Cold | | |
| Os.15398.1.S1_at | 0.355 | 0.582 | 0.561 | 0.809 | LOC_Os07g17970 | AMP-binding enzyme family protein, expressed |
| Os.15917.1.S1_at | 0.356 | 4.545 | 0.675 | 5.079 | LOC_Os04g41960 | NADP-dependent oxidoreductase P1, putative, expressed |
| Os.3391.1.S1_at | 0.357 | 4.220 | 4.324 | 0.544 | LOC_Os09g23620 | typical P-type R2R3 Myb protein, putative, expressed |
| Os.2211.1.S1_at | 0.359 | 0.568 | 0.573 | 1.727 | LOC_Os08g09010 | Germin-like protein subfamily 1 member 11 precursor, putative, expressed |
| Os.10245.1.S1_at | 0.359 | 1.135 | 2.508 | 1.681 | LOC_Os04g37700 | expressed protein |
| Os.53717.1.S1_at | 0.360 | 0.342 | 0.914 | 0.628 | LOC_Os06g01250 | Cytochrome P450 family protein, expressed |
| Os.17766.1.S1_at | 0.362 | 1.397 | 0.962 | 1.429 | LOC_Os06g12120 | BRASSINOSTEROID INSENSITIVE 1-associated receptor kinase 1 precursor, putative, expressed |
| Os.55654.1.S1_at | 0.362 | 2.813 | 2.875 | 4.313 | LOC_Os04g24300 | Protein kinase domain containing protein, expressed |
| Os.35456.1.S1_at | 0.363 | 0.824 | 0.746 | 1.197 | LOC_Os06g38450 | Papain family cysteine protease containing protein, expressed |
| Os.31858.1.S1_at | 0.365 | 4.131 | 1.885 | 2.555 | LOC_Os07g35340 | Protein kinase domain containing protein, expressed |
| Os.171.1.S1_at | 0.366 | 0.797 | 1.090 | 1.302 | LOC_Os01g47070 | Acidic endochitinase precursor, putative, expressed |
| Os.53148.1.S1_at | 0.366 | 1.119 | 0.648 | 2.101 | LOC_Os03g52720 | magnesium-dependent phosphatase-1 family protein, expressed |
| Os.12751.1.S1_at | 0.367 | 0.676 | 0.803 | 1.181 | LOC_Os02g48470 | expressed protein |
| Os.53887.2.S1_x_at | 0.367 | 6.939 | 1.310 | 1.432 | LOC_Os07g49480 | expressed protein |
| Os.26907.1.S1_at | 0.367 | 0.188 | 0.306 | 0.836 | LOC_Os02g17710 | Leucine Rich Repeat family protein, expressed |
| Os.50799.1.S1_at | 0.367 | 0.588 | 0.375 | 0.816 | LOC_Os04g48130 | membrane protein, putative, expressed |
| Os.32819.1.S1_at | 0.368 | 1.246 | 1.263 | 1.045 | LOC_Os07g36590 | KI domain interacting kinase 1, putative, expressed |
| Os.40417.1.A1_at | 0.368 | 0.550 | 0.322 | 1.073 | LOC_Os07g14740 | Harpin-induced protein 1 containing protein, expressed |
| OsAffx.15485.1.S1_at | 0.368 | 0.729 | 0.585 | 1.585 | LOC_Os06g18900 | embryogenesis transmembrane protein, putative, expressed |
| Os.23977.1.S1_at | 0.369 | 0.378 | 0.538 | 0.763 | LOC_Os03g43510 | expressed protein |
| Os.12724.1.S1_a_at | 0.369 | 2.123 | 2.034 | 1.030 | LOC_Os08g36320 | Glutamate decarboxylase, putative, expressed |
| OsAffx.8290.1.S1_at | 0.370 | 1.580 | 1.037 | 0.370 | LOC_Os10g17960 | receptor-like protein kinase homolog RK20-1, putative, expressed |
| OsAffx.11789.1.S1_s_at | 0.372 | 0.092 | 0.083 | 0.712 | LOC_Os01g68470 | hypothetical protein |

| ProbeSetID | *OMTN6* -OE[a] | Stress response[b] | | | TIGR Locus ID[c] | Annotation |
|---|---|---|---|---|---|---|
| | | Drought | Salt | Cold | | |
| Os.32267.1.S1_at | 0.372 | 1.583 | 0.967 | 1.346 | LOC_Os01g56330 | protein kinase family protein, putative, expressed |
| Os.11354.1.S1_at | 0.373 | 1.361 | 0.188 | 7.684 | LOC_Os04g51460 | xyloglucan endotransglucosylase/hydrolase protein 15precursor, putative, expressed |
| Os.10416.1.S1_at | 0.373 | 0.924 | 0.725 | 2.495 | LOC_Os01g61850 | expressed protein |
| Os.8597.1.S1_at | 0.374 | 0.479 | 0.516 | 1.271 | LOC_Os03g02040 | Remorin, C-terminal region family protein, expressed |
| Os.26810.1.A1_s_at | 0.374 | 0.333 | 0.619 | 1.059 | LOC_Os02g16940 | Subtilisin N-terminal Region family protein, expressed |
| OsAffx.6270.1.S1_at | 0.376 | 0.318 | 0.435 | 0.368 | LOC_Os09g14450 | NB-ARC domain containing protein, expressed |
| OsAffx.17220.1.S1_at | 0.376 | 3.300 | 3.450 | 0.550 | LOC_Os08g29040 | Protein kinase domain containing protein |
| Os.17900.1.S1_s_at | 0.379 | 0.738 | 0.940 | 0.174 | LOC_Os05g35690 | Gibberellin-regulated protein 2 precursor, putative, expressed |
| Os.51787.1.S1_at | 0.379 | 0.435 | 0.444 | 1.350 | LOC_Os12g24320 | Cell Division Protein AAA ATPase family, putative, expressed |
| Os.7348.1.S1_at | 0.379 | 3.093 | 1.127 | 6.184 | LOC_Os08g38910 | O-methyltransferase family protein, expressed |
| Os.24878.1.A1_at | 0.380 | 0.738 | 1.013 | 1.470 | LOC_Os01g41750 | Leucine Rich Repeat family protein, expressed |
| Os.27175.1.S1_a_at | 0.381 | 0.295 | 0.562 | 1.051 | LOC_Os07g12890 | ZIP zinc/iron transport family protein, expressed |
| Os.9456.1.S1_at | 0.381 | 4.517 | 1.775 | 1.354 | LOC_Os02g28170 | Transferase family protein, expressed |
| Os.46956.1.S1_at | 0.381 | 31.068 | 5.481 | 3.056 | LOC_Os01g50940 | Helix-loop-helix DNA-binding domain containing protein, expressed |
| OsAffx.17563.1.S1_at | 0.382 | 0.241 | 0.725 | 0.128 | LOC_Os09g04430 | hypothetical protein |
| OsAffx.7414.1.S1_at | 0.382 | 0.129 | 0.259 | 0.345 | LOC_Os11g44700 | expressed protein |
| Os.32801.1.S1_x_at | 0.384 | 1.697 | 0.949 | 2.881 | LOC_Os01g54700 | transposon protein, putative, CACTA, En/Spm sub-class, expressed |
| Os.19575.1.A1_at | 0.386 | 2.288 | 1.662 | 1.322 | LOC_Os04g30010 | Calcium binding EGF domain containing protein, expressed |
| Os.1191.1.S1_at | 0.387 | 1.164 | 1.075 | 1.912 | LOC_Os03g04060 | Basic endochitinase precursor, putative, expressed |
| Os.8957.1.S1_at | 0.387 | 8.261 | 2.533 | 2.136 | LOC_Os01g63060 | phosphatidic acid phosphatase, putative, expressed |
| Os.8202.1.S1_at | 0.388 | 2.181 | 1.704 | 1.680 | LOC_Os12g37560 | phospholipase C, putative, expressed |
| OsAffx.18575.1.S1_x_at | 0.388 | 4.573 | 1.433 | 1.803 | LOC_Os10g39140 | oxidoreductase, 2OG-Fe oxygenase family protein, expressed |
| Os.26543.1.S1_at | 0.388 | 0.891 | 0.397 | 1.177 | LOC_Os03g58110 | Leucine Rich Repeat family protein, expressed |

| ProbeSetID | *OMTN6* -OE[a] | Stress response[b] | | | TIGR Locus ID[c] | Annotation |
|---|---|---|---|---|---|---|
| | | Drought | Salt | Cold | | |
| Os.11309.1.S1_x_at | 0.389 | 1.181 | 0.961 | 0.901 | LOC_Os05g04490 | Peroxidase family protein, expressed |
| Os.9776.1.S1_a_at | 0.392 | 0.264 | 0.559 | 1.456 | LOC_Os03g32470 | oxidoreductase, 2OG-Fe oxygenase family protein, expressed |
| Os.9533.1.S1_at | 0.392 | 0.949 | 1.477 | 1.911 | LOC_Os03g02750 | Subtilisin N-terminal Region family protein, expressed |
| Os.55984.1.S1_at | 0.393 | 3.634 | 2.166 | 2.055 | LOC_Os05g46840 | expressed protein |
| Os.6418.1.S1_at | 0.393 | 9.068 | 3.843 | 0.848 | LOC_Os01g07530 | Raffinose synthase or seed imbibition protein Sip1 containing protein, expressed |
| Os.49973.1.S1_at | 0.393 | 0.879 | 0.436 | 1.258 | LOC_Os03g20210 | Eukaryotic aspartyl protease family protein, expressed |
| Os.53763.1.A1_at | 0.394 | 2.714 | 0.229 | 0.314 | LOC_Os06g38340 | Leucine Rich Repeat family protein, expressed |
| Os.12038.1.S1_at | 0.394 | 1.198 | 1.136 | 0.730 | LOC_Os01g65700 | expressed protein |
| Os.26382.1.S1_at | 0.395 | 0.869 | 0.811 | 1.059 | LOC_Os05g12680 | Protein kinase domain containing protein, expressed |
| OsAffx.2891.1.S1_s_at | 0.395 | 0.822 | 1.243 | 2.211 | LOC_Os02g37330 | heavy metal-associated domain containing protein, expressed |
| Os.15943.1.S1_at | 0.395 | 0.533 | 0.961 | 1.654 | LOC_Os01g67310 | Patatin-like phospholipase family protein, expressed |
| Os.27799.1.S1_at | 0.396 | 0.743 | 0.619 | 1.254 | LOC_Os04g52190 | Vacuolar sorting receptor 7 precursor, putative, expressed |
| Os.18178.1.S1_at | 0.396 | 3.665 | 1.087 | 0.947 | LOC_Os03g03550 | bZIP family transcription factor, putative, expressed |
| Os.9514.1.S1_at | 0.397 | 0.526 | 0.736 | 1.181 | LOC_Os05g04820 | Myb-like DNA-binding domain containing protein, expressed |
| Os.33145.1.S1_at | 0.397 | 3.483 | 4.395 | 1.273 | LOC_Os07g36570 | D-mannose binding lectin family protein, expressed |
| OsAffx.15826.1.S1_at | 0.397 | 0.818 | 0.409 | 1.000 | LOC_Os06g38870 | hypothetical protein |
| Os.7095.1.S1_at | 0.398 | 6.341 | 6.429 | 1.600 | LOC_Os04g49210 | oxidoreductase, 2OG-Fe oxygenase family protein, expressed |
| Os.57142.1.S1_at | 0.399 | 0.535 | 0.853 | 1.806 | LOC_Os06g45970 | Auxin responsive protein, expressed |
| Os.9923.1.S1_s_at | 0.399 | 3.257 | 2.200 | 4.491 | LOC_Os03g08330 | ZIM motif family protein, expressed |
| Os.3721.1.S1_at | 0.399 | 1.451 | 1.140 | 1.466 | LOC_Os01g03980 | expressed protein |
| Os.46551.1.S1_at | 0.399 | 0.120 | 0.190 | 0.229 | LOC_Os10g17260 | Flavonoid 3'-monooxygenase, putative, expressed |
| OsAffx.26318.1.S1_at | 0.400 | 2.024 | 1.362 | 0.937 | LOC_Os04g34000 | digalactosyldiacylglycerol synthase 1, putative, expressed |
| Os.54493.1.S1_at | 0.400 | 0.898 | 0.671 | 1.993 | LOC_Os06g14490 | calmodulin-binding heat-shock protein, putative, expressed |

| ProbeSetID | *OMTN6* -OE[a] | Stress response[b] | | | TIGR Locus ID[c] | Annotation |
|---|---|---|---|---|---|---|
| | | Drought | Salt | Cold | | |
| OsAffx.19285.1.S1_at | 0.400 | 0.808 | 2.512 | 1.146 | LOC_Os11g40810 | Leucine Rich Repeat family protein, expressed |
| Os.24971.1.A1_s_at | 0.402 | 0.477 | 0.565 | 0.322 | LOC_Os04g29960 | Calcium binding EGF domain containing protein, expressed |
| Os.10546.1.S1_s_at | 0.402 | 5.328 | 0.988 | 1.402 | LOC_Os09g34230 | UDP-glucoronosyl and UDP-glucosyl transferase family protein, expressed |
| Os.27986.1.A1_at | 0.402 | 0.730 | 0.835 | 0.984 | LOC_Os09g32550 | Glycosyl hydrolases family 17 protein, expressed |
| Os.52509.1.S1_at | 0.403 | 1.306 | 0.981 | 0.794 | LOC_Os04g33950 | Transcription factor E2F/dimerisation partner family protein, expressed |
| Os.54269.1.S1_s_at | 0.403 | 2.176 | 1.297 | 1.255 | LOC_Os05g48060 | Phosphatidyl serine synthase family protein, expressed |
| OsAffx.12721.1.S1_at | 0.404 | 2.570 | 1.367 | 1.457 | LOC_Os03g03510 | CIPK-like protein 1, putative, expressed |
| Os.9046.1.S1_x_at | 0.404 | 2.156 | 1.676 | 0.986 | LOC_Os10g22980 | Leucine Rich Repeat family protein, expressed |
| Os.23790.1.S1_at | 0.404 | 1.350 | 0.915 | 1.198 | LOC_Os10g29650 | retrotransposon protein, putative, unclassified, expressed |
| Os.14576.1.S1_at | 0.405 | 0.897 | 0.802 | 1.039 | LOC_Os09g07380 | ABI3-interacting protein 2, putative, expressed |
| Os.27797.1.A1_at | 0.405 | 2.723 | 2.578 | 1.053 | LOC_Os03g06570 | IQ calmodulin-binding motif family protein, expressed |
| OsAffx.19473.1.S1_at | 0.405 | 1.539 | 1.309 | 1.893 | LOC_Os12g03740 | F-box domain containing protein, expressed |
| Os.2653.1.S1_at | 0.405 | 1.160 | 0.884 | 1.839 | LOC_Os10g39520 | MLO-like protein 10, putative, expressed |
| Os.6075.1.S1_at | 0.407 | 3.281 | 1.502 | 1.893 | LOC_Os04g06520 | expressed protein |
| OsAffx.17899.1.S1_at | 0.407 | 0.870 | 1.141 | 1.167 | LOC_Os09g25930 | Ligand-gated ion channel family protein |
| OsAffx.2803.1.S1_at | 0.407 | 0.439 | 1.314 | 1.029 | LOC_Os02g30190 | hypothetical protein |
| Os.648.1.S1_at | 0.408 | 7.494 | 3.226 | 2.188 | LOC_Os01g43480 | ATPase, AAA family protein, expressed |
| Os.34462.1.S1_at | 0.409 | 4.930 | 1.073 | 1.361 | LOC_Os01g62950 | Ras-related protein Rab11C, putative, expressed |
| OsAffx.16015.1.S1_at | 0.409 | 0.607 | 4.093 | 4.252 | LOC_Os06g51070 | No apical meristem protein, expressed |
| Os.27059.1.S1_at | 0.409 | 1.113 | 0.941 | 1.091 | LOC_Os08g10070 | Protein kinase domain containing protein, expressed |
| Os.54934.1.S1_at | 0.410 | 57.309 | 36.282 | 3.129 | LOC_Os05g37060 | Myb-like DNA-binding domain containing protein, expressed |
| Os.13708.1.S1_at | 0.410 | 1.034 | 0.643 | 1.287 | LOC_Os03g14250 | expressed protein |
| Os.50342.1.S1_at | 0.410 | 1.618 | 2.276 | 0.727 | LOC_Os04g52780 | Leucine Rich Repeat family protein, expressed |
| Os.12642.1.S1_at | 0.410 | 8.266 | 2.362 | 5.968 | LOC_Os08g42750 | Calcium-dependent protein kinase, isoform AK1, putative, expressed |

| ProbeSetID | *OMTN6* -OE[a] | Stress response[b] | | | TIGR Locus ID[c] | Annotation |
|---|---|---|---|---|---|---|
| | | Drought | Salt | Cold | | |
| Os.2225.1.S1_at | 0.410 | 2.697 | 1.320 | 1.242 | LOC_Os04g44870 | Elicitor-responsive protein 3, putative, expressed |
| Os.14971.1.S1_at | 0.410 | 0.648 | 0.363 | 1.566 | LOC_Os08g40850 | Mitochondrial carrier protein, expressed |
| Os.27767.1.A1_s_at | 0.410 | 0.107 | 0.663 | 0.826 | LOC_Os04g29680 | Calcium binding EGF domain containing protein, expressed |
| Os.28301.1.S1_at | 0.411 | 0.225 | 0.520 | 1.377 | LOC_Os07g05360 | Photosystem II 10 kDa polypeptide, chloroplast precursor, putative, expressed |
| OsAffx.29016.1.S1_at | 0.411 | 1.325 | 1.152 | 1.424 | LOC_Os08g03600 | magnesium transporter CorA, putative, expressed |
| Os.49527.1.S1_at | 0.412 | 0.143 | 0.069 | 0.916 | LOC_Os03g03790 | acyl-activating enzyme 11, putative, expressed |
| OsAffx.13523.1.S1_at | 0.412 | 1.984 | 0.889 | 1.238 | LOC_Os03g57400 | hypothetical protein |
| OsAffx.3951.1.S1_at | 0.413 | 0.702 | 0.226 | 0.806 | LOC_Os04g30340 | wall-associated kinase 2, putative |
| Os.30909.1.S1_at | 0.413 | 0.504 | 1.065 | 0.187 | LOC_Os01g09190 | expressed protein |
| Os.9511.1.S1_at | 0.413 | 0.904 | 0.686 | 1.889 | LOC_Os03g40670 | Glycerophosphoryl diester phosphodiesterase family protein, expressed |
| Os.54530.1.S1_at | 0.413 | 0.945 | 0.691 | 0.602 | LOC_Os06g49100 | leucine-rich repeat family protein, putative, expressed |
| Os.25316.2.S1_x_at | 0.414 | 1.367 | 1.161 | 0.853 | LOC_Os03g56260 | Kinesin motor domain containing protein, expressed |
| Os.34139.1.S1_at | 0.414 | 10.314 | 4.954 | 1.162 | LOC_Os08g04630 | EF hand family protein, expressed |
| Os.12201.1.S1_at | 0.414 | 2.447 | 1.470 | 1.403 | LOC_Os09g27750 | 1-aminocyclopropane-1-carboxylate oxidase 1, putative, expressed |
| Os.7678.1.S1_at | 0.415 | 2.222 | 2.997 | 6.049 | LOC_Os03g12500 | Cytochrome P450 74A2, putative, expressed |
| Os.34779.1.S1_at | 0.416 | 0.839 | 2.607 | 0.607 | LOC_Os01g06520 | Leucine Rich Repeat family protein, expressed |
| Os.51063.1.S1_at | 0.417 | 22.692 | 10.647 | 4.776 | LOC_Os09g28210 | Helix-loop-helix DNA-binding domain containing protein, expressed |
| Os.12277.1.S1_at | 0.417 | 1.967 | 1.438 | 1.845 | LOC_Os06g04070 | Arginine decarboxylase, putative, expressed |
| Os.27112.1.S1_at | 0.417 | 1.923 | 2.318 | 1.247 | LOC_Os01g09800 | Regulatory protein NPR1, putative, expressed |
| OsAffx.4214.1.S1_x_at | 0.418 | 0.691 | 0.865 | 1.070 | LOC_Os04g59330 | expressed protein |
| Os.27509.1.S1_at | 0.418 | 1.075 | 1.417 | 1.319 | LOC_Os01g53790 | expressed protein |
| Os.15872.1.S1_at | 0.418 | 1.094 | 0.920 | 1.450 | LOC_Os05g50710 | Late embryogenesis abundant protein Lea14-A, putative, expressed |
| Os.46574.1.S1_at | 0.419 | 0.180 | 0.527 | 1.105 | LOC_Os10g42040 | expressed protein |
| Os.11246.1.S1_at | 0.419 | 1.115 | 0.775 | 0.496 | LOC_Os01g02940 | glycosyltransferase, putative, expressed |

| ProbeSetID | *OMTN6* -OE[a] | Stress response[b] | | | TIGR Locus ID[c] | Annotation |
|---|---|---|---|---|---|---|
| | | Drought | Salt | Cold | | |
| Os.5360.2.S1_at | 0.419 | 1.378 | 0.987 | 0.592 | LOC_Os09g26660 | respiratory burst oxidase protein E, putative, expressed |
| Os.14210.1.S1_at | 0.419 | 0.847 | 1.132 | 3.718 | LOC_Os10g36360 | expressed protein |
| Os.23209.1.S1_at | 0.419 | 0.383 | 0.368 | 0.787 | LOC_Os05g39350 | expressed protein |
| Os.4683.2.S1_at | 0.420 | 0.925 | 0.695 | 0.888 | LOC_Os01g42520 | expressed protein |
| Os.10747.1.S1_s_at | 0.420 | 4.549 | 1.871 | 1.779 | LOC_Os08g38220 | Dof domain, zinc finger family protein, expressed |
| Os.6035.1.S1_at | 0.421 | 0.391 | 0.690 | 0.662 | LOC_Os07g46350 | Serine carboxypeptidase II-3 precursor, putative, expressed |
| Os.31670.2.S1_at | 0.422 | 1.196 | 0.901 | 0.994 | LOC_Os01g43330 | expressed protein |
| Os.4651.1.S1_at | 0.422 | 17.742 | 1.580 | 3.231 | LOC_Os07g48710 | VQ motif family protein, expressed |
| Os.49400.1.S2_s_at | 0.422 | 1.133 | 0.830 | 0.786 | LOC_Os11g11960 | NBS-LRR type disease resistance protein, putative, expressed |
| Os.21066.1.S1_at | 0.423 | 1.137 | 1.242 | 1.610 | LOC_Os03g56250 | wound and phytochrome signaling involved receptor like kinase, putative, expressed |
| Os.30572.1.S1_at | 0.423 | 1.216 | 0.811 | 0.634 | LOC_Os01g50610 | SAM dependent carboxyl methyltransferase family protein, expressed |
| Os.26411.1.A1_at | 0.423 | 38.364 | 20.293 | 2.357 | LOC_Os08g28710 | Protein kinase domain containing protein, expressed |
| OsAffx.5948.1.S1_at | 0.424 | 0.981 | 0.858 | 1.317 | LOC_Os08g27170 | calmodulin-binding protein, putative |
| Os.51866.1.S1_at | 0.424 | 9.248 | 5.017 | 2.709 | LOC_Os11g10770 | NB-ARC domain containing protein, expressed |
| Os.52699.1.S1_at | 0.424 | 0.928 | 0.737 | 1.267 | LOC_Os04g55420 | Leucine Rich Repeat family protein, expressed |
| Os.19001.1.S1_at | 0.424 | 1.622 | 1.076 | 1.040 | LOC_Os05g31890 | expressed protein |
| OsAffx.23947.1.S1_at | 0.424 | 0.753 | 0.384 | 1.465 | LOC_Os01g66740 | Leucine Rich Repeat family protein |
| OsAffx.31475.1.S1_at | 0.425 | 0.927 | 1.232 | 1.277 | LOC_Os11g44630 | hypothetical protein |
| Os.50346.1.S1_at | 0.426 | 3.223 | 1.628 | 1.876 | LOC_Os05g04680 | expressed protein |
| Os.30059.2.S1_x_at | 0.426 | 1.027 | 0.681 | 0.829 | LOC_Os01g60420 | expressed protein |
| Os.26900.1.A1_at | 0.426 | 0.757 | 0.393 | 1.131 | LOC_Os09g16950 | lectin protein kinase, putative, expressed |
| Os.6376.1.S1_at | 0.427 | 14.447 | 3.975 | 1.809 | LOC_Os06g10210 | expressed protein |
| Os.11322.1.S1_at | 0.427 | 1.168 | 0.685 | 1.311 | LOC_Os04g50950 | POT family protein, expressed |

| ProbeSetID | *OMTN6* -OE[a] | Stress response[b] | | | TIGR Locus ID[c] | Annotation |
|---|---|---|---|---|---|---|
| | | Drought | Salt | Cold | | |
| Os.50287.1.S1_at | 0.428 | 4.973 | 2.965 | 5.186 | LOC_Os04g39010 | heavy metal-associated domain containing protein, expressed |
| Os.53337.1.S1_at | 0.429 | 1.609 | 0.203 | 1.563 | LOC_Os02g17090 | Subtilisin N-terminal Region family protein, expressed |
| Os.32736.1.S1_at | 0.430 | 0.526 | 0.456 | 0.951 | LOC_Os03g56820 | Fatty acid hydroxylase family protein, expressed |
| OsAffx.31710.1.S1_x_at | 0.430 | 5.188 | 2.823 | 3.467 | LOC_Os12g12260 | Diacylglycerol kinase 1, putative, expressed |
| Os.17393.1.S1_at | 0.431 | 1.762 | 1.119 | 2.108 | LOC_Os01g12810 | leaf protein, putative, expressed |
| Os.46840.1.S1_x_at | 0.431 | 0.338 | 0.425 | 0.884 | LOC_Os10g34700 | expressed protein |
| Os.19497.1.S1_at | 0.432 | 0.977 | 0.629 | 1.161 | LOC_Os01g72380 | expressed protein |
| Os.23778.1.S1_at | 0.433 | 2.671 | 0.846 | 3.101 | LOC_Os02g32590 | heat stress transcription factor A3, putative, expressed |
| OsAffx.28760.2.S1_at | 0.433 | 2.087 | 6.879 | 0.656 | LOC_Os07g35180 | hypothetical protein |
| OsAffx.12887.2.S1_s_at | 0.434 | 25.013 | 17.562 | 1.386 | LOC_Os03g16920 | Heat shock cognate 70 kDa protein, putative, expressed |
| Os.5592.1.S1_at | 0.434 | 2.292 | 1.751 | 0.681 | LOC_Os02g48710 | expressed protein |
| Os.32141.1.S1_at | 0.435 | 1.144 | 0.862 | 1.241 | LOC_Os03g49610 | Glycosyl hydrolase family 1 protein, expressed |
| Os.10322.1.S1_s_at | 0.435 | 4.503 | 2.836 | 3.131 | LOC_Os07g38170 | Remorin, C-terminal region family protein, expressed |
| Os.39087.1.S1_at | 0.436 | 1.762 | 1.344 | 1.987 | LOC_Os01g14550 | Pathogen-related protein, putative, expressed |
| OsAffx.27219.1.S1_at | 0.436 | 50.407 | 27.327 | 1.124 | LOC_Os05g38290 | Protein phosphatase 2C, putative, expressed |
| Os.27177.1.S1_at | 0.436 | 6.058 | 2.417 | 1.819 | LOC_Os03g16390 | expressed protein |
| Os.26755.1.S1_at | 0.436 | 0.280 | 0.700 | 0.626 | LOC_Os01g48610 | expressed protein |
| Os.55829.1.S1_at | 0.438 | 3.196 | 1.379 | 2.648 | LOC_Os05g30760 | hydrolase, alpha/beta fold family protein, expressed |
| Os.6210.1.S1_at | 0.438 | 0.255 | 0.440 | 0.593 | LOC_Os03g56270 | Receptor protein kinase CLAVATA1 precursor, putative, expressed |
| Os.23207.1.S1_at | 0.439 | 10.042 | 5.885 | 1.391 | LOC_Os08g04340 | Plastocyanin-like domain containing protein, expressed |
| Os.21634.1.S1_at | 0.439 | 9.253 | 5.817 | 2.315 | LOC_Os01g55240 | Gibberellin 2-beta-dioxygenase, putative, expressed |
| Os.53114.1.S1_at | 0.439 | 1.014 | 0.855 | 1.318 | LOC_Os07g48610 | hydrolase, alpha/beta fold family protein, expressed |
| Os.11668.1.S1_at | 0.440 | 0.762 | 0.934 | 1.157 | LOC_Os03g19390 | Seed maturation protein PM36, putative, expressed |
| Os.9719.1.S1_at | 0.441 | 0.500 | 0.520 | 0.701 | LOC_Os07g47460 | expressed protein |

| ProbeSetID | *OMTN6* -OE[a] | Stress response[b] | | | TIGR Locus ID[c] | Annotation |
|---|---|---|---|---|---|---|
| | | Drought | Salt | Cold | | |
| Os.17997.2.S1_x_at | 0.442 | 1.430 | 1.551 | 1.042 | LOC_Os10g26570 | Phosphofructokinase family protein, expressed |
| Os.49265.1.S2_a_at | 0.442 | 0.233 | 0.714 | 0.896 | LOC_Os03g61720 | Acyltransferase family protein, expressed |
| Os.14195.1.S1_at | 0.442 | 0.669 | 1.073 | 1.587 | LOC_Os03g19200 | TPR Domain containing protein, expressed |
| Os.49812.1.S1_at | 0.442 | 2.394 | 1.440 | 2.817 | LOC_Os01g33110 | Protein kinase domain containing protein, expressed |
| Os.21774.1.S1_at | 0.443 | 1.024 | 0.829 | 1.478 | LOC_Os03g18310 | 6-phosphofructo-2-kinase family protein, expressed |
| OsAffx.10980.1.S1_at | 0.443 | 1.235 | 1.330 | 3.191 | LOC_Os01g09150 | hypothetical protein |
| Os.17763.1.S1_at | 0.444 | 1.556 | 1.874 | 1.237 | LOC_Os12g05420 | calmodulin-binding family protein, putative, expressed |
| Os.23518.1.A1_at | 0.445 | 0.151 | 0.473 | 0.710 | LOC_Os02g36190 | Cytochrome P450 family protein, expressed |
| Os.46776.1.S1_s_at | 0.446 | 0.299 | 0.372 | 1.037 | LOC_Os09g26960 | Cytochrome P450 family protein, expressed |
| Os.49253.1.S1_at | 0.446 | 1.231 | 0.697 | 0.789 | LOC_Os06g12390 | Galactoside-binding lectin family protein, expressed |
| Os.10054.1.S1_at | 0.447 | 2.876 | 0.768 | 1.099 | LOC_Os02g46650 | Ubiquitin carboxyl-terminal hydrolase family protein, expressed |
| Os.36033.1.S1_at | 0.447 | 0.635 | 1.191 | 0.907 | LOC_Os01g49910 | Protein kinase domain containing protein, expressed |
| Os.17826.1.A1_at | 0.447 | 0.755 | 0.912 | 0.667 | LOC_Os02g04690 | Cycloartenol synthase, putative, expressed |
| Os.15125.1.S1_at | 0.448 | 0.328 | 0.223 | 0.636 | LOC_Os04g07110 | expressed protein |
| Os.31171.1.S1_at | 0.449 | 0.538 | 0.992 | 1.506 | LOC_Os01g40290 | expressed protein |
| Os.44751.1.S1_at | 0.450 | 0.959 | 0.901 | 1.390 | LOC_Os06g06760 | U-box domain containing protein, expressed |
| Os.14285.2.S1_at | 0.450 | 0.406 | 0.511 | 1.465 | LOC_Os04g31550 | expressed protein |
| OsAffx.28714.1.S1_x_at | 0.451 | 2.187 | 1.946 | 0.769 | LOC_Os07g32570 | 5'-adenylylsulfate reductase 2, chloroplast precursor, putative, expressed |
| Os.32454.1.S1_at | 0.451 | 0.545 | 0.782 | 1.234 | LOC_Os01g01650 | Isoflavone reductase homolog IRL, putative, expressed |
| Os.45238.1.S1_at | 0.451 | 2.479 | 1.641 | 2.560 | LOC_Os03g28270 | Leucine Rich Repeat family protein, expressed |
| Os.609.3.S1_a_at | 0.452 | 2.656 | 1.656 | 1.838 | LOC_Os08g37670 | Plastocyanin-like domain containing protein, expressed |
| Os.46627.1.S1_at | 0.452 | 2.347 | 1.432 | 1.300 | LOC_Os10g40640 | Glycogenin, putative, expressed |
| Os.19954.1.S1_at | 0.452 | 1.100 | 0.778 | 0.389 | LOC_Os09g28690 | 2-Hydroxyisoflavanone dehydratase, putative, expressed |
| Os.49496.1.S1_at | 0.453 | 0.474 | 0.540 | 0.970 | LOC_Os06g46500 | L-ascorbate oxidase homolog precursor, putative, expressed |

| ProbeSetID | *OMTN6* -OE[a] | Stress response[b] | | | TIGR Locus ID[c] | Annotation |
|---|---|---|---|---|---|---|
| | | Drought | Salt | Cold | | |
| Os.51022.1.A1_at | 0.453 | 0.588 | 0.588 | 6.882 | LOC_Os09g29520 | wall-associated kinase 3, putative, expressed |
| Os.11657.1.S1_at | 0.453 | 4.788 | 3.002 | 2.751 | LOC_Os01g47760 | glutaredoxin family protein, putative, expressed |
| Os.4695.1.S1_s_at | 0.453 | 3.282 | 1.768 | 2.395 | LOC_Os03g28940 | ZIM motif family protein, expressed |
| Os.56210.1.S1_at | 0.455 | 3.781 | 17.738 | 2.172 | LOC_Os04g42950 | Myb protein, putative, expressed |
| Os.2321.1.S1_at | 0.455 | 0.659 | 0.543 | 1.943 | LOC_Os08g09060 | Germin-like protein subfamily 1 member 11 precursor, putative, expressed |
| Os.52536.1.S1_at | 0.455 | 0.488 | 0.307 | 0.968 | LOC_Os04g43800 | Phenylalanine ammonia-lyase, putative, expressed |
| OsAffx.9584.1.S1_at | 0.455 | 2.371 | 0.902 | 4.423 | LOC_Os01g60600 | WRKY DNA binding domain containing protein, expressed |
| Os.11520.1.S1_at | 0.455 | 0.207 | 0.433 | 0.680 | LOC_Os05g12580 | hypothetical protein |
| OsAffx.5607.1.S1_x_at | 0.455 | 11.223 | 3.489 | 1.433 | LOC_Os07g42280 | von Willebrand factor type A domain containing protein, expressed |
| Os.34196.1.S1_at | 0.455 | 0.989 | 0.697 | 2.237 | LOC_Os01g60110 | expressed protein |
| Os.37872.1.S1_at | 0.457 | 6.281 | 2.762 | 1.102 | LOC_Os06g08280 | Protein kinase domain containing protein, expressed |
| Os.53174.1.A1_s_at | 0.457 | 0.541 | 0.841 | 1.116 | LOC_Os02g48350 | Diacylglycerol acyltransferase family protein, expressed |
| Os.51809.1.S1_at | 0.457 | 1.842 | 2.377 | 1.091 | LOC_Os11g06780 | Protein kinase domain containing protein, expressed |
| Os.44937.1.S1_at | 0.458 | 0.905 | 1.192 | 1.207 | LOC_Os03g60810 | Protein kinase domain containing protein, expressed |
| Os.53673.1.S1_at | 0.458 | 2.416 | 2.377 | 2.022 | LOC_Os04g01310 | D-mannose binding lectin family protein, expressed |
| Os.26787.2.S1_x_at | 0.458 | 0.608 | 1.616 | 1.057 | LOC_Os06g36270 | Leucine Rich Repeat family protein, expressed |
| Os.49406.1.S1_at | 0.458 | 1.239 | 0.873 | 0.576 | LOC_Os02g29510 | Non-imprinted in Prader-Willi/Angelman syndrome region protein 2 homolog, putative, expressed |
| Os.55059.1.S1_at | 0.458 | 4.465 | 1.370 | 0.624 | LOC_Os03g53540 | expressed protein |
| Os.15803.1.S1_at | 0.458 | 0.560 | 0.634 | 1.275 | LOC_Os02g49950 | U-box domain containing protein, expressed |
| Os.820.1.S1_s_at | 0.459 | 1.069 | 0.868 | 1.220 | LOC_Os01g19020 | Peroxidase family protein, expressed |
| Os.49583.1.S1_at | 0.459 | 0.908 | 0.758 | 1.751 | LOC_Os03g15080 | expressed protein |
| OsAffx.19579.1.S1_at | 0.460 | 5.257 | 4.009 | 1.092 | LOC_Os12g09640 | Protein phosphatase 2C containing protein, expressed |
| Os.5095.1.S1_at | 0.460 | 0.521 | 0.643 | 0.778 | LOC_Os07g40130 | transposon protein, putative, CACTA, En/Spm sub-class |

| ProbeSetID | *OMTN6* -OE[a] | Stress response[b] | | | TIGR Locus ID[c] | Annotation |
|---|---|---|---|---|---|---|
| | | Drought | Salt | Cold | | |
| Os.11986.2.S1_x_at | 0.460 | 0.435 | 0.918 | 0.873 | LOC_Os01g49590 | receptor serine/threonine kinase PR5K, putative, expressed |
| OsAffx.30204.1.S1_at | 0.460 | 1.521 | 0.827 | 1.311 | LOC_Os09g39190 | Copine family protein, expressed |
| Os.15295.2.S1_x_at | 0.460 | 1.727 | 1.105 | 3.192 | LOC_Os08g19670 | expressed protein |
| Os.27067.2.A1_at | 0.460 | 0.472 | 0.723 | 0.865 | LOC_Os08g02230 | plant-specific FAD-dependent oxidoreductase family protein, expressed |
| Os.10384.1.S1_at | 0.461 | 3.570 | 2.101 | 2.041 | LOC_Os03g10950 | protein phosphatase 2C family protein, putative, expressed |
| Os.2678.1.S1_at | 0.461 | 0.537 | 0.585 | 0.758 | LOC_Os02g13870 | Aquaporin NIP1.2, putative, expressed |
| Os.12167.1.S1_at | 0.461 | 8.843 | 5.536 | 2.456 | LOC_Os02g44870 | Dehydrin family protein, expressed |
| Os.36960.1.S1_at | 0.461 | 0.204 | 1.196 | 0.990 | LOC_Os01g49230 | Limonoid UDP-glucosyltransferase, putative, expressed |
| Os.15216.1.S1_at | 0.463 | 2.200 | 1.785 | 0.884 | LOC_Os05g36190 | Tubby protein, putative, expressed |
| Os.18717.2.S1_at | 0.463 | 1.557 | 3.307 | 0.634 | LOC_Os09g28180 | D-mannose binding lectin family protein, expressed |
| Os.55254.1.S1_at | 0.463 | 1.036 | 1.313 | 1.133 | LOC_Os06g10130 | expressed protein |
| OsAffx.2268.1.S1_at | 0.463 | 0.338 | 0.916 | 0.297 | LOC_Os01g47820 | S-locus-like receptor protein kinase, putative, expressed |
| Os.17681.1.S1_at | 0.463 | 0.409 | 0.664 | 0.821 | LOC_Os04g38220 | Sugar transporter family protein, expressed |
| OsAffx.18423.1.S1_at | 0.463 | 0.527 | 0.872 | 1.115 | LOC_Os10g27430 | expressed protein |
| OsAffx.6930.1.S1_at | 0.464 | 0.601 | 0.731 | 0.939 | LOC_Os10g40810 | GATA zinc finger family protein, expressed |
| Os.55085.1.S1_at | 0.464 | 0.322 | 1.373 | 2.271 | LOC_Os09g01580 | hypothetical protein |
| OsAffx.21790.1.S1_at | 0.464 | 1.252 | 0.981 | 1.385 | LOC_Os01g67540 | AMP-binding enzyme family protein, expressed |
| OsAffx.22797.1.S1_at | 0.464 | 0.365 | 0.325 | 0.506 | LOC_Os01g04070 | Leucine Rich Repeat family protein, expressed |
| Os.47706.2.A1_at | 0.465 | 0.755 | 0.934 | 1.635 | LOC_Os10g03570 | NB-ARC domain containing protein |
| Os.11749.1.S1_at | 0.465 | 2.770 | 1.425 | 3.107 | LOC_Os03g08860 | FH protein interacting protein FIP1, putative, expressed |
| Os.49816.1.S1_at | 0.466 | 2.137 | 1.860 | 0.726 | LOC_Os04g51040 | wall-associated kinase 1, putative, expressed |
| Os.54602.1.S1_s_at | 0.466 | 0.754 | 1.026 | 1.288 | LOC_Os04g32850 | expressed protein |
| Os.56999.1.S1_at | 0.467 | 2.037 | 1.361 | 1.473 | LOC_Os03g17940 | PAP2 superfamily protein, expressed |
| Os.41335.2.S1_x_at | 0.467 | 1.705 | 1.725 | 1.418 | LOC_Os01g42030 | ATPase, AAA family protein, expressed |

| ProbeSetID | *OMTN6* -OE[a] | Stress response[b] | | | TIGR Locus ID[c] | Annotation |
|---|---|---|---|---|---|---|
| | | Drought | Salt | Cold | | |
| Os.8266.1.A1_at | 0.468 | 4.728 | 2.364 | 2.060 | LOC_Os03g55800 | Cytochrome P450 74A1, chloroplast precursor, putative, expressed |
| Os.46777.1.S1_at | 0.468 | 0.421 | 0.363 | 0.888 | LOC_Os04g29580 | Calcium binding EGF domain containing protein, expressed |
| OsAffx.28651.1.S1_x_at | 0.468 | 1.429 | 1.527 | 2.905 | LOC_Os07g28850 | Piwi domain containing protein, expressed |
| Os.4416.1.S1_at | 0.469 | 21.096 | 6.069 | 2.925 | LOC_Os08g39840 | Lipoxygenase 7, chloroplast precursor, putative, expressed |
| Os.35433.1.S1_at | 0.470 | 0.402 | 0.165 | 14.371 | LOC_Os04g39320 | expressed protein |
| OsAffx.29230.1.S1_at | 0.470 | 0.547 | 1.955 | 0.823 | LOC_Os08g16030 | expressed protein |
| Os.8136.1.A1_at | 0.471 | 0.921 | 2.511 | 1.132 | LOC_Os06g40030 | D-mannose binding lectin family protein, expressed |
| Os.50127.1.S1_at | 0.471 | 0.877 | 1.261 | 0.433 | LOC_Os04g30030 | Protein kinase domain containing protein |
| OsAffx.12575.1.S1_at | 0.471 | 0.591 | 0.828 | 1.173 | LOC_Os02g50330 | RNA-directed RNA polymerase 2, putative, expressed |
| Os.15570.1.S1_at | 0.471 | 0.144 | 0.326 | 0.609 | LOC_Os07g32630 | UDP-glucoronosyl and UDP-glucosyl transferase family protein, expressed |
| OsAffx.12626.1.S1_at | 0.472 | 0.189 | 0.222 | 0.751 | LOC_Os02g53620 | CCAAT-binding transcription factor subunit B family protein, expressed |
| Os.52344.1.S1_at | 0.472 | 0.469 | 0.676 | 0.503 | LOC_Os08g26840 | expressed protein |
| Os.55342.1.S1_at | 0.473 | 1.260 | 1.162 | 0.542 | LOC_Os08g33990 | expressed protein |
| Os.12035.1.S1_at | 0.473 | 0.316 | 0.703 | 1.982 | LOC_Os02g50130 | hydrolase, NUDIX family protein, expressed |
| OsAffx.30194.1.S1_x_at | 0.474 | 1.372 | 1.937 | 0.679 | LOC_Os09g38840 | wall-associated kinase-like 1, putative, expressed |
| Os.51853.1.S1_at | 0.474 | 0.834 | 0.626 | 0.863 | LOC_Os06g28480 | Leucine Rich Repeat family protein, expressed |
| Os.35935.2.S1_at | 0.474 | 4.341 | 1.545 | 4.909 | LOC_Os07g31250 | Protein kinase domain containing protein, expressed |
| OsAffx.12740.1.S1_s_at | 0.474 | 1.102 | 0.834 | 1.145 | LOC_Os03g05520 | expressed protein |
| OsAffx.5568.1.S1_at | 0.474 | 0.914 | 0.771 | 1.686 | LOC_Os07g38630 | hypothetical protein |
| Os.10855.1.S1_at | 0.475 | 4.595 | 1.533 | 1.105 | LOC_Os03g56060 | glycosyl transferase, group 2 family protein, expressed |
| Os.46028.1.S1_at | 0.475 | 1.142 | 0.302 | 1.538 | LOC_Os01g06510 | arginyl-tRNA synthetase family protein, expressed |
| Os.53258.1.S1_at | 0.476 | 6.270 | 2.597 | 1.151 | LOC_Os03g60220 | expressed protein |
| OsAffx.29638.1.S1_at | 0.476 | 1.086 | 0.293 | 1.000 | LOC_Os08g42840 | Leucine Rich Repeat family protein |
| OsAffx.13276.1.S1_at | 0.477 | 0.724 | 0.761 | 1.192 | LOC_Os03g40930 | Lipase, putative, expressed |

| ProbeSetID | *OMTN6* -OE[a] | Stress response[b] | | | TIGR Locus ID[c] | Annotation |
|---|---|---|---|---|---|---|
| | | Drought | Salt | Cold | | |
| Os.17479.1.S1_at | 0.477 | 0.854 | 0.526 | 1.884 | LOC_Os01g49320 | Acidic endochitinase precursor, putative, expressed |
| Os.45928.1.S1_at | 0.477 | 0.267 | 0.380 | 1.193 | LOC_Os01g32460 | expressed protein |
| Os.49208.1.S1_at | 0.478 | 0.595 | 0.243 | 1.681 | LOC_Os04g34270 | D-mannose binding lectin family protein, expressed |
| Os.53683.1.S1_at | 0.478 | 3.210 | 1.390 | 1.691 | LOC_Os09g26160 | Receptor family ligand binding region containing protein, expressed |
| OsAffx.24166.1.S1_at | 0.478 | 5.067 | 5.892 | 7.203 | LOC_Os02g08440 | WRKY transcription factor, putative, expressed |
| Os.17020.1.S1_x_at | 0.479 | 3.832 | 2.116 | 1.385 | LOC_Os08g29570 | PDR-type ABC transporter 1, putative, expressed |
| Os.57152.1.S1_at | 0.479 | 13.554 | 1.308 | 5.344 | LOC_Os02g38890 | hypothetical protein |
| OsAffx.7086.1.S1_at | 0.480 | 0.442 | 0.653 | 0.923 | LOC_Os11g11970 | expressed protein |
| Os.48061.1.A1_x_at | 0.480 | 0.323 | 0.575 | 0.946 | LOC_Os06g18670 | Flavonol 3-O-glucosyltransferase 5, putative, expressed |
| OsAffx.28964.1.S1_at | 0.480 | 3.000 | 1.984 | 0.741 | LOC_Os07g49510 | expressed protein |
| Os.35858.2.S1_at | 0.481 | 0.612 | 0.815 | 0.701 | LOC_Os01g44110 | Protein kinase domain containing protein, expressed |
| Os.27279.1.A1_at | 0.481 | 26.757 | 6.295 | 1.570 | LOC_Os03g26870 | WD-40 repeat family protein, putative, expressed |
| Os.36226.1.S1_at | 0.481 | 0.883 | 1.562 | 1.105 | LOC_Os07g03030 | expressed protein |
| Os.54784.1.S1_at | 0.481 | 1.397 | 0.852 | 0.949 | LOC_Os05g38460 | Myb-like DNA-binding domain containing protein, expressed |
| OsAffx.24703.1.S1_s_at | 0.482 | 3.028 | 3.992 | 0.805 | LOC_Os02g43540 | expressed protein |
| Os.54472.1.S1_at | 0.482 | 0.210 | 0.609 | 0.551 | LOC_Os07g32060 | UDP-glucoronosyl and UDP-glucosyl transferase family protein, expressed |
| Os.10591.1.S1_at | 0.482 | 2.406 | 1.002 | 0.904 | LOC_Os04g50790 | Aldose reductase-related protein, putative, expressed |
| Os.6876.1.S1_at | 0.483 | 2.758 | 0.977 | 3.336 | LOC_Os04g38950 | Anthranilate synthase component II, putative, expressed |
| Os.37565.1.S1_x_at | 0.484 | 1.609 | 0.400 | 9.052 | LOC_Os05g25770 | WRKY DNA binding domain containing protein, expressed |
| Os.10391.1.S1_a_at | 0.484 | 3.638 | 1.705 | 1.249 | LOC_Os05g51630 | early-responsive to dehydration protein, putative, expressed |
| Os.1563.1.S1_at | 0.485 | 1.144 | 1.108 | 0.994 | LOC_Os01g22370 | Peroxidase family protein, expressed |
| OsAffx.26488.1.S1_at | 0.485 | 0.967 | 0.629 | 1.186 | LOC_Os04g46730 | Pollen thioesterase, putative, expressed |
| Os.27412.1.S1_at | 0.487 | 0.446 | 0.633 | 0.947 | LOC_Os02g42370 | Leucine Rich Repeat family protein, expressed |
| OsAffx.13993.1.S1_at | 0.487 | 1.238 | 1.103 | 1.270 | LOC_Os04g27100 | WRKY transcription factor 19, putative |

| ProbeSetID | *OMTN6* -OE[a] | Stress response[b] | | | TIGR Locus ID[c] | Annotation |
|---|---|---|---|---|---|---|
| | | Drought | Salt | Cold | | |
| Os.31344.1.S1_at | 0.487 | 1.964 | 1.596 | 0.534 | LOC_Os01g63970 | sialyltransferase, putative, expressed |
| Os.9212.1.S1_at | 0.487 | 0.565 | 0.756 | 0.722 | LOC_Os07g22930 | Granule-bound starch synthase 1b, chloroplast precursor, putative, expressed |
| Os.30124.1.S1_x_at | 0.487 | 1.323 | 1.129 | 1.070 | LOC_Os10g30880 | expressed protein |
| Os.49819.1.S1_at | 0.487 | 1.462 | 2.590 | 3.825 | LOC_Os11g36200 | receptor kinase, putative, expressed |
| Os.6089.1.S1_at | 0.488 | 0.285 | 0.264 | 1.267 | LOC_Os04g15920 | mannitol dehydrogenase, putative, expressed |
| Os.6363.1.S1_at | 0.488 | 0.867 | 3.866 | 1.141 | LOC_Os10g38080 | Subtilisin N-terminal Region family protein, expressed |
| Os.12421.1.S1_at | 0.488 | 0.378 | 0.265 | 0.724 | LOC_Os11g42960 | plant integral membrane protein TIGR01569 containing protein, expressed |
| Os.38984.1.S1_at | 0.488 | 1.139 | 0.861 | 0.877 | LOC_Os01g23580 | Pyrophosphate-energized vacuolar membrane proton pump, putative, expressed |
| Os.5431.1.S1_at | 0.488 | 0.931 | 0.808 | 1.594 | LOC_Os04g49690 | protein kinase family protein, putative, expressed |
| Os.50881.1.S1_x_at | 0.489 | 0.608 | 1.184 | 1.543 | LOC_Os09g39650 | Protein kinase domain containing protein, expressed |
| Os.10500.1.S1_at | 0.489 | 1.171 | 0.831 | 1.687 | LOC_Os05g39610 | Exo70 exocyst complex subunit family protein, expressed |
| Os.5154.1.S1_at | 0.489 | 0.995 | 0.626 | 1.241 | LOC_Os07g49080 | Phytochelatin synthetase-like conserved region family protein, expressed |
| Os.37798.1.S1_at | 0.489 | 2.318 | 2.725 | 1.274 | LOC_Os01g11460 | Zinc finger, C3HC4 type family protein, expressed |
| Os.46287.1.S1_a_at | 0.489 | 2.099 | 1.490 | 2.182 | LOC_Os05g40770 | Protein kinase domain containing protein, expressed |
| Os.57191.1.S1_at | 0.489 | 16.608 | 9.364 | 4.114 | LOC_Os06g05470 | expressed protein |
| OsAffx.29832.2.S1_at | 0.489 | 0.615 | 0.214 | 0.957 | LOC_Os09g13440 | expressed protein |
| Os.37006.1.S1_at | 0.490 | 4.111 | 3.023 | 1.686 | LOC_Os06g11660 | Phosphate-induced protein 1 conserved region containing protein, expressed |
| Os.55746.1.S1_at | 0.490 | 3.472 | 0.778 | 0.778 | LOC_Os11g28530 | Terpene synthase family, metal binding domain containing protein, expressed |
| OsAffx.27130.1.S1_at | 0.490 | 1.204 | 0.698 | 1.401 | LOC_Os05g33000 | zinc finger family protein, putative, expressed |
| Os.18023.1.S1_at | 0.490 | 0.669 | 0.633 | 1.171 | LOC_Os11g36030 | AT hook motif family protein, expressed |
| Os.27407.1.A1_at | 0.490 | 1.041 | 0.856 | 1.602 | LOC_Os01g62200 | expressed protein |
| Os.5093.1.S1_at | 0.490 | 3.541 | 1.555 | 1.380 | LOC_Os08g38210 | Helix-loop-helix DNA-binding domain containing protein, expressed |
| Os.5194.1.S1_x_at | 0.490 | 0.462 | 0.573 | 0.925 | LOC_Os01g66110 | dehydration-responsive protein, putative, expressed |

| ProbeSetID | *OMTN6* -OE[a] | Stress response[b] | | | TIGR Locus ID[c] | Annotation |
|---|---|---|---|---|---|---|
| | | Drought | Salt | Cold | | |
| Os.21875.1.S1_at | 0.491 | 0.756 | 0.864 | 1.169 | LOC_Os01g74340 | RNA recognition motif family protein |
| OsAffx.25832.1.S1_at | 0.492 | 0.484 | 1.325 | 1.302 | LOC_Os04g02030 | NB-ARC domain containing protein |
| Os.25104.2.S1_at | 0.493 | 0.978 | 0.702 | 1.151 | LOC_Os02g52280 | expressed protein |
| Os.6284.1.S1_at | 0.493 | 1.028 | 0.799 | 1.434 | LOC_Os03g04710 | expressed protein |
| Os.12416.1.S1_at | 0.493 | 1.003 | 1.010 | 1.084 | LOC_Os11g32650 | Chalcone synthase, putative, expressed |
| Os.22086.1.S1_at | 0.493 | 0.129 | 0.779 | 1.147 | LOC_Os07g48020 | Peroxidase 2 precursor, putative, expressed |
| Os.12412.1.S1_at | 0.494 | 0.421 | 0.410 | 1.001 | LOC_Os02g16500 | expressed protein |
| OsAffx.13437.1.S1_at | 0.494 | 6.744 | 1.294 | 2.329 | LOC_Os03g50280 | expressed protein |
| Os.17941.2.S1_x_at | 0.494 | 0.730 | 0.569 | 0.768 | LOC_Os03g03590 | K+ efflux antiporter, putative, expressed |
| Os.52465.1.S1_at | 0.494 | 0.428 | 0.559 | 0.518 | LOC_Os07g36210 | expressed protein |
| Os.55674.1.S1_at | 0.495 | 3.286 | 1.114 | 1.829 | LOC_Os05g46830 | expressed protein |
| Os.14313.1.S1_s_at | 0.495 | 0.854 | 0.710 | 0.718 | LOC_Os01g61620 | protein kinase family protein, putative, expressed |
| Os.16142.1.S1_at | 0.496 | 3.188 | 1.268 | 1.483 | LOC_Os03g31510 | Cystatin, putative, expressed |
| OsAffx.26114.1.S1_at | 0.497 | 1.178 | 0.654 | 1.509 | LOC_Os04g23440 | Helix-loop-helix DNA-binding domain containing protein |
| Os.10861.1.S1_at | 0.498 | 2.865 | 1.730 | 3.323 | LOC_Os11g08100 | Eukaryotic aspartyl protease family protein, expressed |
| Os.8789.1.S1_at | 0.498 | 1.209 | 0.826 | 1.877 | LOC_Os08g44850 | C2 domain-containing protein, putative, expressed |
| Os.53236.1.S1_at | 0.498 | 1.354 | 0.593 | 1.832 | LOC_Os02g43170 | B-box zinc finger family protein, expressed |
| Os.5768.1.S1_at | 0.499 | 2.041 | 2.326 | 1.237 | LOC_Os09g15320 | Ubiquitin-conjugating enzyme E2 M, putative, expressed |
| Os.34952.1.S1_at | 0.500 | 1.481 | 1.136 | 1.623 | LOC_Os01g55610 | POT family protein, expressed |
| Os.56918.1.S1_at | 0.500 | 0.691 | 0.833 | 3.404 | LOC_Os03g62330 | expressed protein |
| Os.18395.1.S1_s_at | 0.500 | 1.088 | 0.581 | 0.567 | LOC_Os06g15620 | GAST1 protein precursor, putative, expressed |

NOTE: All values in the table are expression level change folds (transgenic/WT, or stress/normal)(mean of the repeats).The folds higher than 2 were indicated by red colour, and the folds lower than 0.5 were indicated by green colour.

[a]Expression level change folds in the *OMTN6*-OE plants.

[b]Expression profile of the genes under drought, salt and cold stress was download from the GEO database (http://www.ncbi.nlm.nih.gov/geo/, accession number: GSE6901).
[c]The locus ID was download from Rice Annotation Project (http://rice.plantbiology.msu.edu/).

**Supplementary Table S8. List of genes with consistent expression change patterns in the *OMTN2*, *OMTN3*, *OMTN4*, and *OMTN6* overexpressors.**

| ProbeSetID | *OMTN2* -OE[a] | *OMTN3* -OE[b] | *OMTN4* -OE[c] | *OMTN6* -OE[d] | Stress response[e] | | | TIGR Locus ID[f] | Annotation |
|---|---|---|---|---|---|---|---|---|---|
| | | | | | Drought | Salt | Cold | | |
| Os.10266.1.S1_at | 4.308 | 7.733 | 7.489 | 5.037 | 0.167 | 0.362 | 0.630 | LOC_Os03g43100 | expressed protein |
| Os.17047.1.A1_at | 3.627 | 5.360 | 6.827 | 2.591 | 0.564 | 1.205 | 0.910 | LOC_Os07g18230 | Protein kinase domain containing protein, expressed |
| Os.7991.1.S1_at | 2.840 | 2.373 | 4.348 | 8.962 | 3.486 | 1.811 | 1.730 | LOC_Os04g41620 | Endochitinase A precursor, putative, expressed |
| OsAffx.21073.1.S1_at | 3.022 | 3.826 | 3.856 | 2.307 | 0.559 | 0.401 | 0.494 | LOC_Os07g02620 | NB-ARC domain containing protein |
| Os.27497.1.S1_at | 2.506 | 2.432 | 3.781 | 2.676 | 0.191 | 0.428 | 0.681 | LOC_Os12g12390 | transposon protein, putative, CACTA, En/Spm sub-class, expressed |
| Os.34982.1.A1_at | 6.415 | 3.611 | 3.517 | 8.067 | 0.348 | 0.828 | 0.751 | LOC_Os04g17660 | Rhodanese-like domain containing protein, expressed |
| Os.10556.1.S1_at | 3.081 | 3.495 | 2.490 | 4.682 | 8.593 | 9.683 | 0.495 | LOC_Os02g15860 | expressed protein |
| Os.11707.1.A1_at | 5.369 | 4.141 | 2.359 | 2.544 | 0.143 | 1.445 | 0.298 | LOC_Os03g54130 | Papain family cysteine protease containing protein, expressed |
| Os.1385.1.S1_at | 2.370 | 2.448 | 2.328 | 3.142 | 1.305 | 1.446 | 1.301 | LOC_Os01g71670 | Glucan endo-1,3-beta-glucosidase GII precursor, putative, expressed |
| Os.11065.1.A1_at | 2.329 | 7.766 | 2.142 | 12.391 | 1.191 | 0.512 | 1.153 | LOC_Os03g19600 | retrotransposon protein, putative, Ty3-gypsy subclass, expressed |
| Os.50470.1.S1_at | 2.996 | 3.611 | 2.136 | 2.627 | 3.270 | 2.470 | 0.793 | LOC_Os01g71310 | Cytokinin dehydrogenase 1 precursor, putative, expressed |
| OsAffx.11788.1.S1_at | 0.246 | 0.089 | 0.072 | 0.155 | 0.376 | 0.313 | 0.649 | LOC_Os01g68460 | DC1 domain-containing protein, putative, expressed |
| Os.54961.1.S1_at | 0.456 | 0.182 | 0.120 | 0.121 | 5.200 | 1.663 | 2.109 | LOC_Os05g46350 | IQ calmodulin-binding motif family protein, expressed |
| Os.12452.1.S1_s_at | 0.333 | 0.145 | 0.138 | 0.333 | 7.189 | 4.123 | 1.011 | LOC_Os08g36910 | Alpha-amylase isozyme 3D precursor, putative, expressed |
| Os.18463.1.S1_at | 0.421 | 0.183 | 0.139 | 0.117 | 9.807 | 2.097 | 5.040 | LOC_Os05g37660 | AER, putative, expressed |
| Os.49634.1.S1_x_at | 0.247 | 0.131 | 0.145 | 0.175 | 2.255 | 3.515 | 5.648 | LOC_Os01g55510 | Dynein light chain type 1 family protein, expressed |
| OsAffx.17348.1.S1_at | 0.281 | 0.197 | 0.162 | 0.176 | 0.699 | 0.928 | 2.325 | LOC_Os08g35590 | hypothetical protein |
| Os.53407.1.S1_at | 0.414 | 0.246 | 0.168 | 0.155 | 3.573 | 1.288 | 0.763 | LOC_Os08g06210 | expressed protein |
| OsAffx.12733.1.S1_at | 0.280 | 0.212 | 0.172 | 0.215 | 1.472 | 1.239 | 3.033 | LOC_Os03g04740 | expressed protein |

| ProbeSetID | *OMTN2* -OE[a] | *OMTN3* -OE[b] | *OMTN4* -OE[c] | *OMTN6* -OE[d] | Stress response[e] | | | TIGR Locus ID[f] | Annotation |
|---|---|---|---|---|---|---|---|---|---|
| | | | | | Drought | Salt | Cold | | |
| Os.12201.2.S1_at | 0.246 | 0.208 | 0.175 | 0.115 | 2.702 | 2.693 | 0.977 | LOC_Os09g27820 | 1-aminocyclopropane-1-carboxylate oxidase 1, putative, expressed |
| OsAffx.24782.1.S1_s_at | 0.207 | 0.215 | 0.179 | 0.244 | 3.917 | 1.965 | 2.304 | LOC_Os02g48320 | DNA-binding protein, putative, expressed |
| OsAffx.14201.1.S1_at | 0.286 | 0.209 | 0.183 | 0.164 | 0.337 | 0.136 | 0.472 | LOC_Os04g39360 | heavy metal-associated domain containing protein, expressed |
| Os.32455.1.S1_at | 0.159 | 0.184 | 0.202 | 0.265 | 1.276 | 0.980 | 1.156 | LOC_Os01g54670 | expressed protein |
| Os.51757.1.S1_at | 0.234 | 0.443 | 0.203 | 0.226 | 1.405 | 1.042 | 0.813 | LOC_Os06g26270 | expressed protein |
| Os.15701.1.S1_x_at | 0.387 | 0.239 | 0.207 | 0.256 | 0.893 | 1.201 | 1.459 | LOC_Os07g47350 | Potassium transporter 7, putative, expressed |
| OsAffx.26389.1.S1_at | 0.298 | 0.153 | 0.208 | 0.198 | 0.619 | 0.619 | 0.952 | LOC_Os04g39300 | heavy metal-associated domain containing protein, expressed |
| Os.52661.1.S1_at | 0.343 | 0.245 | 0.217 | 0.278 | 1.003 | 0.490 | 3.697 | LOC_Os11g44680 | expressed protein |
| Os.27494.1.S1_at | 0.280 | 0.201 | 0.217 | 0.286 | 0.474 | 0.665 | 1.051 | LOC_Os07g41200 | expressed protein |
| Os.15706.1.S1_a_at | 0.461 | 0.266 | 0.220 | 0.279 | 1.408 | 0.692 | 1.753 | LOC_Os01g47580 | Lipid phosphate phosphatase 2, putative, expressed |
| Os.28301.1.S1_at | 0.188 | 0.242 | 0.221 | 0.411 | 0.225 | 0.520 | 1.377 | LOC_Os07g05360 | Photosystem II 10 kDa polypeptide, chloroplast precursor, putative, expressed |
| Os.27688.1.A1_at | 0.136 | 0.214 | 0.231 | 0.169 | 0.957 | 0.731 | 9.416 | LOC_Os04g21350 | flowering promoting factor-like 1, putative, expressed |
| Os.49322.1.S1_at | 0.159 | 0.166 | 0.231 | 0.318 | 0.511 | 0.660 | 2.426 | LOC_Os09g15050 | Ent-kaurene synthase A, chloroplast precursor, putative, expressed |
| Os.54453.1.S1_at | 0.388 | 0.257 | 0.237 | 0.263 | 0.375 | 0.219 | 1.231 | LOC_Os08g44220 | expressed protein |
| Os.27019.1.S1_at | 0.205 | 0.201 | 0.258 | 0.212 | 3.466 | 2.619 | 0.967 | LOC_Os04g43410 | Glycosyl hydrolase family 1 protein, expressed |
| Os.22731.1.S1_at | 0.299 | 0.459 | 0.259 | 0.280 | 9.240 | 14.323 | 0.888 | LOC_Os03g16020 | 17.4 kDa class I heat shock protein, putative, expressed |
| Os.27011.1.A1_s_at | 0.235 | 0.268 | 0.263 | 0.329 | 0.551 | 0.802 | 1.551 | LOC_Os01g04550 | serine/threonine protein kinase, putative, expressed |
| OsAffx.23406.1.S1_at | 0.435 | 0.276 | 0.294 | 0.321 | 0.836 | 0.954 | 1.289 | LOC_Os01g26300 | wall-associated kinase 1, putative, expressed |
| OsAffx.7606.1.S1_at | 0.119 | 0.227 | 0.307 | 0.323 | 1.419 | 4.132 | 1.940 | LOC_Os12g15680 | Multicopper oxidase family protein, expressed |
| Os.47945.1.A1_at | 0.428 | 0.378 | 0.314 | 0.281 | 0.810 | 0.490 | 0.792 | LOC_Os06g37510 | senescence-associated protein, putative, expressed |
| Os.37773.1.S1_at | 0.317 | 0.312 | 0.318 | 0.349 | 1.643 | 7.595 | 2.434 | LOC_Os03g16030 | 17.4 kDa class I heat shock protein, putative, expressed |

| ProbeSetID | *OMTN2* -OE[a] | *OMTN3* -OE[b] | *OMTN4* -OE[c] | *OMTN6* -OE[d] | Stress response[e] | | | TIGR Locus ID[f] | Annotation |
|---|---|---|---|---|---|---|---|---|---|
| | | | | | Drought | Salt | Cold | | |
| Os.56988.1.S1_at | 0.442 | 0.361 | 0.326 | 0.340 | 1.338 | 0.632 | 1.368 | LOC_Os03g53730 | flavodoxin family protein, expressed |
| Os.54385.1.S1_at | 0.420 | 0.299 | 0.326 | 0.352 | 0.889 | 0.715 | 1.847 | LOC_Os04g01320 | D-mannose binding lectin family protein, expressed |
| Os.53009.1.A1_x_at | 0.344 | 0.317 | 0.331 | 0.295 | 0.078 | 0.444 | 0.883 | LOC_Os02g54640 | Receptor family ligand binding region containing protein, expressed |
| Os.2211.1.S1_at | 0.215 | 0.221 | 0.335 | 0.359 | 0.568 | 0.573 | 1.727 | LOC_Os08g09010 | Germin-like protein subfamily 1 member 11 precursor, putative, expressed |
| OsAffx.11789.1.S1_s_at | 0.441 | 0.313 | 0.337 | 0.372 | 0.092 | 0.083 | 0.712 | LOC_Os01g68470 | hypothetical protein |
| OsAffx.3951.1.S1_at | 0.418 | 0.434 | 0.341 | 0.413 | 0.702 | 0.226 | 0.806 | LOC_Os04g30340 | wall-associated kinase 2, putative |
| Os.55254.1.S1_at | 0.470 | 0.444 | 0.341 | 0.463 | 1.036 | 1.313 | 1.133 | LOC_Os06g10130 | expressed protein |
| OsAffx.19473.1.S1_at | 0.305 | 0.267 | 0.341 | 0.405 | 1.539 | 1.309 | 1.893 | LOC_Os12g03740 | F-box domain containing protein, expressed |
| Os.5905.1.S1_at | 0.275 | 0.325 | 0.343 | 0.347 | 0.897 | 0.223 | 0.877 | LOC_Os06g32990 | Peroxidase family protein, expressed |
| Os.26938.1.A1_at | 0.479 | 0.437 | 0.346 | 0.300 | 0.149 | 0.409 | 0.701 | LOC_Os02g42150 | Calcium binding EGF domain containing protein, expressed |
| Os.6042.1.S1_at | 0.281 | 0.343 | 0.357 | 0.267 | 4.173 | 1.419 | 0.716 | LOC_Os07g25810 | transposon protein, putative, CACTA, En/Spm sub-class, expressed |
| Os.33316.1.S1_at | 0.175 | 0.302 | 0.357 | 0.289 | 3.624 | 0.960 | 1.115 | LOC_Os01g34560 | very-long-chain fatty acid condensing enzyme, putative, expressed |
| Os.17112.1.S1_at | 0.360 | 0.390 | 0.359 | 0.310 | 15.064 | 12.497 | 1.767 | LOC_Os03g18030 | oxidoreductase, 2OG-Fe oxygenase family protein, expressed |
| Os.50346.1.S1_at | 0.331 | 0.392 | 0.366 | 0.426 | 3.223 | 1.628 | 1.876 | LOC_Os05g04680 | expressed protein |
| OsAffx.15812.1.S1_at | 0.178 | 0.148 | 0.366 | 0.136 | 1.455 | 1.000 | 1.182 | LOC_Os06g38120 | expressed protein |
| OsAffx.30204.1.S1_at | 0.431 | 0.449 | 0.366 | 0.460 | 1.521 | 0.827 | 1.311 | LOC_Os09g39190 | Copine family protein, expressed |
| OsAffx.29016.1.S1_at | 0.380 | 0.367 | 0.367 | 0.411 | 1.325 | 1.152 | 1.424 | LOC_Os08g03600 | magnesium transporter CorA, putative, expressed |
| Os.8202.1.S1_at | 0.387 | 0.372 | 0.367 | 0.388 | 2.181 | 1.704 | 1.680 | LOC_Os12g37560 | phospholipase C, putative, expressed |
| Os.39087.1.S1_at | 0.432 | 0.454 | 0.368 | 0.436 | 1.762 | 1.344 | 1.987 | LOC_Os01g14550 | Pathogen-related protein, putative, expressed |
| Os.30909.1.S1_at | 0.187 | 0.288 | 0.374 | 0.413 | 0.504 | 1.065 | 0.187 | LOC_Os01g09190 | expressed protein |

| ProbeSetID | *OMTN2* -OE[a] | *OMTN3* -OE[b] | *OMTN4* -OE[c] | *OMTN6* -OE[d] | Stress response[e] | | | TIGR Locus ID[f] | Annotation |
|---|---|---|---|---|---|---|---|---|---|
| | | | | | Drought | Salt | Cold | | |
| Os.10245.1.S1_at | 0.330 | 0.375 | 0.375 | 0.359 | 1.135 | 2.508 | 1.681 | LOC_Os04g37700 | expressed protein |
| OsAffx.17220.1.S1_at | 0.318 | 0.328 | 0.377 | 0.376 | 3.300 | 3.450 | 0.550 | LOC_Os08g29040 | Protein kinase domain containing protein |
| Os.30572.1.S1_at | 0.265 | 0.389 | 0.378 | 0.423 | 1.216 | 0.811 | 0.634 | LOC_Os01g50610 | SAM dependent carboxyl methyltransferase family protein, expressed |
| Os.23977.1.S1_at | 0.480 | 0.391 | 0.381 | 0.369 | 0.378 | 0.538 | 0.763 | LOC_Os03g43510 | expressed protein |
| Os.57191.1.S1_at | 0.430 | 0.305 | 0.387 | 0.489 | 16.608 | 9.364 | 4.114 | LOC_Os06g05470 | expressed protein |
| OsAffx.22380.1.S1_at | 0.442 | 0.398 | 0.392 | 0.323 | 1.580 | 0.910 | 3.608 | LOC_Os07g39680 | XYPPX repeat family protein, expressed |
| Os.52699.1.S1_at | 0.353 | 0.352 | 0.393 | 0.424 | 0.928 | 0.737 | 1.267 | LOC_Os04g55420 | Leucine Rich Repeat family protein, expressed |
| Os.50572.1.S1_at | 0.243 | 0.234 | 0.399 | 0.350 | 1.092 | 0.941 | 1.701 | LOC_Os12g36920 | calmodulin-binding protein, putative, expressed |
| OsAffx.8290.1.S1_at | 0.257 | 0.460 | 0.400 | 0.370 | 1.580 | 1.037 | 0.370 | LOC_Os10g17960 | receptor-like protein kinase homolog RK20-1, putative, expressed |
| Os.11322.1.S1_at | 0.323 | 0.345 | 0.402 | 0.427 | 1.168 | 0.685 | 1.311 | LOC_Os04g50950 | POT family protein, expressed |
| OsAffx.21092.2.S1_at | 0.426 | 0.371 | 0.405 | 0.320 | 0.326 | 0.874 | 0.407 | LOC_Os01g26310 | expressed protein |
| Os.55829.1.S1_at | 0.330 | 0.378 | 0.418 | 0.438 | 3.196 | 1.379 | 2.648 | LOC_Os05g30760 | hydrolase, alpha/beta fold family protein, expressed |
| Os.171.1.S1_at | 0.394 | 0.386 | 0.430 | 0.366 | 0.797 | 1.090 | 1.302 | LOC_Os01g47070 | Acidic endochitinase precursor, putative, expressed |
| Os.10855.1.S1_at | 0.474 | 0.458 | 0.435 | 0.475 | 4.595 | 1.533 | 1.105 | LOC_Os03g56060 | glycosyl transferase, group 2 family protein, expressed |
| Os.30376.1.S1_at | 0.457 | 0.358 | 0.435 | 0.265 | 1.004 | 1.603 | 1.411 | LOC_Os01g02130 | expressed protein |
| Os.47445.1.S1_at | 0.291 | 0.443 | 0.445 | 0.265 | 1.503 | 1.340 | 1.357 | LOC_Os02g26210 | flowering promoting factor-like 1, putative, expressed |
| Os.52228.1.S1_at | 0.275 | 0.303 | 0.445 | 0.319 | 0.224 | 0.400 | 0.903 | LOC_Os11g36000 | Leucine Rich Repeat family protein, expressed |
| Os.17479.1.S1_at | 0.485 | 0.376 | 0.445 | 0.477 | 0.854 | 0.526 | 1.884 | LOC_Os01g49320 | Acidic endochitinase precursor, putative, expressed |
| Os.19369.1.S1_at | 0.455 | 0.380 | 0.454 | 0.351 | 1.610 | 1.080 | 0.969 | LOC_Os12g05990 | No apical meristem protein, expressed |
| Os.7678.1.S1_at | 0.493 | 0.329 | 0.455 | 0.415 | 2.222 | 2.997 | 6.049 | LOC_Os03g12500 | Cytochrome P450 74A2, putative, expressed |
| Os.18717.2.S1_at | 0.322 | 0.422 | 0.459 | 0.463 | 1.557 | 3.307 | 0.634 | LOC_Os09g28180 | D-mannose binding lectin family protein, expressed |
| Os.40417.1.A1_at | 0.381 | 0.403 | 0.465 | 0.368 | 0.550 | 0.322 | 1.073 | LOC_Os07g14740 | Harpin-induced protein 1 containing protein, expressed |

| ProbeSetID | *OMTN2* -OE[a] | *OMTN3* -OE[b] | *OMTN4* -OE[c] | *OMTN6* -OE[d] | Stress response[e] | | | TIGR Locus ID[f] | Annotation |
|---|---|---|---|---|---|---|---|---|---|
| | | | | | Drought | Salt | Cold | | |
| Os.53236.1.S1_at | 0.500 | 0.408 | 0.468 | 0.498 | 1.354 | 0.593 | 1.832 | LOC_Os02g43170 | B-box zinc finger family protein, expressed |
| Os.49400.1.S2_s_at | 0.428 | 0.418 | 0.472 | 0.422 | 1.133 | 0.830 | 0.786 | LOC_Os11g11960 | NBS-LRR type disease resistance protein, putative, expressed |
| Os.49973.1.S1_at | 0.465 | 0.388 | 0.473 | 0.393 | 0.879 | 0.436 | 1.258 | LOC_Os03g20210 | Eukaryotic aspartyl protease family protein, expressed |
| Os.18395.1.S1_s_at | 0.348 | 0.440 | 0.474 | 0.500 | 1.088 | 0.581 | 0.567 | LOC_Os06g15620 | GAST1 protein precursor, putative, expressed |
| Os.35433.1.S1_at | 0.233 | 0.390 | 0.478 | 0.470 | 0.402 | 0.165 | 14.37 1 | LOC_Os04g39320 | expressed protein |
| OsAffx.7414.1.S1_at | 0.300 | 0.357 | 0.486 | 0.382 | 0.129 | 0.259 | 0.345 | LOC_Os11g44700 | expressed protein |
| OsAffx.10980.1.S1_at | 0.261 | 0.388 | 0.495 | 0.443 | 1.235 | 1.330 | 3.191 | LOC_Os01g09150 | hypothetical protein |
| Os.46840.1.S1_x_at | 0.170 | 0.288 | 0.500 | 0.431 | 0.338 | 0.425 | 0.884 | LOC_Os10g34700 | expressed protein |

NOTE: All values in the table are expression level change folds (transgenic/WT, or stress/normal)(mean of the repeats).The folds higher than 2 were indicated by red colour, and the folds lower than 0.5 were indicated by green colour.

[a]Expression level change folds in the *OMTN2*-OE plants.

[b]Expression level change folds in the *OMTN3*-OE plants.

[c]Expression level change folds in the *OMTN4*-OE plants.

[d]Expression level change folds in the *OMTN6*-OE plants.

[e]Expression profile of the genes under drought, salt and cold stress was download from the GEO database (http://www.ncbi.nlm.nih.gov/geo/, accession number: GSE6901).

[f]The locus ID was download from Rice Annotation Project (http://rice.plantbiology.msu.edu/).
